# Targeting CDK9-dependent transcriptional addiction: a novel chemoprevention strategy for oral carcinogenesis via adenosine deaminase modulation

**DOI:** 10.1038/s41419-025-08224-5

**Published:** 2025-12-08

**Authors:** Qingwen Zeng, Zhangci Su, Yujia Bai, Wei Li, Bing Wang, Mi Lin, Chao Lv, Bin Cheng, Xiaoan Tao

**Affiliations:** 1 Hospital of Stomatology, Guanghua School of Stomatology, Sun Yat-sen University, Guangzhou, China; 2 Guangdong Provincial Key Laboratory of Stomatology, Guangzhou, China

**Keywords:** Oral cancer, Cancer prevention

## Abstract

Oncogenic dysregulation of transcription can entail defective control of gene expression and drive tumor initiation. This addiction to certain transcriptional programs provides opportunities to prevent carcinogenesis, and targeting transcriptional cyclin-dependent kinases (tCDKs) holds promise to show clinical benefit. Here, we firstly reported that transcriptional addiction existed in the process of oral mucosal carcinogenesis and high expression of CDK9 contributed to transcriptional dysregulation. CDK9 inhibition paused RNA Pol II transcription cycle to induce cell apoptosis in vitro and in vivo, effectively hampering carcinogenesis in 4-NQO-induced mouse models. Mechanically, targeting CDK9 decreased adenosine deaminase (ADA) expression and suppressed ADA activity, impacting on the enzymatic conversion of adenosine to inosine and resultantly caused cell apoptosis. Our findings indicate the important roles of the CDK9-dependent transcriptional addiction in precancerous stage of oral mucosal carcinogenesis, and come up with novel strategy to prevent malignant transformation of precancerous diseases.

**Figure:**
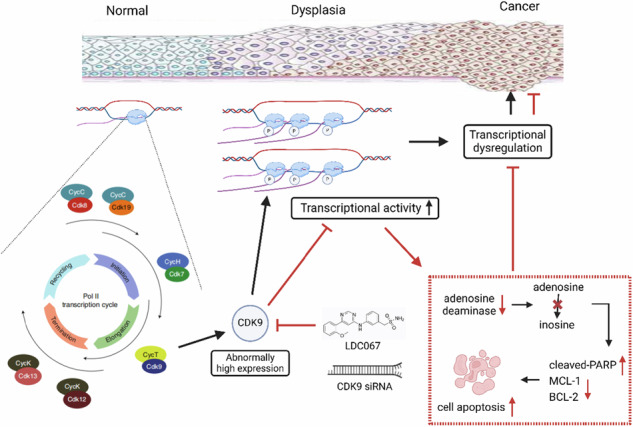
Model diagram for the role of CDK9-dependent transcriptional addiction in oral carcinogenesis. Transcriptional addiction is an important feature in the process of oral mucosal carcinogenesis. Targeting this CDK9-dependent transcriptional addiction induces cell apoptosis by downregulating ADA, and thereby interfering the enzymatic conversion of adenosine to inosine to hamper oral mucosal carcinogenesis.

Model diagram for the role of CDK9-dependent transcriptional addiction in oral carcinogenesis. Transcriptional addiction is an important feature in the process of oral mucosal carcinogenesis. Targeting this CDK9-dependent transcriptional addiction induces cell apoptosis by downregulating ADA, and thereby interfering the enzymatic conversion of adenosine to inosine to hamper oral mucosal carcinogenesis.

## Introduction

Gene dysregulation is a hallmark of cancer, and genetic alterations invariably lead to dysregulated transcriptional programs [1]. In cancer, the resultant dependency on the core-transcriptional machinery, coined ‘transcriptional addiction’, drives tumor sensitive to perturbation of transcription [2]. Thus, targeting transcriptional cyclin-dependent kinases (tCDKs) and associated proteins hold promise for the prophylaxis and treatment of cancer. Currently, quite a few tCDKs inhibitors have enter clinical trials [3], such as samuraciclib in patients with advanced malignancies (NCT03363893) [4], and dinaciclib in patients with hematologic malignancies (NCT02684617) [5].

tCDKs are a family of serine/threonine kinases, including CDK7, CDK8, CDK9, CDK12, CDK13 and CDK19. They orchestrate the transcription cycle by phosphorylating the carboxyterminal domain (CTD) of RPB1, the largest subunit of RNA polymerase II (RNA Pol II). Patterns of CTD phosphorylation differ at different stages of transcription and allow for the timely recruitment of factors important for mRNA elongation and maturation [6]. In the transcription cycle, RNA Pol II requires recruitment of the CDK9 and cyclin T complex, which phosphorylates Ser2 residue on the CTD of RNA Pol II, to regulate the transition from the pausing to the elongation phase of transcription [7]. Dysregulation of the CDK9 pathway has been observed in a variety of human tumors, such as osteosarcoma [8], cervical cancer [9], hepatocellular carcinoma [10] and hematological malignancies [11]. Accordingly, targeting CDK9, or blocking its pathway of transcription, offers a potentially effective therapy for malignant tumors. However, it remains an open question about whether targeting CDK9 could function in precancerous stage to hamper carcinogenesis.

Many cancers have a natural history of progression, evolving from dysplasia to hyperplasia to in situ carcinoma and eventually to a malignant invasive tumor [12]. Typical precancerous stages exist in multiple kinds of tumors, such as oral leukoplakia (OLK), colon polyps, COPD and ductal carcinoma in situ of breast. The changes in gene expression often occur before histopathological changes [13]. These precancerous conditions could show some molecular and phenotypic properties that characterize the cancer, and harbor typical features of transcriptional addiction, including overexpression of oncogenes and underexpression of tumor suppressor genes induced by epigenetic changes such as DNA methylation and histone acetylation [14]. Therefore, targeting transcriptional addiction in precancerous diseases may help to reverse the malignant transformation of related diseases.

Currently, the 4-Nitroquinoline N-oxide (4-NQO)-induced mouse model remains the most ideal model that accurately reflects the process of oral squamous cell carcinoma (OSCC) with a typical precancerous stage (OLK), promoting the development of new targets and new drugs to prevent oral mucosal carcinogenesis [15, 16]. It helps to further elucidate the mechanism of transcriptional addiction in the tumor initiation stage. Considering that OSCC is a common type of cancers and causes hundreds of thousands of deaths each year [17], there is a critical clinical need to understand the gene features in oral mucosal carcinogenesis and to come up with more effective strategies to hamper the happening of OSCC.

Adenosine deaminase (ADA) is a housekeeping enzyme crucial in purine metabolism. Acting as catalyst, ADA deaminates adenosine to inosine thereby regulating the intracellular and extracellular concentrations of adenosine and inosine [18]. Both adenosine and inosine are low-weight molecules and participate in several signaling processes. Adenosine serves as a constituent of ATP and ADP, regulating the energy balance and metabolism [19]. Inosine can be metabolized into hypoxanthine, xanthine, and uric acid, modulating oxidative stress and inflammatory responses [20]. Changes in the purine metabolism, especially the conversion of adenosine to inosine, have been involved in diseases such as cardiovascular diseases [21], cancers, and neurodegenerative diseases [22]. This suggests ADA as a therapeutic target in various diseases.

In this study, we firstly identified that CDK9-dependent transcriptional addiction existed in the process of oral mucosal carcinogenesis. We found that CDK9 inhibition could effectively suppress OSCC progress both in vitro and in vivo. Mechanistically, CDK9 inhibition hampered oral mucosal carcinogenesis by downregulating ADA expression to impact on adenosine-inosine metabolism. These findings highlight the important roles of the CDK9-dependent transcriptional addiction in precancerous stage of oral mucosal carcinogenesis, and come up with more effective antitumor strategy in the initial stage of cancers.

## Results

### Transcriptional dysregulation exists in the process of oral mucosal carcinogenesis

**Fig. 1:**
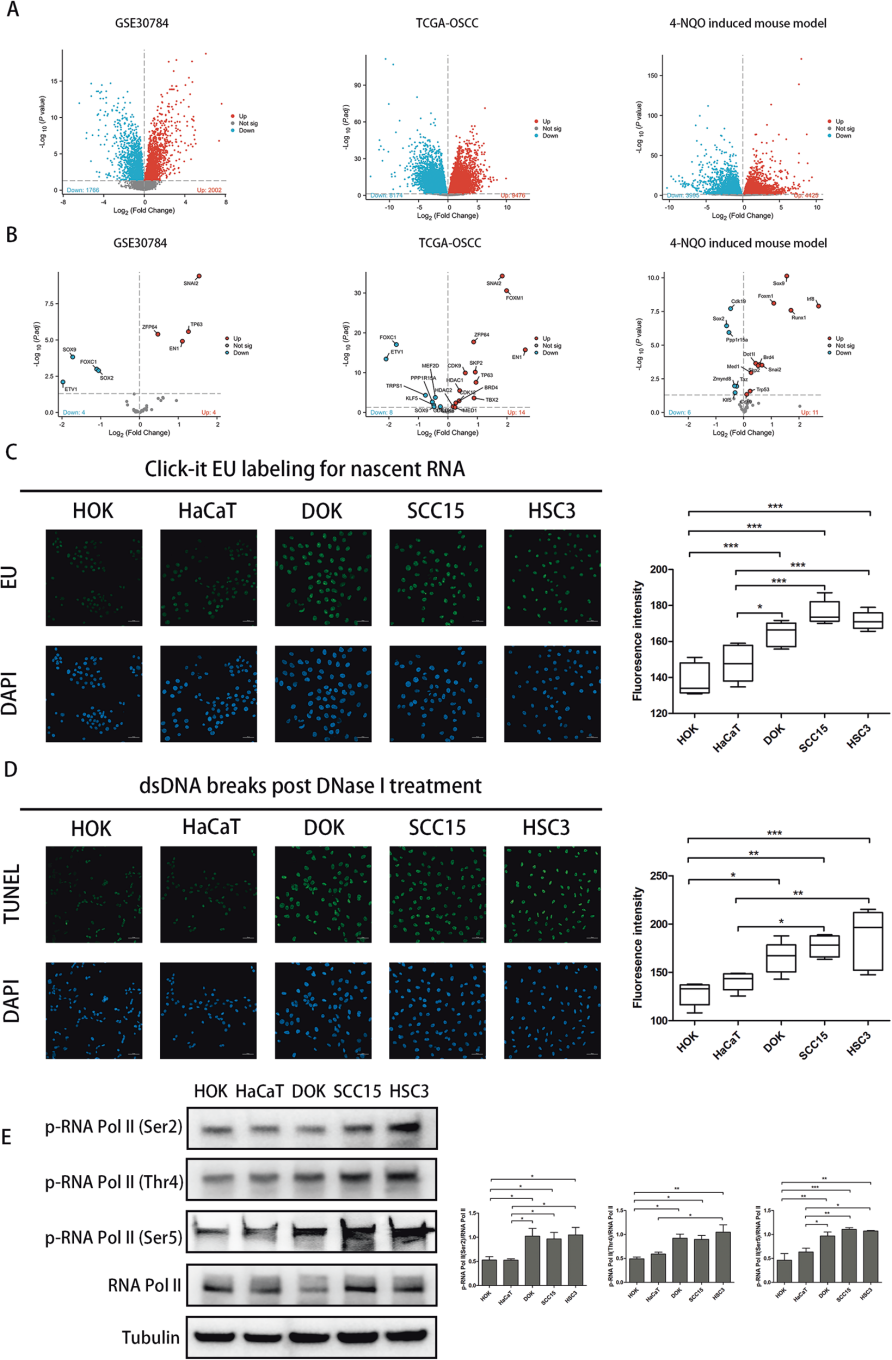
Transcriptional dysregulation is a typical feature in the carcinogenesis of oral mucosa. **A** Volcano plot depicting differentially expressed genes in GSE30784 dataset, TCGA-OSCC set and 4-NQO induced tongue carcinogenesis mouse model. The numbers of significant differentially expressed upregulated genes (red) or downregulated genes (blue) are displayed at the bottom. **B** Volcano plot depicting differentially expressed ‘transcriptional addiction genes’ in GSE30784 dataset, TCGA-OSCC set and 4-NQO induced tongue carcinogenesis mouse model. **C** Analysis of nascent RNA synthesis in HOK, HaCaT, DOK, SCC15, HSC3 cell lines. Nascent RNA synthesis was detected by using a click-it RNA Alexa fluor 488 imaging kit. EU, 5-ethynyl uridine; DAPI, 4′,6-diamidino-2-phenylindole. **D** Analysis of chromatin accessibility in HOK, HaCaT, DOK, SCC15, HSC3 cell lines. DNase I–treated TUNEL assay was performed. **E** Western blot of p-RNA Pol II (Ser2), p-RNA Pol II (Thr4), p-RNA Pol II (Ser5)in HOK, HaCaT, DOK, SCC15, HSC3. Quantification(right) was measured by the ratio of RNA Pol II phosphorylation at Ser2 relative to total RNA Pol II. (*P < 0.05, **P < 0.01, ***P < 0.001).

Transcription dysregulated state engenders a disproportional reliance on the activity of various components of the core-transcriptional machinery to support tumor growth [2]. To determine whether transcription dysregulated state exists in oral mucosal carcinogenesis, we firstly identified DEGs from three datasets, including GSE30784, TCGA-OSCC and 4-NQO induced tongue carcinogenesis mouse model. The GSE30784 contains 45 healthy control and 17 OLK samples. Differential expression analysis in this dataset yielded 3768 DEGs, among which 2002 were upregulated. In the TCGA-OSCC set (32 paraneoplastic tissues and 362 tumor tissues), 9476 genes were upregulated. For the tongue carcinogenesis mouse model, 6 C57BL/6 mice were fed with 4-NQO (100 mg/L) for 16 weeks. Compared with the control group, the number of upregulated genes was 4425 (Fig. 1A). To some extent, the number of highly expressed genes could reflect the transcriptional activity, so we preliminarily speculated that transcriptional dysregulation occurred in the process of oral mucosal carcinogenesis. Some researchers have identified a series of genes associated with cancer transcriptional addiction and referred these 38 genes as “transcriptional addiction genes” [23]. We searched and organized the roles of these genes in transcription and analyzed their expression in the three platforms. In the GSE30784 datasets, 8 genes (21.1%) were differentially expressed in OLK patients. In the TCGA-OSCC set, 22 genes (57.9%) were differentially expressed in OSCC patients, and in the tongue carcinogenesis mouse model, 16 genes (42.1%) were differentially expressed (Fig. 1B). Especially, we found that most of the upregulated transcriptional addiction genes were able to promote transcription, while most of those downregulated transcriptional addiction genes could inhibit transcription (Table S1). Thus, we assumed that OSCC could be classified as “transcriptionally addicted” cancer, and there existed increasing transcriptional activity during oral mucosal carcinogenesis.

To verify our assumption, we next investigated 5 cell lines and analyzed their newly transcribed RNA levels. As shown in Fig. 1C, the precancerous cell line DOK, the OSCC cell lines SCC15 and HSC3, displayed marked increases in nascent transcripts synthesis compared with normal epidermal cells HOK and HaCaT cells. Moreover, we performed deoxyribonuclease (DNase) I-treated terminal deoxynucleotidyl transferase-mediated deoxyuridine triphosphate nick end labeling (TUNEL) assay and observed a notable increase in chromatin accessibility in DOK, SCC15 and HSC3 cells compared with HOK and HaCaT cells (Fig. 1D). In the transcription cycle, CDK7 phosphorylated RNA Pol II at Ser5 to facilitate promoter escape and CDK9 phosphorylated RNA Pol II at Ser2 and Thr4 to promote transcription elongation. As we expected, the phosphorylation of p-RNA Pol II at Ser2, Thr4 and Ser5 was higher in DOK and OSCC cells (SCC15, HSC3) compared with HOK or HaCaT, which indicated higher activity of RNA Pol II in DOK and OSCC cells (Fig. 1E). Altogether, these results indicated that transcription dysregulation may be a typical feature in different stages of oral mucosal carcinogenesis.

### High expression of CDK9 contributes to transcriptional addiction and associates with poor prognosis of OSCC

**Fig. 2:**
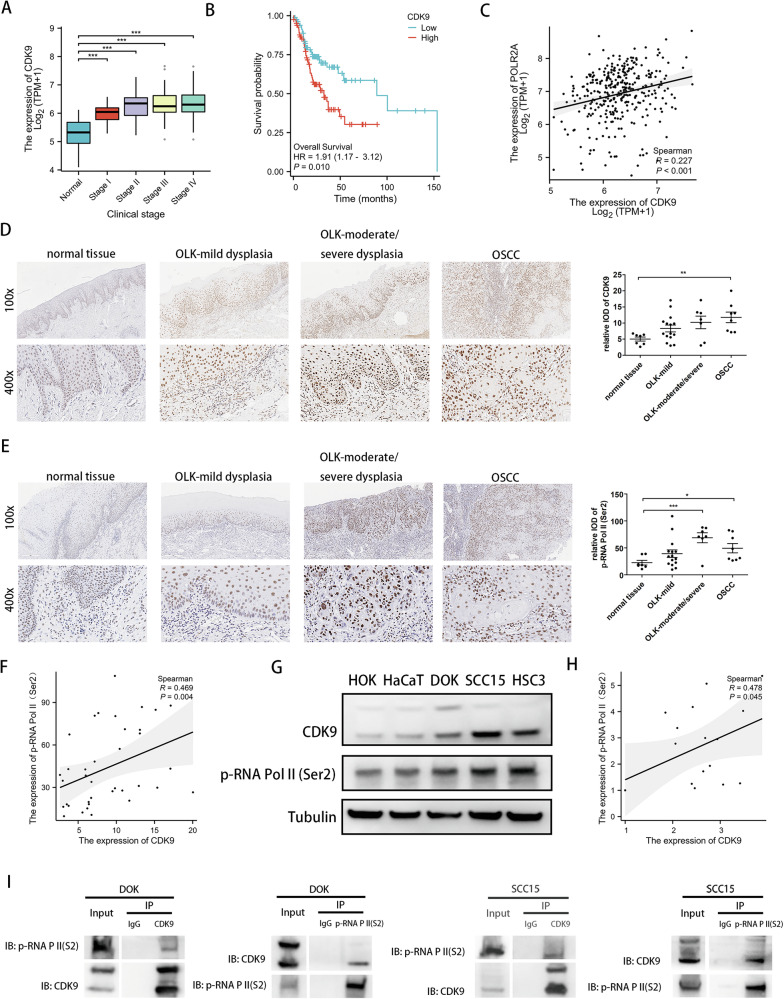
Increased CDK9 expression associates with high RNA Pol II activity and poor prognosis of OSCC. **A** Expression level of CDK9 in different clinical stages according to TCGA-OSCC set. **B** Kaplan-Meier overall survival curves of patients from TCGA-OSCC set which were sub-grouped as either CDK9 low-expression group or high-expression group. **C** Analysis of correlation between CDK9 and RNA Pol II (POLR2A) according to TCGA-OSCC set. **D**, **E** IHC assay to measure the levels of CDK9 (**D**) and p-RNA Pol II (Ser2) (**E**) in the four groups. Left: Representative images for each group, magnification, 100* and 400*; Right: Quantification of CDK9 and p-RNA Pol II (Ser2) in the four groups. **F** Analysis of correlation between CDK9 and RNA Pol II (POLR2A) according to IHC staining. **G** Western blot of CDK9 and p-RNA Pol II (Ser2) in HOK, HaCaT, DOK, SCC15, HSC3. **H** Analysis of correlation between CDK9 and p-RNA Pol II (Ser2) according to western blot. **I** Co-IP to detect CDK9-p-RNA Pol II (Ser2) using anti-p-RNA Pol II (Ser2) beads and anti-CDK9 beads. (*P < 0.05, **P < 0.01, ***P < 0.001).

Firstly, we analyzed the expression of tCDKs (CDK7, CDK8, CDK9, CDK12, CDK13 and CDK19) in different clinical stage according to the TCGA-OSCC dataset. As shown in Fig. S1A, CDK8, CDK9, CDK12 and CDK13 were differentially expressed since the clinical stage I or II of OSCC. The Kaplan-Meier analysis of overall survival revealed that CDK7, CDK8, CDK9, CDK12 and CDK13 could significantly affect the prognosis of OSCC patients (Fig. S1B), among which the hazard ratio of CDK9 was the highest (HR = 1.91; 95% confidence interval [CI], 1.17–3.12; *P* < 0.01) (Fig. 2A, B). Additionally, there was a weak correlation between the expression levels of CDK9 and RNA Pol II (*P* < 0.001) (Fig. 2C). The correlation analysis of CDK9 and RNA Pol II provided further support for our guess.

In order to confirm the above-mentioned findings, we next compared the expression of CDK9 and p-RNA Pol II (Ser2) in patients at different stages of oral mucosal carcinogenesis. There was a significant difference in CDK9 expression between normal tissues and OSCC tissues (*P* < 0.01) and a continuous upward trend during the process of carcinogenesis (Fig. 2D). The phosphorylation of p-RNA Pol II (Ser2) increased remarkably in moderate/severe dysplasia tissues and OSCC tissues (Fig. 2E), and the expression levels of CDK9 and p-RNA Pol II (Ser2) were positively correlated (Fig. 2F).

Finally, we detected the expression of tCDKs in HOK, HaCaT, DOK, SCC15, HSC3 cells and found that only CDK9 showed a successively increasing trend (Fig. S1C), suggesting that CDK9, not other tCDKs, may be an important driver of transcription dysregulation in oral mucosal carcinogenesis. As shown in Fig. 2G, H, the expression level of CDK9 in DOK, SCC15 and HSC3 cells was significantly higher than in HOK or HaCaT, and the correlationship between CDK9 and p-RNA Pol II (Ser2) was also verified in cell lines. These results of cell lines were consistent with tissue samples. Furthermore, to confirm whether CDK9 could interact directly with p-RNA Pol II (Ser2), we applied a Co-IP analysis and observed a robust interaction of them in DOK and SCC15 cells (Fig. 2I). Thus, the results of both clinical samples and relevant cells revealed a special status of CDK9 in transcriptional addiction during carcinogenesis of oral mucosa.

### Inhibition of CDK9 pauses RNA Pol II transcription cycle and induces cell apoptosis in DOK and OSCC cells

**Fig. 3:**
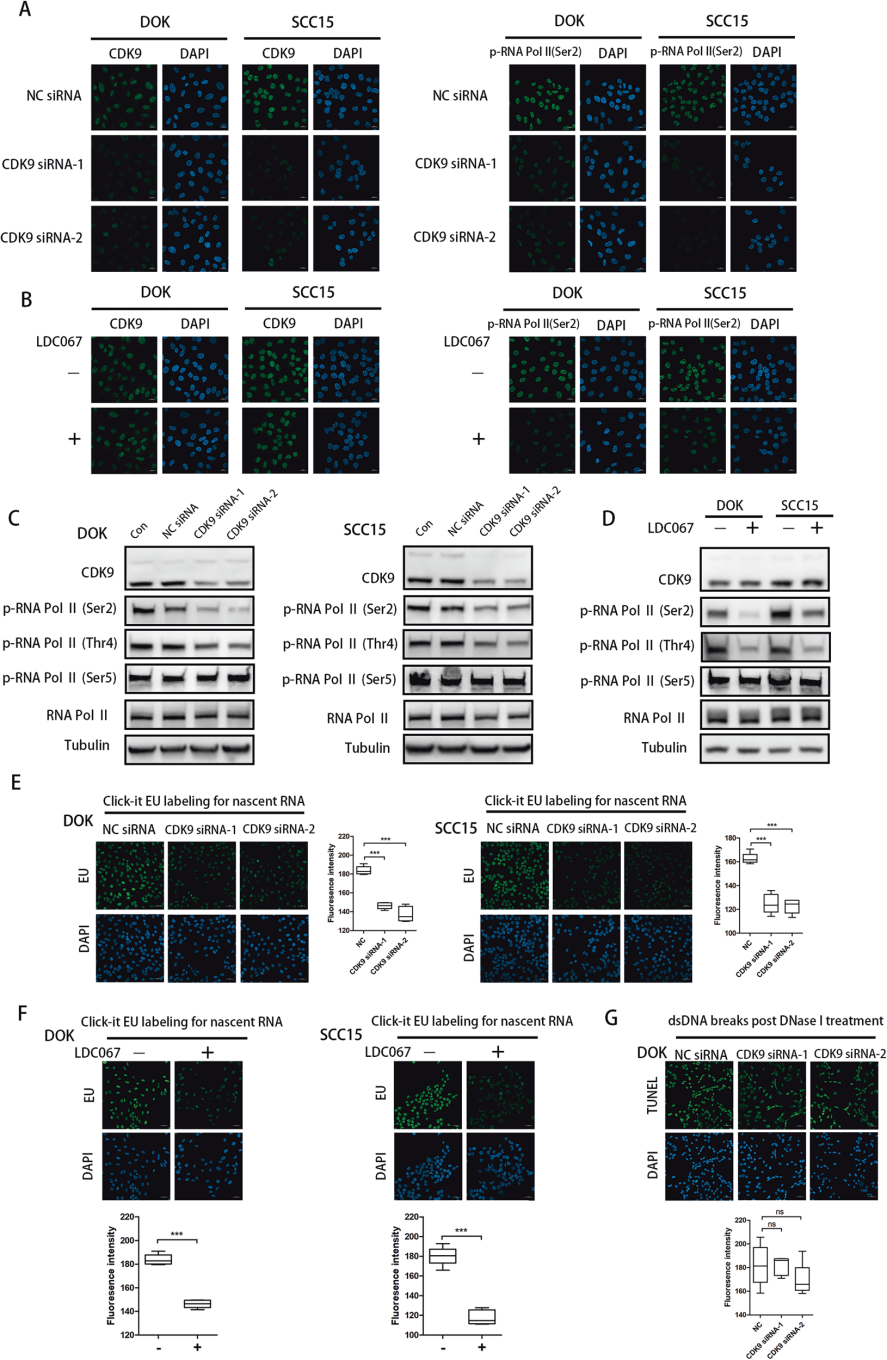
CDK9 inhibition reverses RNA Pol II-dependent transcriptional upregulation in DOK, SCC15 and HSC3 cells. **A** Immunofluorescent staining of CDK9 and p-RNA Pol II (Ser2) in DOK and SCC15 cells transfected with CDK9 siRNA. **B** Immunofluorescent staining of CDK9 and p-RNA Pol II (Ser2) in DOK and SCC15 cells treated with 10 μM LDC067 for 48 h. **C** Western blot of CDK9, p-RNA Pol II (Ser2), p-RNA Pol II (Thr4) and p-RNA Pol II (Ser5) in DOK and SCC15 cells transfected with CDK9 siRNA. **D** Western blot of CDK9, p-RNA Pol II (Ser2), p-RNA Pol II (Thr4) and p-RNA Pol II (Ser5) in DOK and SCC15 cells treated with 10 μM LDC067 for 48 h. **E** Analysis of nascent RNA synthesis in DOK and SCC15 cells transfected with CDK9 siRNA. **F** Analysis of nascent RNA synthesis in DOK and SCC15 cells treated with 10 μM LDC067 for 48 h. **G** Analysis of chromatin accessibility in DOK cells transfected with CDK9 siRNA. (*P < 0.05, **P < 0.01, ***P < 0.001).

Given that CDK9 binds RNA Pol II to drive transcriptional elongation and regulate transcription activities, CDK9 siRNA and CDK9 inhibitor were employed respectively to validate the function of CDK9 in transcriptional activity in DOK, SCC15 and HSC3 cells. As shown in Fig. 3A, in DOK and SCC15 cells, CDK9 siRNA transfection significantly inhibited CDK9 expression. Meanwhile, as expect, silencing of CDK9 with siRNA inhibited the phosphorylation of p-RNA Pol II (Ser2). LDC067 is a highly selective inhibitor of CDK9, which could inhibit the activity of CDK9 without decreasing its expression level. In DOK and SCC15 cells, after treated with LDC067, the phosphorylation of p-RNA Pol II (Ser2) remarkably decreased while there was no obvious difference in CDK9 expression (Fig. 3B). Furthermore, western blot revealed that CDK9 inhibition caused lower phosphorylation of p-RNA Pol II at Ser2, Thr4, while the phosphorylation of p-RNA Pol II at Ser5 did not markedly change (Fig. 3C, D). And similarly, the expression changes were also observed in HSC3 cells (Fig. S2). We next assessed the effect of CDK9 inhibition on the newly transcribed RNA levels and chromatin accessibility. Nascent transcripts synthesis in DOK and SCC15 cells, when transfected with CDK9 siRNA or treated with LDC067, was significantly reduced compared with untreated cells (Fig. 3E, F). However, there were no obvious changes in chromatin accessibility (Fig. 3G and S3). We searched for the reason in the discussion. These results support the notion that CDK9 plays an important role to trigger transcriptional addiction and CDK9 inhibition could pause RNA Pol II transcription cycle in oral cells.

**Fig. 4:**
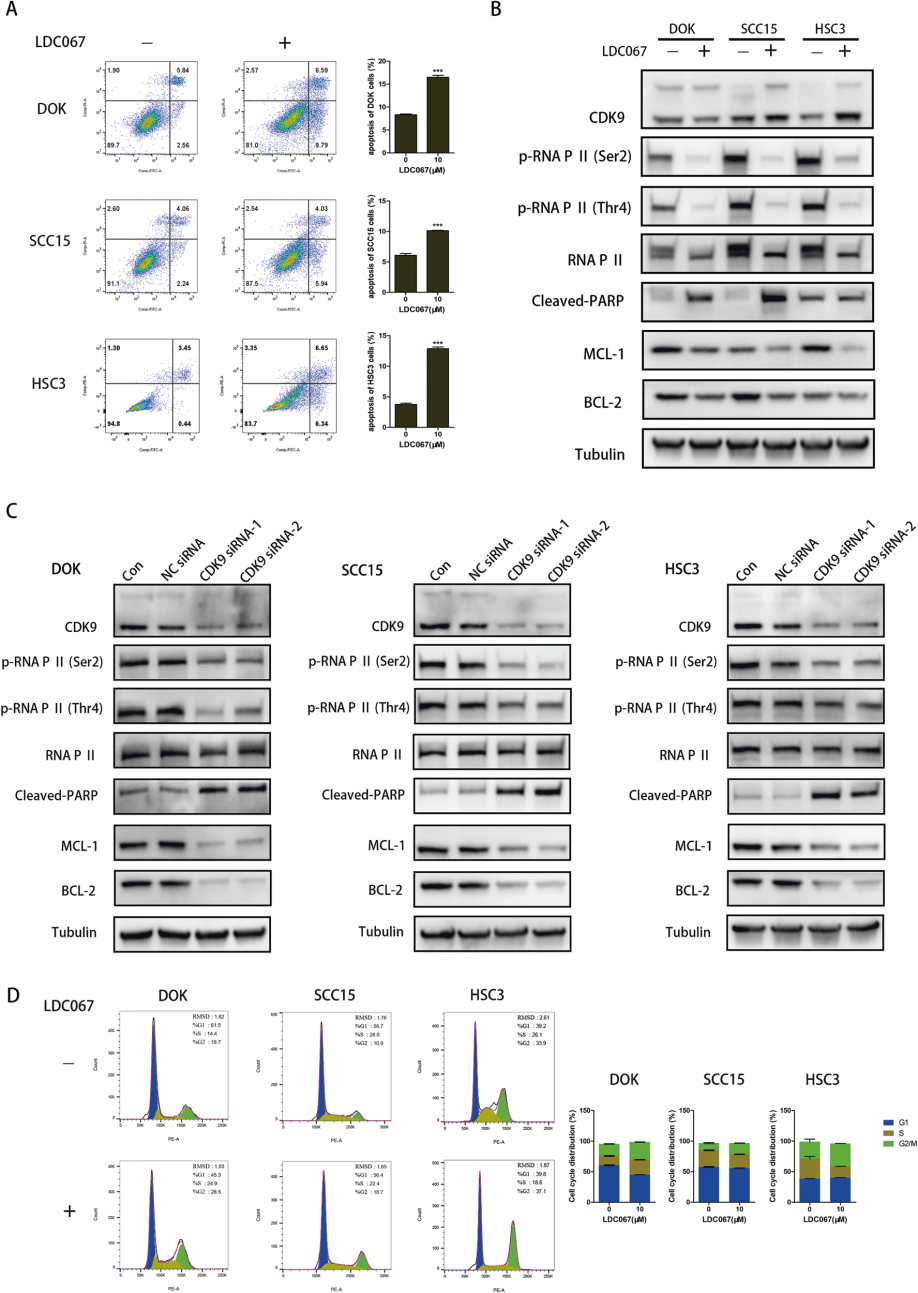
Inhibition of CDK9 induces cell apoptosis and G2/M cell cycle arrest in DOK, SCC15 and HSC3 cells. **A** Flow cytometry to detect apoptosis changes in DOK, SCC15 and HSC3 cells treated with 10 μM LDC067 for 48 h. **B** Western blot of apoptosis-related proteins in DOK, SCC15 and HSC3 cells treated with 10 μM LDC067 for 48 h. **C** Western blot of apoptosis-related proteins in DOK, SCC15 and HSC3 cells transfected with CDK9 siRNA. **D** Cell cycle assay in DOK, SCC15 and HSC3 cells treated with 10 μM LDC067 for 48 h. (*P < 0.05, **P < 0.01, ***P < 0.001).

After that, we further tested the role of CDK9 inhibition in cell proliferation and apoptosis. After treated with LDC067 for 48 h, there was a dose-dependent decrease in the cell viability of DOK, SCC15 and HSC3 cell lines, and the IC50 value of DOK (8.78 μM) was lower than SCC15 (13.49 μM) and HSC3 (10.77 μM) cells (Fig. S4A). The results of clone formation showed that the clonogenicity of DOK, SCC15 and HSC3 cells was significantly reduced when transfected with CDK9 siRNA (Fig. S4B). Besides, LDC067 efficiently induced apoptosis, measured by the accumulation of FITC-positive cells (Fig. 4A). A panel of apoptotic signaling molecules was also examined in treated cells and control cells by western blot. Increased cleaved PARP expression and decreased levels of MCL-1 and BCL-2 expression were detected in cells treated with LDC067 compared to those in control cells (Fig. 4B and Fig. S5). Similar results were observed in the cells transfected with CDK9 siRNA (Fig. 4C), suggesting that CDK9 inhibition induced apoptotic cell death. The results of cell cycle assay provided evidence that CDK9 inhibition caused G2/M cell cycle arrest in DOK, SCC15 and HSC3 cells (Fig. 4D and S4C). More importantly, compared with OSCC cells, DOK cells had less proliferative capacity and a larger percentage of apoptosis, suggesting that DOK cells may be more sensitive to CDK9 inhibition. Together, CDK9 inhibition block transcription to induce apoptosis in DOK and OSCC cells.

### CDK9 inhibition could effectively block the carcinogenesis of oral mucosa in vivo

**Fig. 5:**
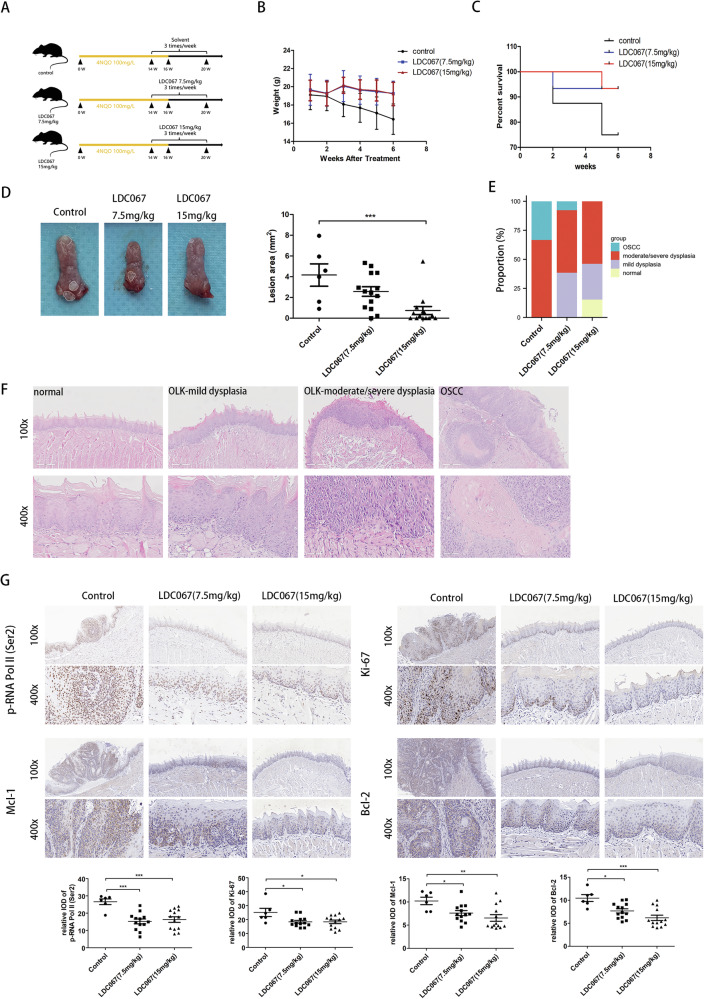
Inhibition of CDK9 effectively blocks the carcinogenesis of oral mucosa in 4NQO-induced mouse model. **A** Schematic diagram of the experimental strategies in vivo (Control, *n* = 6; LDC067 7.5 mg/kg, *n* = 13; LDC067 15 mg/kg, *n* = 13). **B** The body weight changes after LDC067 treatment in different groups. **C** Survival analysis in different groups. **D** Representative gross observation of the tongues in different groups at endpoint and quantification of the lesion size in different groups. **E** Quantification of pathological grade in different groups. **F** Representative H&E staining of different pathological grades, including normal tissue, mild dysplasia, moderate/severe dysplasia, and OSCC. **G** IHC assay to measure the expression levels of p-RNA Pol II (Ser2), Ki-67, Mcl-1 and Bcl-2 in the three groups. Left: Representative images for each group, magnification, 100*; Right: Quantification of p-RNA Pol II (Ser2) in the four groups. (*P < 0.05, **P < 0.01, ***P < 0.001).

To assess the effects of CDK9 inhibition on the oral mucosal carcinogenesis, we established the tongue carcinogenesis mouse model by using 4-NQO, and treated model mice with LDC067 as described in Material and method section (Fig. 5A). The results showed that the weight of mice in the LDC067 treatment group was stable, while the weight of mice in the untreated group continued to decrease (Fig. 5B), and LDC067 improved mouse survival (Fig. 5C). When the experiment was terminated at week 21, we found that high-dose LDC067 significantly reduced the lesion area on the tongue surface (Fig. 5D). Each tongue was pathologically diagnosed and categorized into normal, mild-dysplasia, moderate/severe dysplasia and OSCC areas. The proportion of moderate/severe dysplasia or OSCC in the treated groups was significantly lower than that in the untreated group (Fig. 5E, F). According to IHC experiments, LDC067 significantly reduced the phosphorylation of p-RNA Pol II (Ser2) in vivo (Fig. 5G), which indicated that the transcription cycle of potential carcinogenic genes might be suppressed. Additionally, the expression of Ki-67, Mcl-1 and Bcl-2 also decreased in the treated groups (Fig. 5G). These data indicated that LDC067 could effectively hamper the carcinogenesis of oral mucosa in the 4-NQO-induced tongue carcinogenesis mouse model.

### Gene features analysis of CDK9 inhibition in the carcinogenesis of oral mucosa

**Fig. 6:**
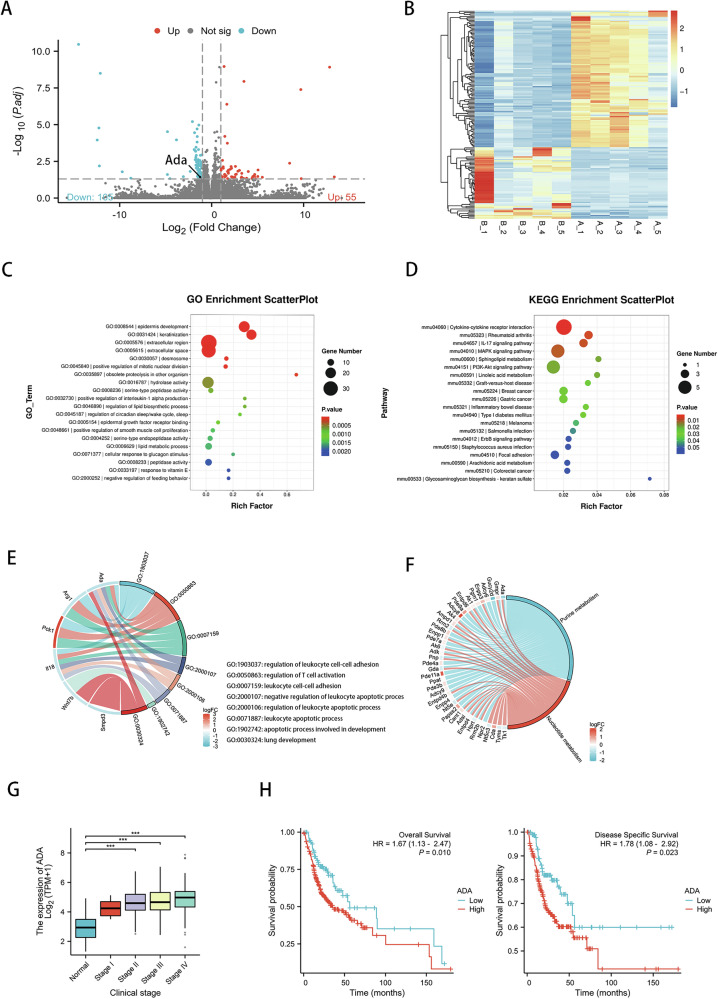
Gene features analysis of CDK9 inhibition in the carcinogenesis of oral mucosa. **A** Volcano plot depicting differentially expressed genes between control group and high-dose group. **B** RNA-seq heatmap showing differentially expressed genes between control group and high-dose group. **C** GO enrichment scatterplot. **D** KEGG enrichment scatterplot. **E** Biological processes (BP) analyzed by GO functional annotation of gene ADA. **F** KEGG enrichment analysis of gene ADA. **G** Expression level of ADA in different clinical stages according to TCGA-OSCC set. **H** Kaplan-Meier overall survival and disease specific survival curves of patients from TCGA-OSCC set which were sub-grouped as either ADA low-expression group or high-expression group. (*P < 0.05, **P < 0.01, ***P < 0.001).

To further explore the mechanism of LDC067 inhibiting the carcinogenesis of oral mucosa, mRNA sequencing was conducted on the tongue epithelium of the high-dose group and the control group. We identified 160 differentially expressed genes, among which 55 genes were upregulated and 105 genes were downregulated (Fig. 6A, B). Afterwards, functional enrichment analysis of differentially expressed genes was performed. Through GO enrichment, we found that these differentially expressed genes were mainly enriched in epidermis development, keratinization, and other biological process (Fig. 6C). As for KEGG pathways, the differentially expressed genes were significantly enriched in cytokine-cytokine receptor interaction, rheumatoid arthritis, IL-17 signaling pathway and MAPK signaling pathway (*P* < 0.01) (Fig. 6D). We then determined ADA as potential candidate which was significantly downregulated in the high-dose LDC067 treated group. On the one hand, GO analysis revealed multiple biological processes related to ADA including regulation of leukocyte cell-cell adhesion, regulation of T cell activation, leukocyte cell-cell adhesion, negative regulation of leukocyte apoptotic process, regulation of leukocyte apoptotic process, leukocyte apoptotic process, lung development (Fig. 6E). On the other hand, KEGG analysis revealed that ADA was significantly correlated with 2 metabolic pathways, purine metabolism and nucleotide metabolism (Fig. 6F).

ADA catalyzes the irreversible hydrolytic deamination of adenosine and deoxyadenosine, the final products of which are inosine and deoxyinosine [24]. Considering that ADA might play an important role in LDC067 blocking the oral mucosal carcinogenesis, we further analysis the expression of ADA in TCGA-OSCC set. Compared with normal control, ADA expression significantly increased in OSCC patients since clinical stage II (Fig. 6G). Meanwhile, patients with high ADA expression had significantly worse overall survival rates as well as disease-free survival rates than the patients with low ADA expression (Fig. 6H). Together, CDK9 inhibition induced broad changes to gene expression to hamper the carcinogenesis of oral mucosa in vivo, and targeting ADA-mediated adenosine-inosine metabolism might have potential to inhibit oral mucosal carcinogenesis.

### Inhibition of CDK9 prevented oral mucosal carcinogenesis by targeting ADA to disrupt adenosine-inosine metabolism

**Fig. 7:**
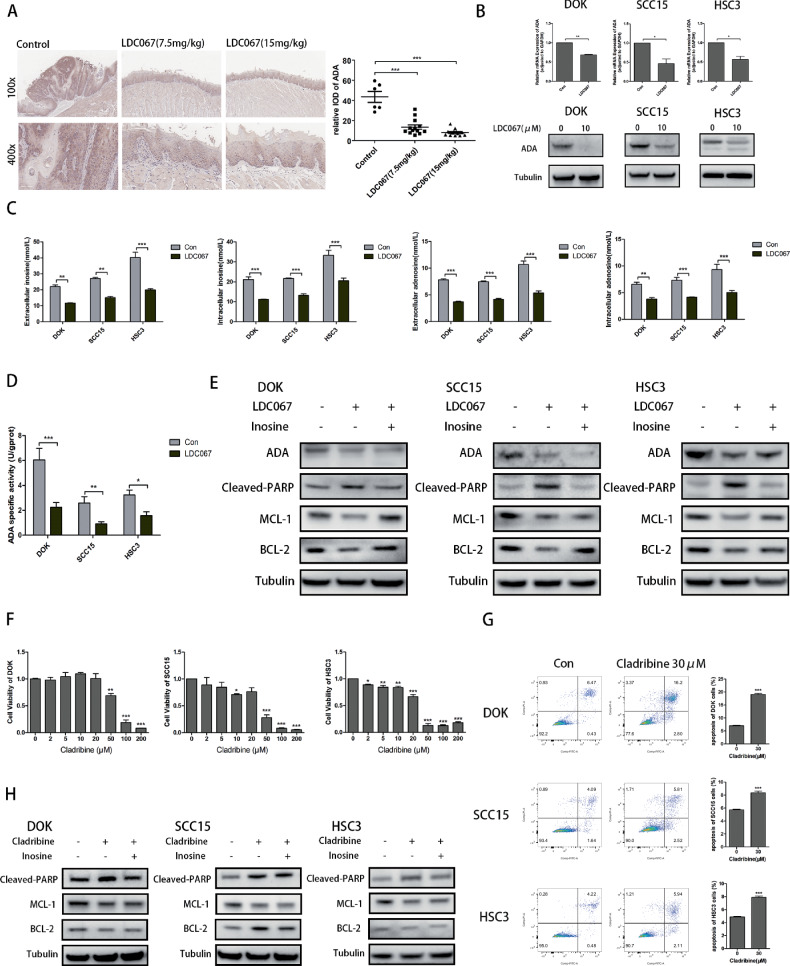
Inhibition of CDK9 prevents oral mucosal carcinogenesis by targeting ADA to disrupt adenosine-inosine metabolism. **A** IHC assay to measure the levels of ADA in the three groups. Left: Representative images for each group, magnification, 100* and 400*; Right: Quantification of ADA in the four groups. **B** qRT-PCR and western blot of ADA in DOK, SCC15 and HSC3 cells treated with 10 μM LDC067 for 48 h. **C** Concentrations of extracellular and intracellular inosine, adenosine in DOK, SCC15 and HSC3 cells treated with 10 μM LDC067 for 48 h. **D** ADA activity in DOK, SCC15 and HSC3 cells treated with 10 μM LDC067 for 48 h. **E** Western blot of apoptosis-related proteins in DOK, SCC15 and HSC3 cells. **F** CCK-8 assay to detect proliferation ability of DOK, SCC15 and HSC3 cells treated with cladribine at the indicated concentrations for 24 h. **G** Flow cytometry to detect apoptosis changes in DOK, SCC15 and HSC3 cells treated with 30 μM cladribine for 24 h. **H** Western blot of apoptosis-related proteins in DOK, SCC15 and HSC3 cells. (*P < 0.05, **P < 0.01, ***P < 0.001).

As previously shown, mRNA sequencing revealed that LDC067 significantly downregulated the expression level of ADA. As for protein level, we performed IHC assay with samples from the mouse model above. Consistent with the mRNA sequence analysis, LDC067 downregulated the expression of ADA in protein level (Fig. 7A). Subsequently, we examined the ADA expression in DOK, SCC15 and HSC3 cells to verify the effect. The results of qRT-PCR and western blot showed that LDC067 also downregulated the expression of ADA in DOK, SCC15 and HSC3 cells (Fig. 7B).

To further test the condition of adenosine-inosine metabolism in DOK, SCC15 and HSC3 cells, we compared the extracellular and intracellular adenosine and inosine, the substrate and product of ADA, respectively. LDC067 significantly decreased the concentration of both extracellular and intracellular inosine. However, the concentration of adenosine was also decreased (Fig. 7C), for there exists other ways to consume adenosine, such as purinergic receptors [25]. Besides, we detected the activity of ADA, and results showed that LDC067 reduced the deaminase activity in DOK, SCC15 and HSC3 cells (Fig. 7D). Thus, we made a conjecture that LDC067 disrupt adenosine-inosine metabolism mediated by ADA to induce cell apoptosis in DOK, SCC15 and HSC3 cells. Afterwards, the rescue experiment was performed. As shown in Fig. 7E, LDC067 could increase the expression of cleaved PARP while inosine rescued this LDC067-induced apoptosis. Especially in DOK cells, both MCL-1and BCL-2 expression were increased by inosine. Meanwhile, inosine did not change the ADA expression level as expected (Fig. 7E).

In addition, we used cladribine, a selective inhibitor of ADA, to further verify the roles of ADA in the proliferation and apoptosis of DOK, SCC15 and HSC3 cells. Cell viability was decreased in a dose-dependent manner in DOK, SCC15 and HSC3 cell lines (Fig. 7F and S6). Flow cytometry analysis results showed that in DOK cells, the proportion of cells in FITC-positive region in cladribine-treated group was 19.06%, which was higher than that in control group (6.97%). Similar results were also observed in SCC15 and HSC3 cells (Fig. 7G). Furthermore, the expression levels of apoptosis-related proteins were assessed. As shown in Fig. 7H and S7, the PARP apoptotic pathway was activated by cladribine, and MCL-1 was also decreased. However, there were no obvious changes in cladribine+inosine group compared with cladribine group, for the reason that decreasing inosine was probably not the main mechanism of cladribine-induced apoptosis [26].

## Discussion

The successful prevention or treatment of precancers has the potential to eliminate deaths owing to cancer [14]. Insights stemming from the identification of ‘transcriptional addiction’ as a consequence of genetic alterations have introduced the potential for therapies targeting the transcriptional addiction in cancers [2, 27]. Cancers or precancers with high transcriptional activity could show increased newly transcribed RNA levels and chromatin accessibility. In our study, we found that high transcriptional activity exists in the process of oral mucosal carcinogenesis, identifying a consequent dependency that can be targeted for therapeutic effect. To our best knowledge, these results firstly identified that transcriptional addiction plays a role in precancerous stages, which strongly suggested that therapeutically beneficial responses may arise from transcriptional perturbation in or before the initial stage of tumors.

Transcriptional regulation is a complex synthesis of numerous oppositional or enhancing regulatory inputs [27]. The critical role played by CDK9 in facilitating RNA Pol II transcriptional elongation makes CDK9 inhibitors well suited for the treatment of cancers characterized by transcriptional dysregulation as a consequence of MYC amplification or MLL1 rearrangements [2, 28, 29]. Our findings are in accord with recent studies that targeting of CDK9 has reduced the downstream phosphorylation of RNA Pol II at Ser2 and decreased nascent transcripts synthesis, showing promising in vitro and in vivo anti-tumor activity in carcinogenesis [8, 30]. In our research, we found that inhibition of CDK9 paused RNA Pol II transcription cycle and induced G2/M cell cycle arrest at the same time. CDK9 affects the cell cycle indirectly through transcriptional regulation, for CDK9 inhibitor could reduce the levels of cyclin B1 and MYC, which both have been considered the direct regulator of cell cycle. Besides, CDK9 inhibitor could induce RNA Pol II promoter pausing at G2/M checkpoint and MYC target genes [31, 32]. However, CDK9 inhibition showed no obvious changes in chromatin accessibility, for the reason that CDK9 inhibition induced epigenetic remodeling with bi-directional changes in chromatin accessibility. It has been reported that CDK9 inhibition brings relatively equal numbers of genomic regions with increased and decreased accessibility and the CTCF binding motif was highly enriched in genomic regions with decreased accessibility, which has been proposed to regulate CDK9 recruitment at the MYC locus [33, 34]. Besides, several factors regulate chromatin accessibility, including post-translational histone modifications, topological organization of nucleosomes, and positioning of chromatin binding factors [35], which might also contribute to the bi-directional changes in chromatin accessibility.

Due to the growing clinical need for more nuanced therapeutic manipulation of transcription cycle, multiple CDK9 small-molecule pharmacologic inhibitors have been investigated in preclinical and clinical studies [36]. To date, at least seven CDK9 inhibitors have demonstrated sufficient antineoplastic activity to be carried forward in phase I or II clinical trials [37–43]. Here, we found that CDK9 inhibition with the small molecule inhibitor LDC067 potently induced apoptosis in vitro and in vivo. LDC067 upmodulated cleaved-PARP and downmodulated MCL-1 and BCL-2 protein levels. Previous reports have demonstrated that osteosarcoma cells treated with LDC067 also suppressed expression of MCL-1 [8]. The efficacy and in vivo tolerability of LDC067 demonstrated in this study position this agent as ideally suited for rapid translation to trials about precancers.

It has been reported that treatment with LDC067 induced apoptosis in several cancer cell lines, which was assumed to be due to loss of short-lived apoptotic transcripts [44, 45] while we firstly found that LDC067 decreased ADA expression in vitro and in vivo. The purine metabolism is tightly regulated by multiple enzymes, and dysfunction in these enzymes leads to excessive cell proliferation and immune imbalance that result in tumor progression [24]. Low purine levels can slow cell proliferation, whereas high levels of purines are often seen in rapidly dividing cells, such as cancer cells, and high ADA activity occurs in various diseases and disorders including different cancers [46]. In addition, its association with disease staging and cancer staging both has been reported [18]. Studies have shown that ADA inhibition decreases tumor size and tumor growth in mouse models of breast cancer and human breast cancer cells as well [47]. Besides, ADA inhibition could also reduce inflammation in animal models of colitis [48]. Consistent with these studies, we find that models of oral mucosal carcinogenesis treated with LDC067 shows decreased ADA expression and in DOK, SCC15 and HSC3 cells, LDC067 suppresses both ADA activity and expression level, regulates the intracellular and extracellular concentrations of adenosine and inosine. It has been reported that adenosine is significantly elevated in the tumor microenvironment compared to normal tissues. Reducing adenosine production in the tumor microenvironment can significantly enhance anti-tumor immune responses and improve the efficacy of other immunotherapies [19]. In our study, the inhibitory effect of LDC067 on ADA did not result in adenosine accumulation, but rather decreased its concentration, suggesting there exists other mechanisms of LDC067 inhibiting carcinogenesis through targeting adenosine generation needed to explore. As for inosine, it has been found to have multifaceted effects across various tissues and diseases. Inosine could function as a crucial biomarker associated with cancer metastasis, drug resistance and/or treatment, and tumor progression [20]. In esophageal squamous cell carcinoma, inosine has been associated with increasing risk of cancer progression [49]. Supplementation of inosine was also able to rescue cell death in pancreatic cancer [50]. Consistent results were also shown in our research that cell apoptosis induced by LDC067 could be rescued by exogenous inosine supplementation in both precancerous cell line DOK, SCC15 and HSC3 cells. Therefore, targeting purine metabolism, especially the enzymatic conversion of adenosine to inosine has promising role in hampering oral mucosal carcinogenesis, and we firstly observe a connection between transcriptional addiction and purine metabolism.

In summary, we identified transcriptional addiction as an important feature in the process of oral mucosal carcinogenesis. Targeting this CDK9-dependent transcriptional addiction induces cell apoptosis by downregulating ADA, and thereby interfering with the enzymatic conversion of adenosine to inosine to hamper oral mucosal carcinogenesis. The anti-carcinogenesis effect demonstrated in 4-NQO-induced mouse models of oral mucosal carcinogenesis using CDK9 inhibitor could prove transformative if translated into clinical trials for preventing cancers in precancerous conditions.

## Materials and methods

### Patients and specimens

Paraffin sections were made using well-preserved biopsy from patients at the Hospital of Stomatology, Sun Yat-sen University [51, 52]. We analyzed a total of 37 samples, including 7 samples of normal tissues, 15 samples of mild-dysplasia, 7 samples of moderate/severe dysplasia, and 8 samples of OSCC, clinically diagnosed according to the WHO criteria for histological typing of cancer and precancer of the oral mucosa by the Department of Pathology, Guanghua School of Stomatology. Normal oral mucosal tissues were collected from volunteers who underwent wisdom teeth extraction. Written informed consents were acquired before biopsy and pathological examination.

### Animal experiments

Four-week-old female wild-type C57BL/6 mice were acquired from Laboratory Animal Center, Sun Yat-sen University East Campus and housed in the Experimental Animal Center at Sun Yat-sen University North Campus. Firstly, 12 C57BL/6 mice were divided into two groups: (1) the 4-NQO group was fed with 4-NQO (Sigma-Aldrich, St. Louis, MO, USA) (100 μg/mL) in drinking water for 16 weeks (*n* = 6); (2) the control group received regular drinking water (*n* = 6). Then the mice were observed for 1 week before sacrifice and tongue tissues were used for RNA-Seq. Secondly, 32 C57BL/6 mice were fed with 4-NQO until the 14th week, and then were divided into three groups randomly and received different treatment: (1) the control group received intraperitoneal injection of solvent (10% DMSO + 40% PEG300 + 5% Tween-80 + 45% Saline) three times a week (*n* = 6); (2) low-dose group received intraperitoneal injection of LDC067 (7.5 mg/kg body weight) (MedChemExpress, Monmouth Junction, NJ) three times a week (*n* = 13); (3) high-dose group received intraperitoneal injection of LDC067 (15 mg/kg body weight) three times a week (*n* = 13). After 2 weeks, 4-NQO was stopped, and LDC067 was continued to be injected intraperitoneally for 4 weeks. Then the mice were observed for 1 week before sacrifice. Inhalation anesthesia method was used. The tongue tissues from the control group and high-dose group were used for RNA-Seq. All animal experiments were approved by the Laboratory Animals Ethics Committee of Sun Yat-sen University (SYSU-IACUC-2023-001519).

### Cell lines and cell culture

The human head and neck squamous cell carcinoma (HNSCC) cell line HSC3, human dysplastic oral keratinocytes (DOK), and human epidermal keratinocytes (HaCaT) were obtained from Dr. Juan Xia and Dr. Xianyue Ren (Hospital of Stomatology, Guanghua School of Stomatology, Sun Yat-sen University). The human oral keratinocyte (HOK), human HNSCC cell line SCC15were procured from the American Type Culture Collection (ATCC; Rockville, MD, USA).

HaCaT, HSC3, and SCC15 were cultured in high-glucose DMEM (Gibco, NY, USA) supplemented with 10% fetal bovine serum (FBS, Gibco, NY, USA). HOK cells were cultured in keratinocyte serum-free medium (KSFM; Invitrogen) supplemented with 5 ng/ml epidermal growth factor (EGF) and 50 μg/mL of bovine pituitary extract (GIBCO). DOK cells were cultured in high-glucose DMEM (Gibco, NY, USA) supplemented with 10% fetal bovine serum (FBS, Gibco, NY, USA) and 5 μg/mL hydrocortisone (Sigma-Aldrich, St. Louis, MO, USA). All cells were incubated in a humidified atmosphere containing 5% CO_2_ at 37 °C.

### RNA-sequencing

The mouse tongues were treated with dispase II enzyme (Roche, Basel, Switzerland) to obtain tongue mucosa epithelium for RNA-Seq analysis. The RNA libraries were sequenced on the illumina Novaseq^TM^ 6000 platform by LC Bio Technology CO.,Ltd (Hangzhou, China). Differentially expressed genes (DEGs) analysis was performed using the DESeq R package. A *P*-value of <0.05 and a fold change of >2 or <0.5 were set as the threshold for significantly differential expression. Gene ontology enrichment (GO) and Kyoto Encyclopedia of Genes and Genomes (KEGG) pathway enrichment analysis of DEGs were performed using R based on the hypergeometric distribution.

### Bioinformatics analysis

Bioinformatics analysis was conducted on the basis of the TCGA cohort of head and neck squamous cell carcinoma (HNSCC), and samples belonging to oral cancer sites (Alveolar Ridge, Base of tongue, Buccal mucosa, Floor of mouth, Hard Palate, Oral Cavity, Oral Tongue) were retained according to the clinical information. The GSE30784 dataset was obtained through the GEO Database (https://www.ncbi.nlm.nih.gov/geo/). Differential analysis was performed using the R package limma (version 4.2.1) to obtain differential genes between the comparison groups and the control group. The survival data were analyzed using the “survival” (v3.3.1) R package. The Spearman’s correlation analysis was used to evaluate the relationship between expression levels of CDK9 and RNA Pol II.

### Nascent RNA labeling assay

Cells were cultured on pre-coated glass over slides. 24 h later, a nascent RNA synthesis assay was conducted using Click-It RNA Imaging Kits (Invitrogen, C10329) following the manufacturer’s protocols. The samples were analyzed using a laser scanning confocal microscope at a specific magnification and quantified using Image J software.

### DNase I-TUNEL experiment

Cells were permeabilized by 0.5% Triton X-100 in PBS buffer (Invitrogen) for 15 min before digesting with 0.2 U/ml of DNase I (NEB). Cells were then fixed in 4% PFA. TUNEL Assays (DeadEnd™ Fluorometric TUNEL System, Promega) were performed subsequently according to manufacturer’s instructions. The nuclear area was defined according to DAPI DNA staining. The samples were analyzed using a laser scanning confocal microscope at a specific magnification and quantified using Image J software.

### siRNA transfection

To investigate the function of CDK9 in DOK, SCC15 and HSC3 cells, corresponding small interfering RNA (siRNA) oligonucleotide duplexes (GenePharma, Shanghai, CN) was used. Using Lipofectamine™ RNAiMAX Transfection Reagent (13778150, Invitrogen, Carlsbad, CA), the siRNA oligonucleotides were transiently transfected into the targeted cells according to the manufacturer’s instructions. The CDK9 siRNA sequences were as follows: 5’-GCUGCUAAUGUGCUUAUCATT-3’ and 5’-CCCUCAACCACGACUUCUUTT-3’.

### Western blot

Cells were harvested, and the total protein was extracted with and lysed in RIPA buffer (P0013B, Beyotime, Shanghai, CN) supplemented with a 1% protease inhibitor cocktail (CW2200s, CWbio, Beijing, CN) and 1% phosphatase inhibitor cocktail (CW2383S, CWbio, Beijing, CN) on ice for 30 min. Protein concentration was quantified using the BCA Protein Assay Kit (CW0014, CWbio, Beijing, CN) according to the manufacturer’s protocol. Protein was separated on a 4%–20% gel (FuturePAGE™, ACE, Changzhou, CN) and blotted using a wet transfer system (Biorad). See Key Resources Table for antibodies utilized.

### Histological hematoxylin-eosin (HE) staining

Tongue tissues of mice were dissected and fixed overnight with 4% paraformaldehyde (Biosharp, Chongqing, China). Samples were then embedded in paraffin. Paraffin sections with 4 mm thick were stained with hematoxylin and eosin (Servicebio Co, Ltd., Wuhan, China) for histology.

### Immunohistochemistry (IHC)

The sections were deparaffinized and rehydrated in a series of gradient alcohols. Then, slides were immersed in sodium citrate buffer and boiled in a microwave for antigen retrieval. After inhibition of endogenous peroxidases and blocking, samples were covered with primary antibodies and incubated at 4 °C overnight. The following antibodies were used: CDK9, p-RNA Pol II(Ser2), Ki-67, MCL-1, BCL-2, ADA. The next day, HRP-conjugated secondary antibodies were applied for 30 min and visualized with DAB. The stained cells were calculated using Image J Pro Plus software.

### Co-Immunoprecipitation (Co-IP)

Immunoprecipitation experiments were performed with whole cell extracts. Cells were lysed in Co-IP lysis buffer (PR20037, Proteintech, Wuhan, CN) and Immunoprecipitation Kit with Protein A + G Magnetic Beads (P2179S, Beyotime, Shanghai, CN), then analyzed by western blot. Immunoprecipitation was performed using anti-CDK9 antibody, anti-p-RNA Pol II (Ser2) antibody and normal rabbit IgG. Antibody/extract mixtures were incubated with complete Co-IP/wash buffer on a 4 °C shaker for 4 h. Magnetic protein G beads were added and incubated another hour, then washed using the complete Co-IP/wash buffer and a magnetic stand. Bead pellets were re-suspended in 1X reducing loading buffer for immunoblotting.

### Immunofluorescence (IF) staining

Cells were seeded into observation dishes. After reaching 60–80% confluence, cells were fixed in 4% paraformaldehyde and incubated for 30 min at room temperature. After fixation, the sections were washed with PBS and permeabilized with PBS containing 0.5% TritonX-100 for 15 min at room temperature. Then, cells were blocked with 3% BSA for 30 min at room temperature and subsequently probed with different primary antibodies overnight at 4 °C. Subsequently, the cells were washed in PBS three times and followed by incubation with secondary antibodies for 2 h incubation in the dark at room temperature. The cells were then counterstained with DAPI (C1005, Beyotime, Shanghai, CN) for 3 min at room temperature and washed with PBS three times. The samples were analyzed using a laser scanning confocal microscope at a specific magnification.

### Cell proliferation assay

Cells were seeded at a cell density of 3000 cells/well into 96-well plates and cultured in the corresponding conditions. After incubation, cell proliferation was analyzed using the Cell Counting Kit-8 (CCK8, Sigma-Aldrich, USA) following the manufacturer’s protocol. We added 10% CCK8 solution (C0037, Beyotime) to each well for 0.5 h. The absorbance was measured at 450 nm using a microplate reader.

### Flow cytometry

Cells were seeded in 6-well plates (300,000 cells/well) overnight and were treated with LDC067 or cladribine. Apoptosis was monitored using the Annexin V, FITC Apoptosis Detection Kit (AD10, DOjindo) according to the manufacturer’s protocol. For cell cycle assay, cell cycle staining kit (Multi Sciences, Hangzhou, China) was applied to examine the DNA content of cells in different stages of cell cycle in accordance with the manufacture’s instruction. Data were acquired on LSRFortessa (BD Bioscience, NJ, USA) and was analyzed using Flowjo software (BD Bioscience, NJ, USA).

### RNA extraction and quantitative real-time PCR (qRT-PCR)

Total RNA was isolated from cells with RNA-Quick Purification kit (ES Science, Shanghai, China). RNA concentration was measured by NanoDrop One (Thermo Fisher Scientific Inc., CA, USA). HiScript III RT SuperMix for qPCR kit (Vazyme Biotech Co., Nanjing, China) was used to reversed-transcribe 1 μg of RNA to acquire cDNA. Quantitative RT-PCR was conducted to quantify gene expression level of GAPDH and ADA by applying ChamQ Universal SYBR qPCR Master Mix (Vazyme Biotech Co., Nanjing, China) and quantified by 2^−^^ΔΔCT^ method. The primers used in this study, purchased from GeneRay biotechnology (Guangzhou, China) were listed in Key Resources Table.

### Measurement of adenosine and inosine concentration

The measurement of adenosine and inosine concentration was conducted using the Adenosine Assay Kit (CB12066-Hu, COIBO, Shanghai, China) and Inosine Assay Kit (CB14808-Hu, COIBO) respectively. Similar principal was adopted in both assay kits. A fluorometric indicator was used to measure the concentration of a product converted from either adenosine or inosine. The adenosine and inosine concentrations were calculated based on the measurement in the presence or absence of the enzymatic converter.

### ADA enzymatic activity

After treatment with LDC067, cells were incubated at 37 °C for 48 h. ADA enzymatic activity was determined using ELISA kit from Elabscience (Wuhan, China). The experimental procedure was performed according to the manufacturer’s recommendation.

### Statistical analysis

Statistical analysis was performed with Prism 5 (GraphPad Software, San Diego, CA, USA). *P*-values of less than 0.05 were considered significant. Unless otherwise stated, comparison and statistical significance between two groups in this paper are based on a two-sided t-test. The analysis of variance (ANOVA) test was used for comparing data from multiple groups.

## Supplementary information

Figure S1

Figure S2

Figure S3

Figure S4

Figure S5

Figure S6

Figure S7

Table S1

Supplementary material legends

Key Resources Table

Original western blots

reproducibility checklist

## Data Availability

All datasets generated and analyzed during this study are included in this published article and its Supplementary material files. Additional data are available from the corresponding author upon reasonable request.
