# Supplementary figures and images for: Targeting CDK9-dependent transcriptional addiction: a novel chemoprevention strategy for oral carcinogenesis via adenosine deaminase modulation
Source: Cell Death Dis. 2025 Dec 8;16(1):881. doi: 10.1038/s41419-025-08224-5 (PMC12686051; doi:10.1038/s41419-025-08224-5)

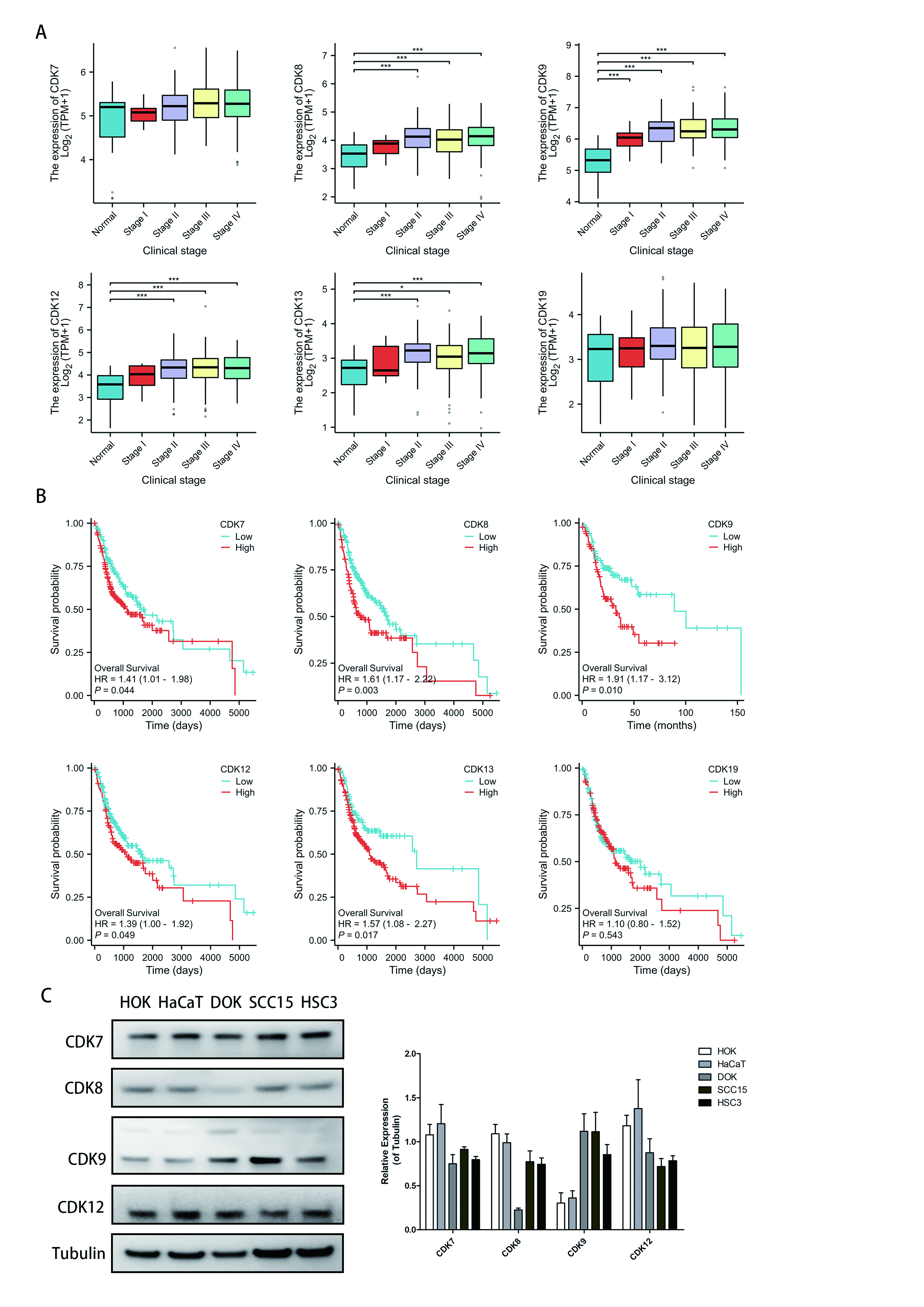

Supplement: Supplementary file 1 — Figure S1 [file 41419_2025_8224_MOESM1_ESM.tif]

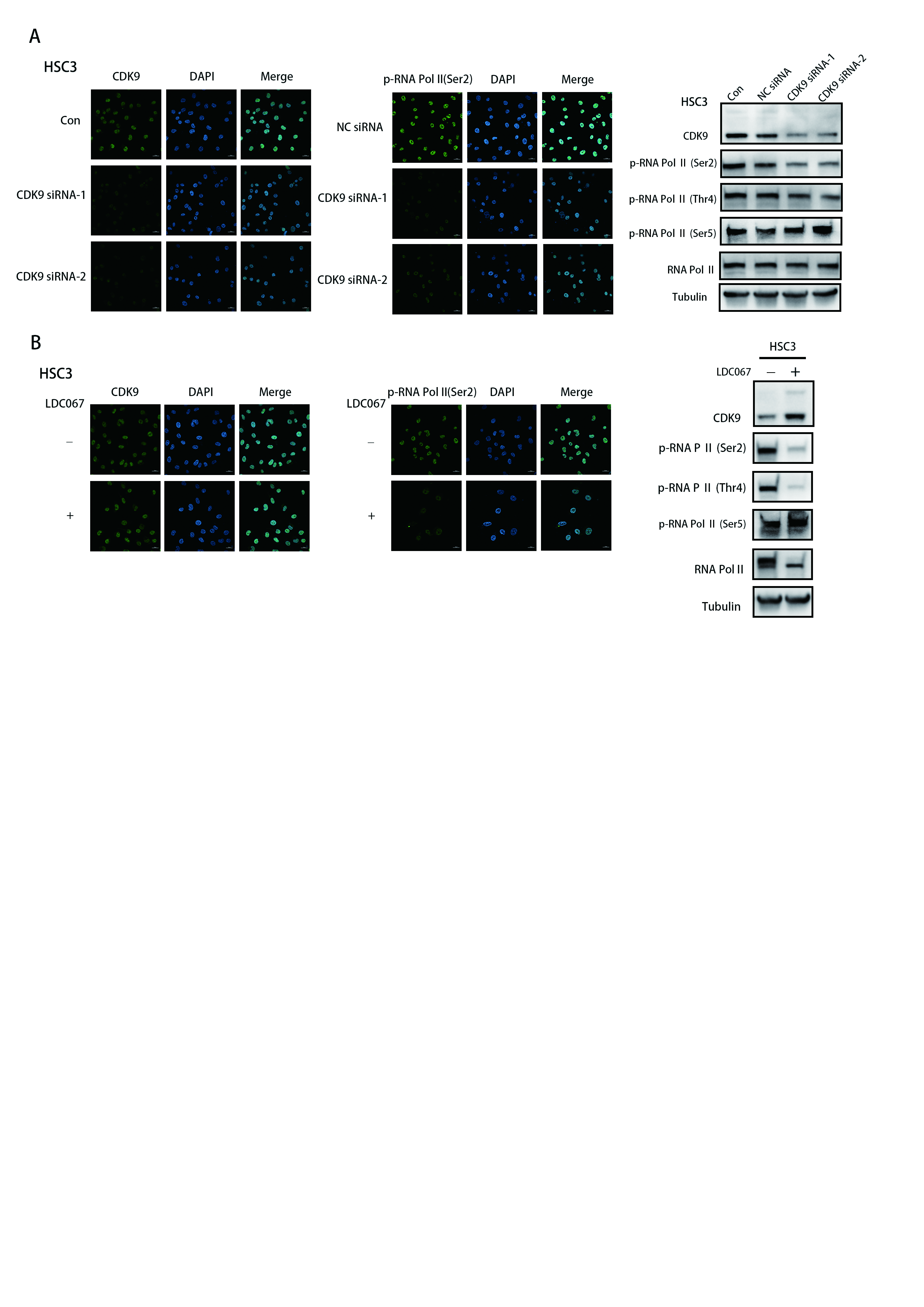

Supplement: Supplementary file 2 — Figure S2 [file 41419_2025_8224_MOESM2_ESM.tif]

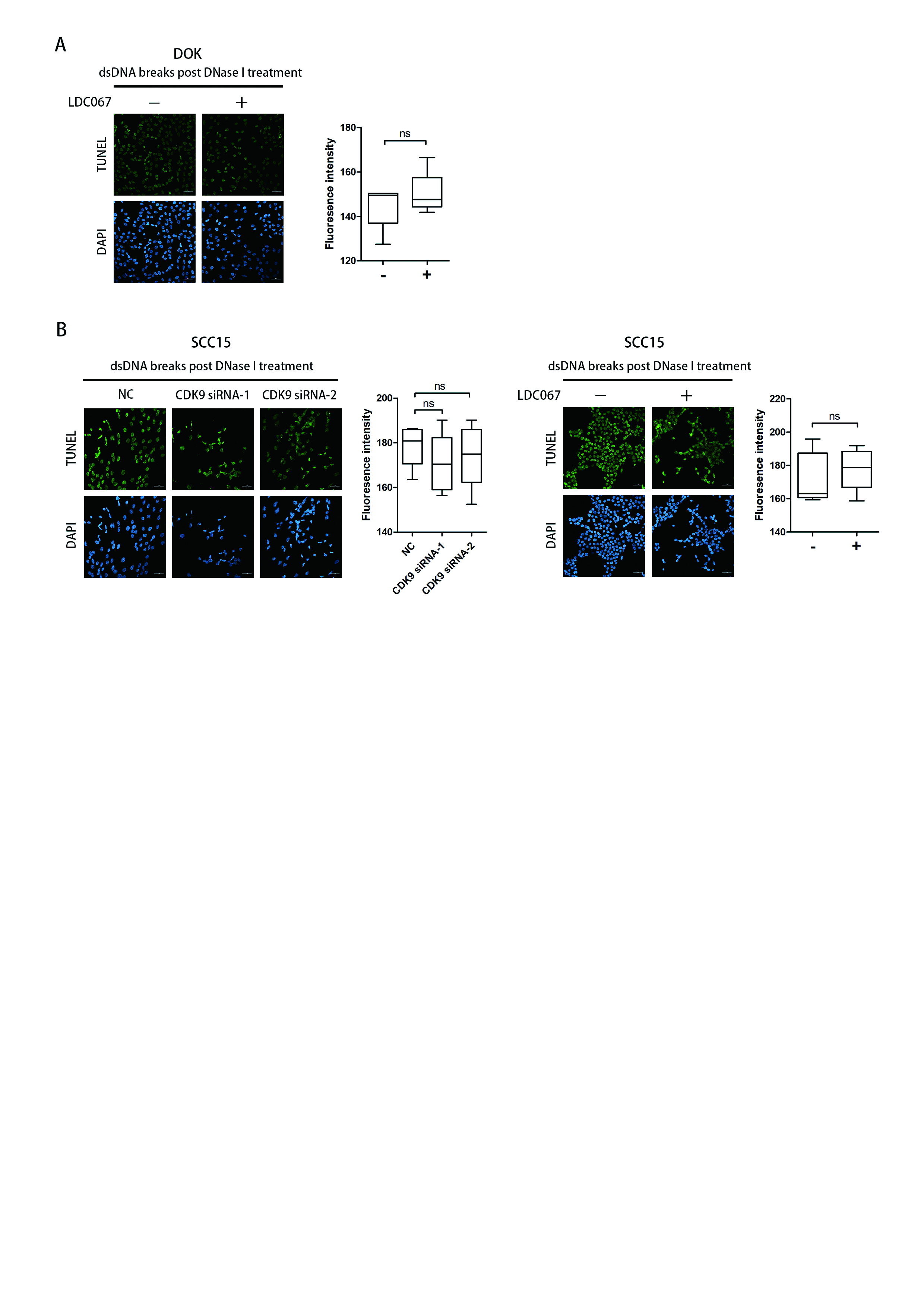

Supplement: Supplementary file 3 — Figure S3 [file 41419_2025_8224_MOESM3_ESM.tif]

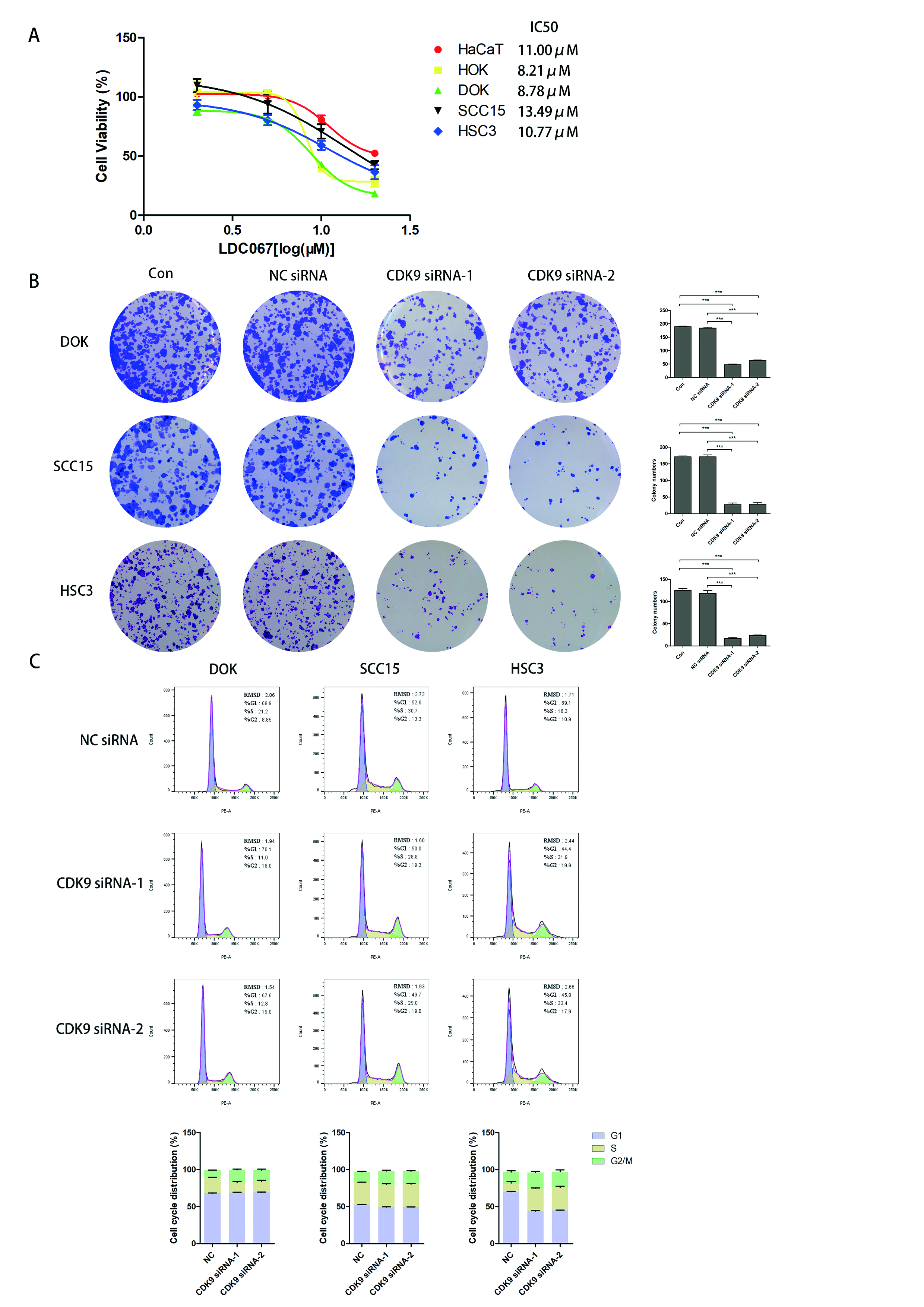

Supplement: Supplementary file 4 — Figure S4 [file 41419_2025_8224_MOESM4_ESM.tif]

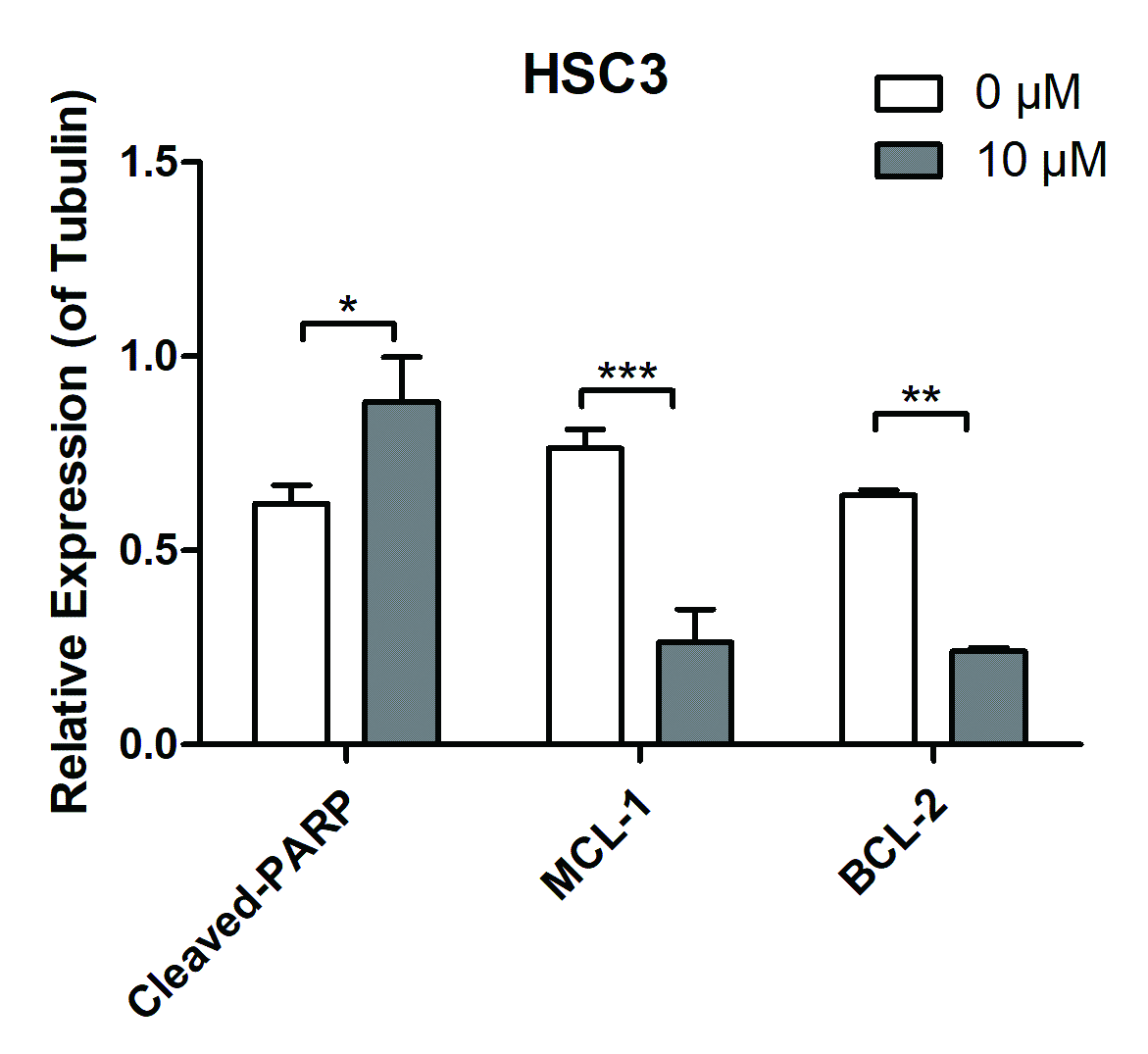

Supplement: Supplementary file 5 — Figure S5 [file 41419_2025_8224_MOESM5_ESM.tif]

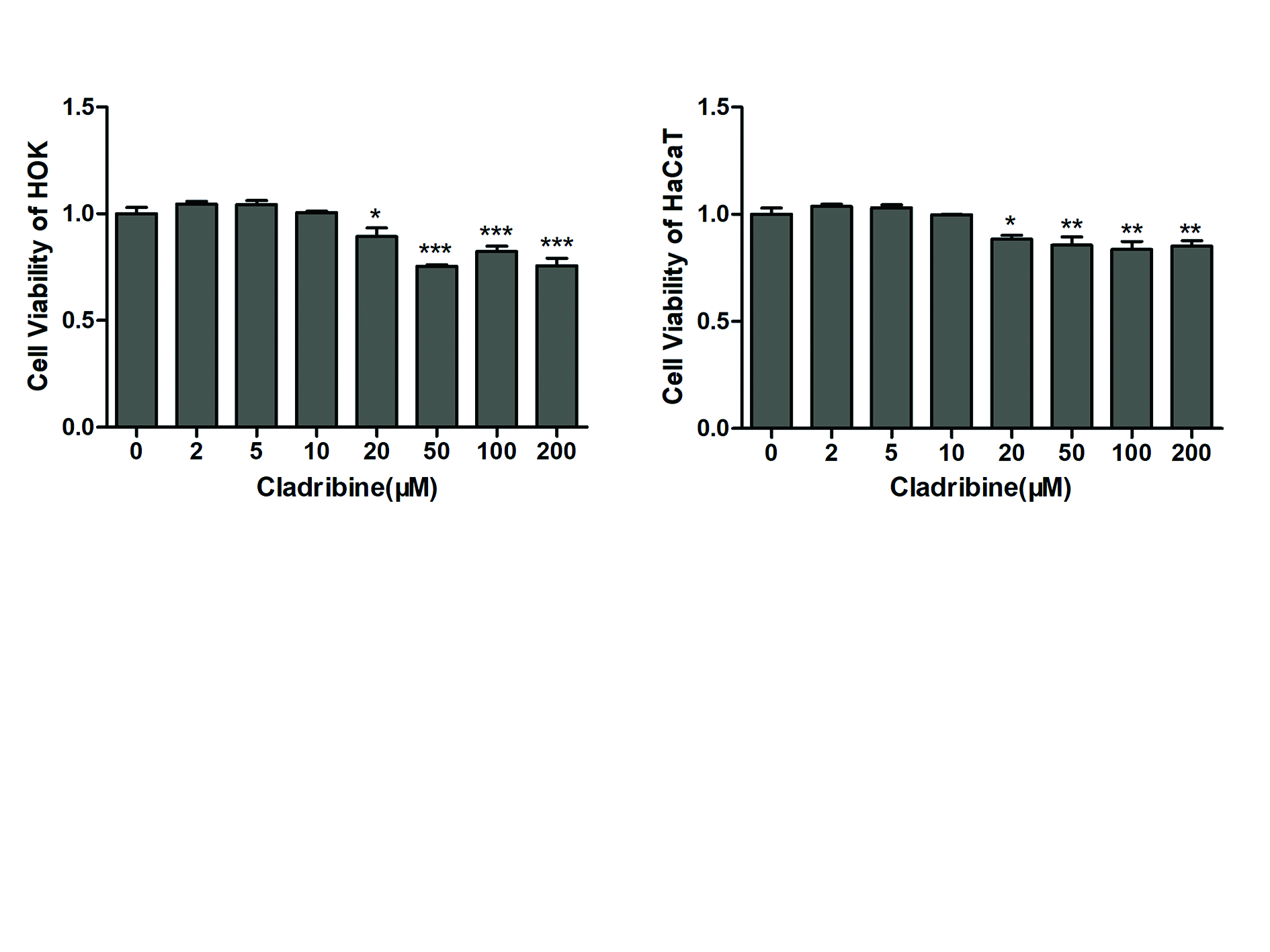

Supplement: Supplementary file 6 — Figure S6 [file 41419_2025_8224_MOESM6_ESM.tif]

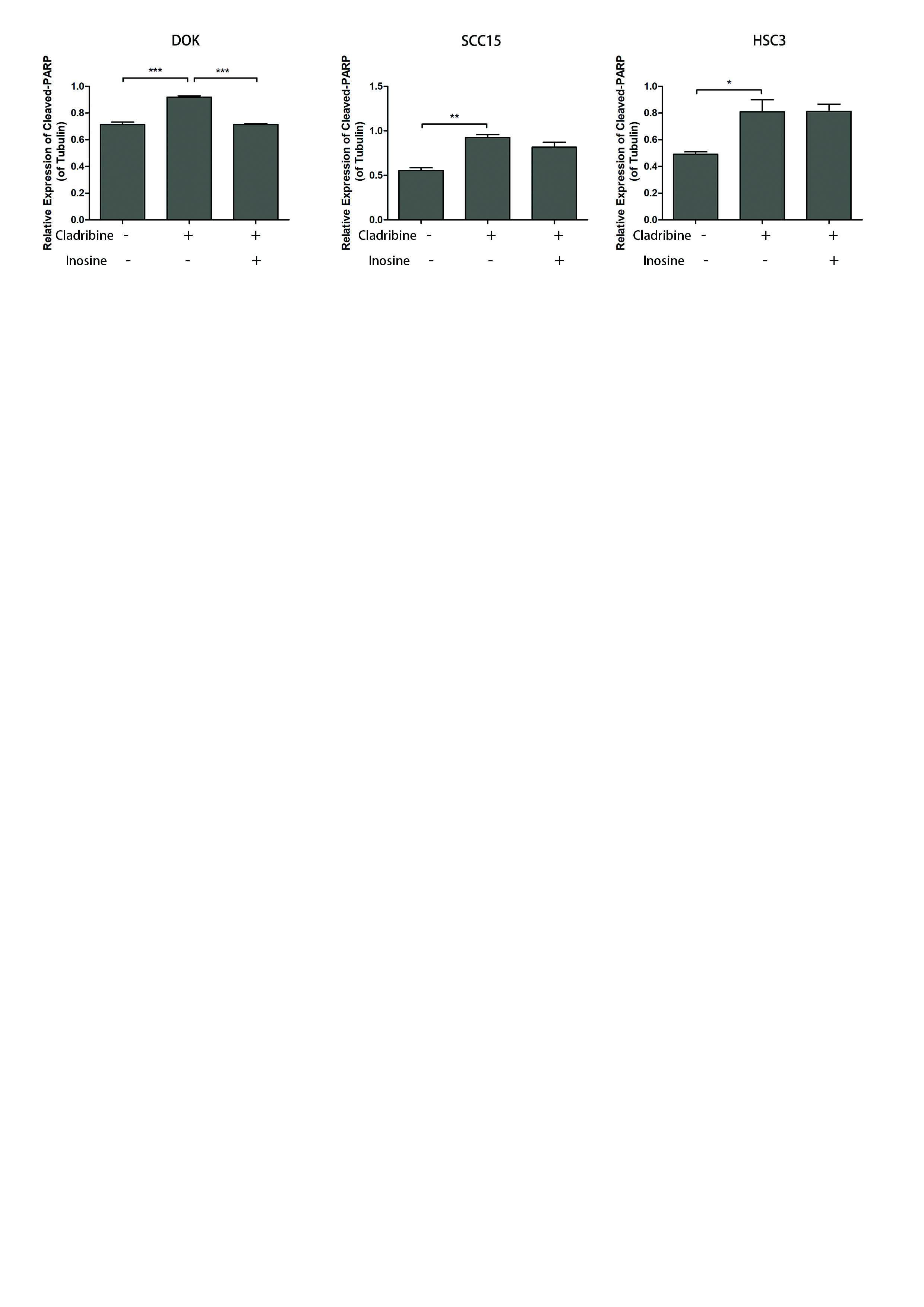

Supplement: Supplementary file 7 — Figure S7 [file 41419_2025_8224_MOESM7_ESM.tif]
