# Supplementary material for: Targeting CDK9-dependent transcriptional addiction: a novel chemoprevention strategy for oral carcinogenesis via adenosine deaminase modulation
Source: Cell Death Dis. 2025 Dec 8;16(1):881. doi: 10.1038/s41419-025-08224-5 (PMC12686051; doi:10.1038/s41419-025-08224-5)
Supplement: Supplementary file 9 — Supplementary material legends [file 41419_2025_8224_MOESM9_ESM.docx]

**Supplementary material legends**

Figure S1 Analysis of tCDKs in TCGA-OSCC set and related cell lines.

(A)Expression level of tCDKs in different clinical stages according to TCGA-OSCC set.

(B) Kaplan-Meier overall survival curves of patients from TCGA-OSCC set.

(C) Western blot of tCDKs in HaCaT, DOK, CAL27, UM1, SCC15, HSC3.

Figure S2 CDK9 inhibition suppresses phosphorylation of RNA Pol II in HSC3 cells.

(A) Immunofluorescent staining and Western blot in HSC3 cells transfected with CDK9 siRNA.

(B) Immunofluorescent staining and Western blot in HSC3 cells treated with 10μM LDC067 for 48h.

Figure S3 CDK9 inhibition decreases nascent transcripts synthesis in SCC15 cells.

(A) Analysis of chromatin accessibility in DOK cells treated with 10μM LDC067 for 48h.

(B) Analysis of chromatin accessibility in SCC15 cells transfected with CDK9 siRNA and treated with 10μM LDC067 for 48h.

Figure S4 CDK9 inhibition decreases proliferation and causes G2/M cell cycle arrest in DOK and OSCC cells.

(A) CCK-8 assay to detect proliferation ability of HOK, HaCaT, DOK, SCC15 and HSC3 cells treated with LDC067 at the indicated concentrations for 48h.

(B) Clone formation experiments to detect proliferation ability of DOK, SCC15 and HSC3 cells transfected with CDK9 siRNA.

(C) Cell cycle assay in DOK, SCC15 and HSC3 cells transfected with CDK9 siRNA.

Figure S5 The quantitative immunoblotting analysis of Figure 4B

Figure S6 CCK-8 assay to detect proliferation ability of HOK, HaCaT, DOK, SCC15 and HSC3 cells treated with cladribine at the indicated concentrations for 48h.

Figure S7 The quantitative immunoblotting analysis of Figure 7H

Table S1 Functions or features of transcriptional addiction genes

(dataset A=GSE30784; B=TCGA-OSCC; C=RNA-Seq)

Arrows in the column indicate an increased (↑) or decreased (↓) expression in the corresponding dataset (P<0.05); ns = not significant.
