# Supplementary material for: Targeting CDK9-dependent transcriptional addiction: a novel chemoprevention strategy for oral carcinogenesis via adenosine deaminase modulation
Source: Cell Death Dis. 2025 Dec 8;16(1):881. doi: 10.1038/s41419-025-08224-5 (PMC12686051; doi:10.1038/s41419-025-08224-5)

Figure1E

p-RNA Pol II (Ser2)


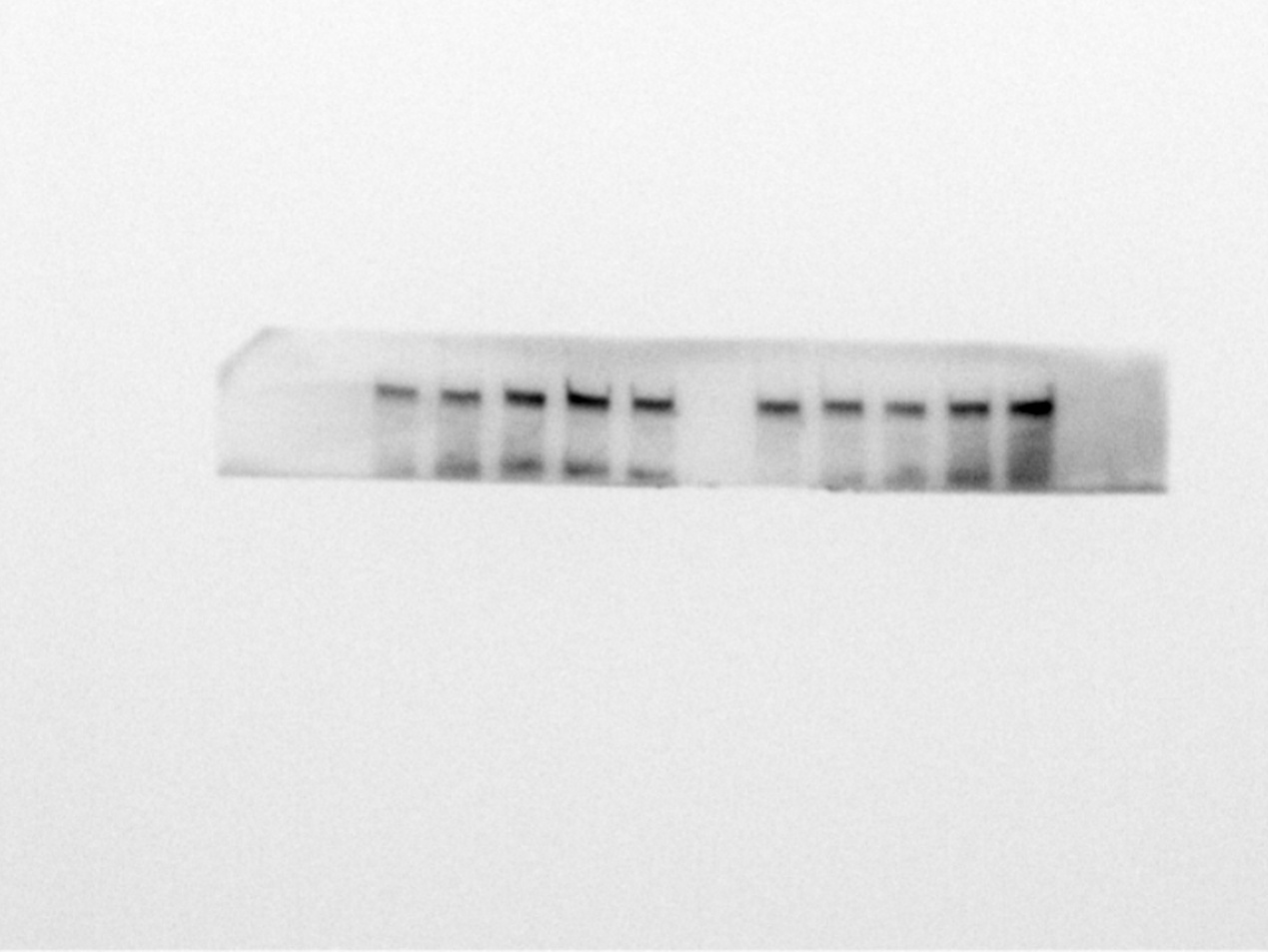


RNA Pol II


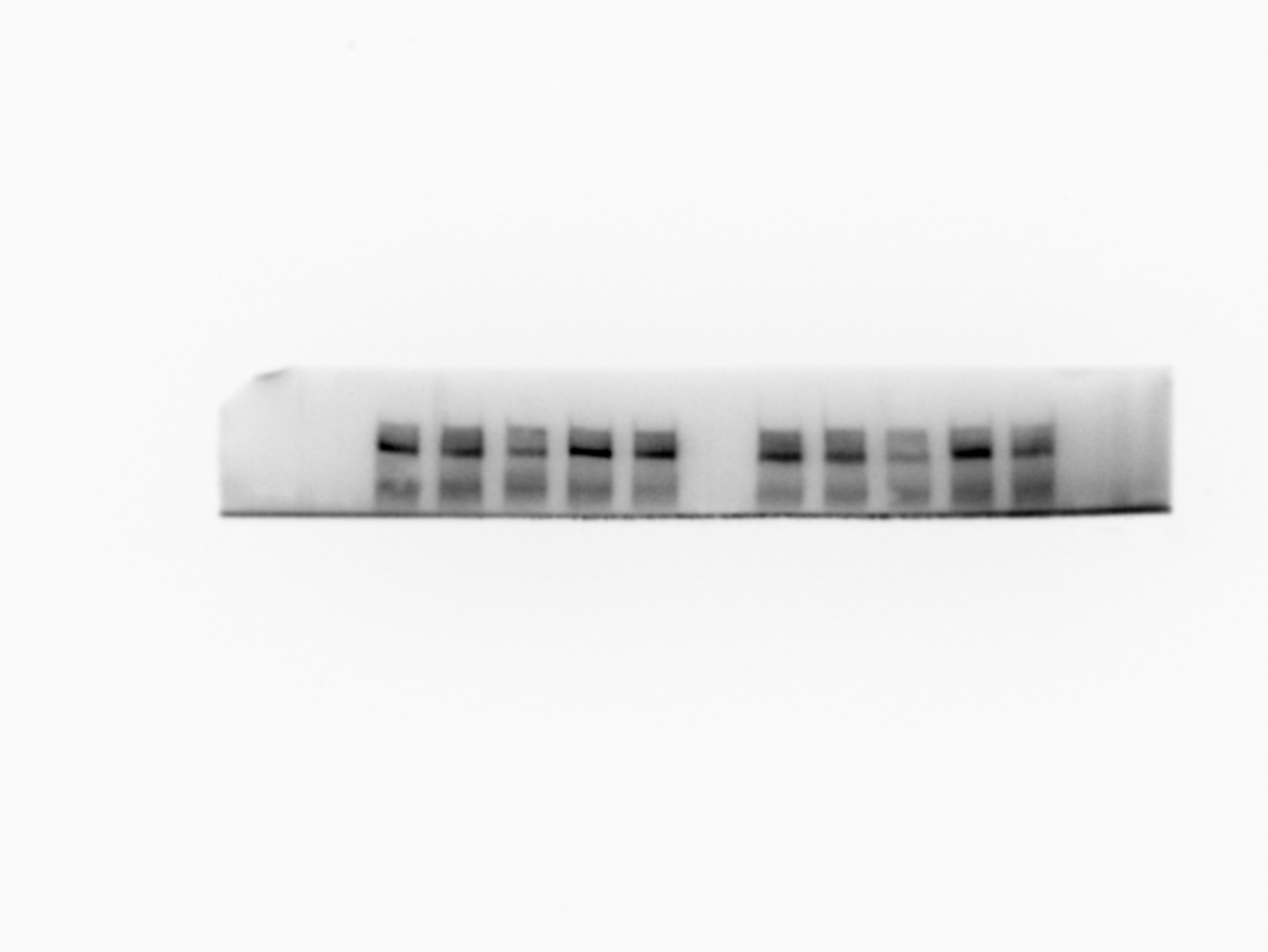


p-RNA Pol II (Thr4)


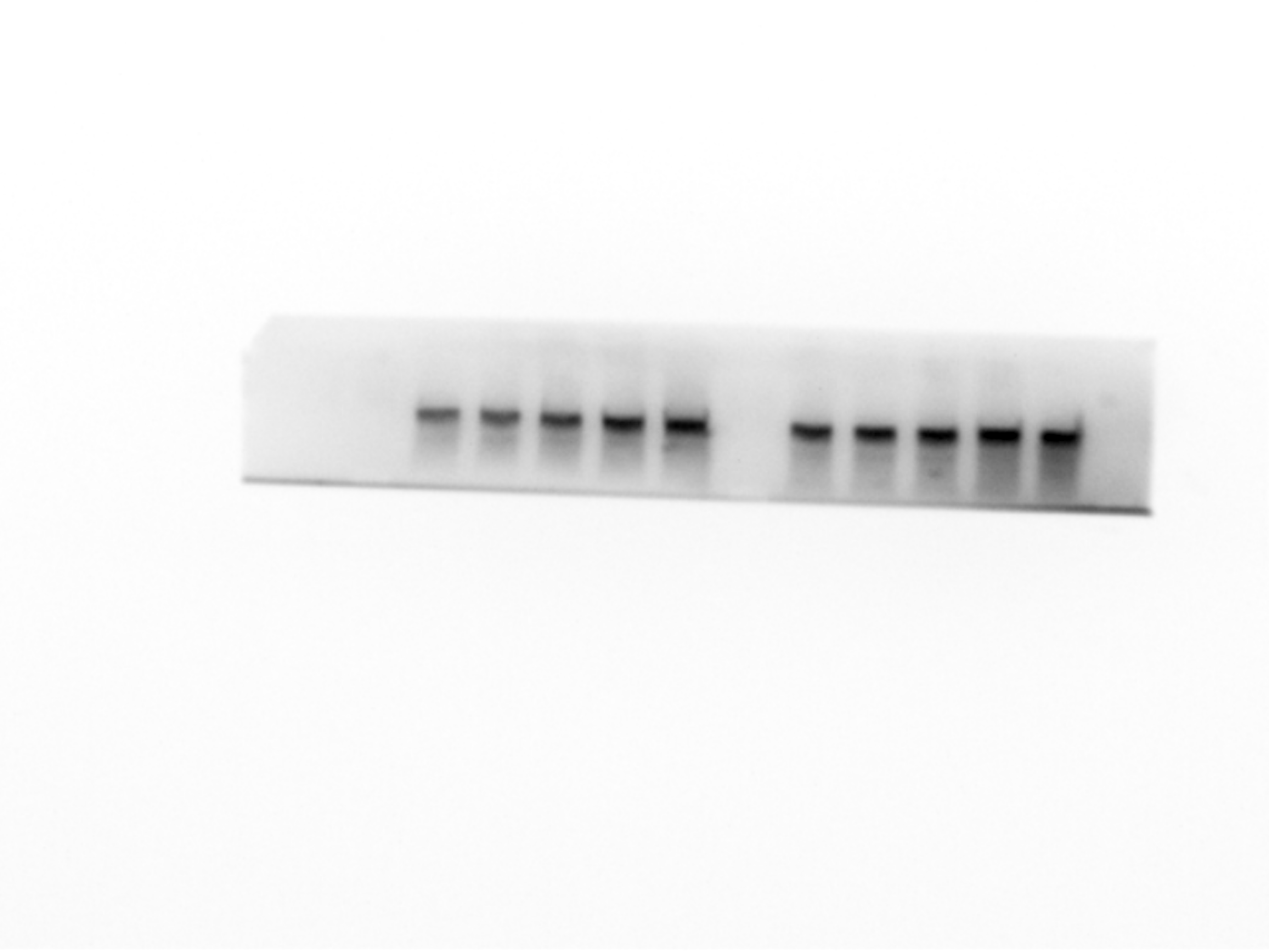


RNA Pol II


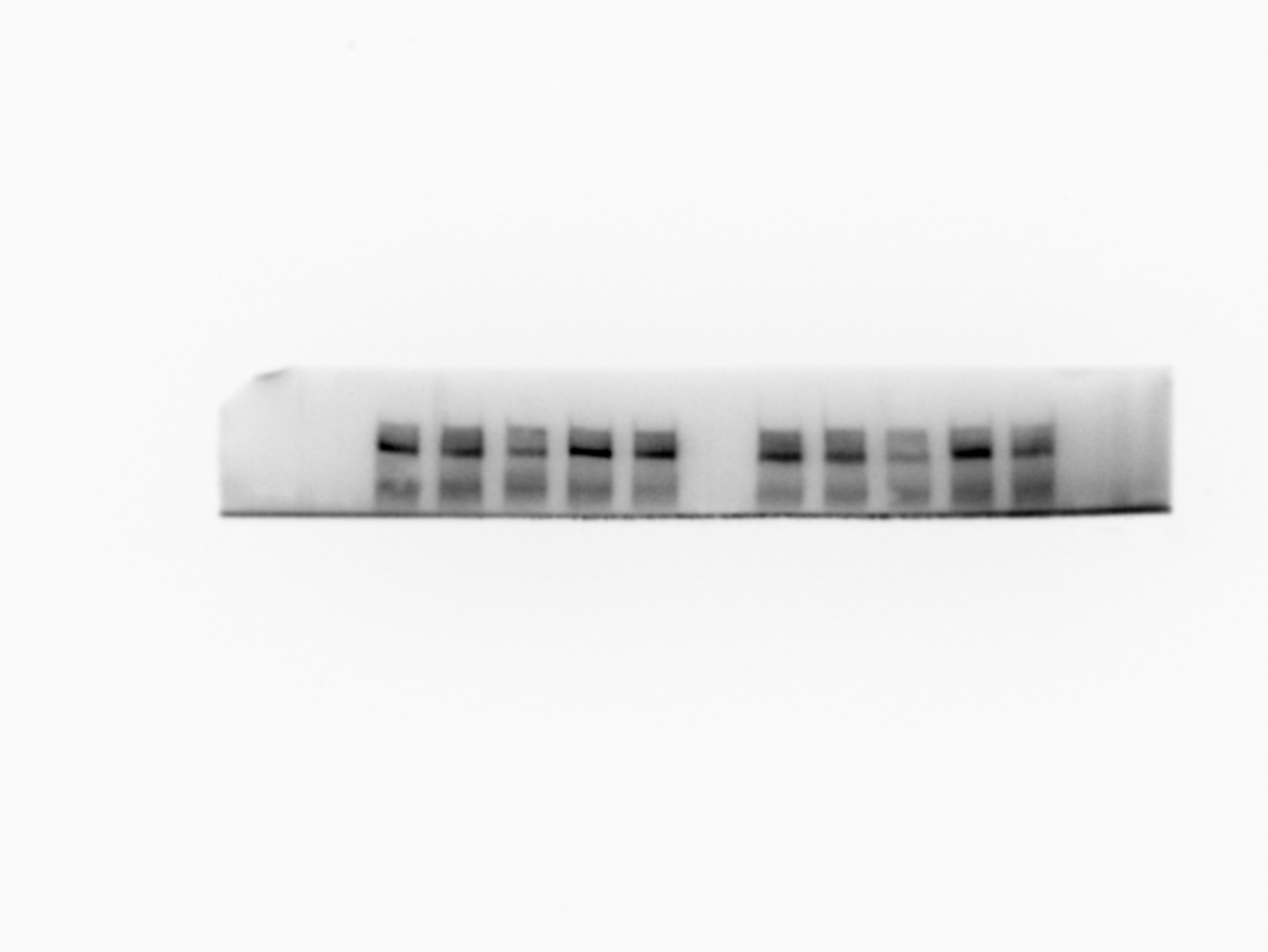


p-RNA Pol II (Ser5)


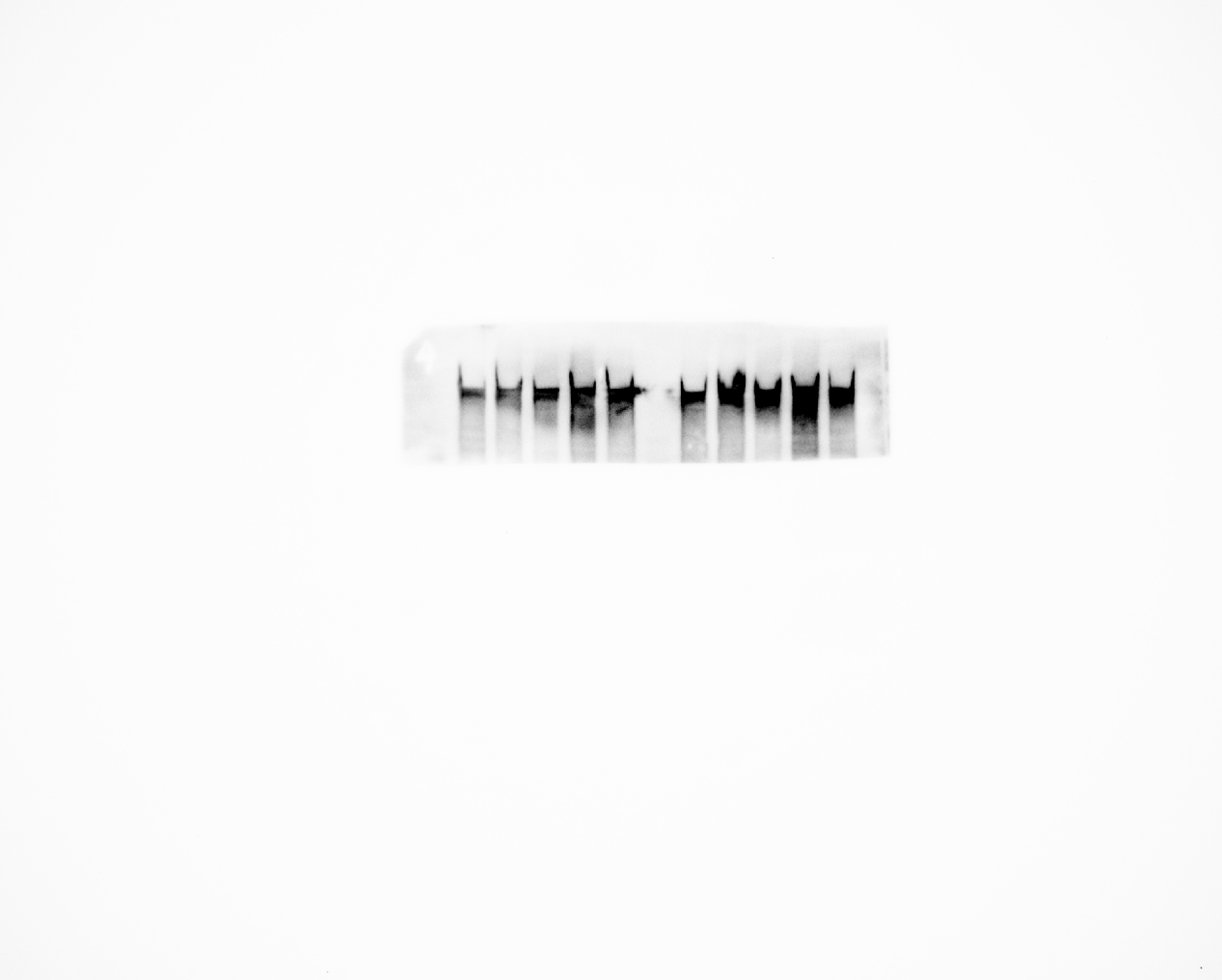


RNA Pol II


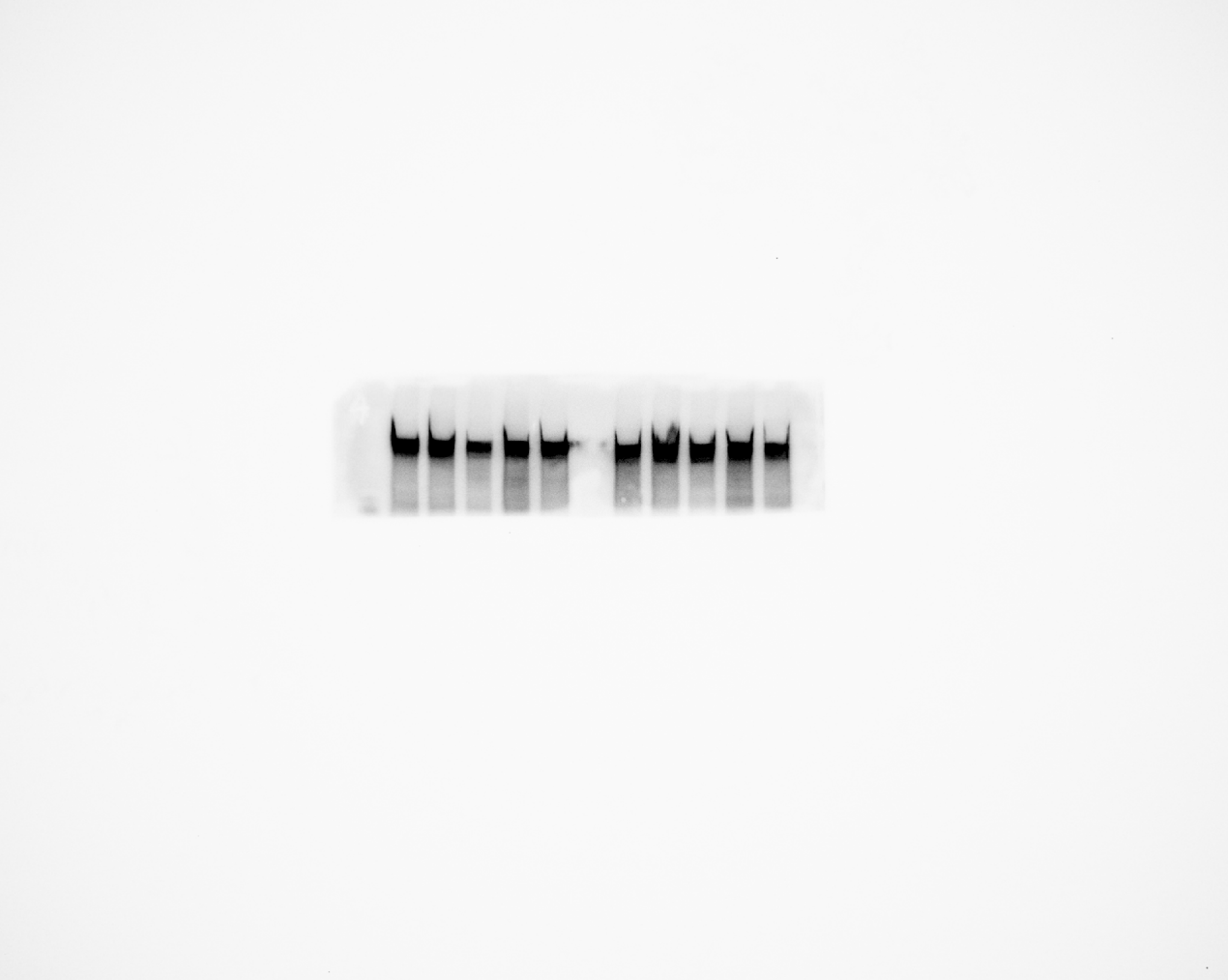


Tubulin


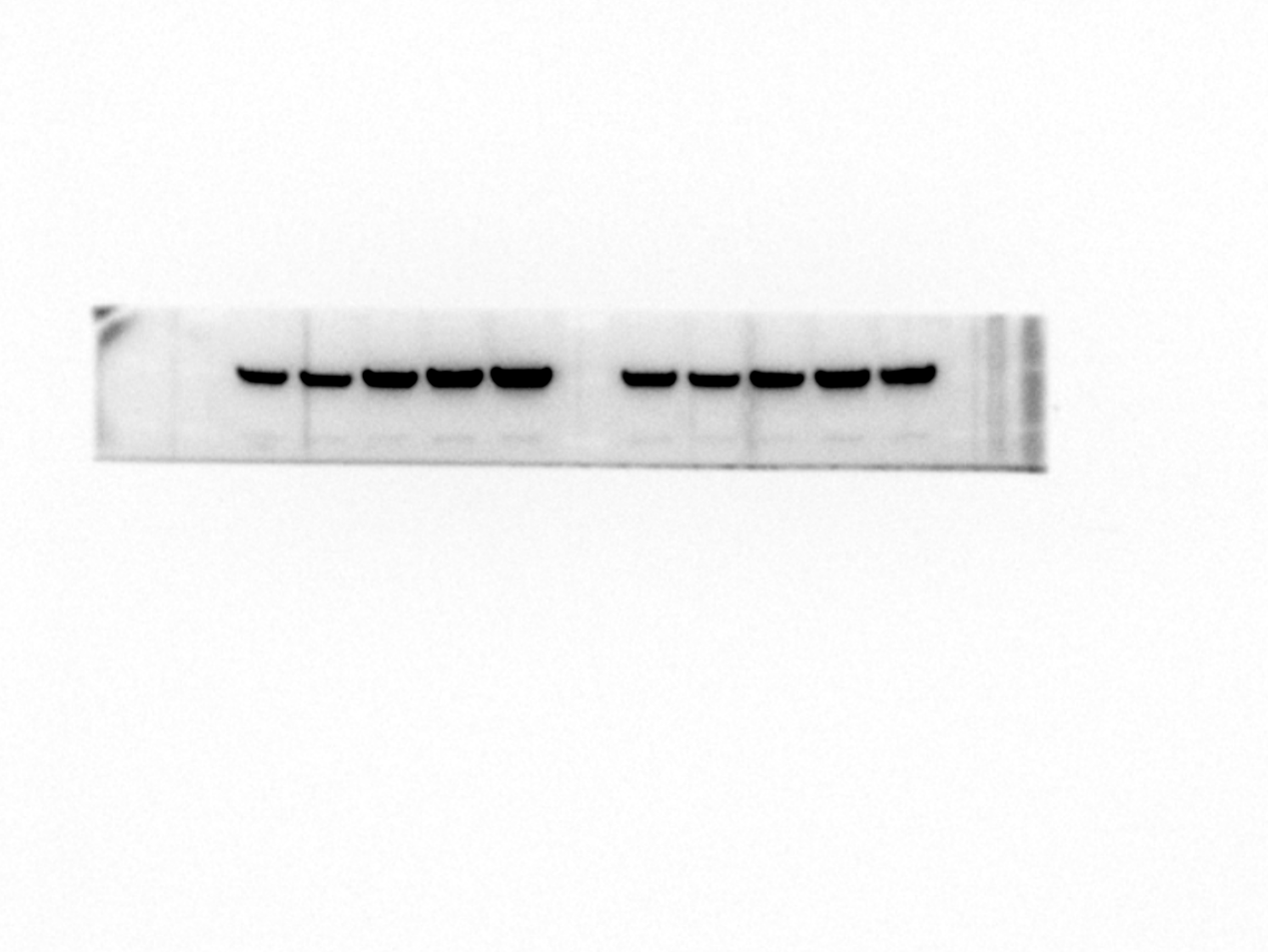


Figure2G

CDK9


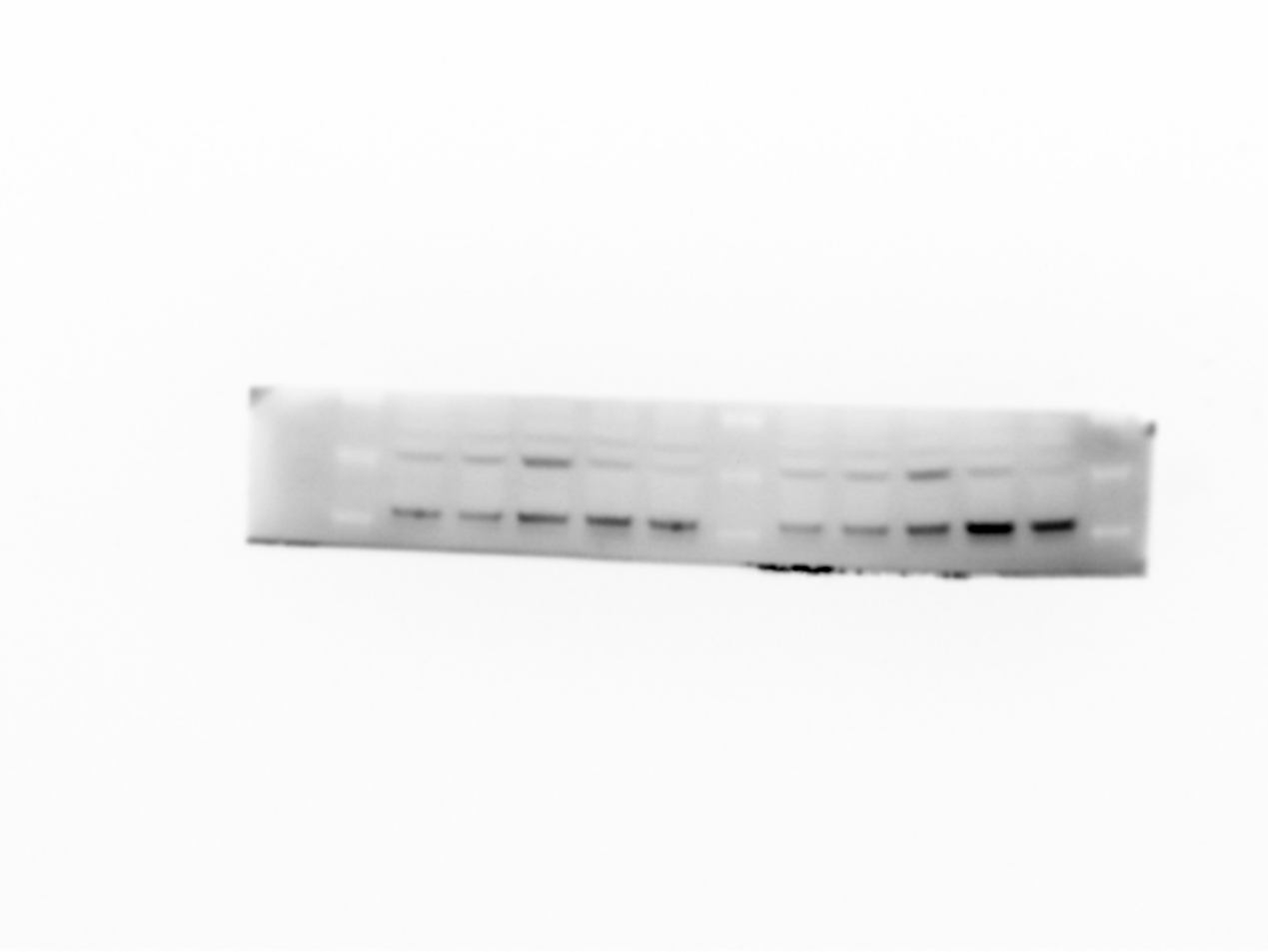


p-RNA Pol II (Ser2)


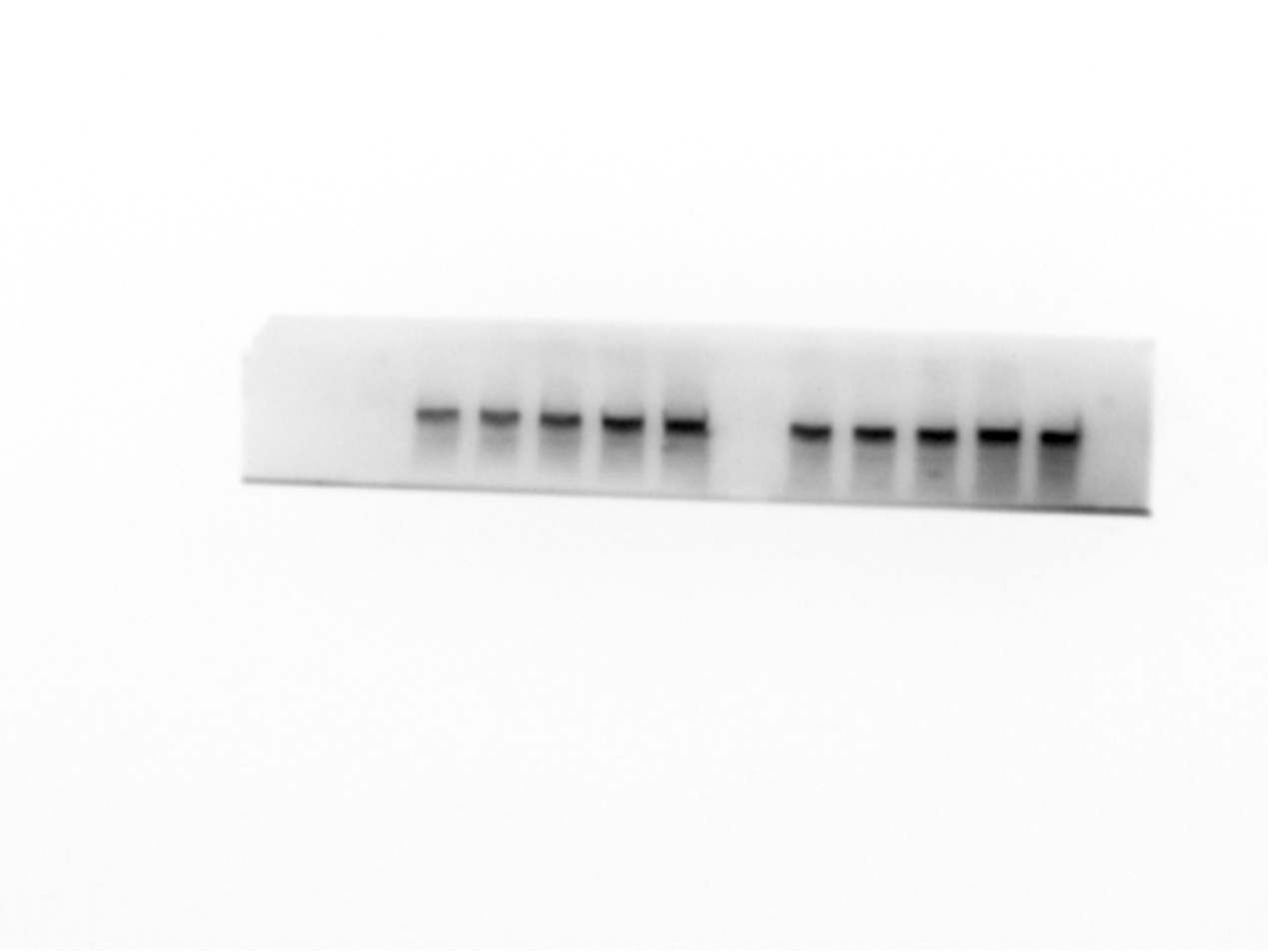


Tubulin


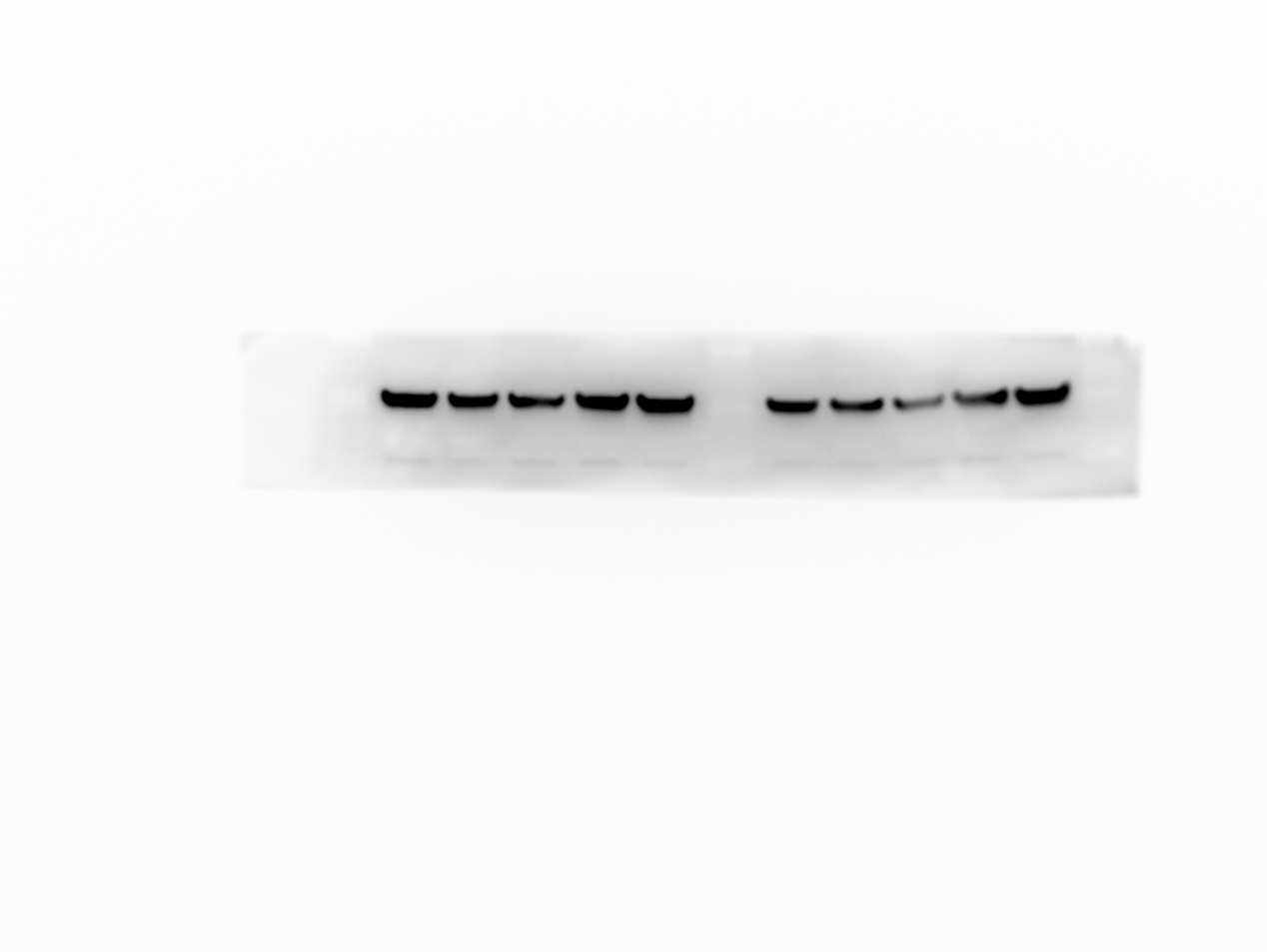


Figure3C

DOK

CDK9


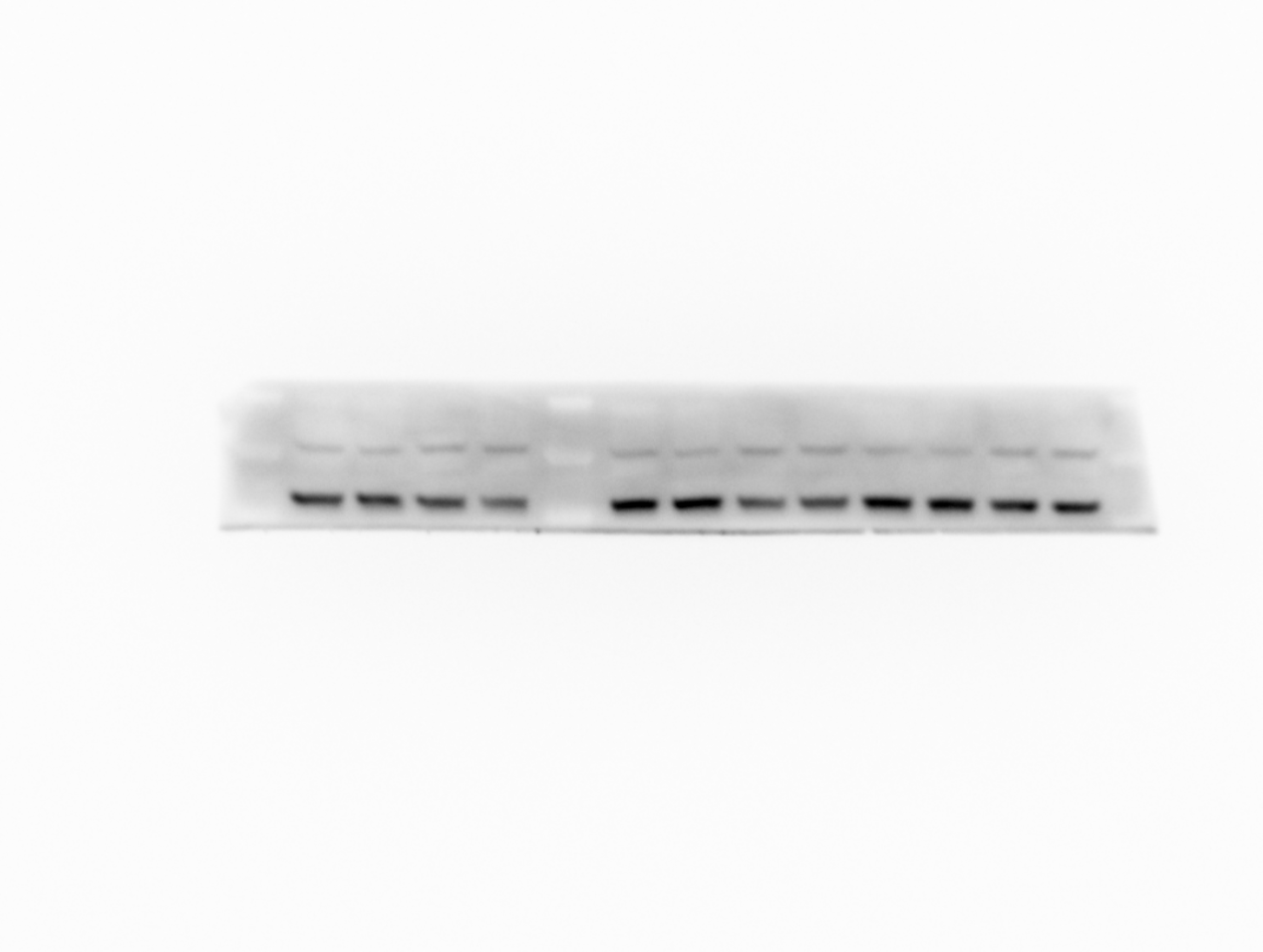


p-RNA Pol II (Ser2)


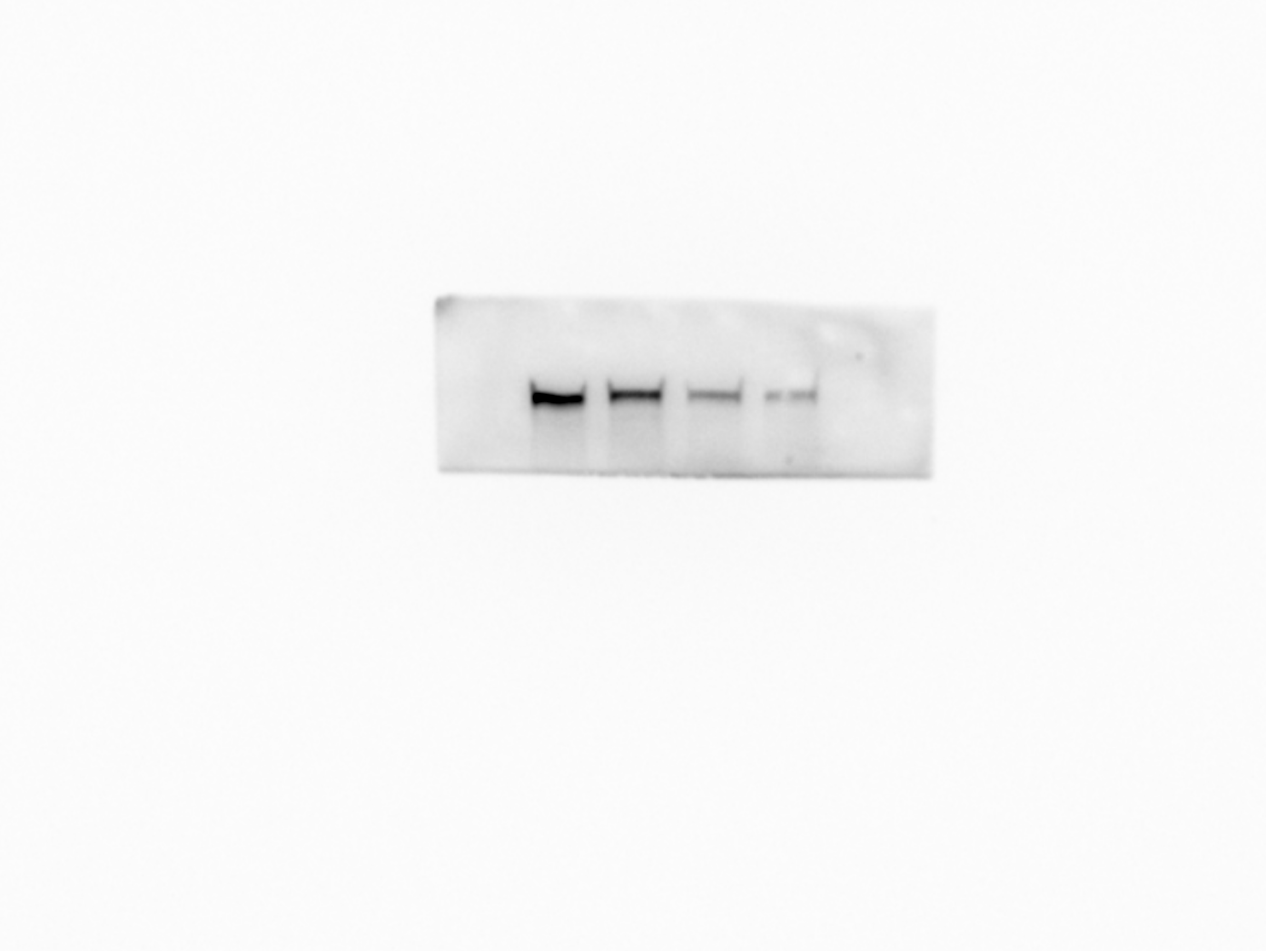


p-RNA Pol II (Thr4)


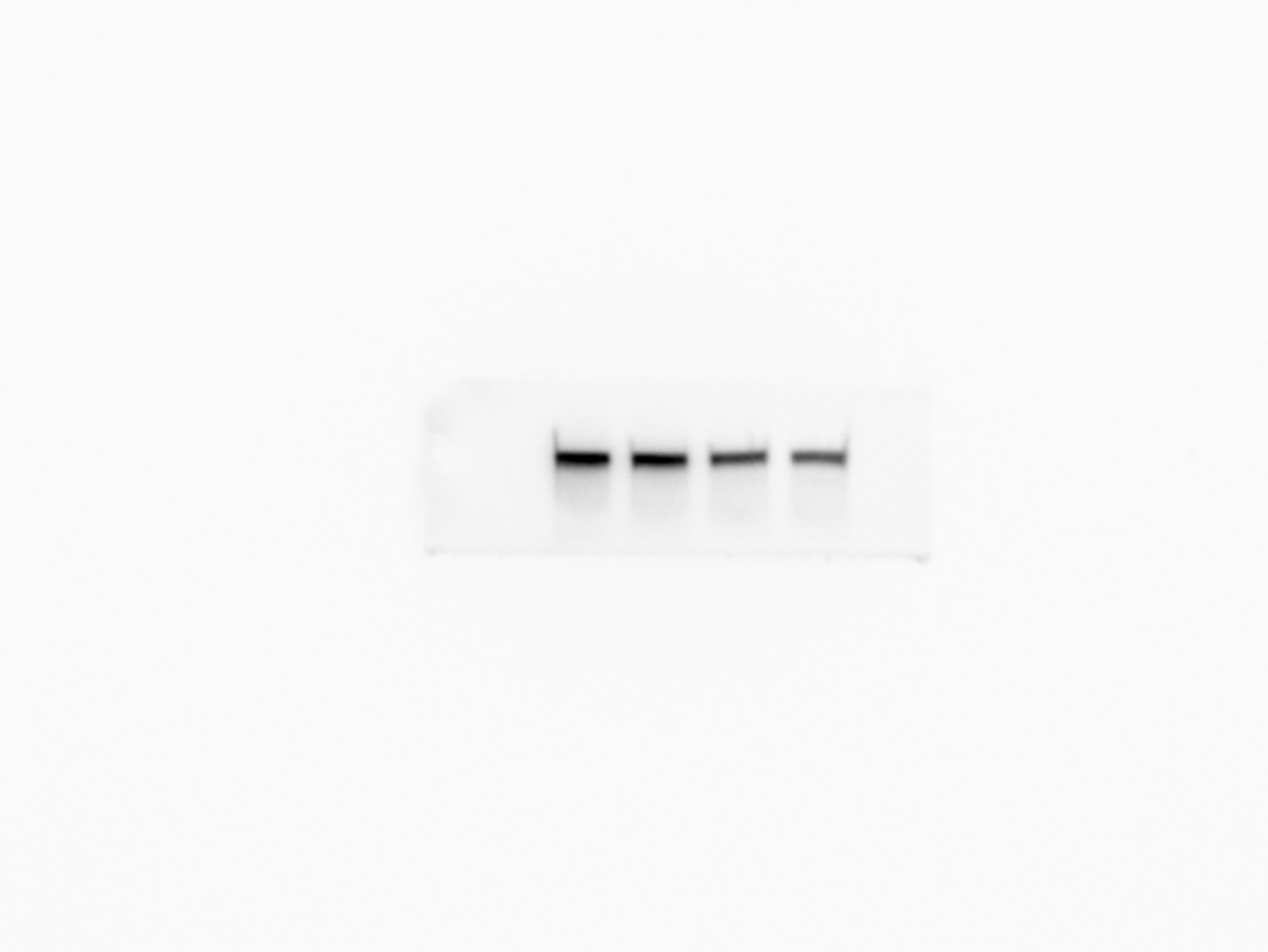


p-RNA Pol II (Ser5)


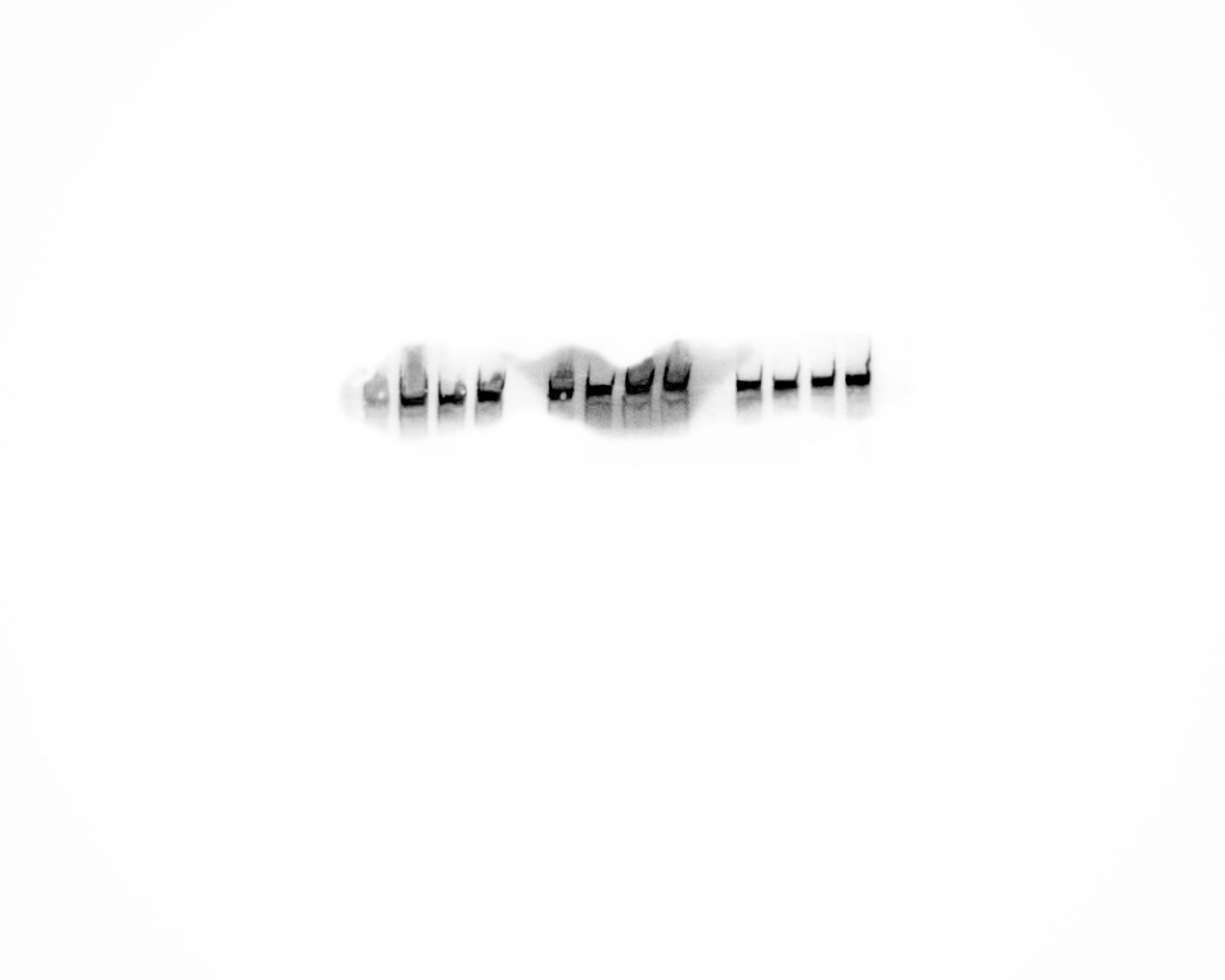


RNA Pol II


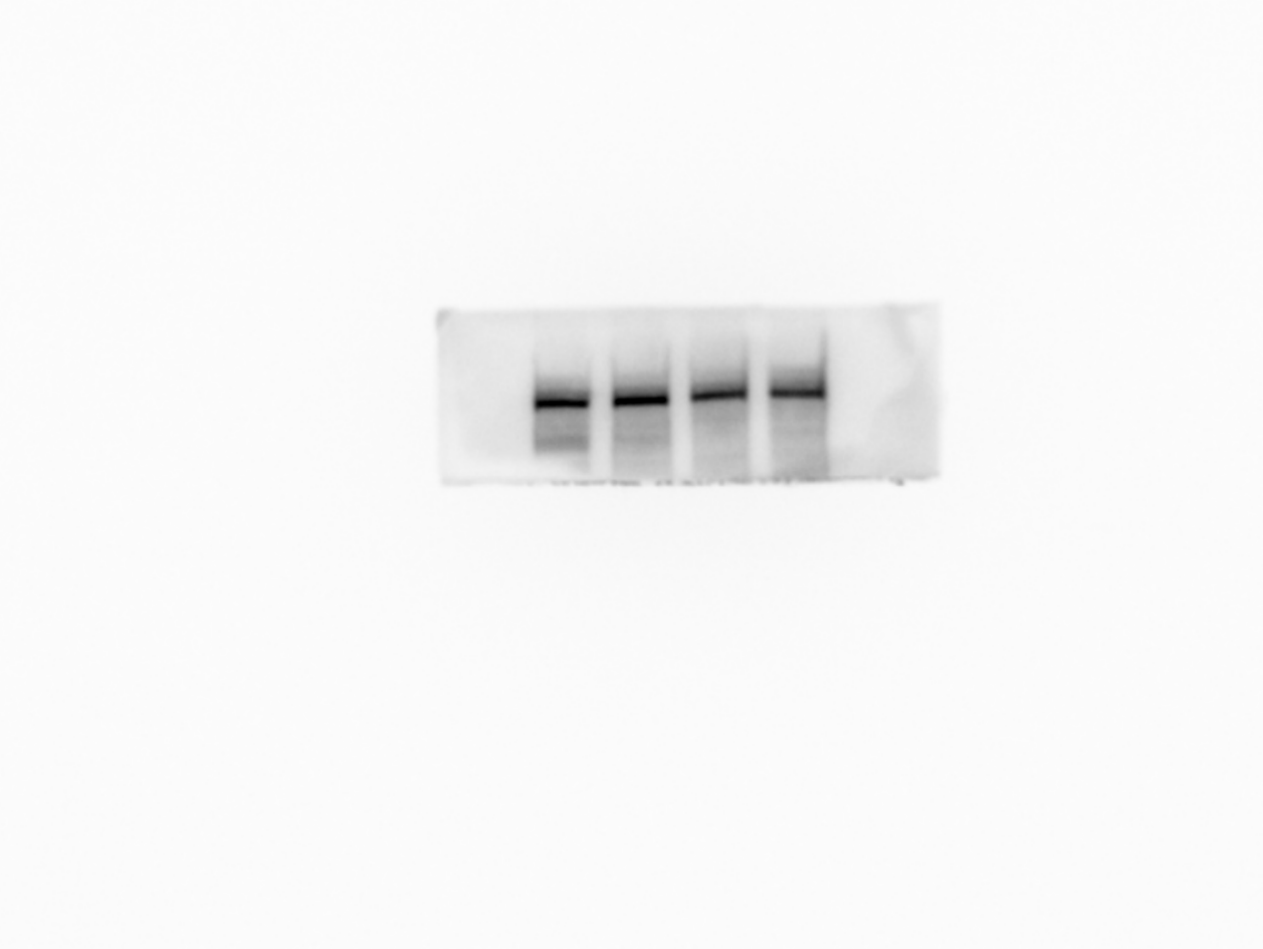


Tubulin


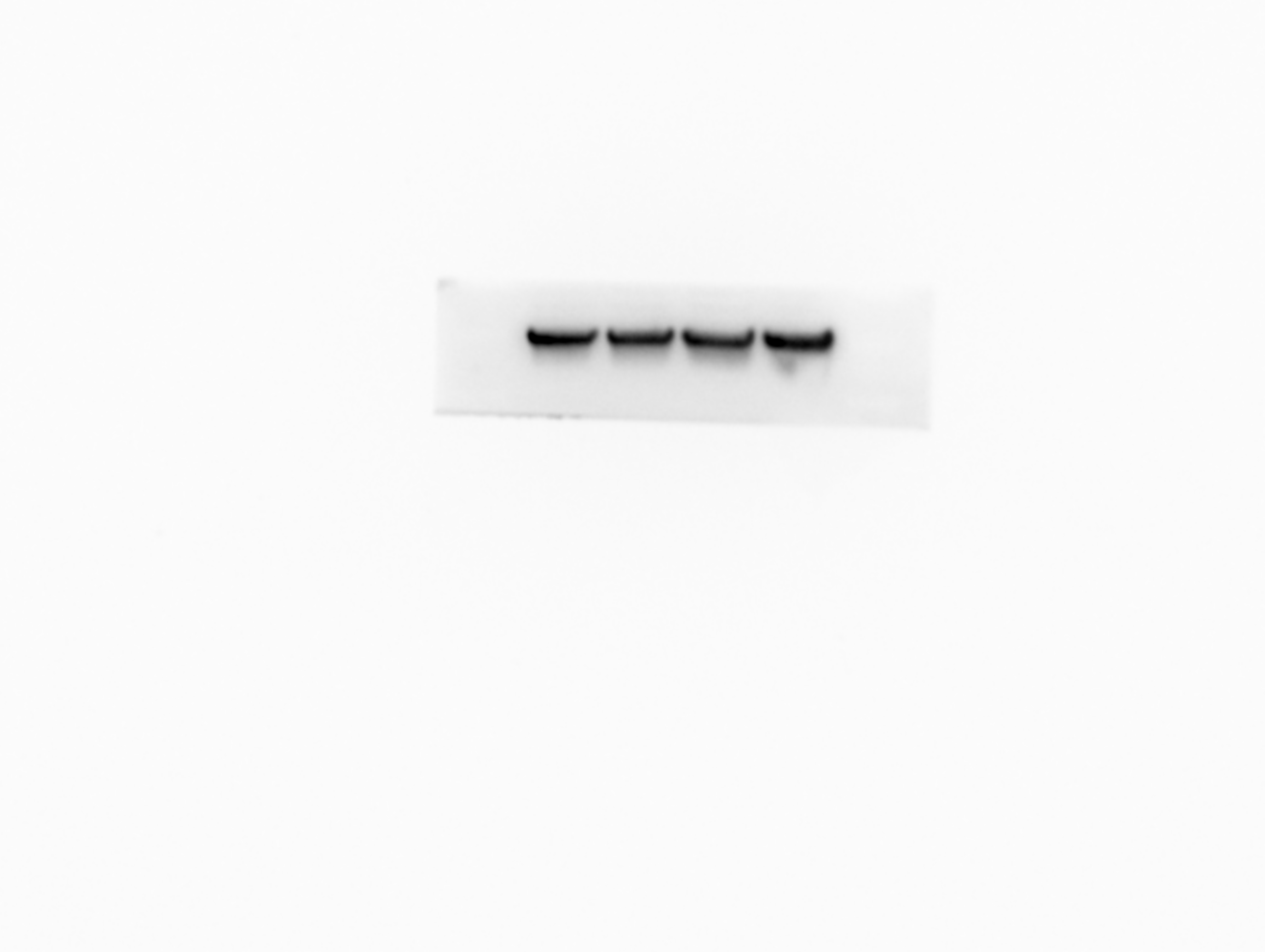


SCC15

CDK9


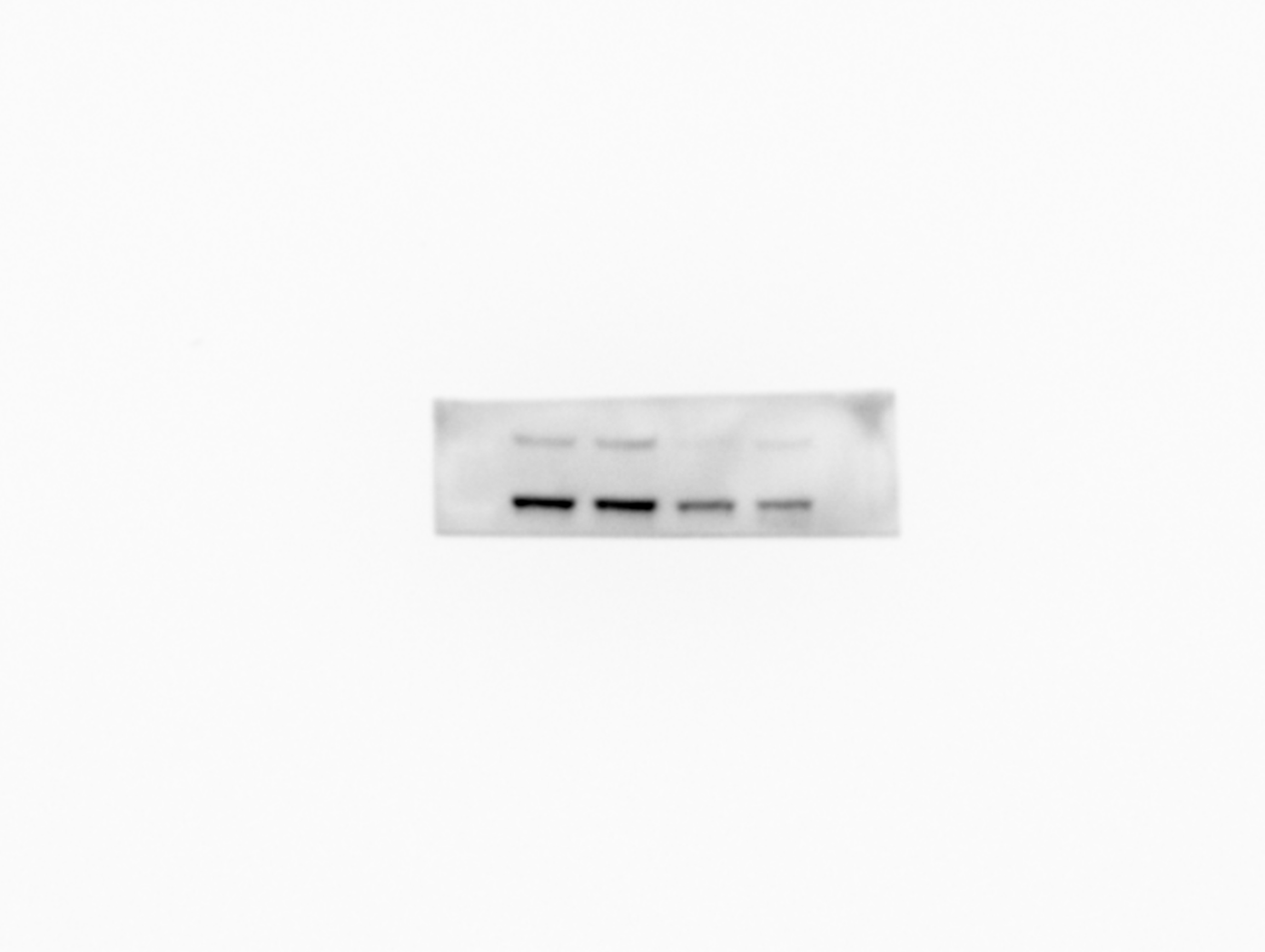


p-RNA Pol II (Ser2)


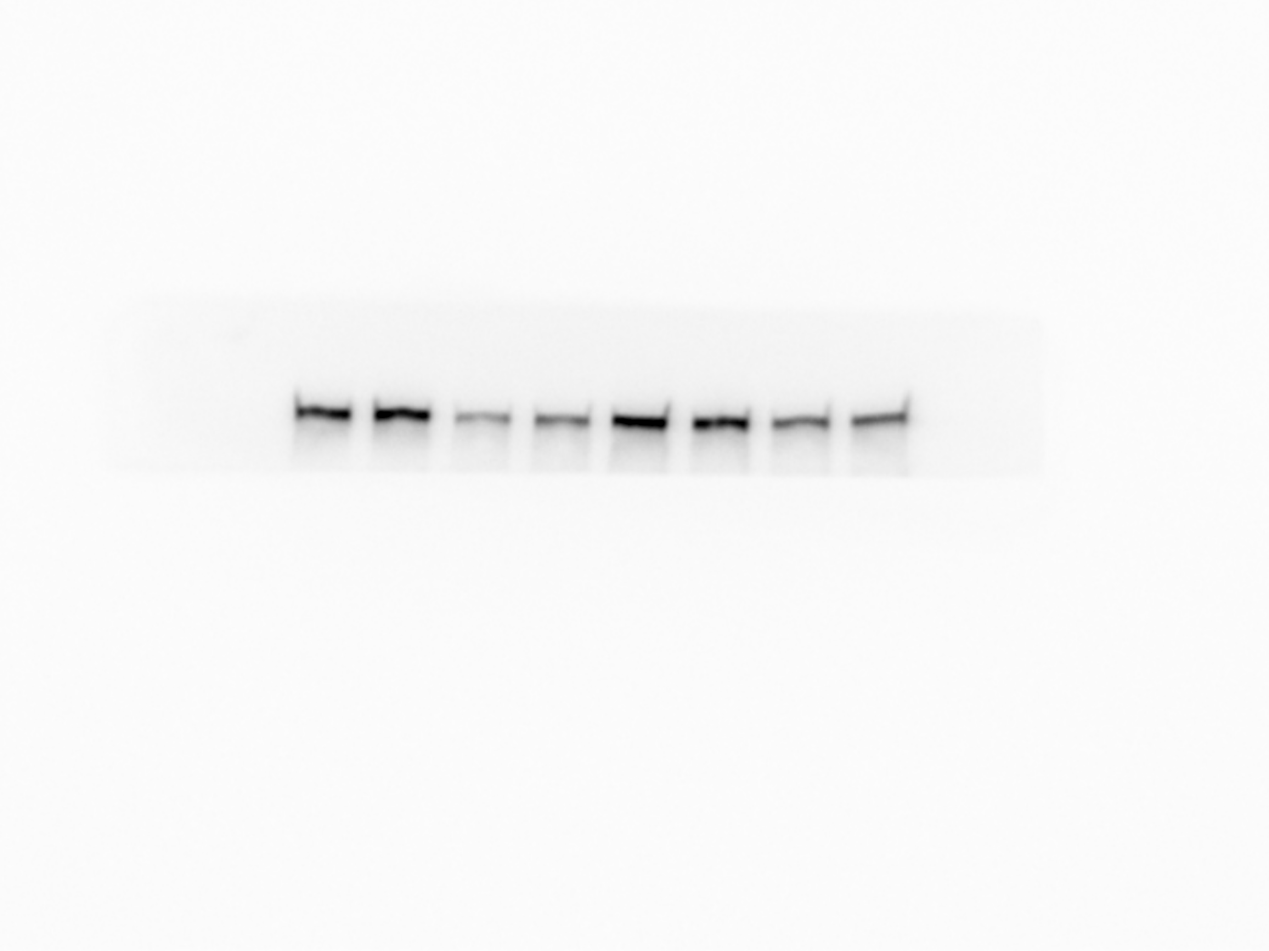


p-RNA Pol II (Thr4)


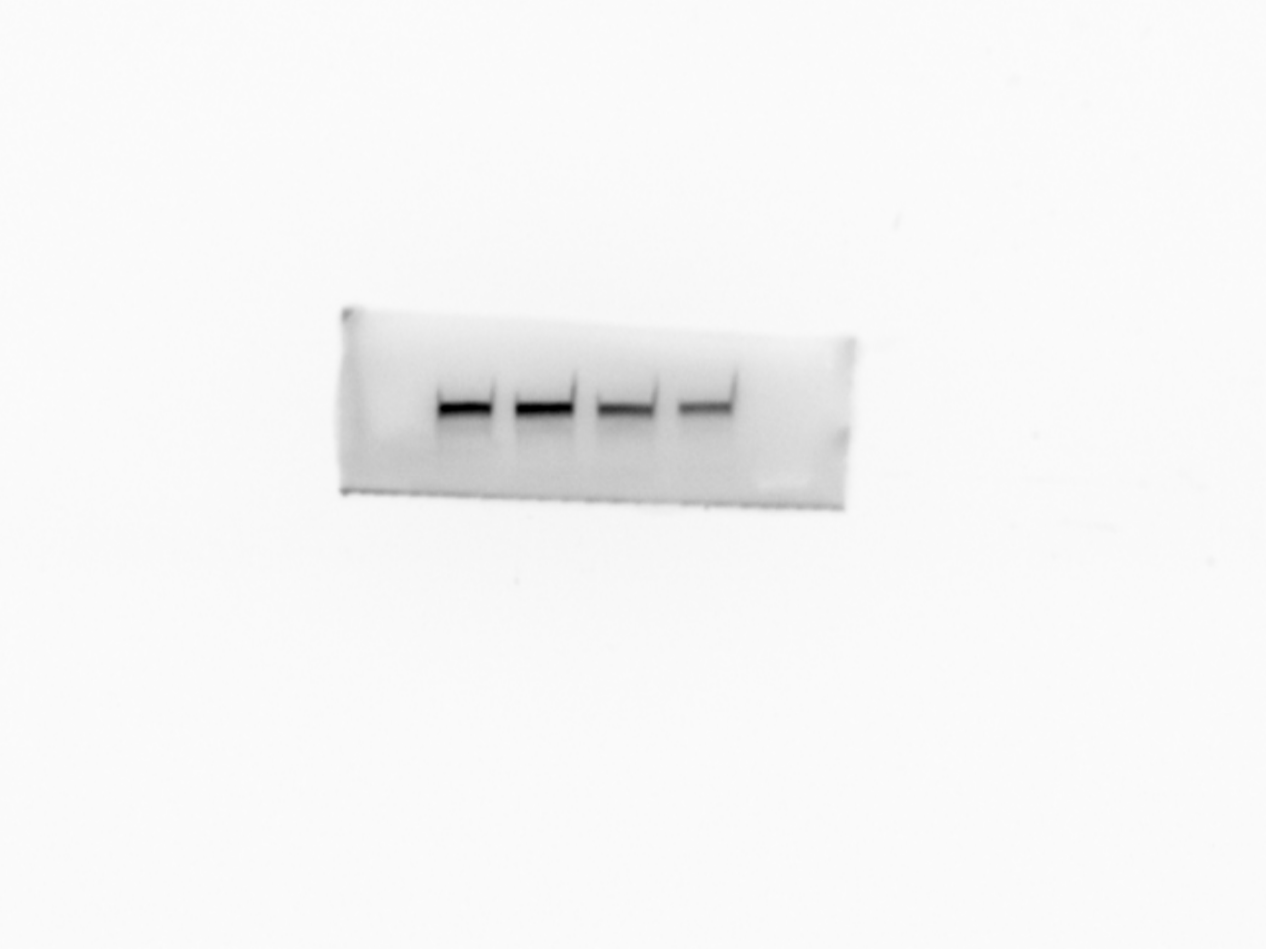


p-RNA Pol II (Ser5)


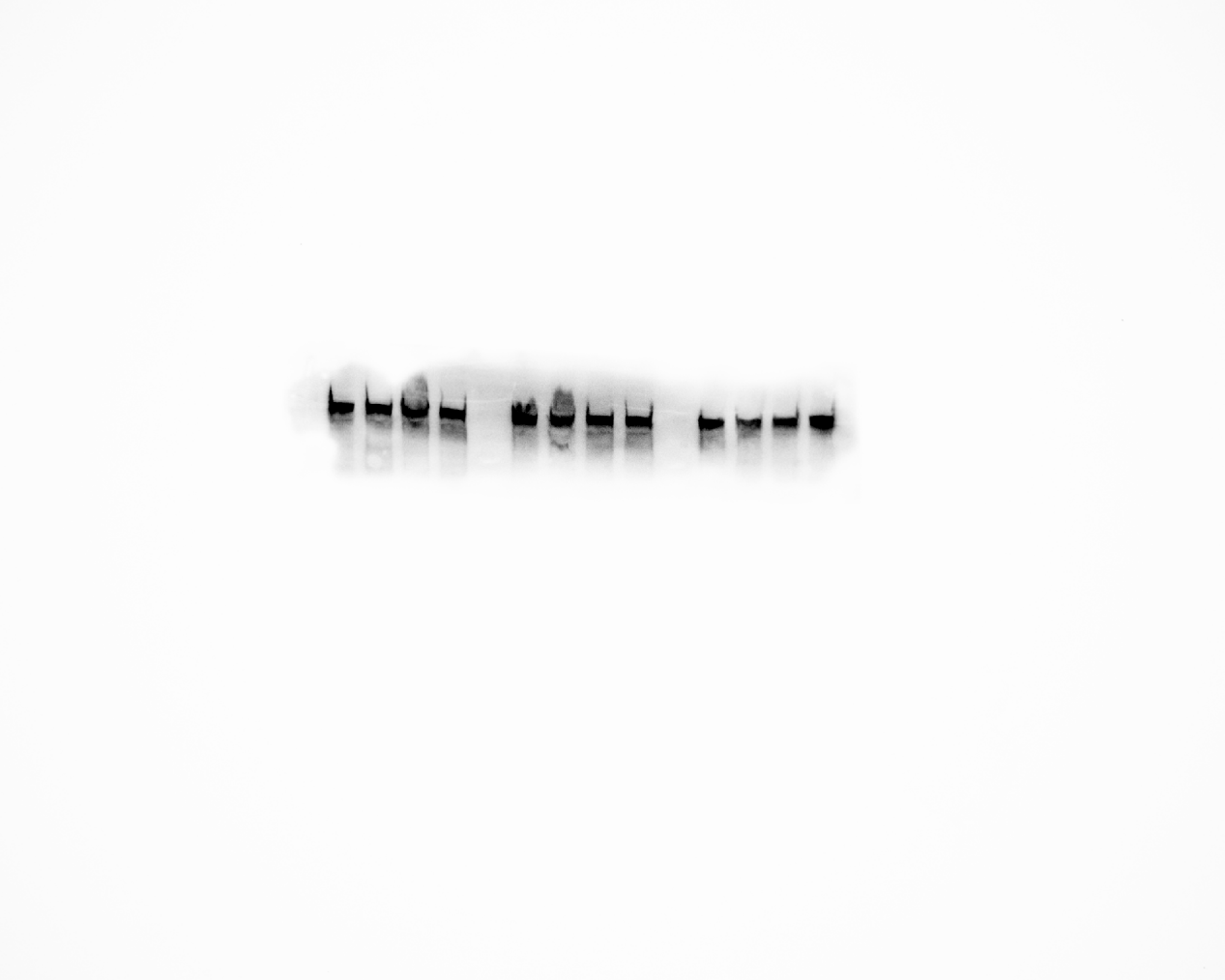


RNA Pol II


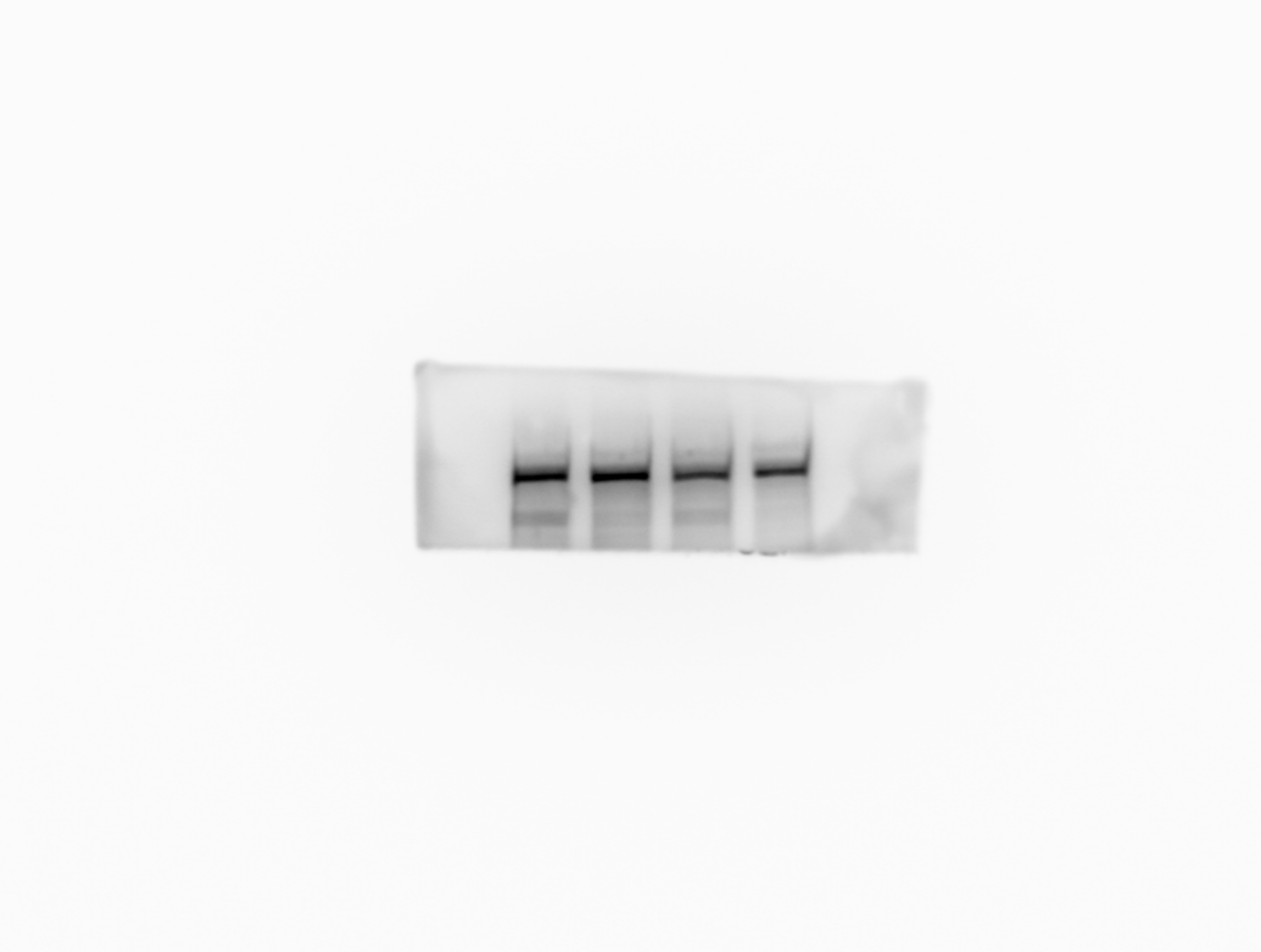


Tubulin


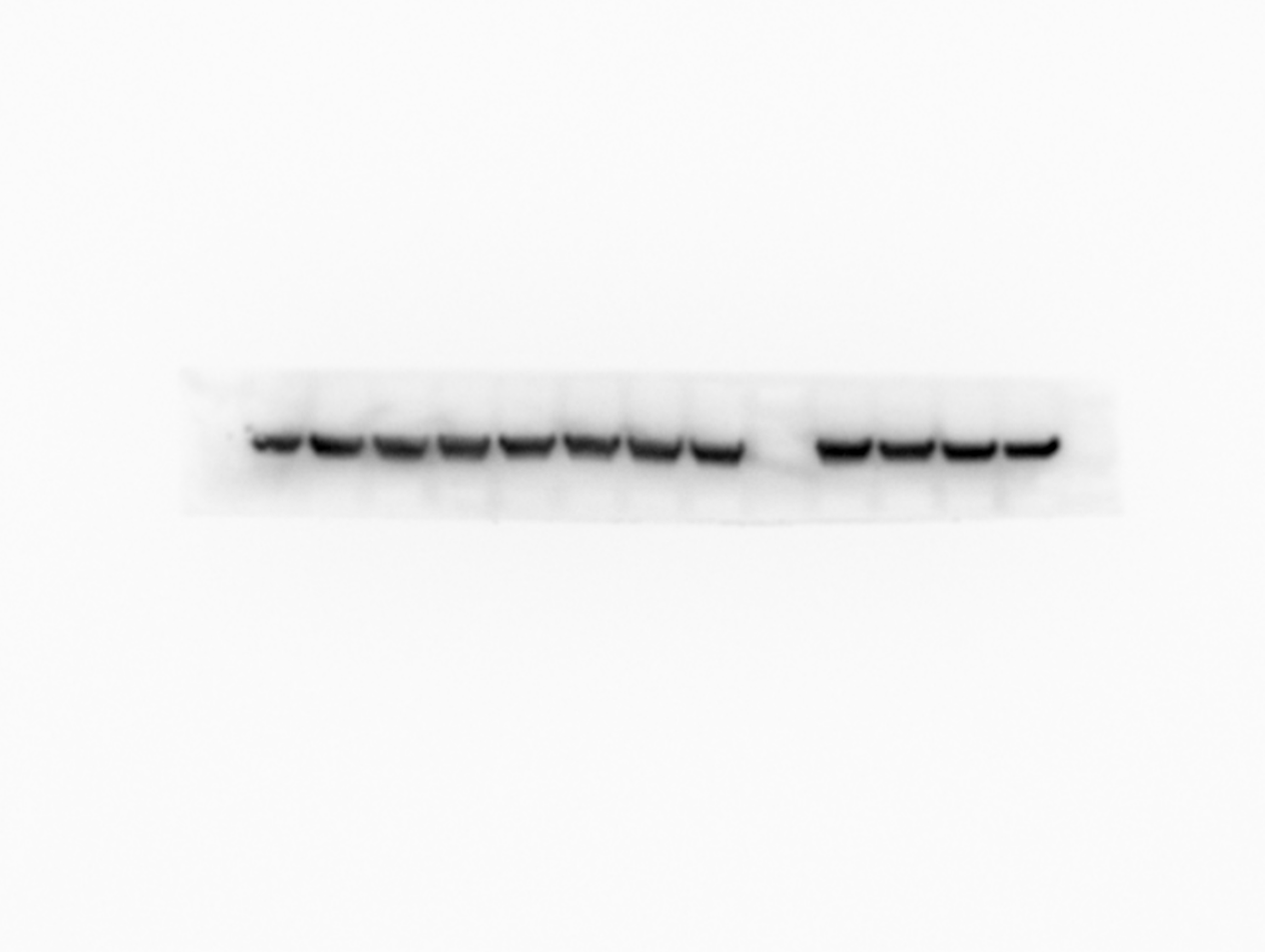


Figure3D

CDK9


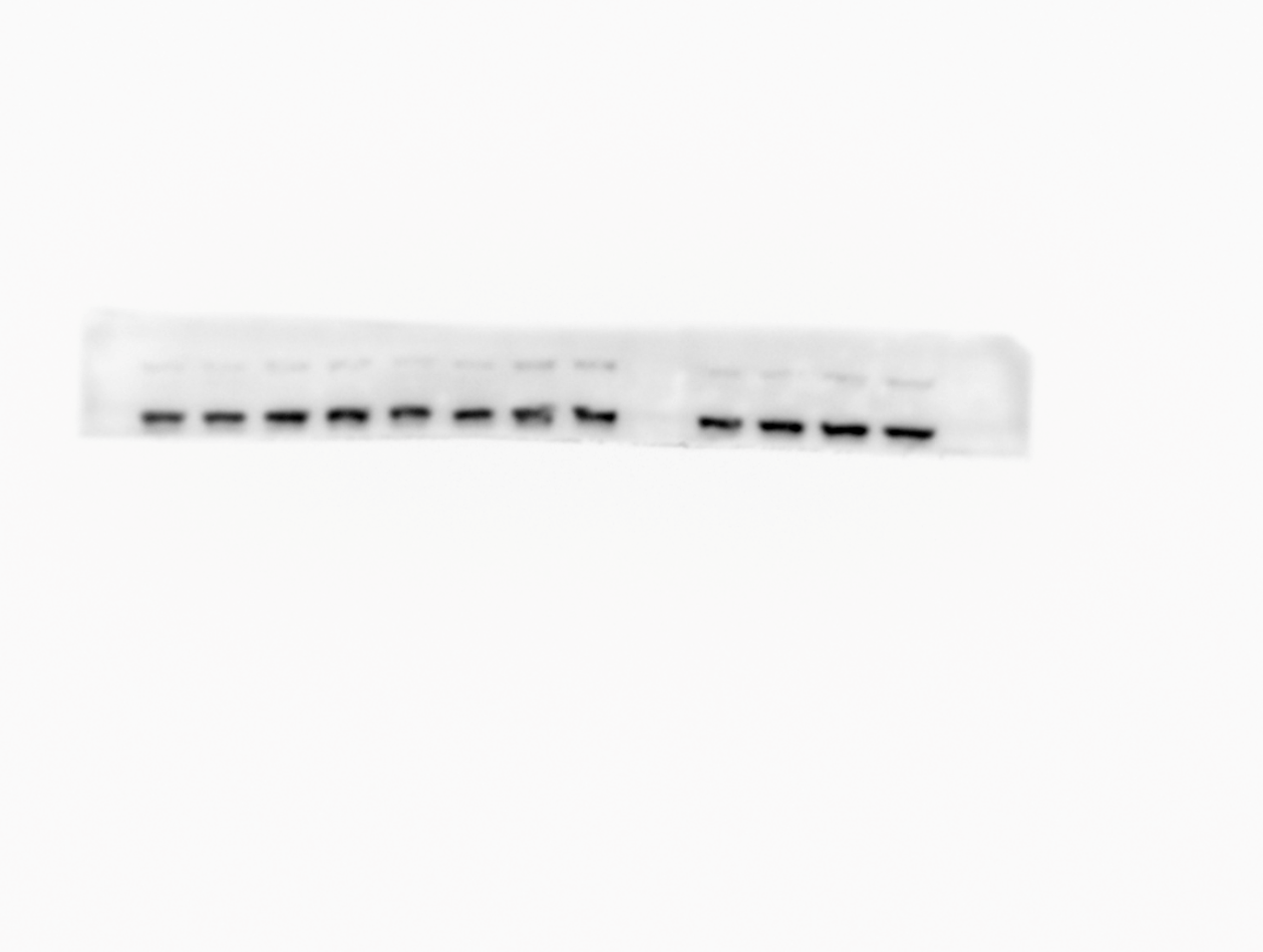


p-RNA Pol II (Ser2)


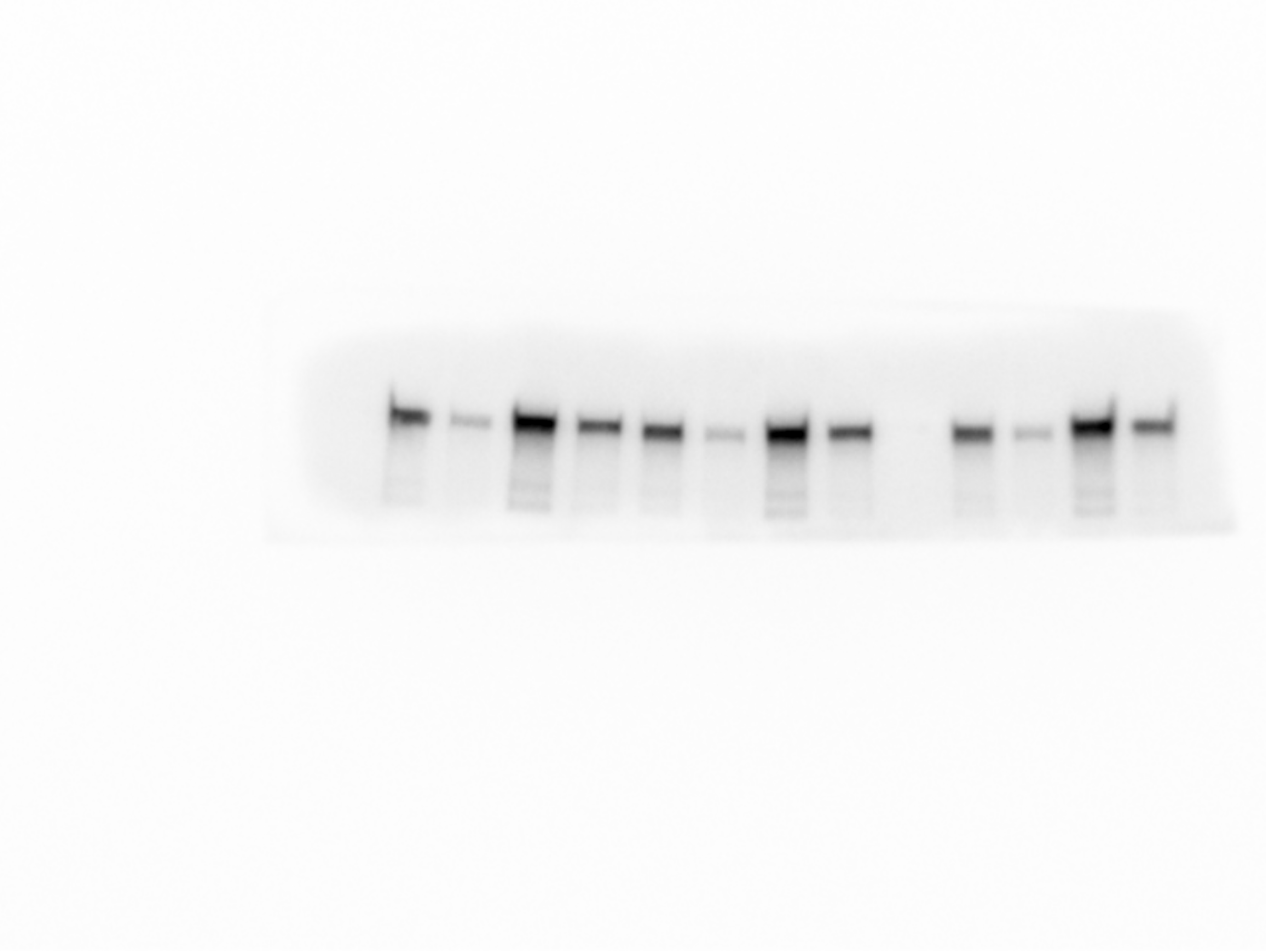


p-RNA Pol II (Thr4)


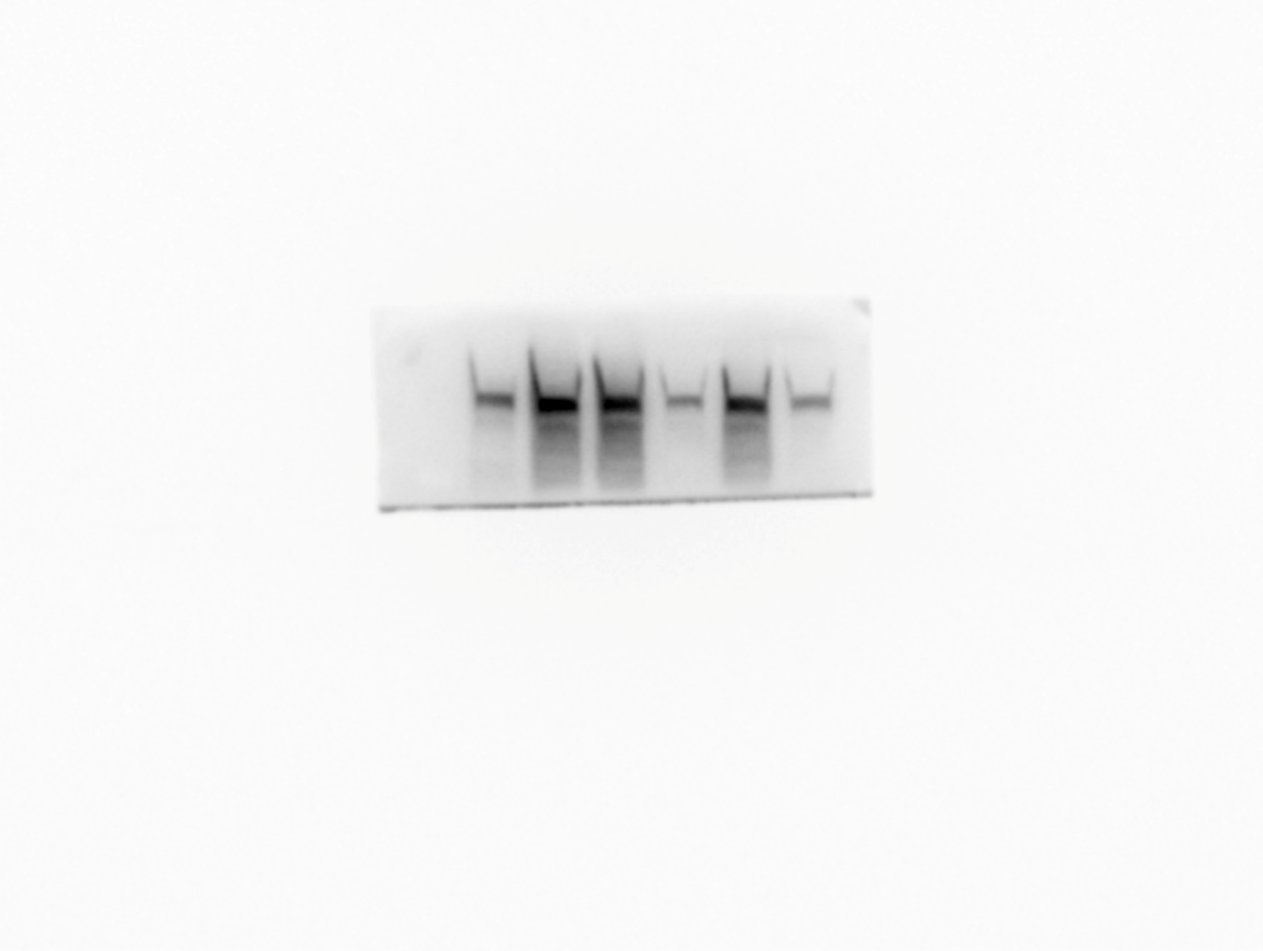


p-RNA Pol II (Ser5)


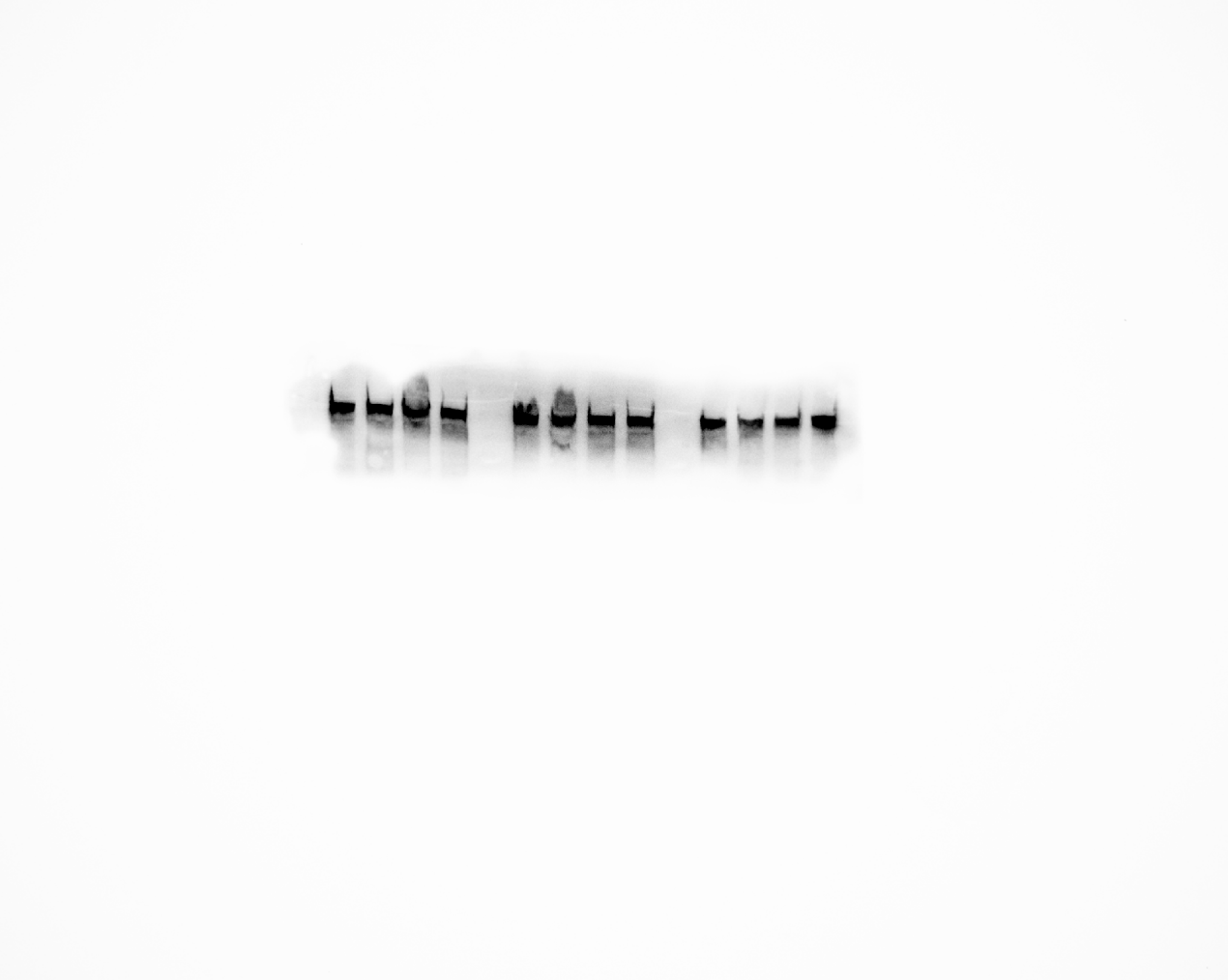


RNA Pol II


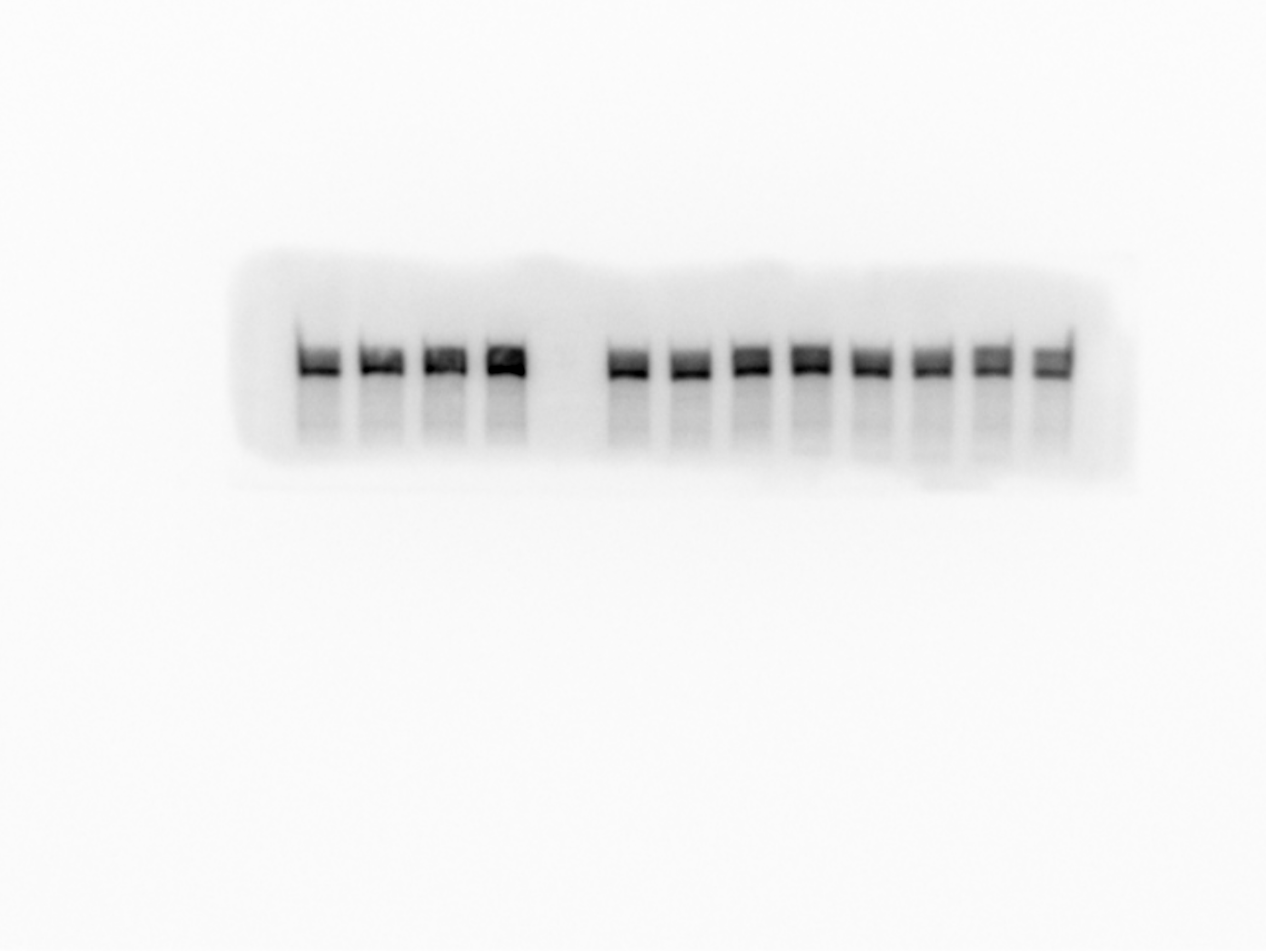


Tubulin


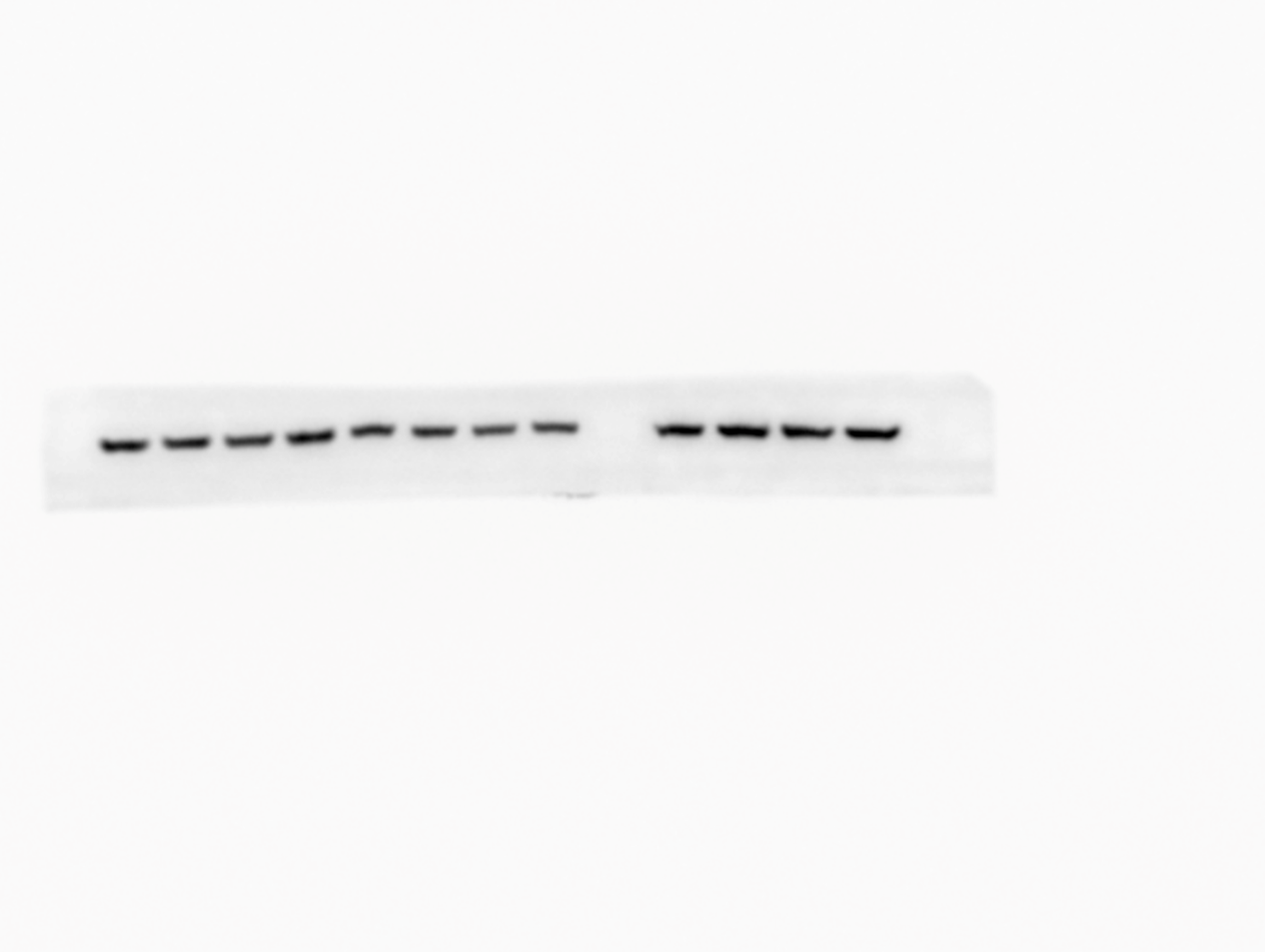


Figure4B

CDK9


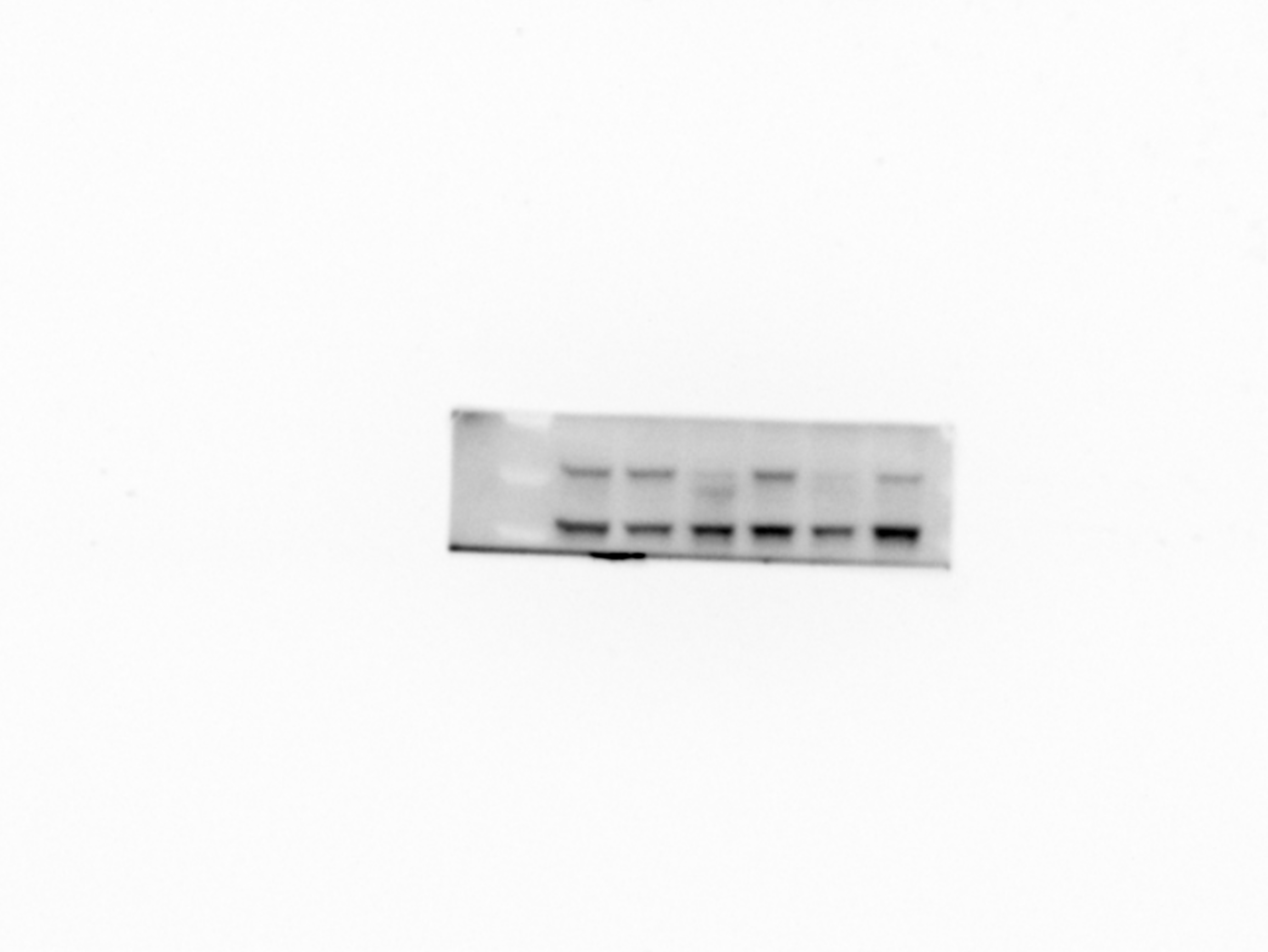


p-RNA Pol II (Ser2)


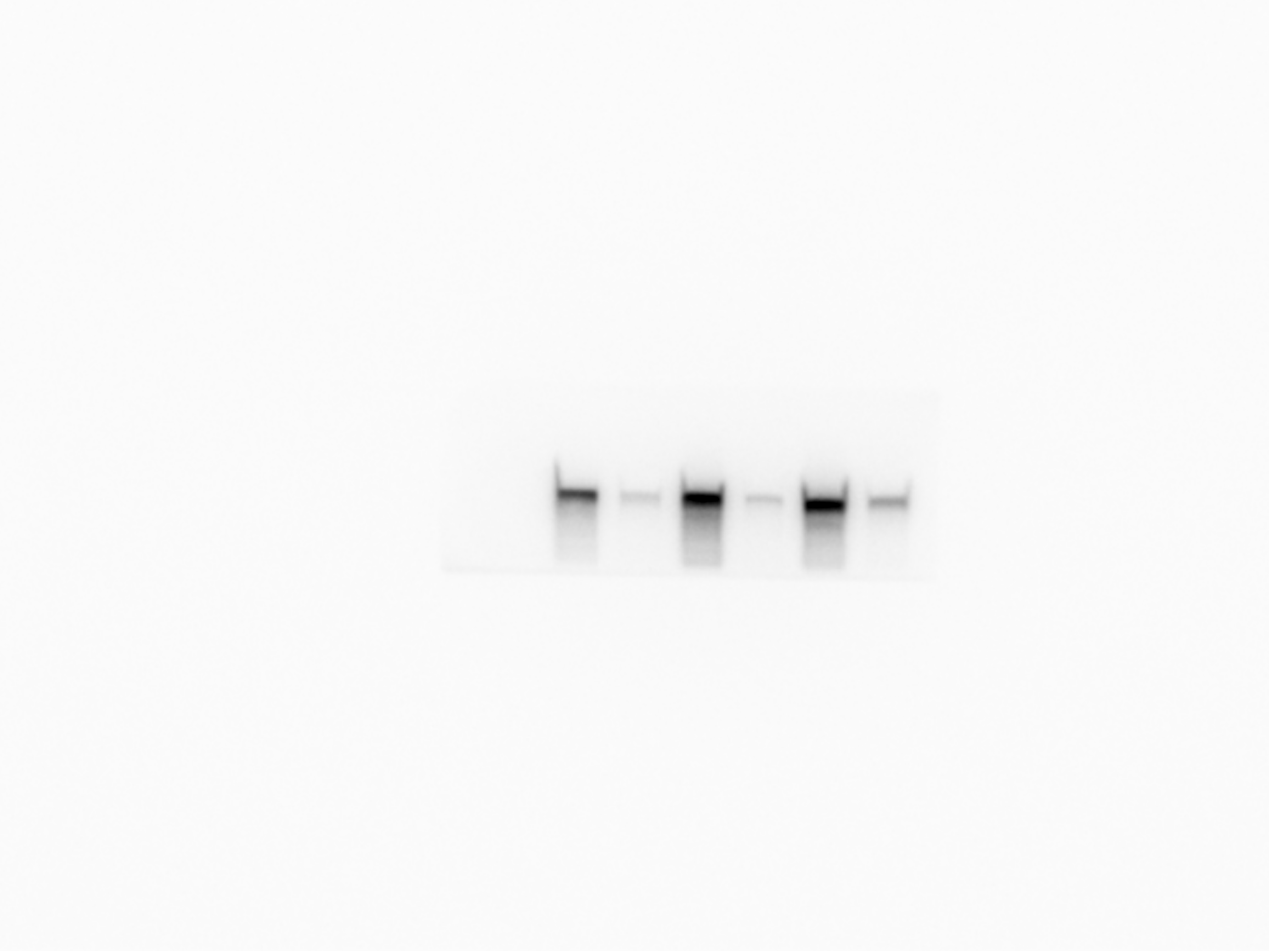


p-RNA Pol II (Thr4)


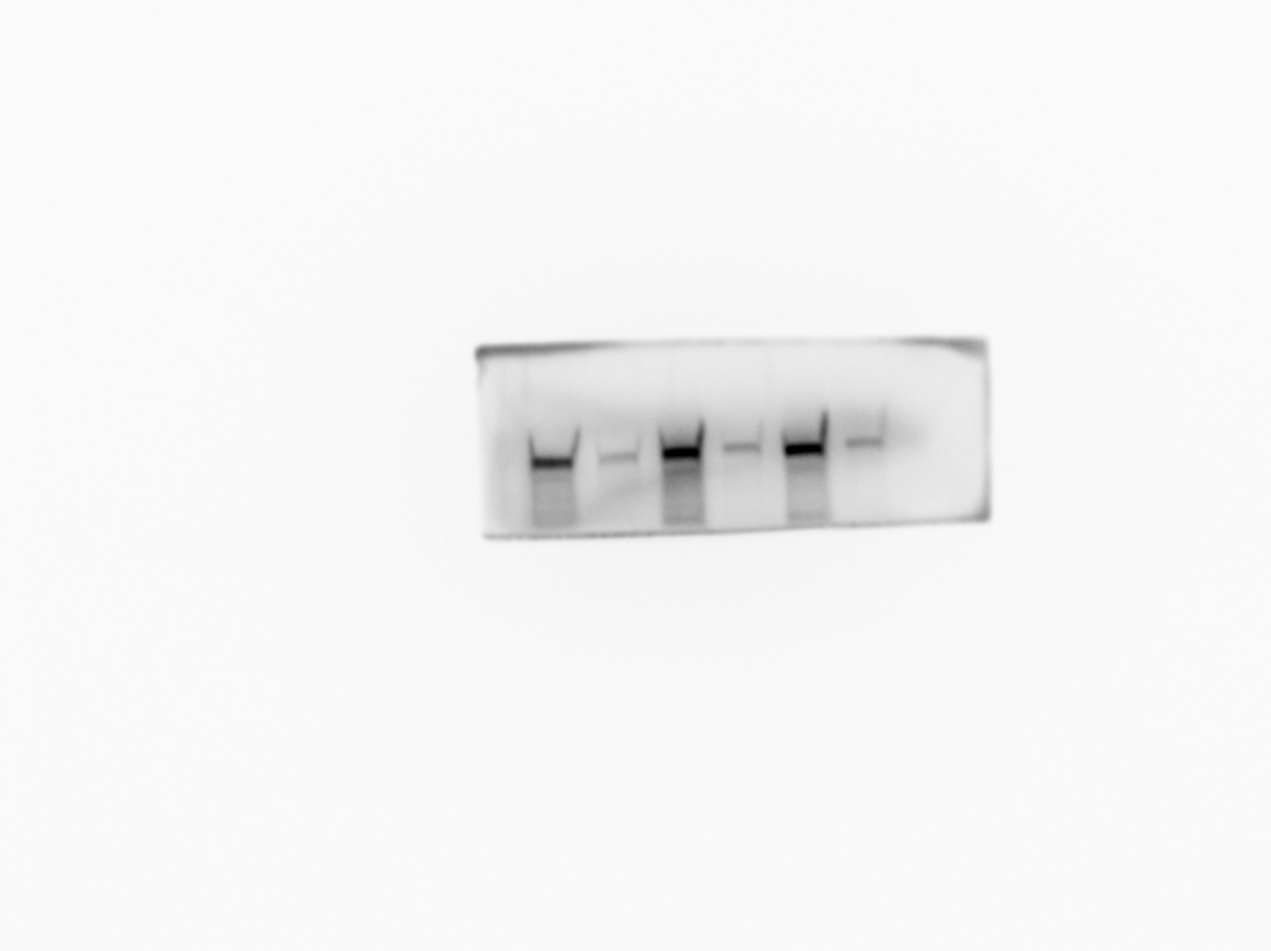


RNA Pol II


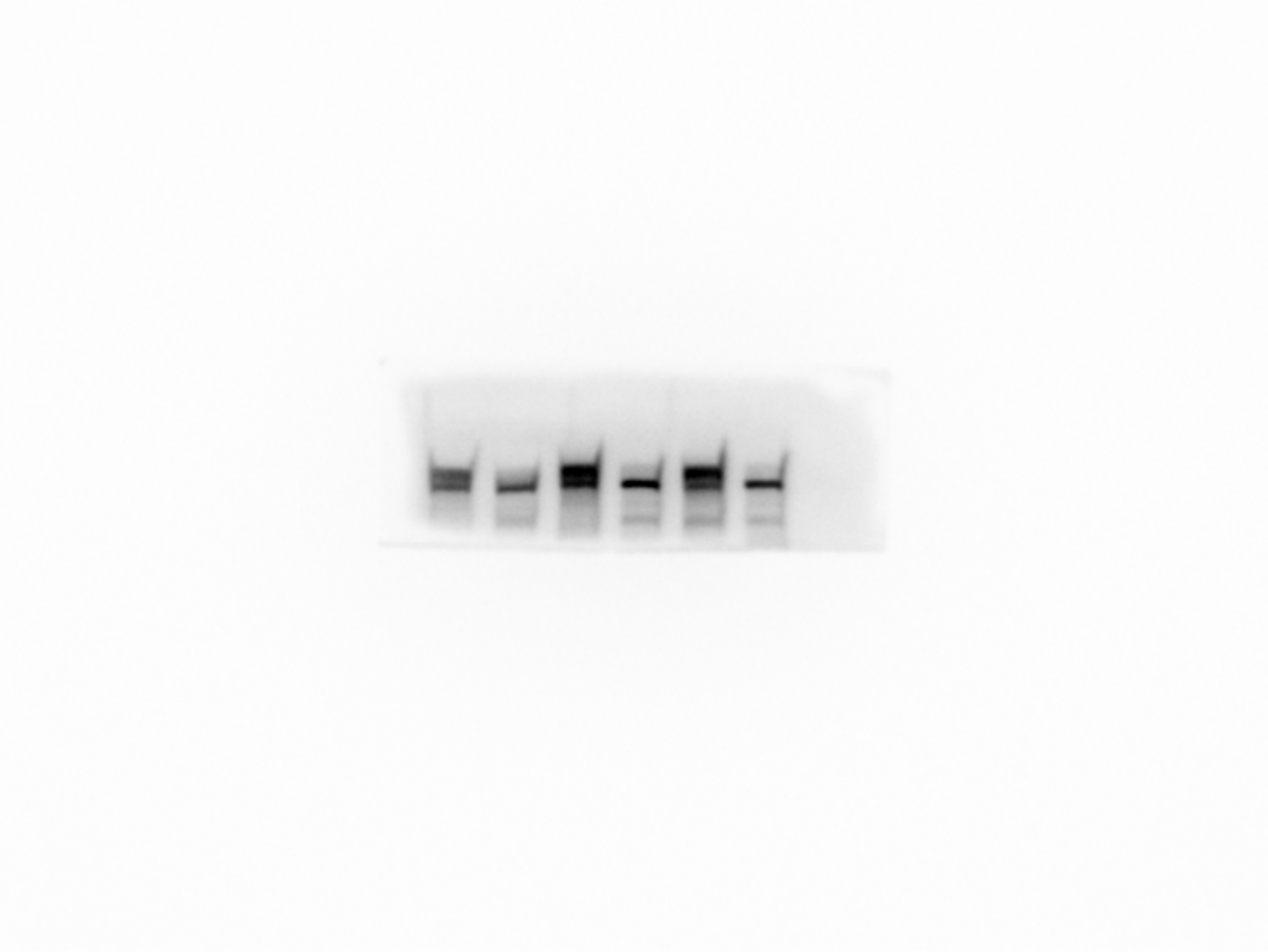


Cleaved-PARP


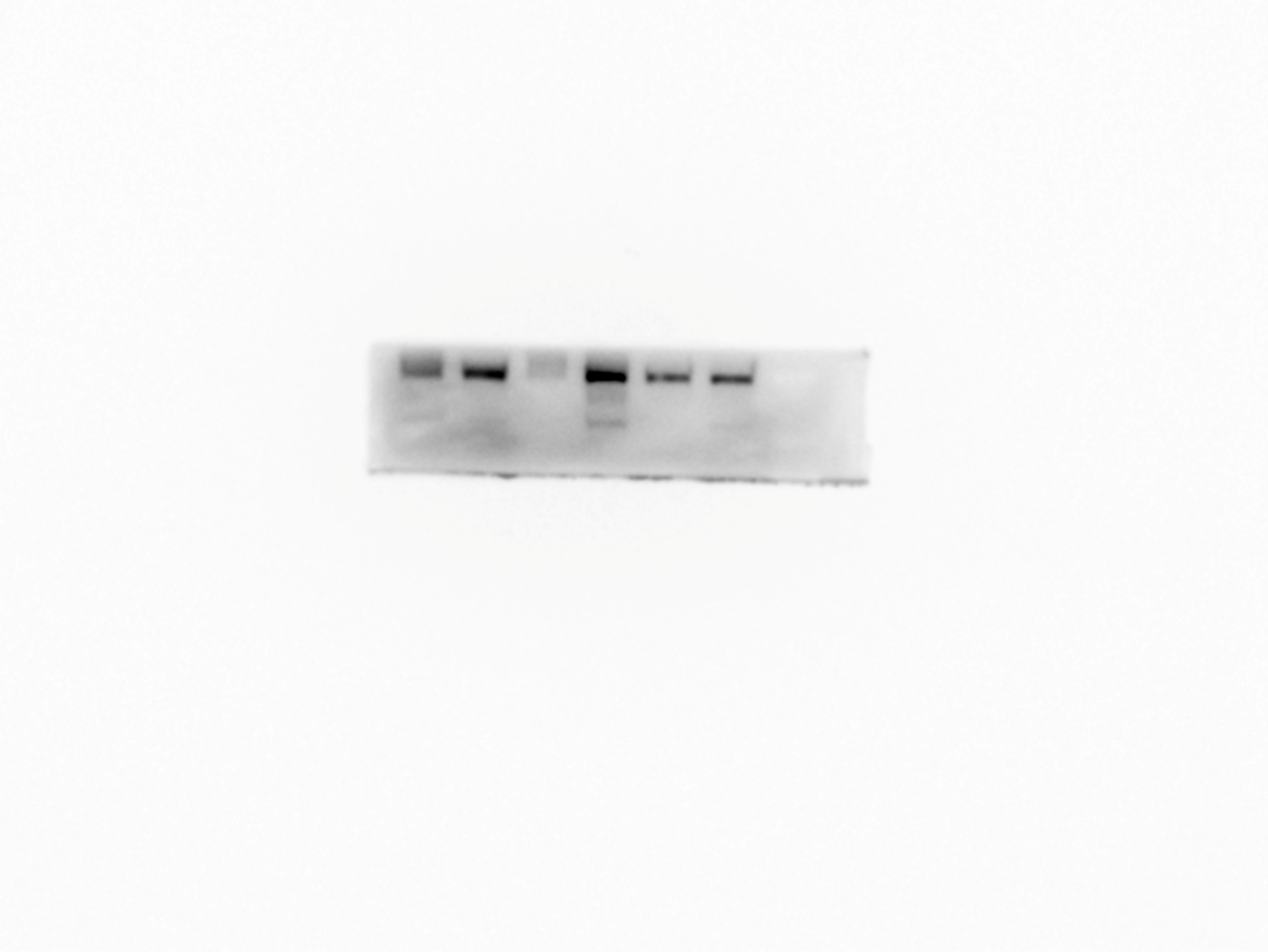


MCL-1


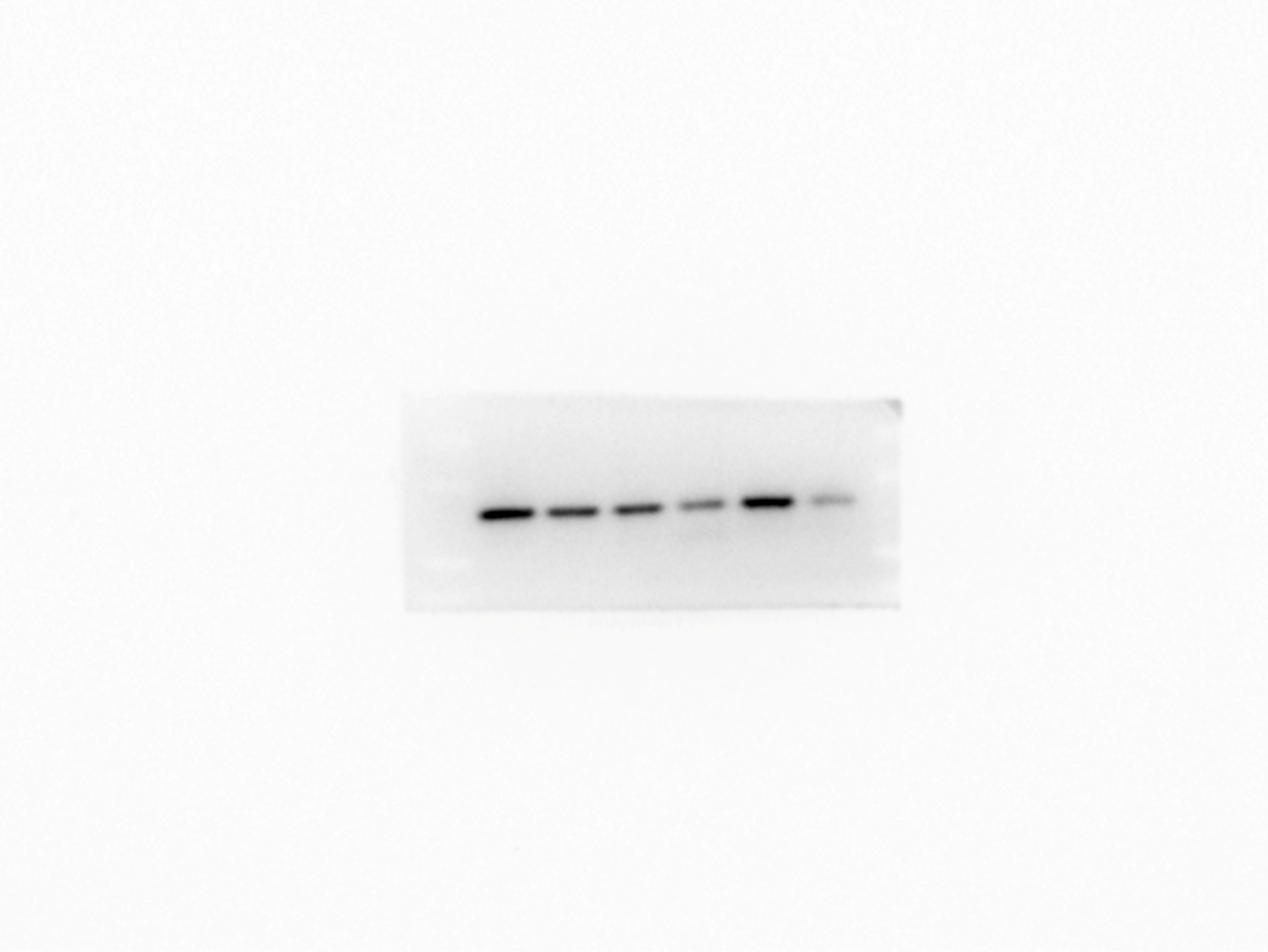


BCL-2


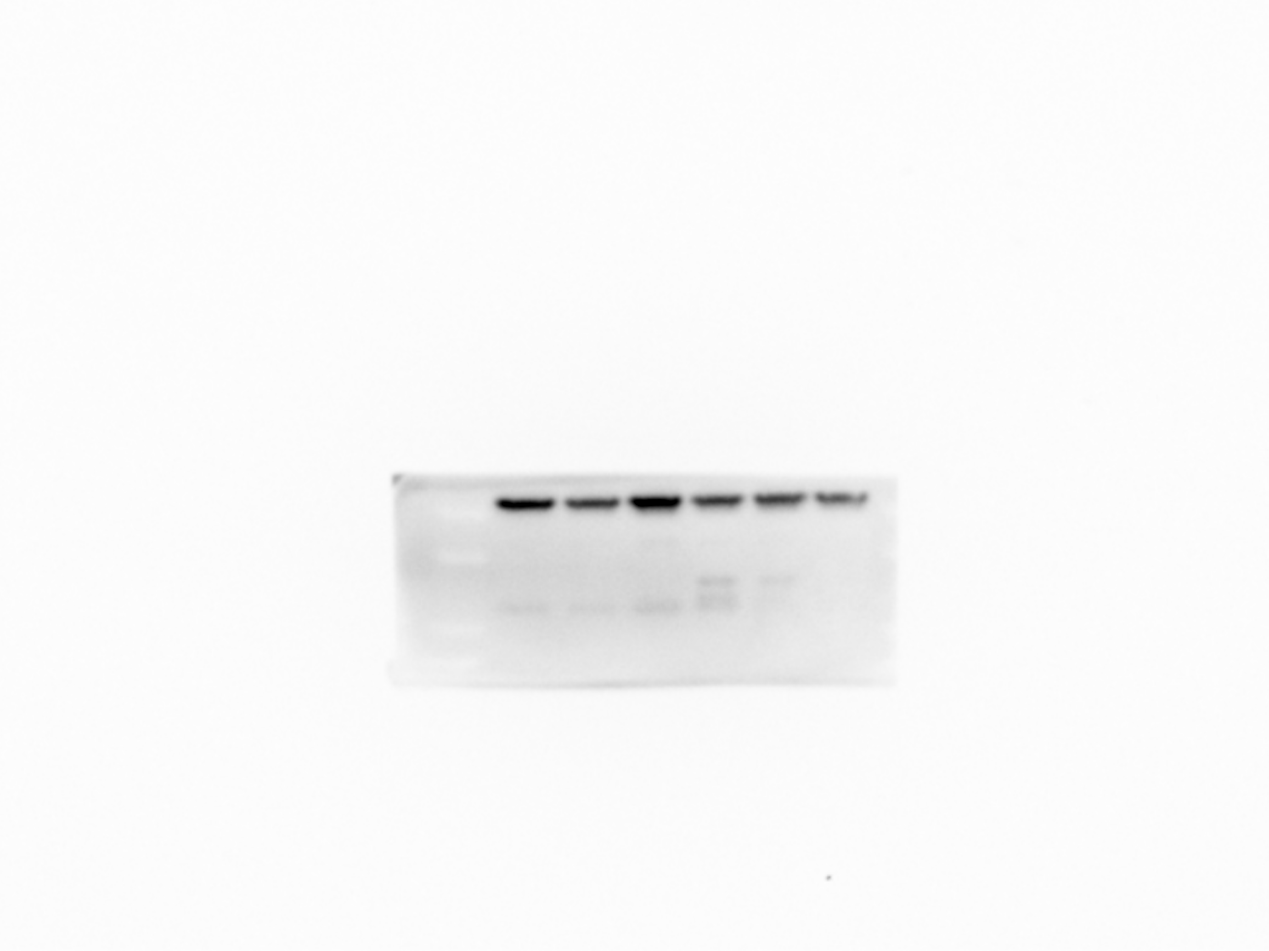


Tubulin


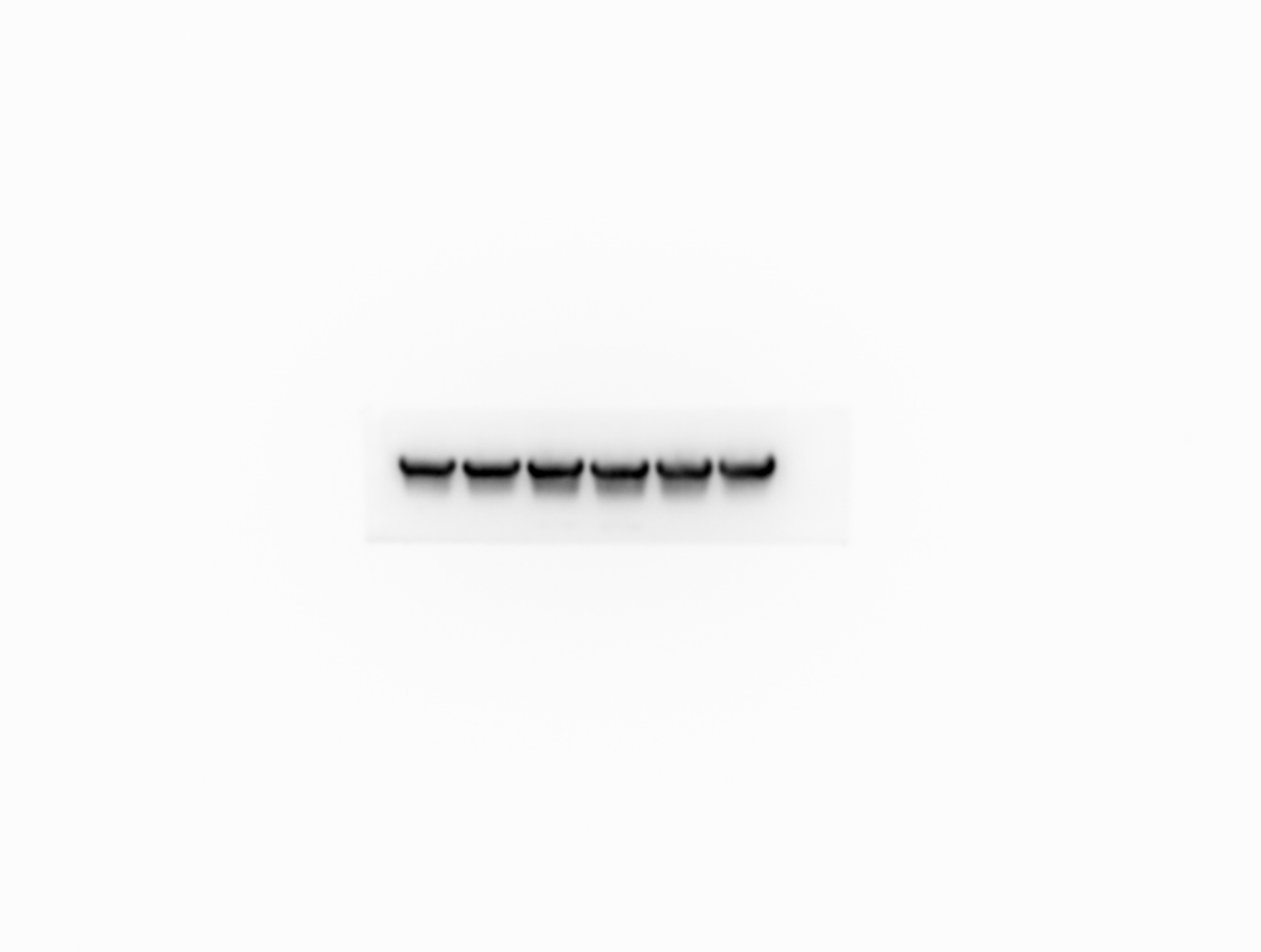


Figure4C

DOK

CDK9


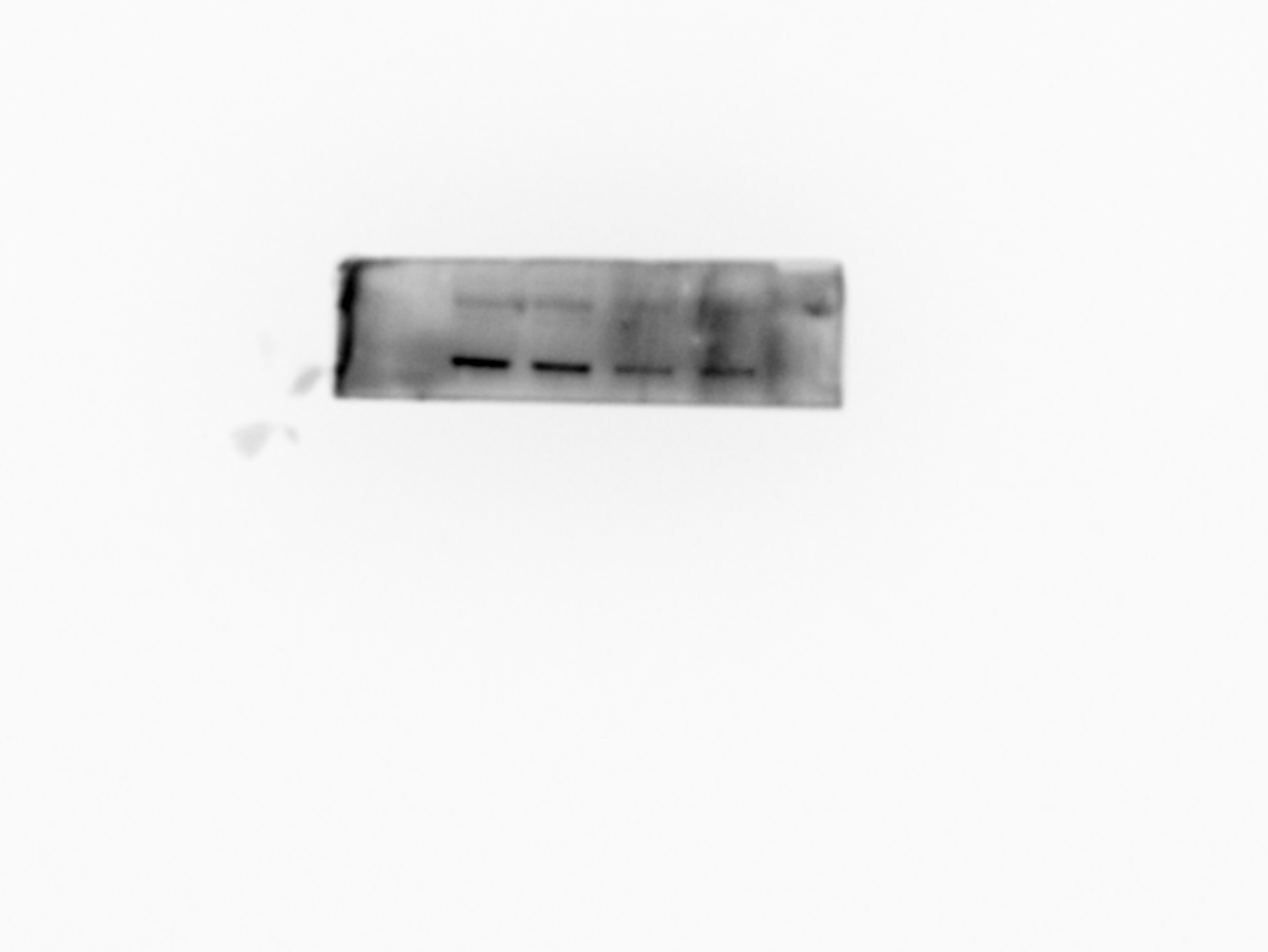


p-RNA Pol II (Ser2)


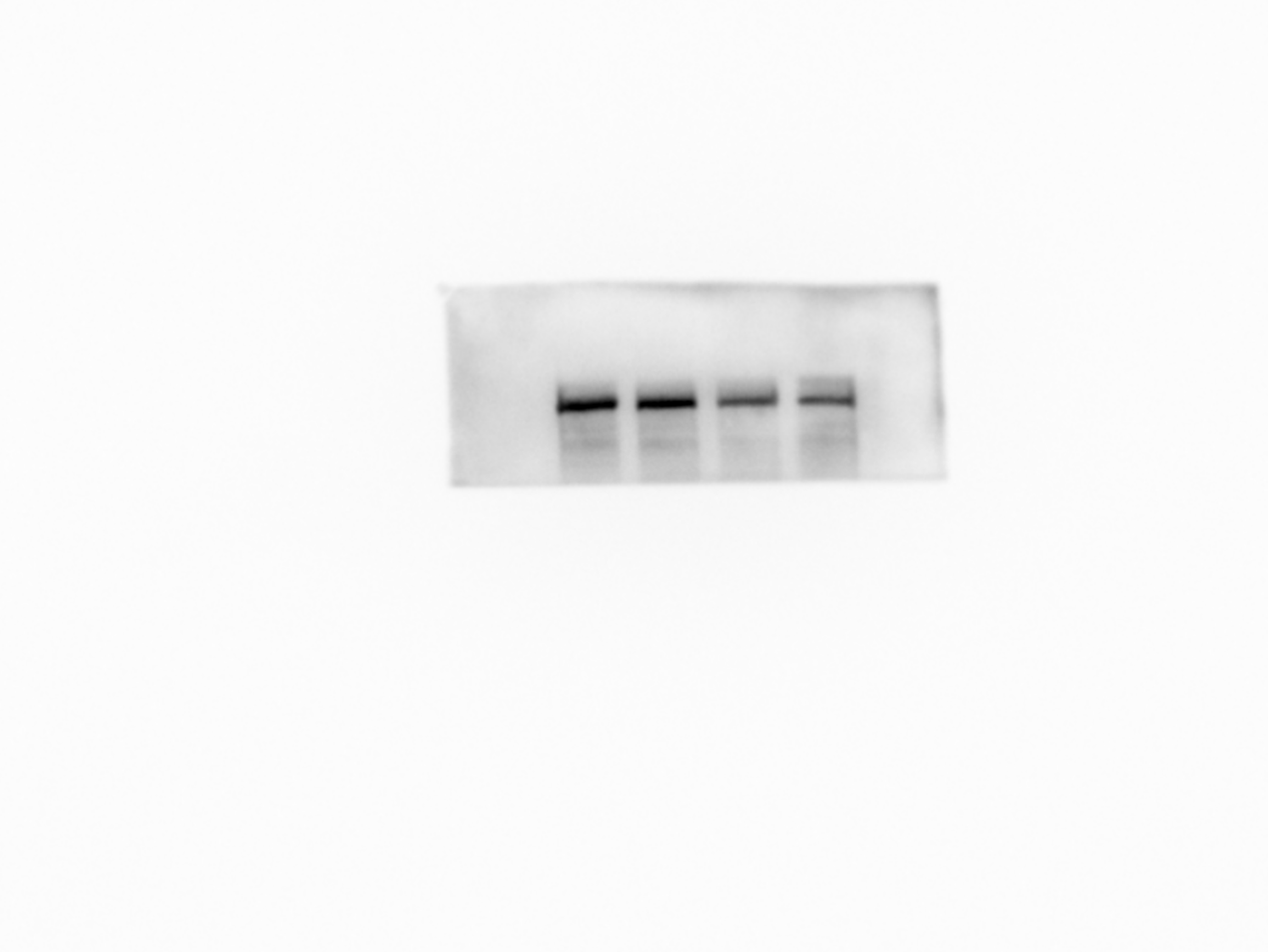


p-RNA Pol II (Thr4)


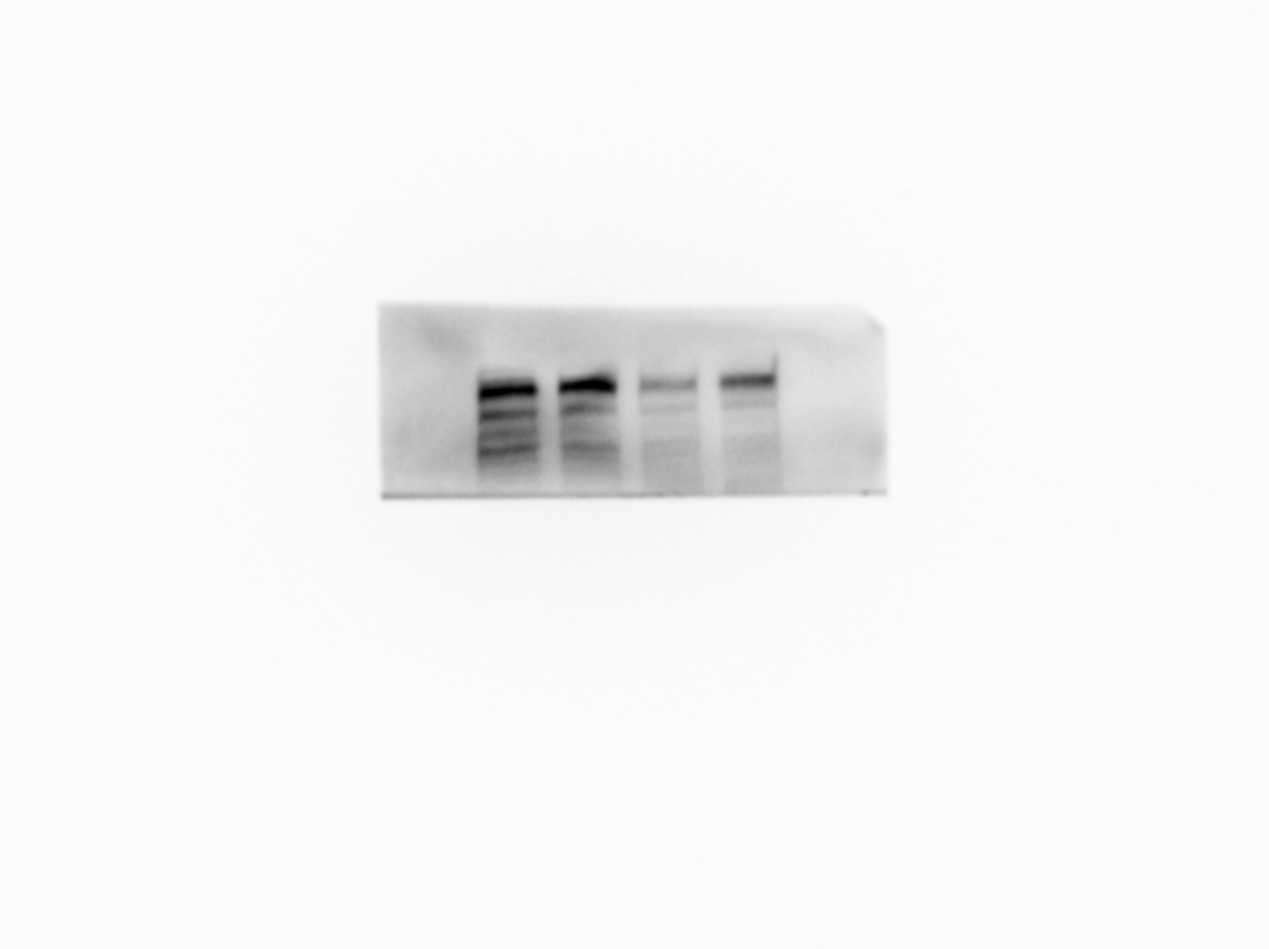


RNA Pol II


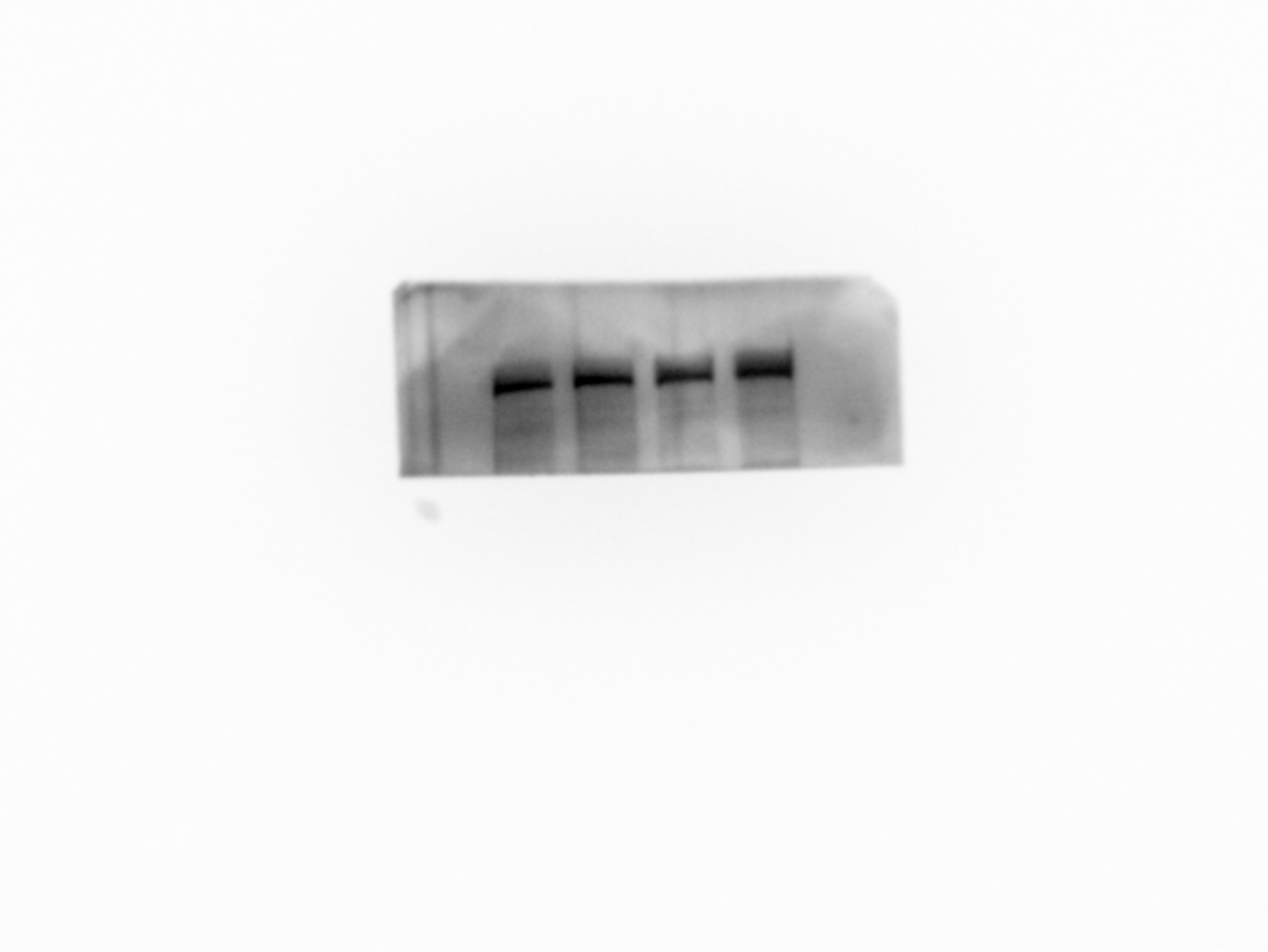


Cleaved-PARP


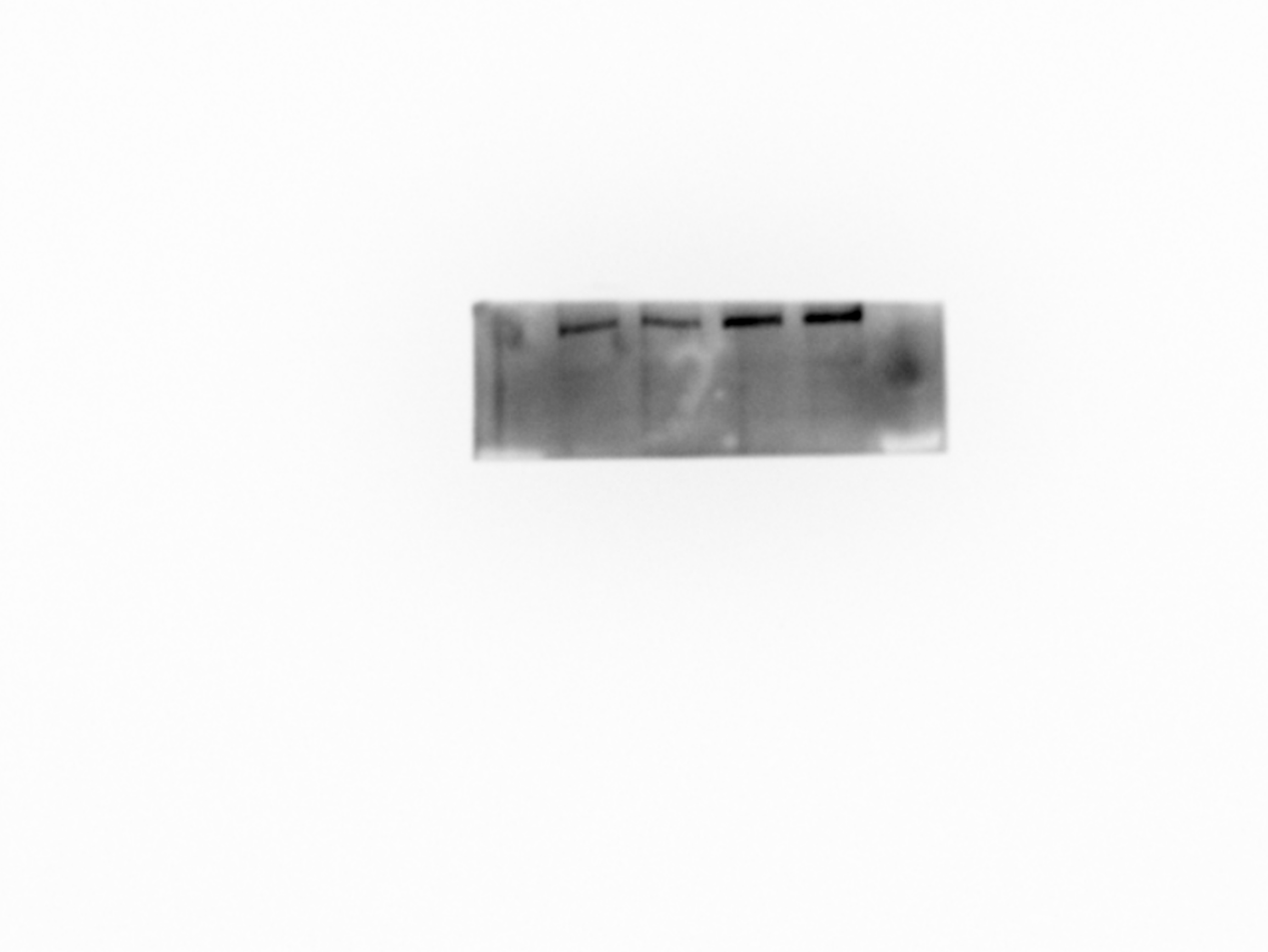


MCL-1


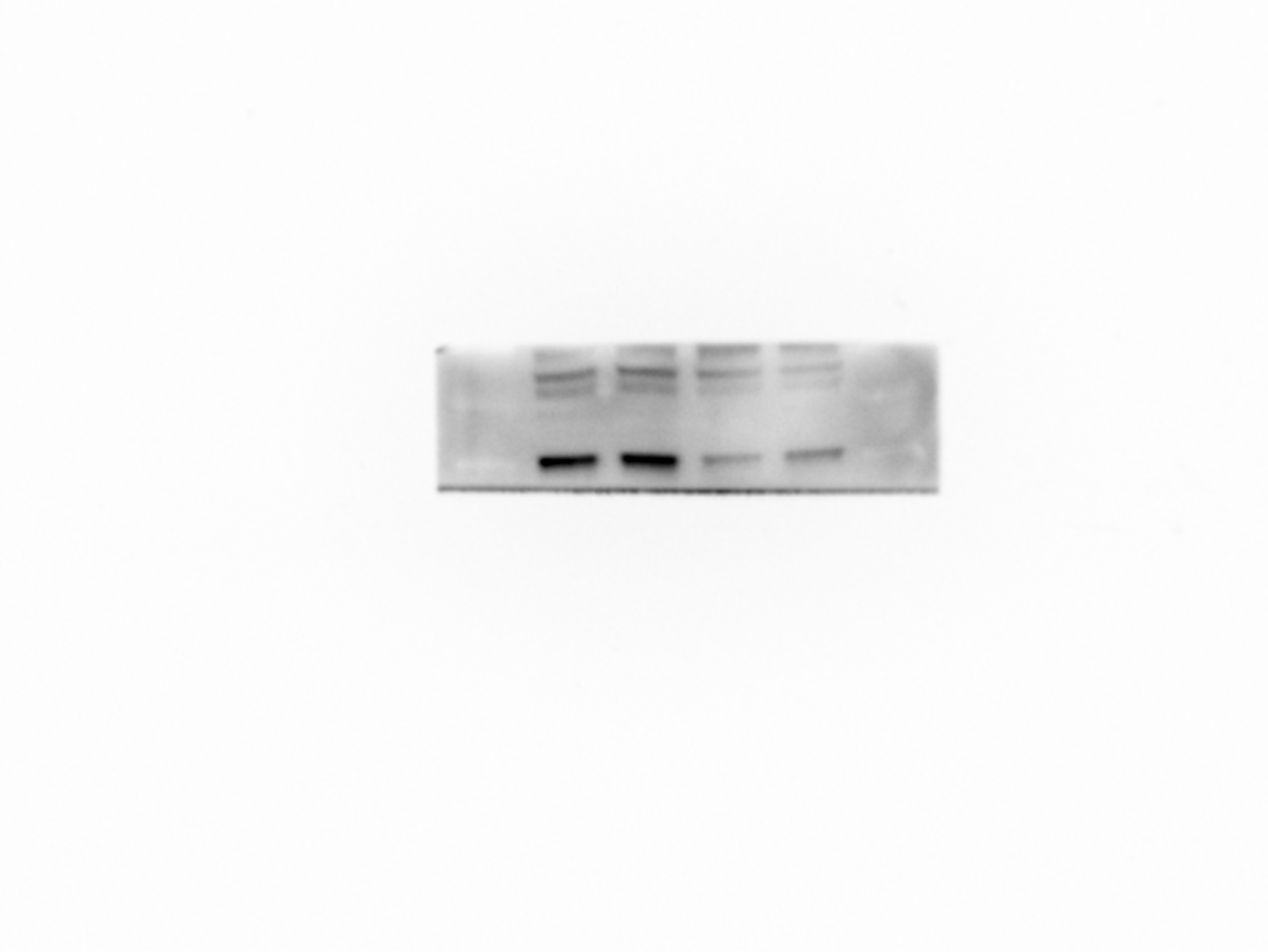


BCL-2


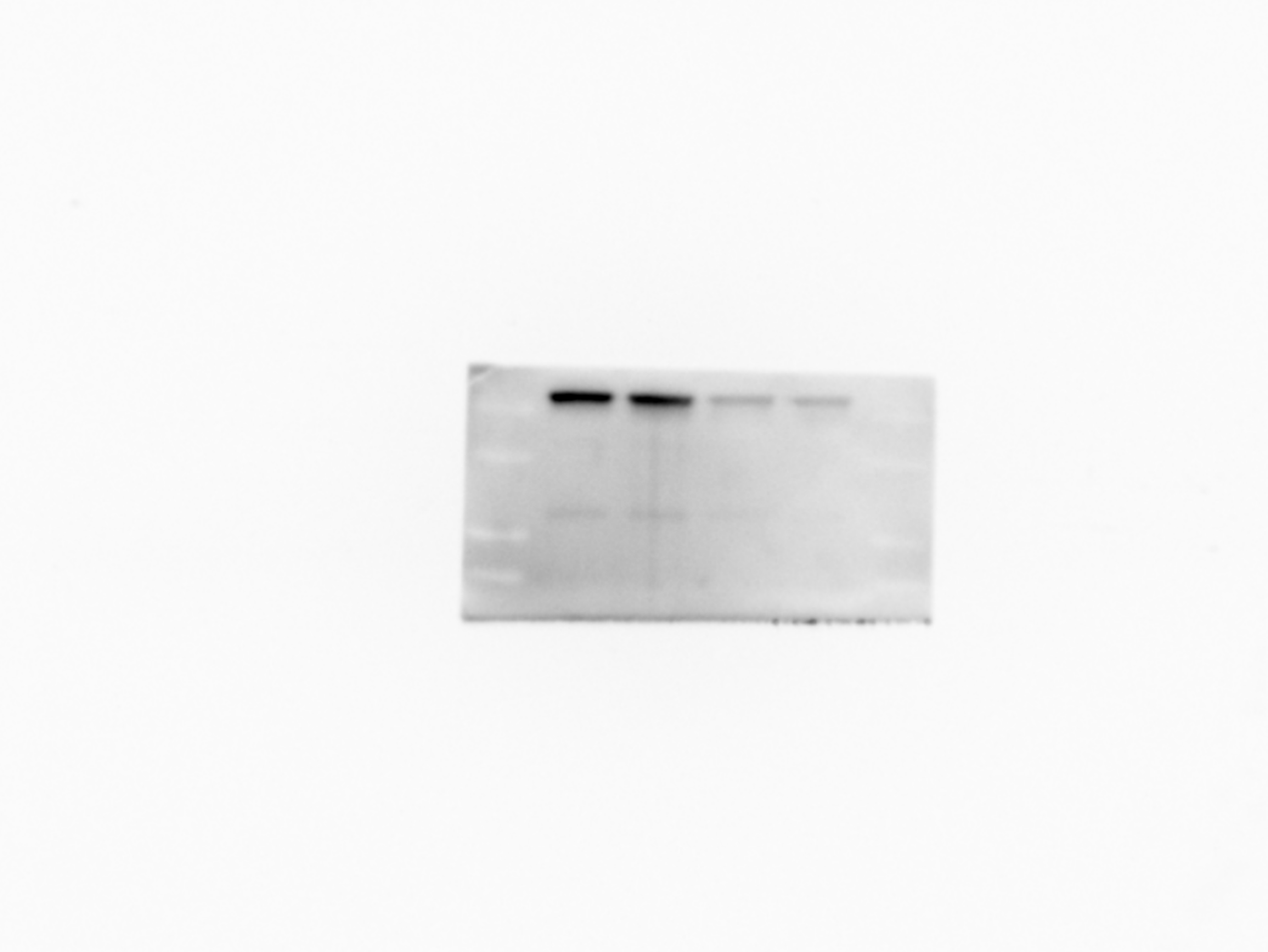


Tubulin


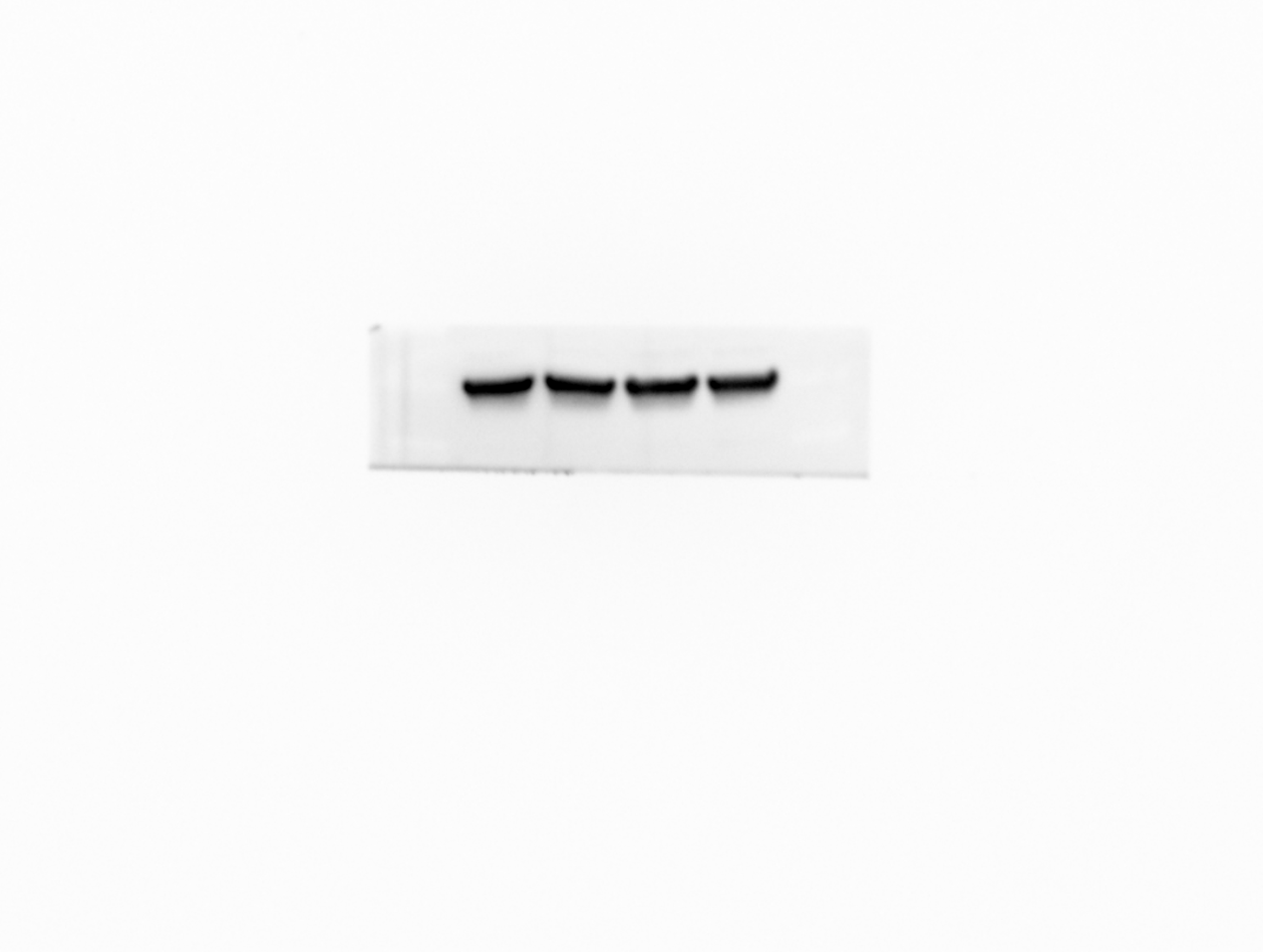


Figure4C

SCC15

CDK9


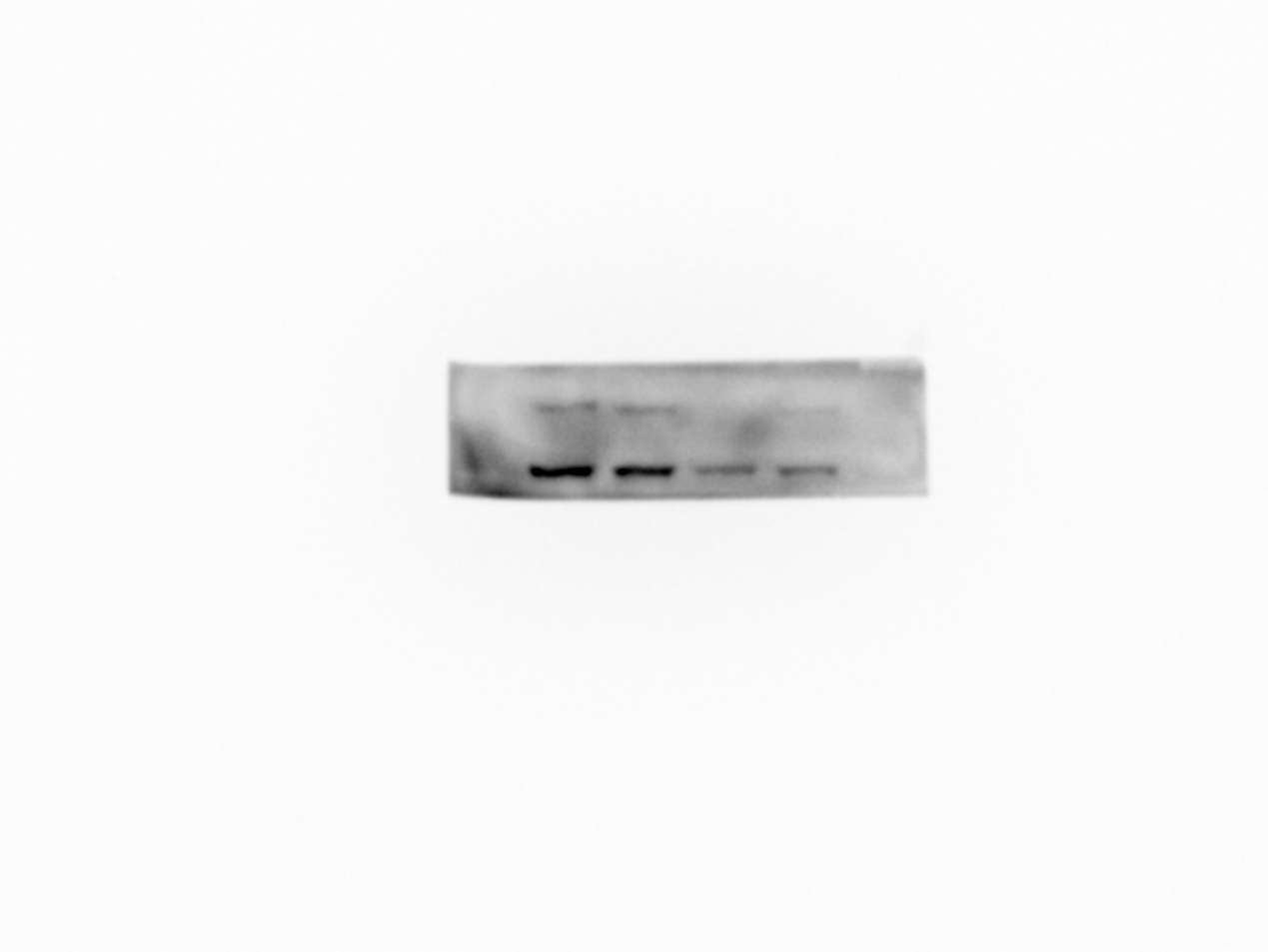


p-RNA Pol II (Ser2)


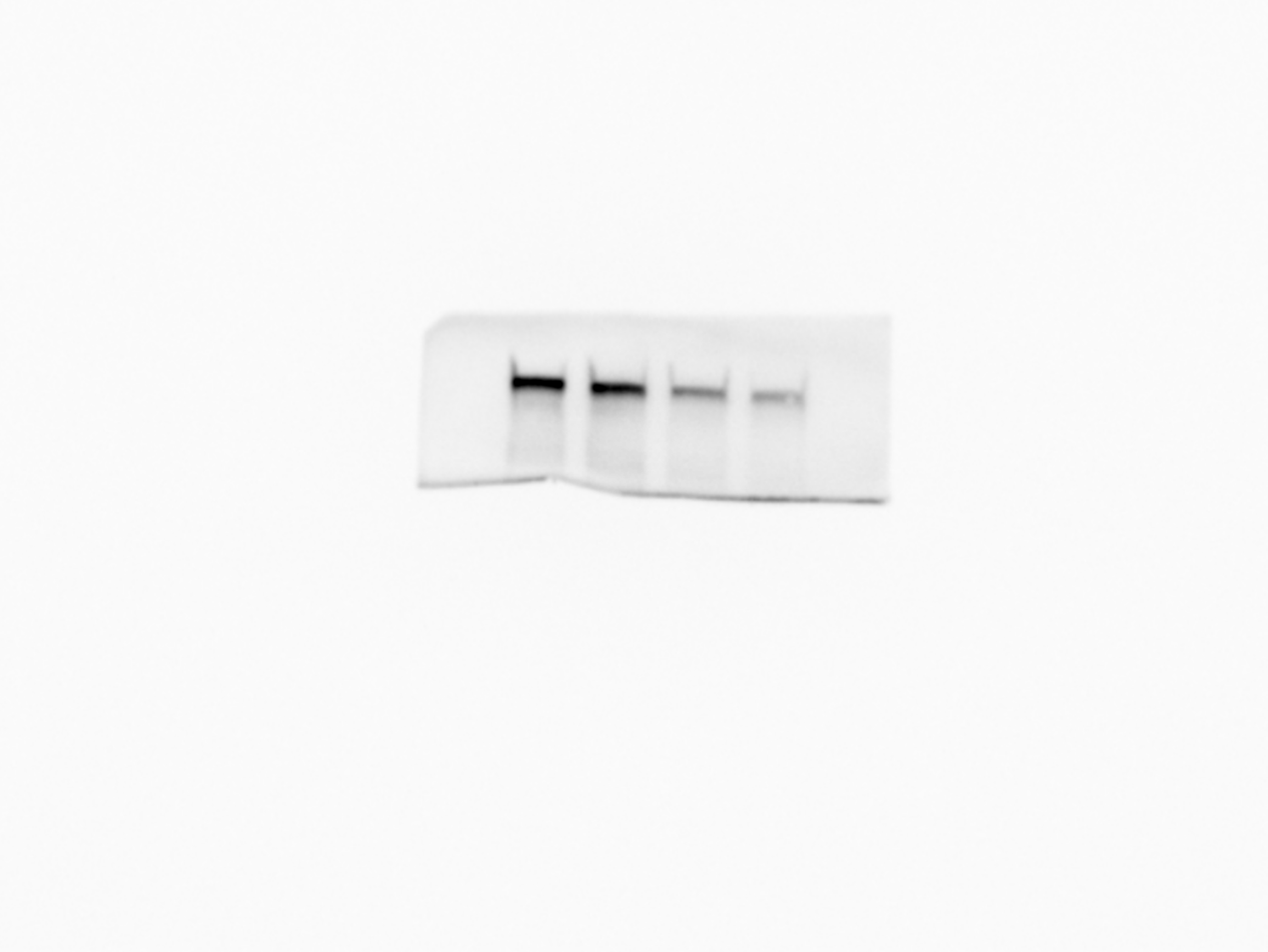


p-RNA Pol II (Thr4)


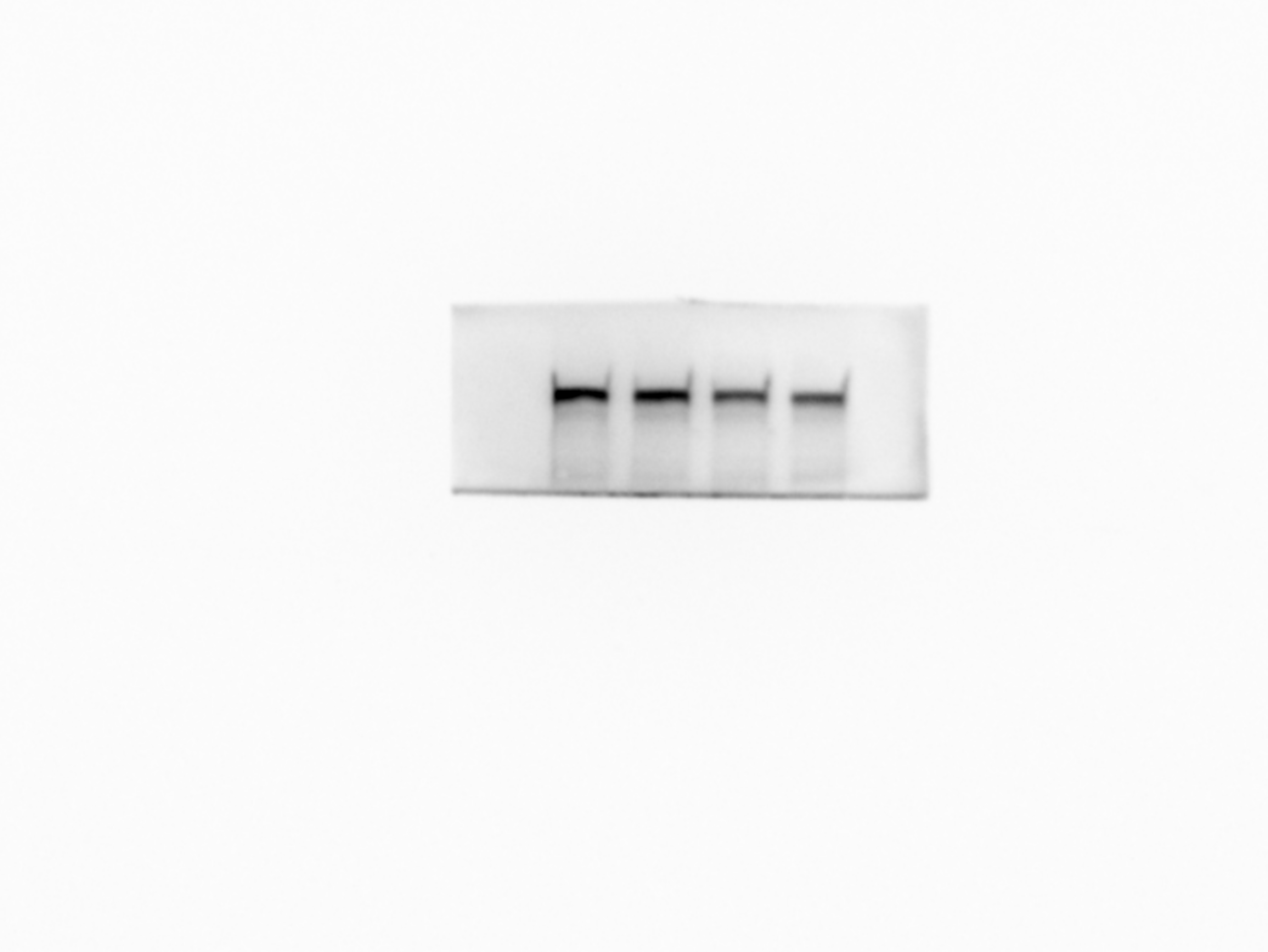


RNA Pol II


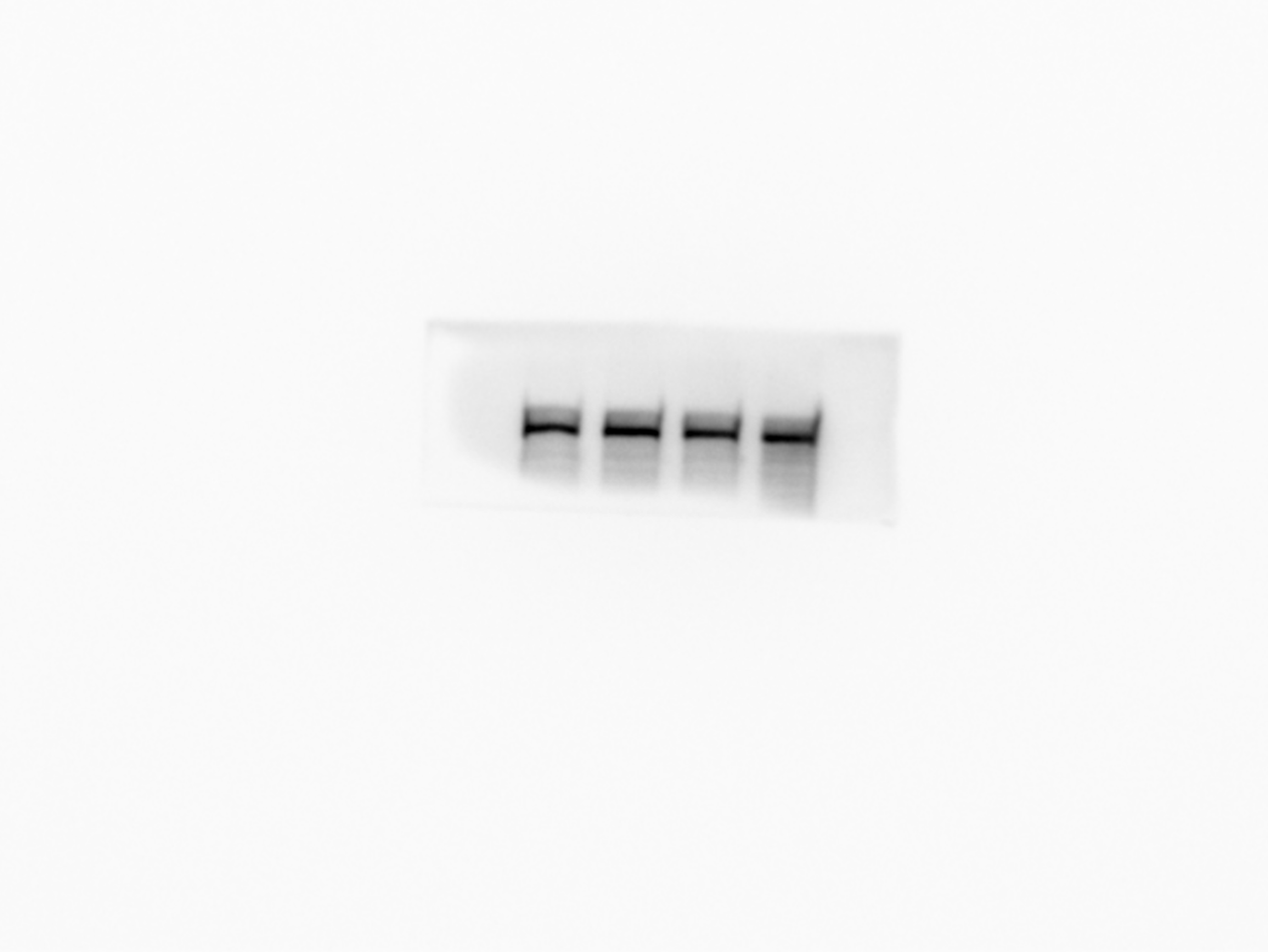


Cleaved-PARP


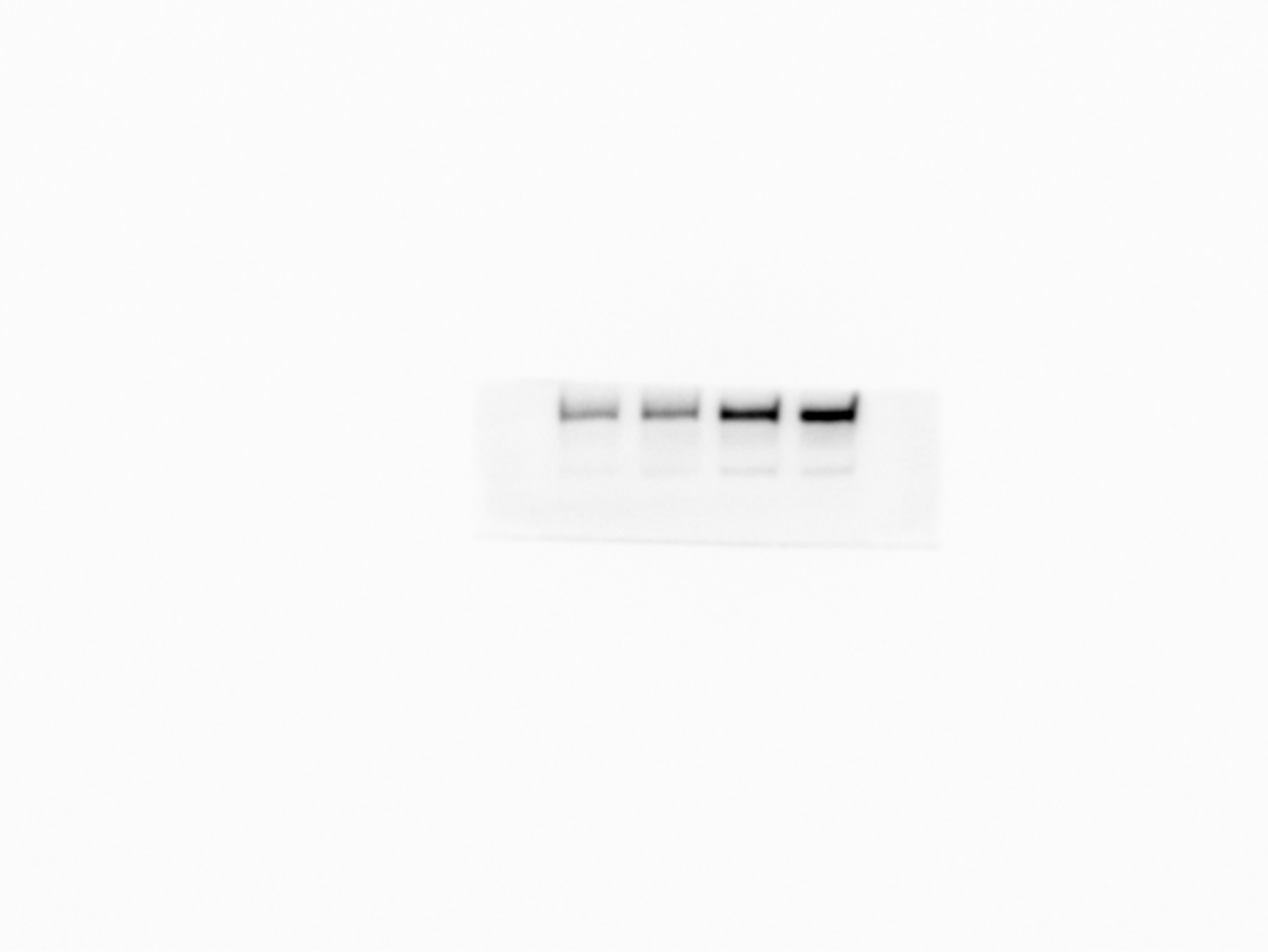


MCL-1


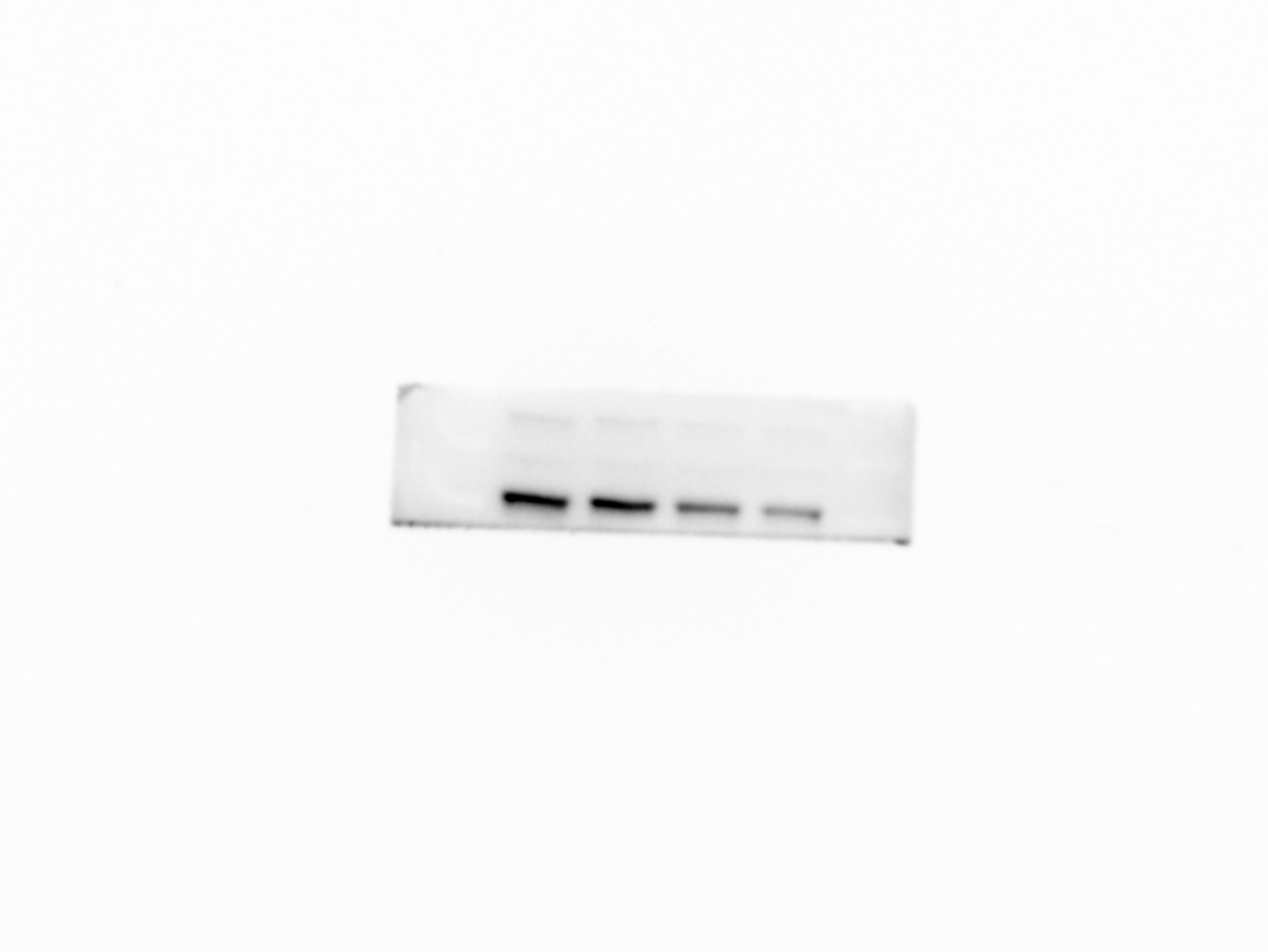


BCL-2


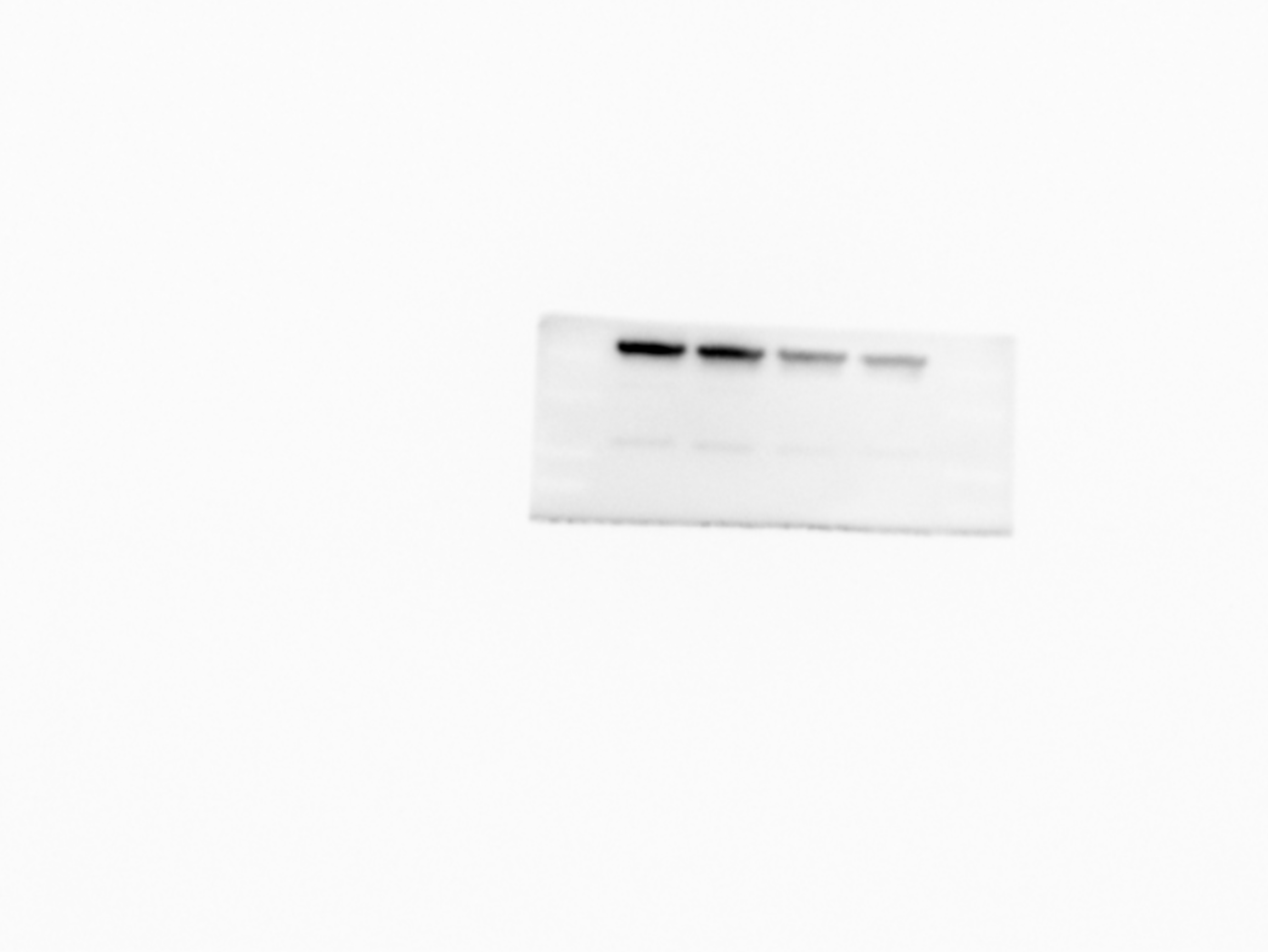


Tubulin


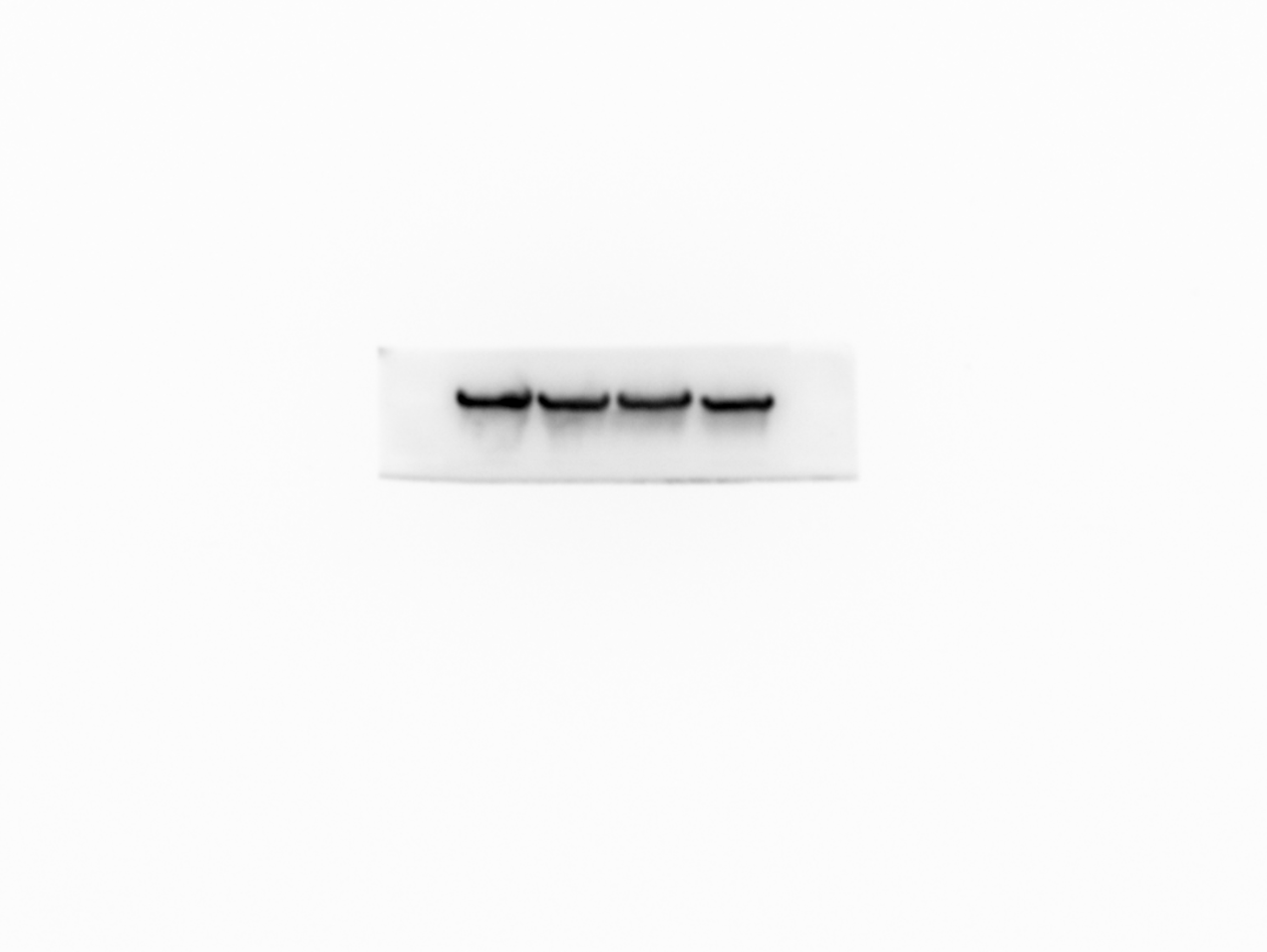


Figure4C

HSC3

CDK9


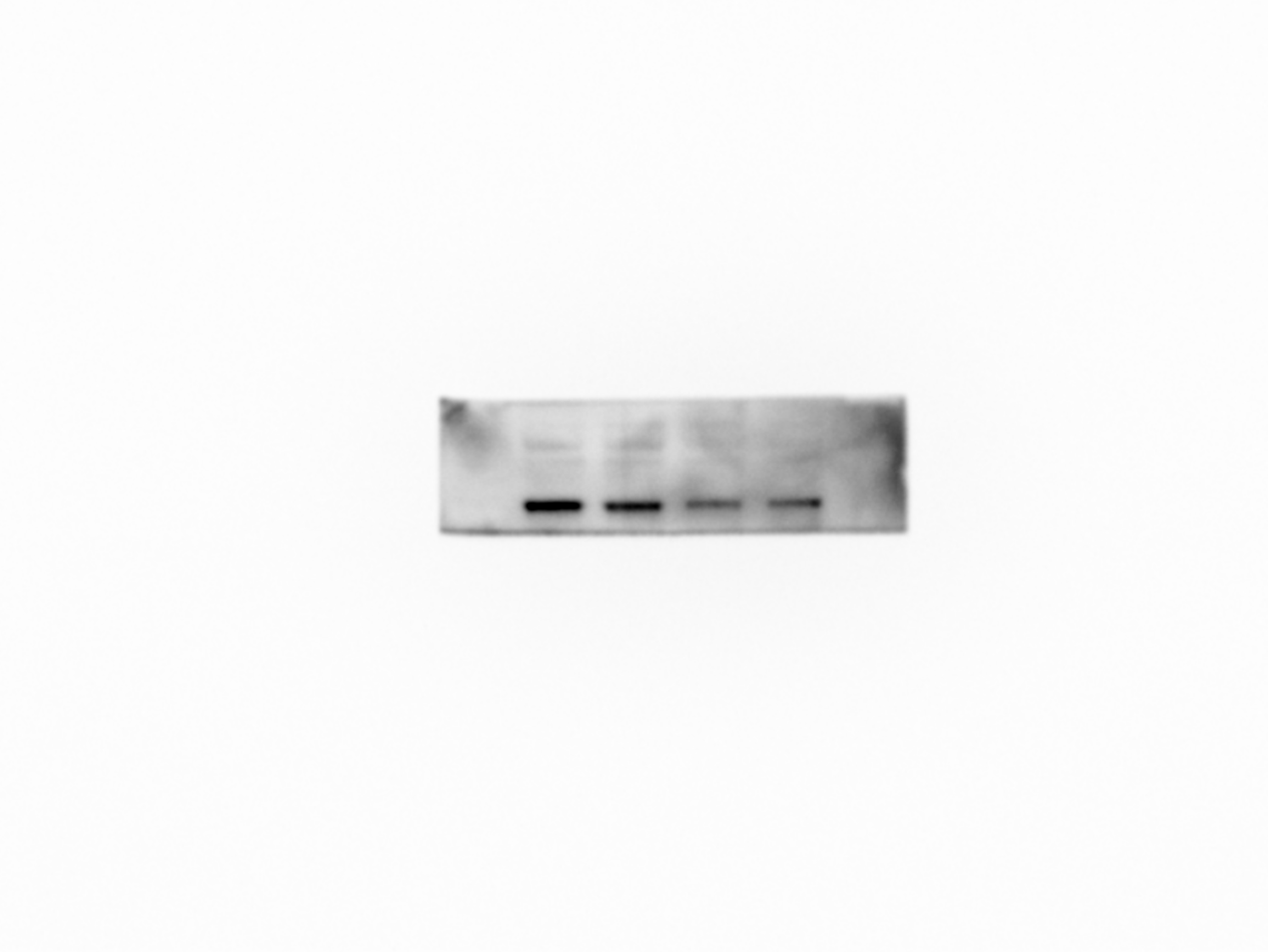


p-RNA Pol II (Ser2)


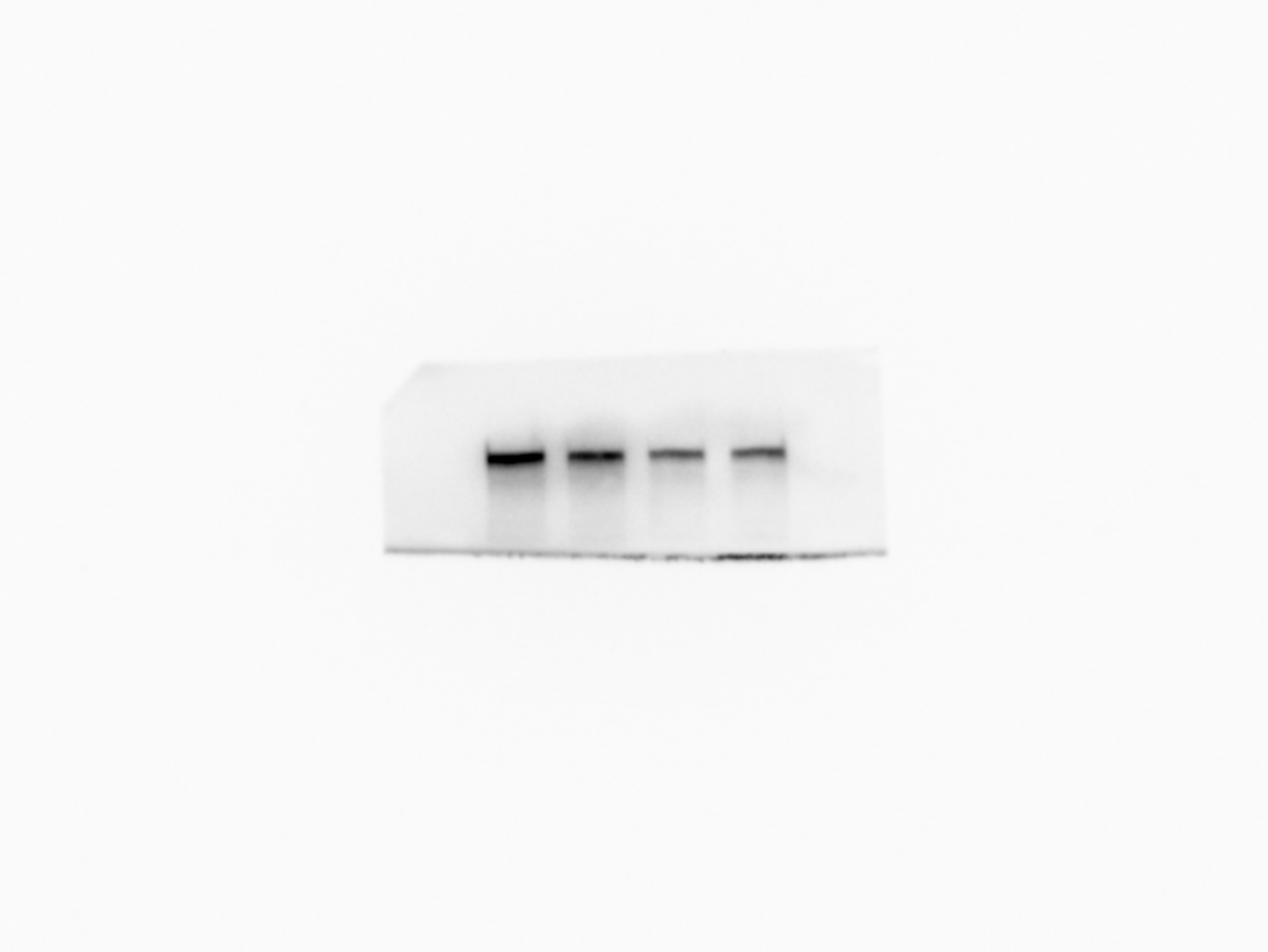


p-RNA Pol II (Thr4)


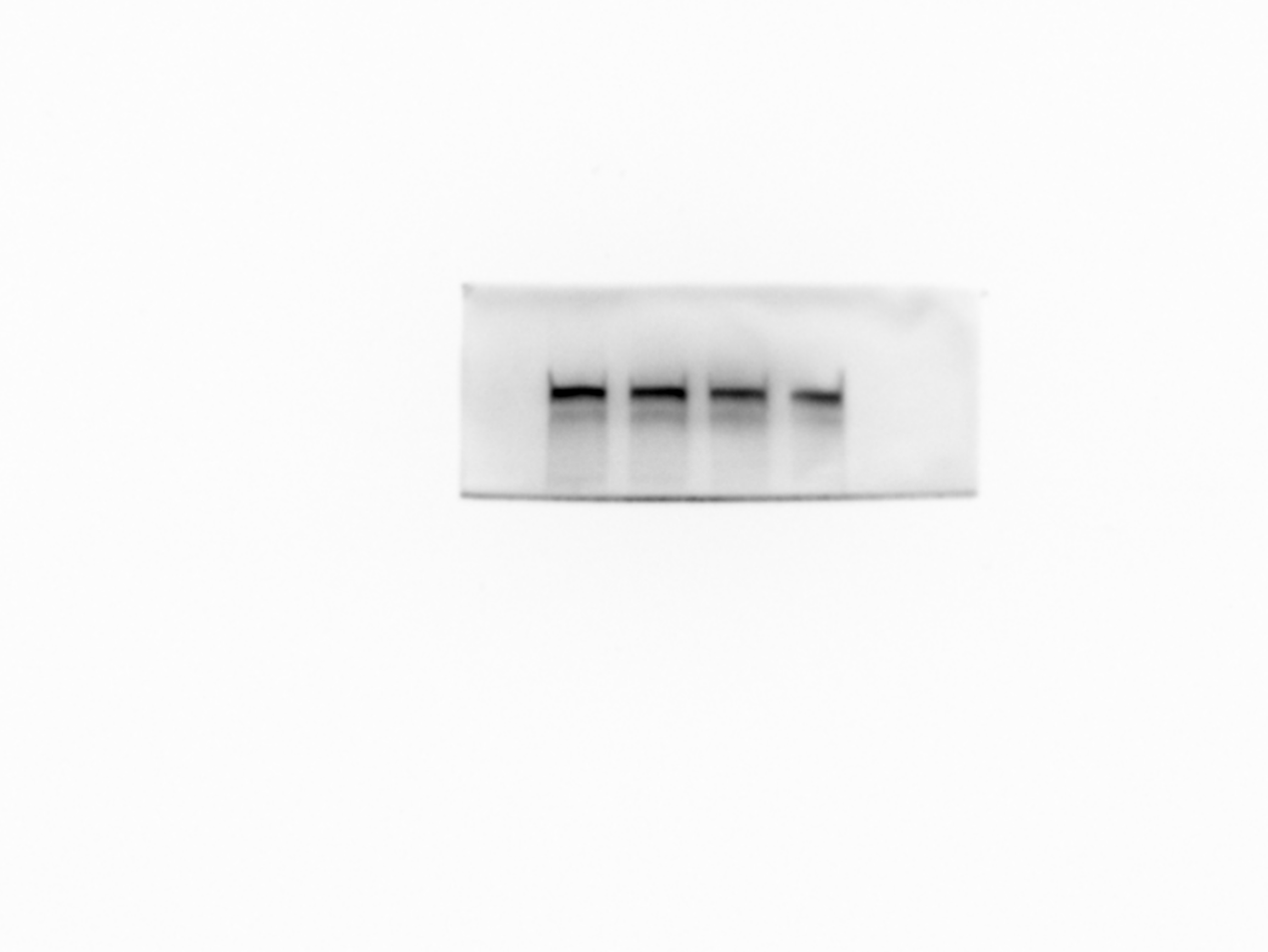


RNA Pol II


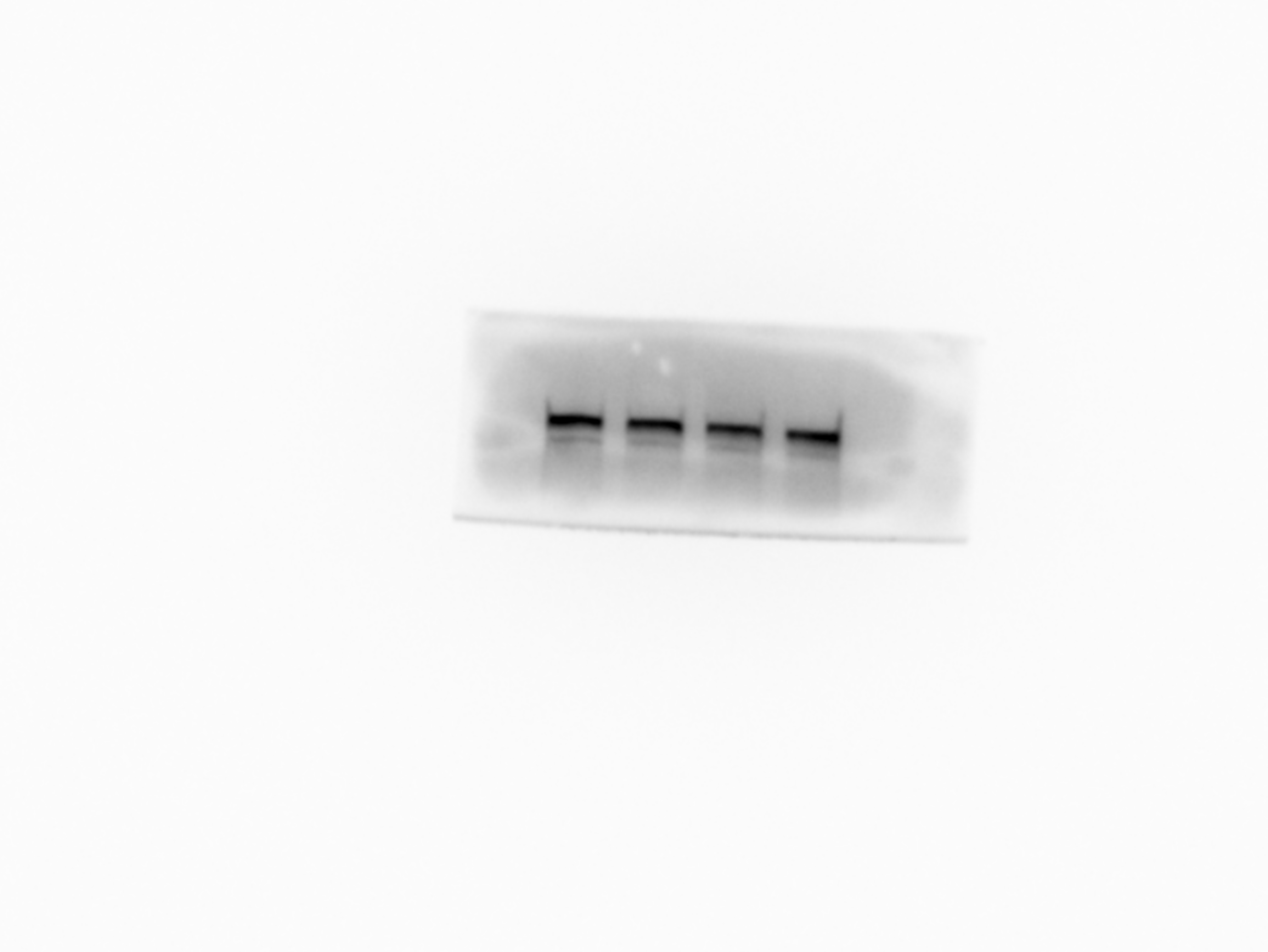


Cleaved-PARP


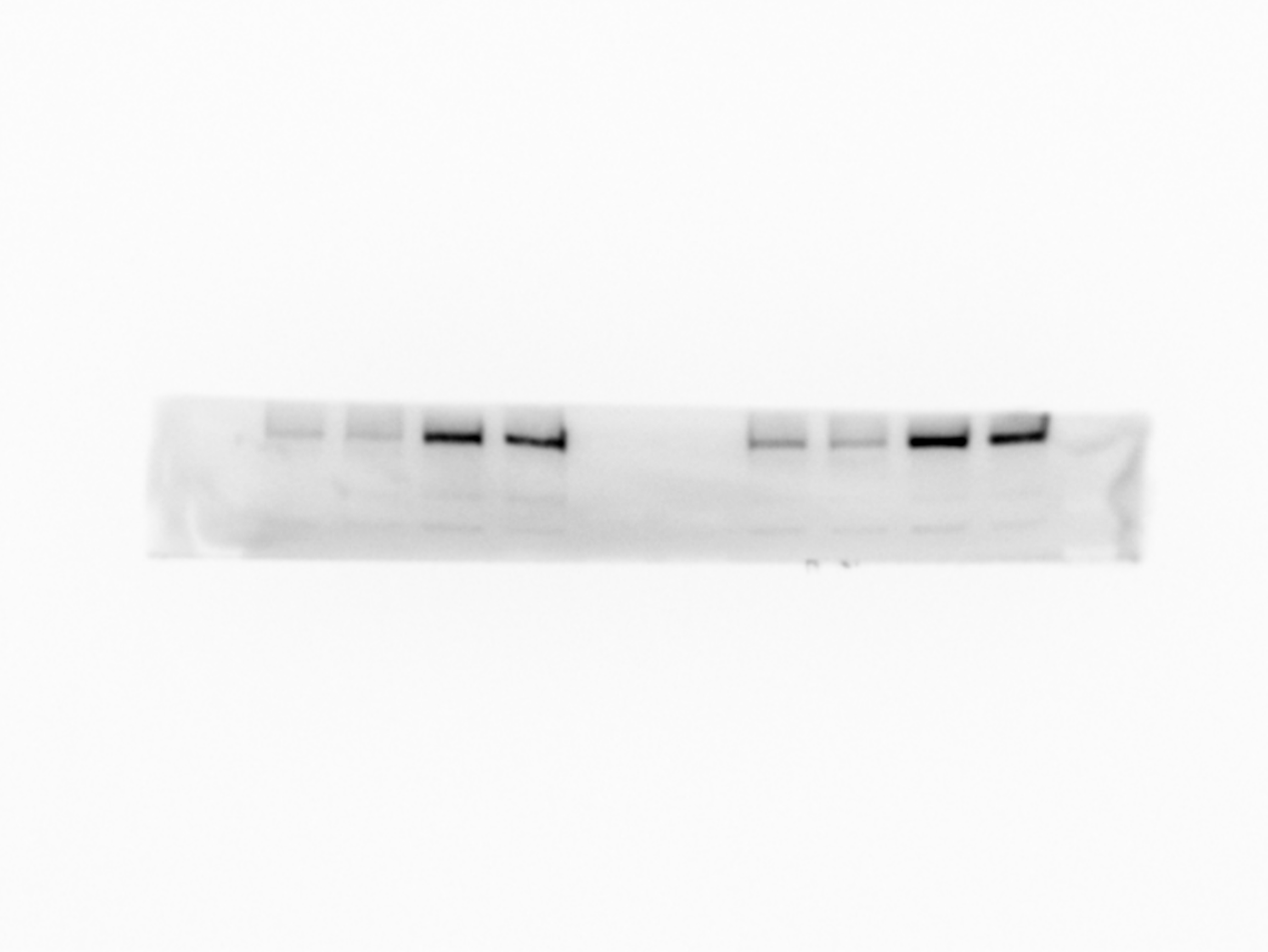


MCL-1


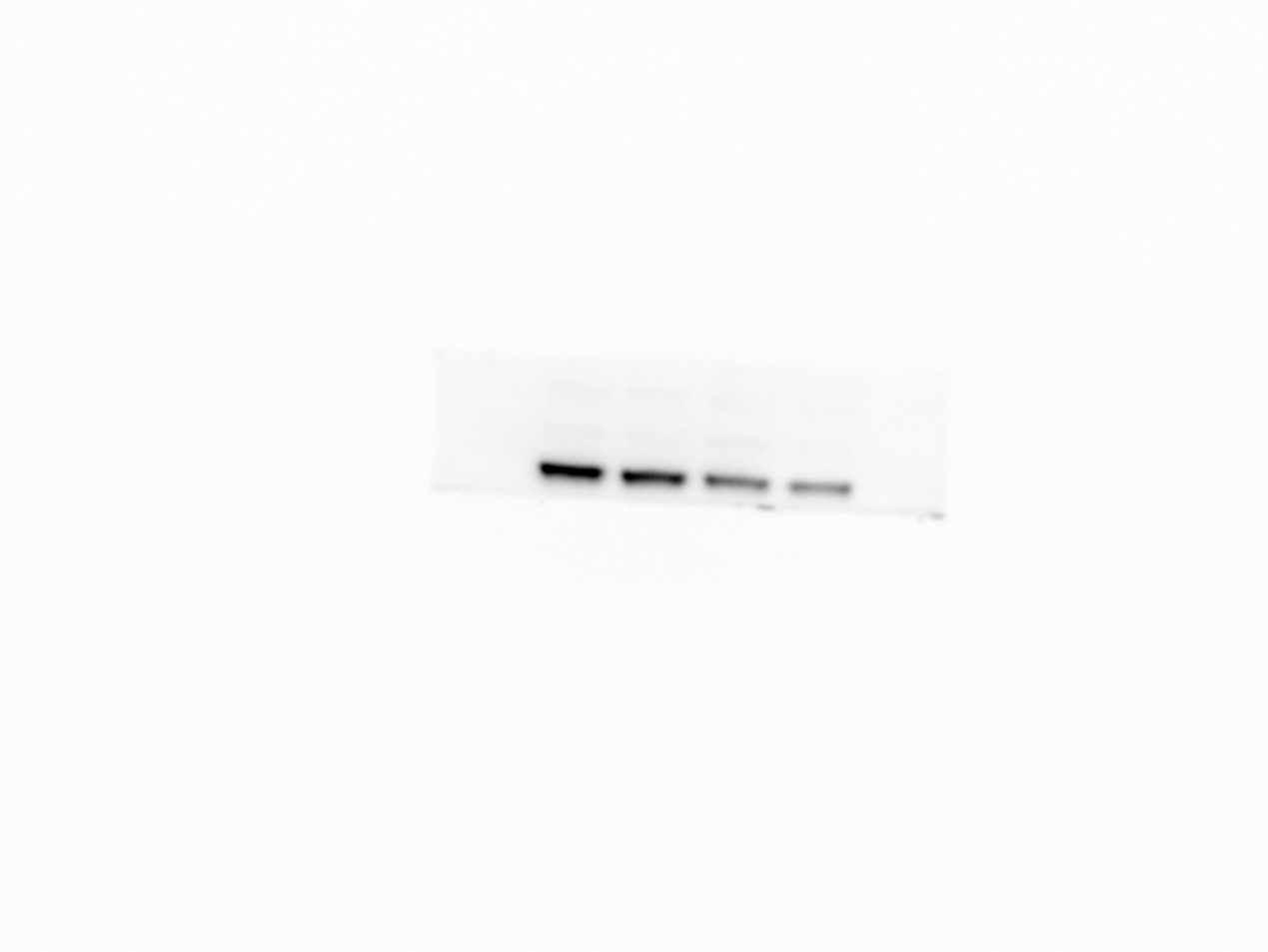


BCL-2


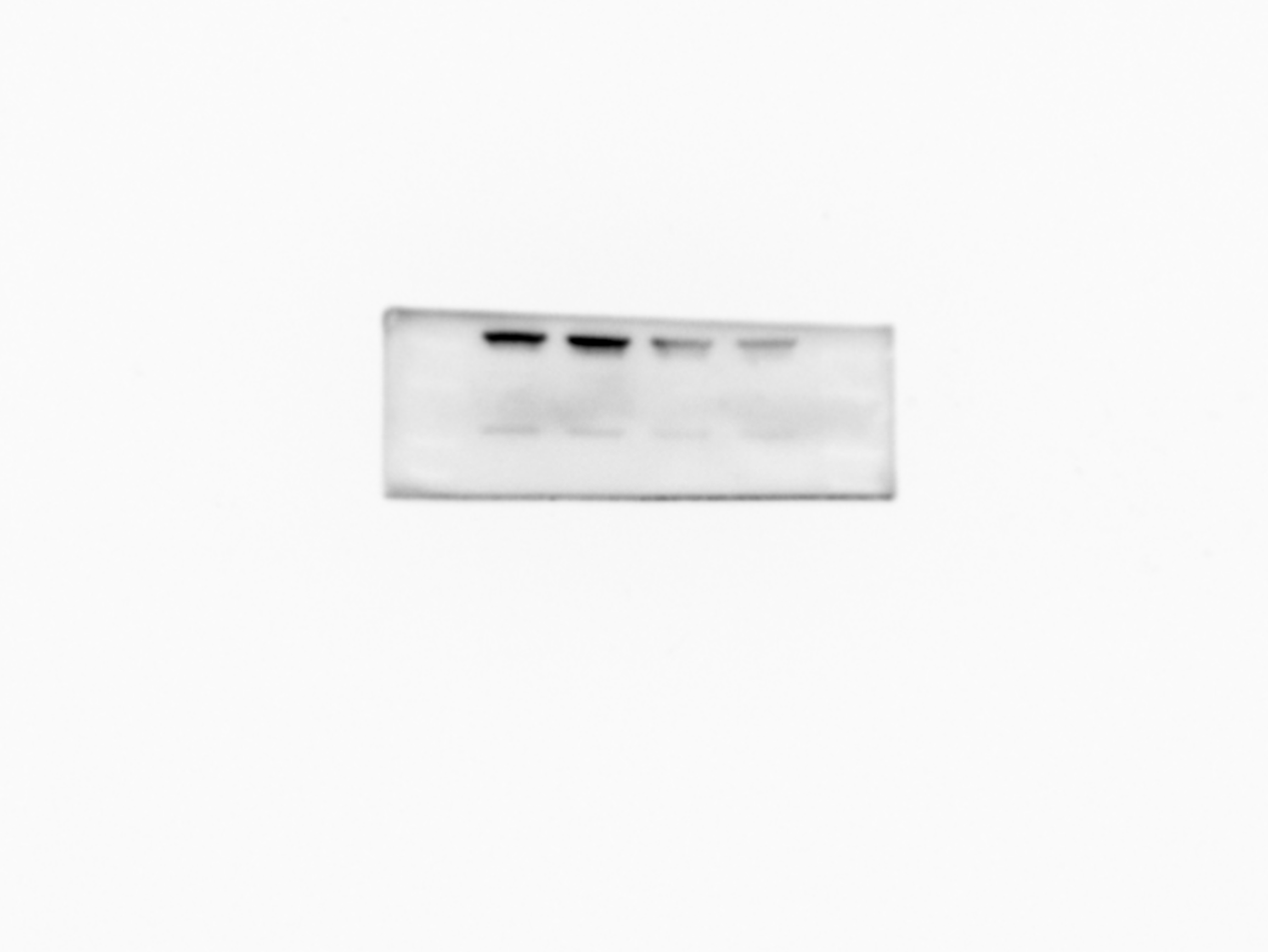


Tubulin


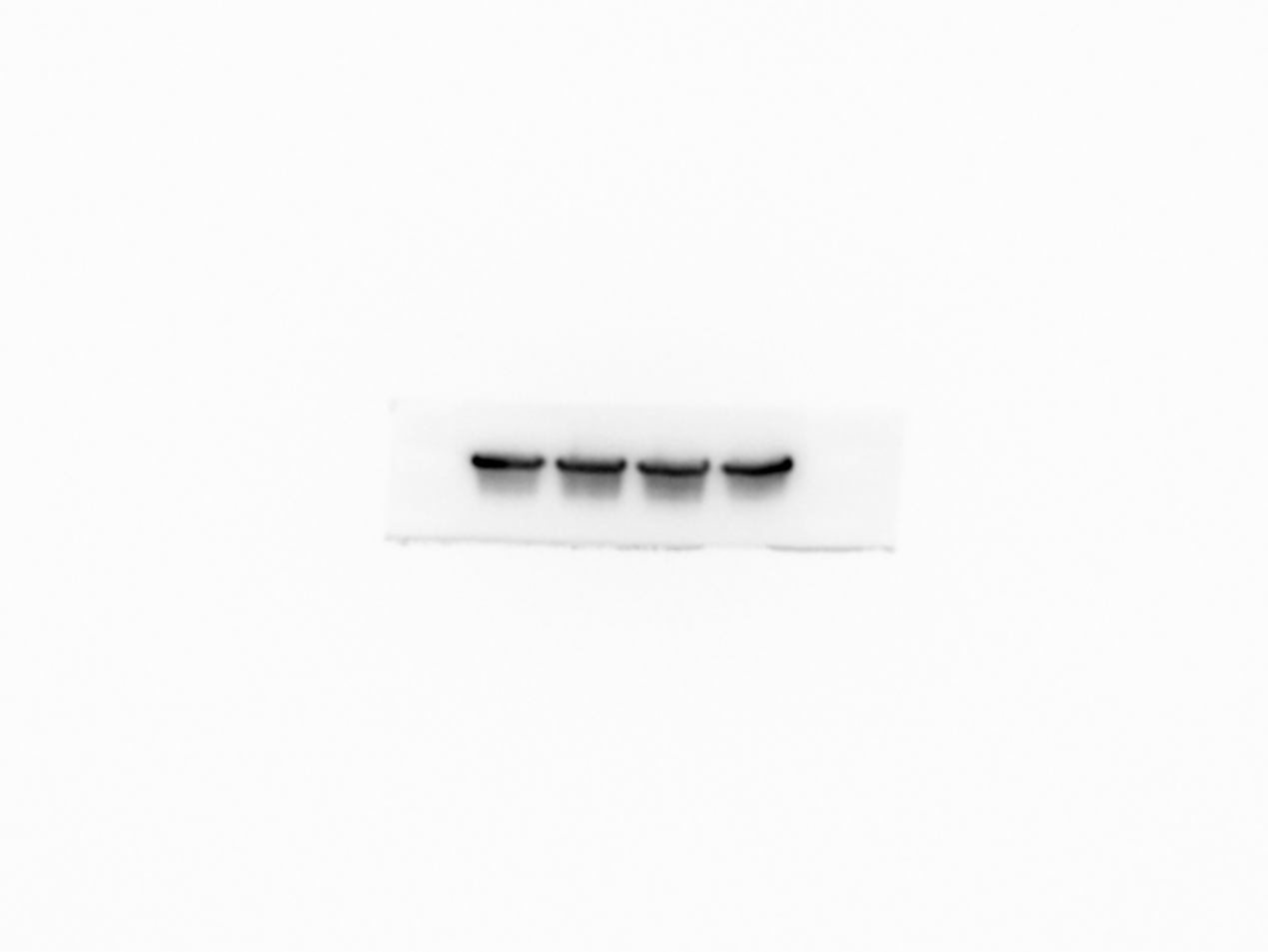


Figure7B

DOK

ADA


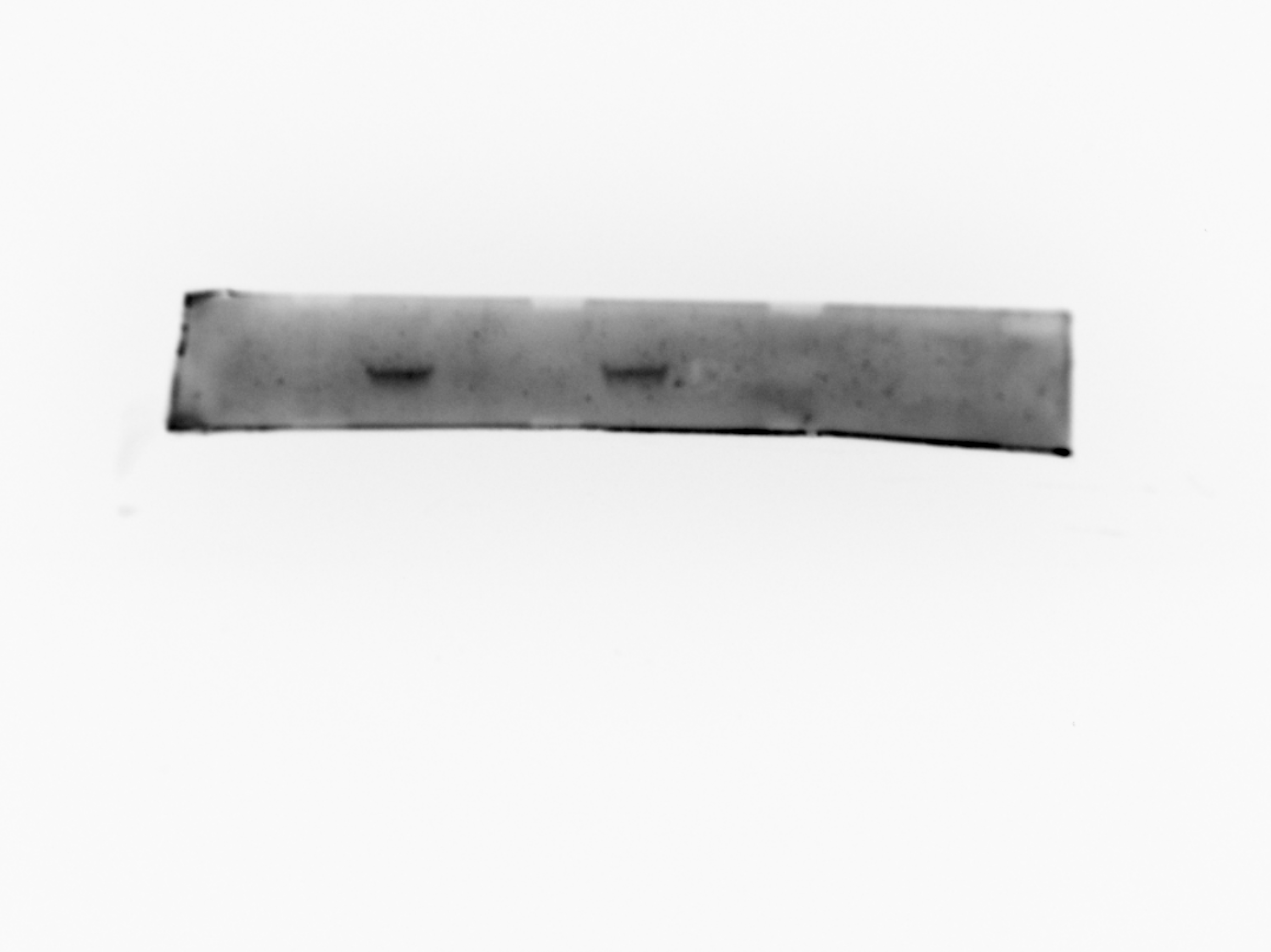


Tubulin


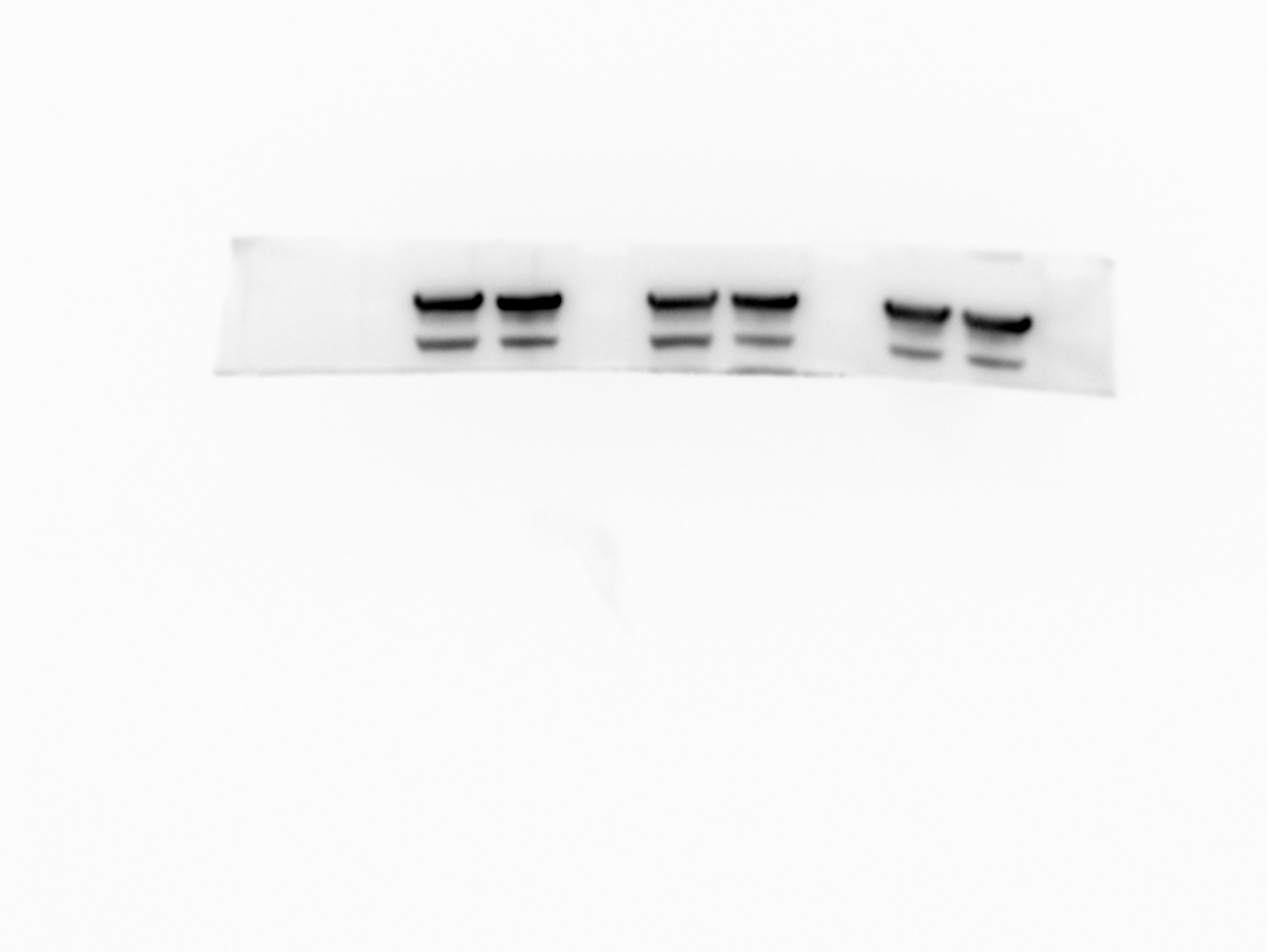


SCC15

ADA


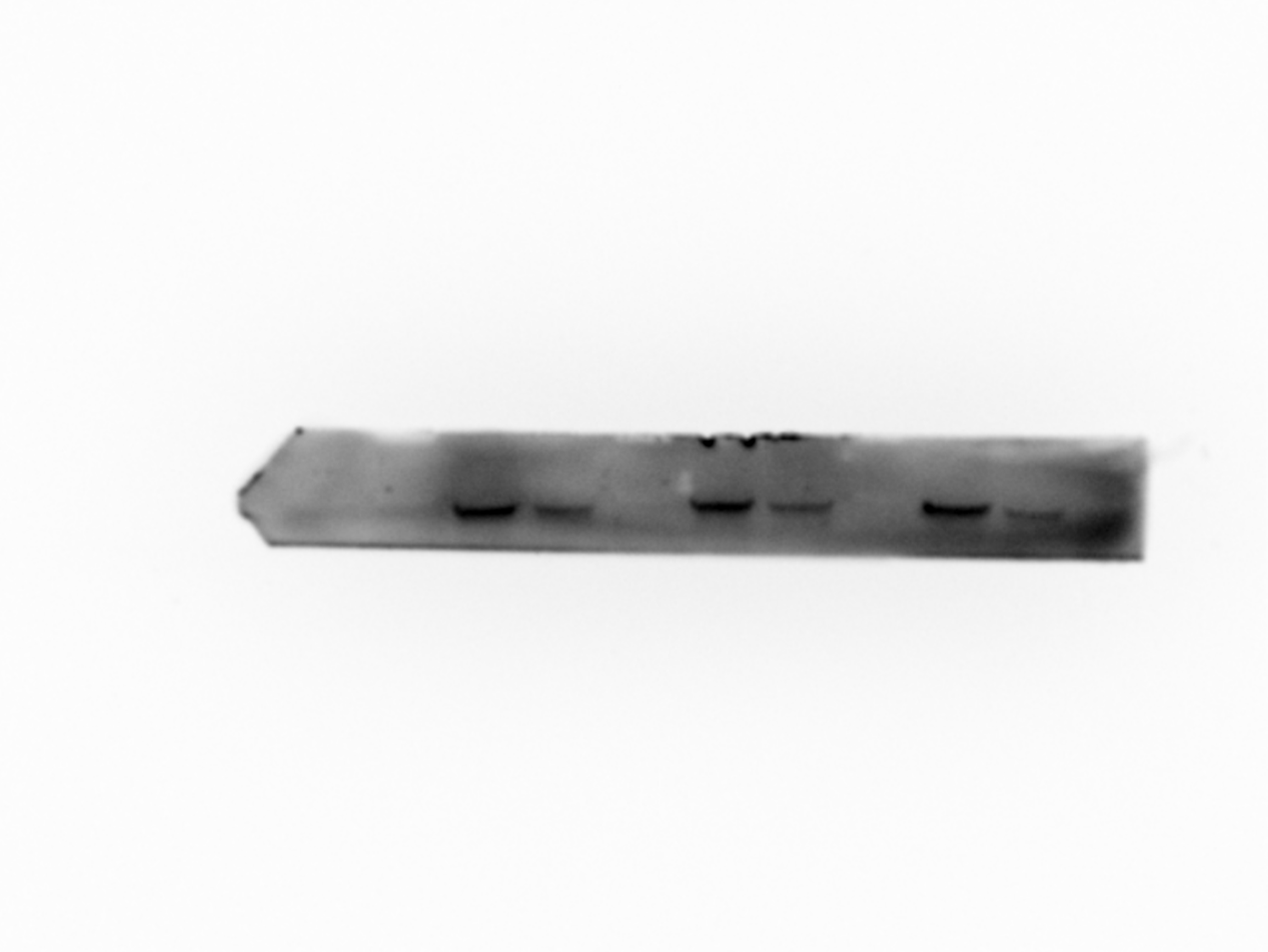


Tubulin


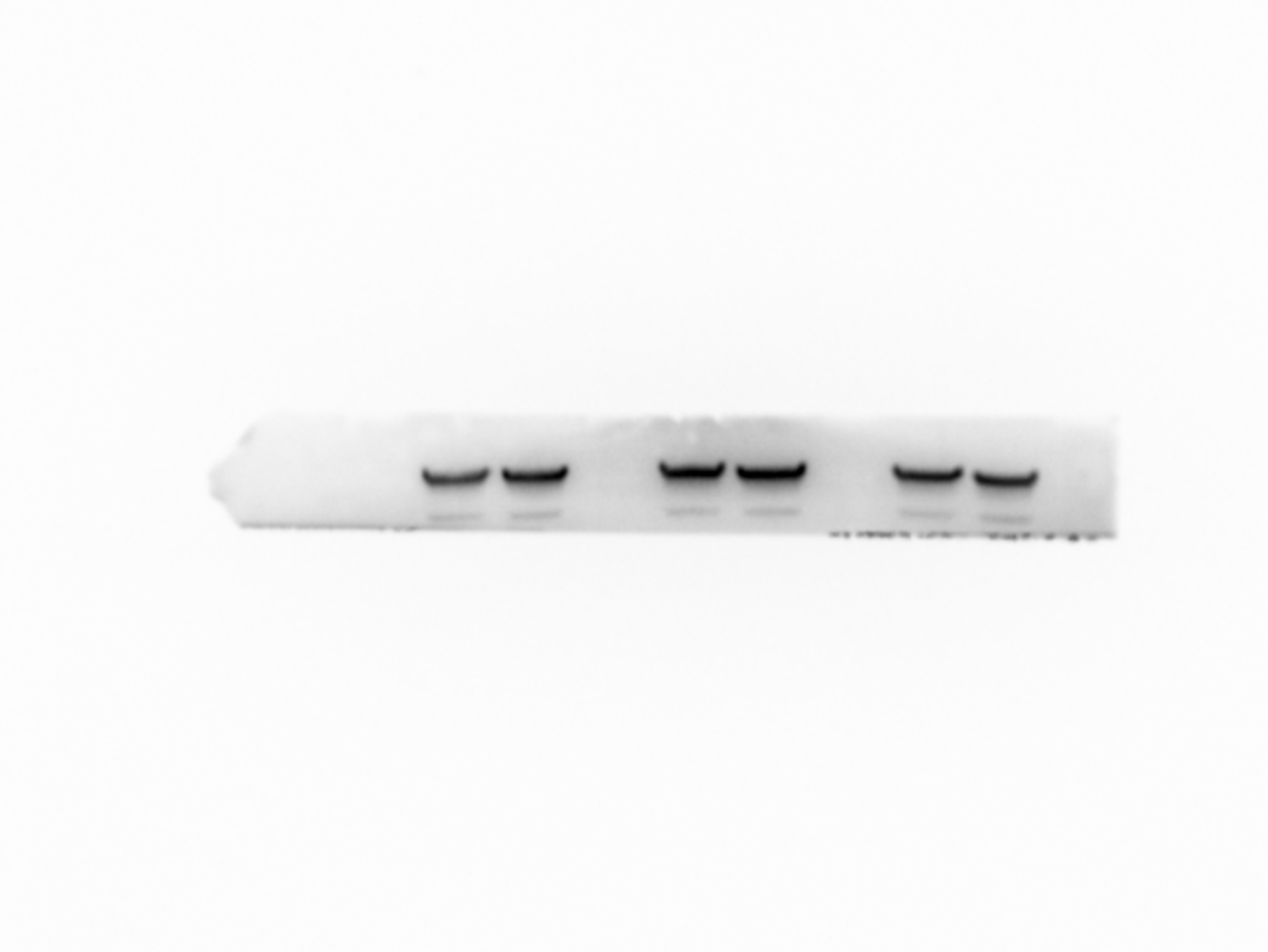


HSC3

ADA


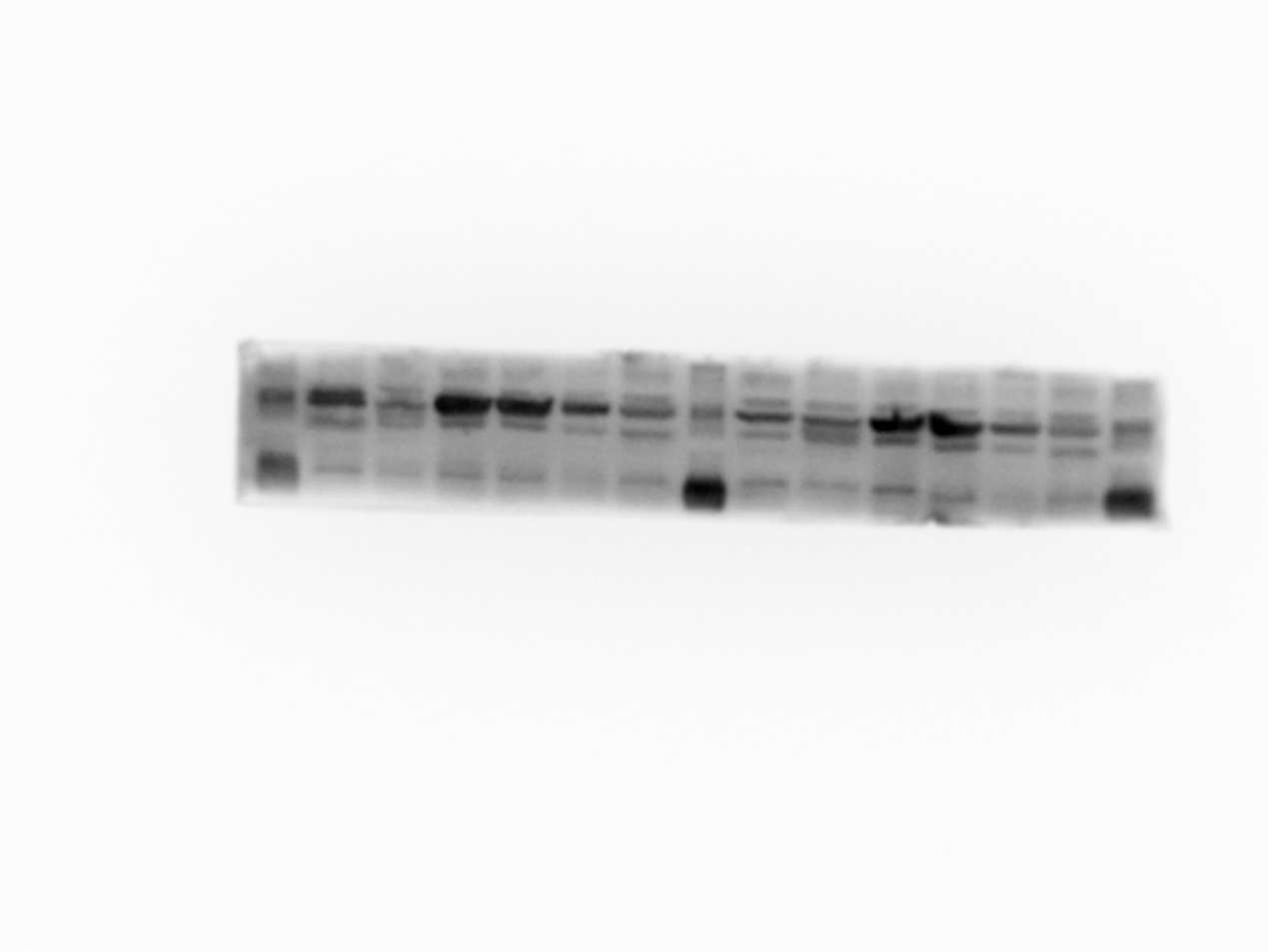


Tubulin


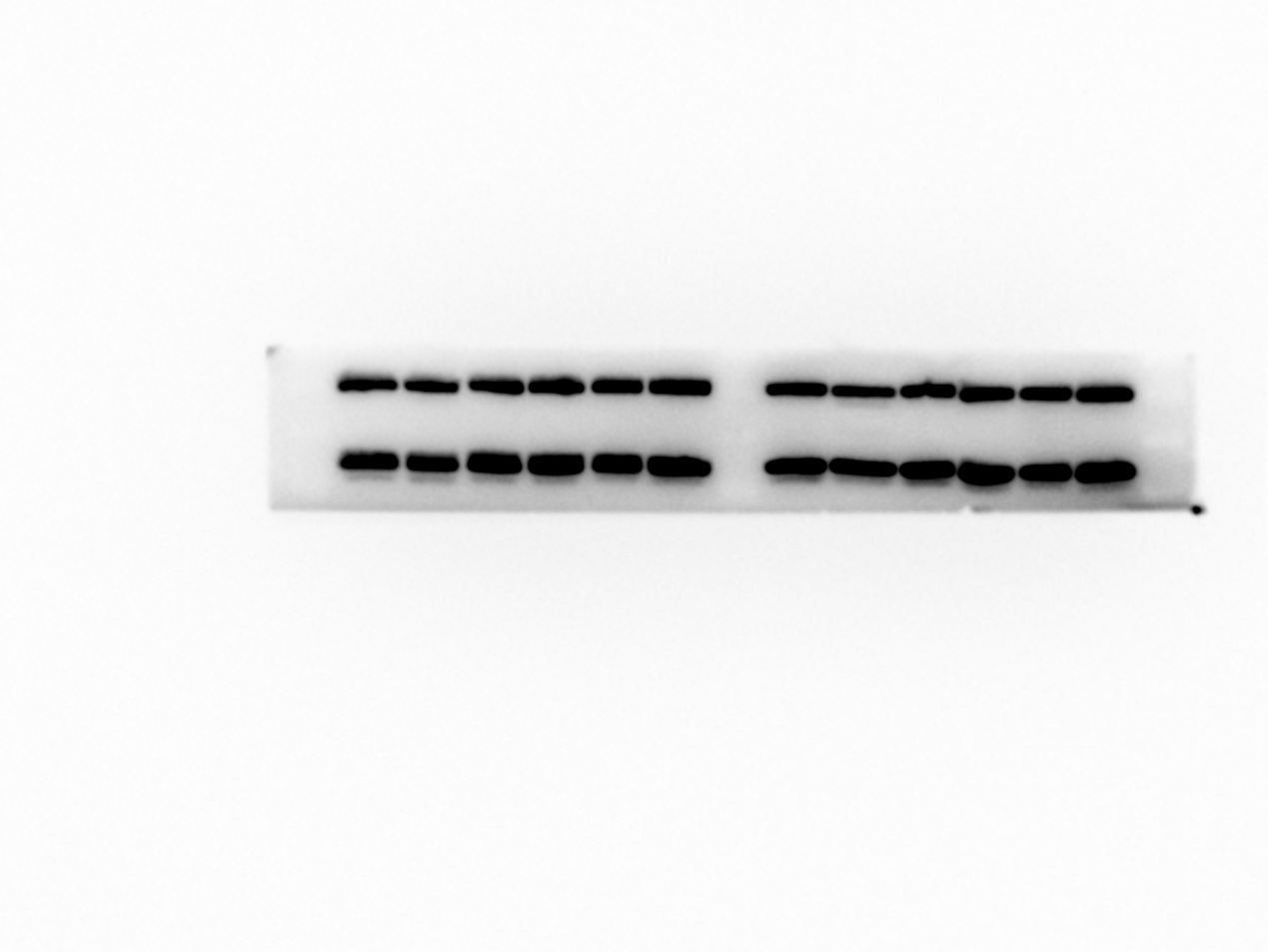


Figure7E

DOK

ADA


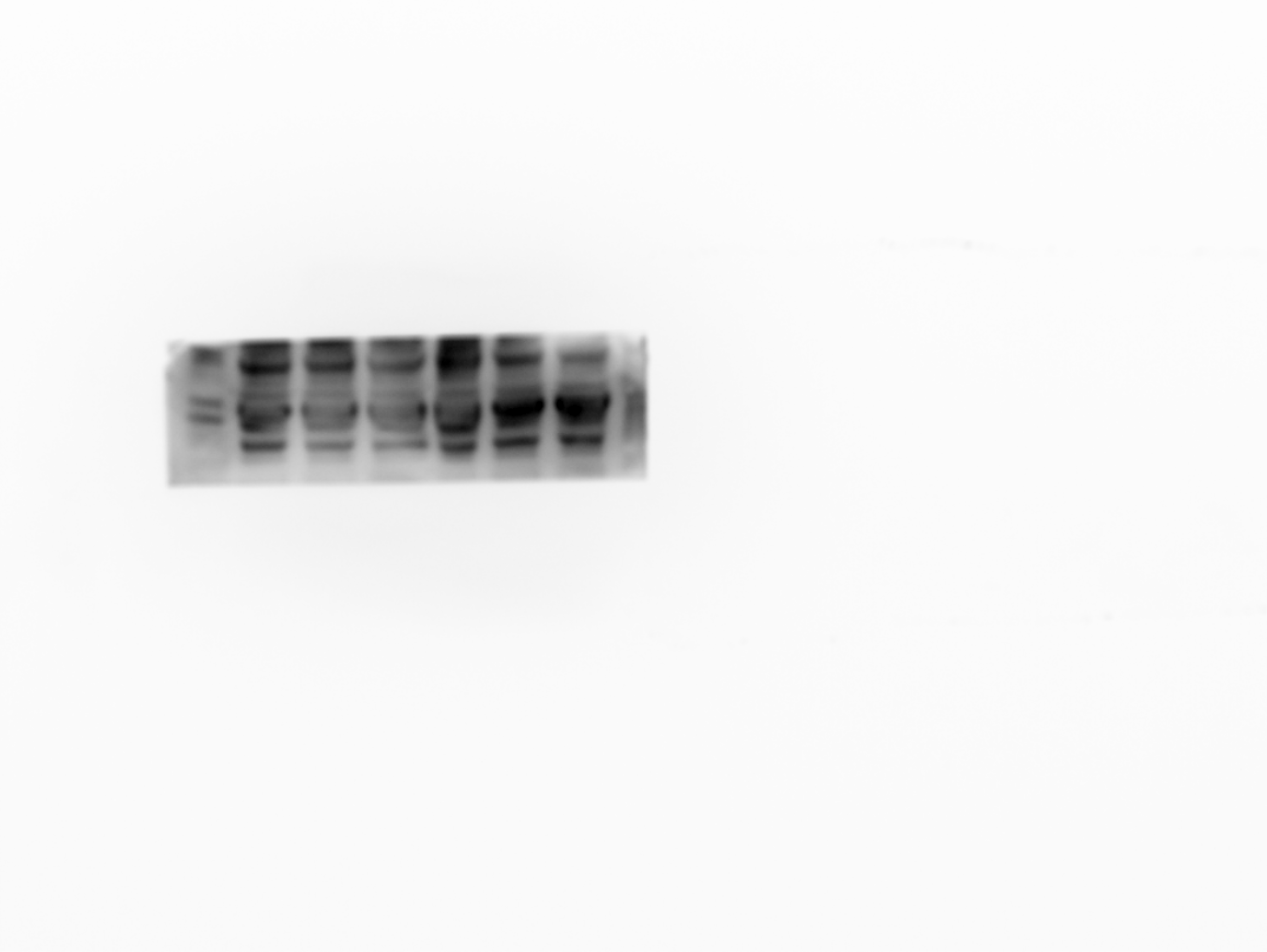


Cleaved-PARP


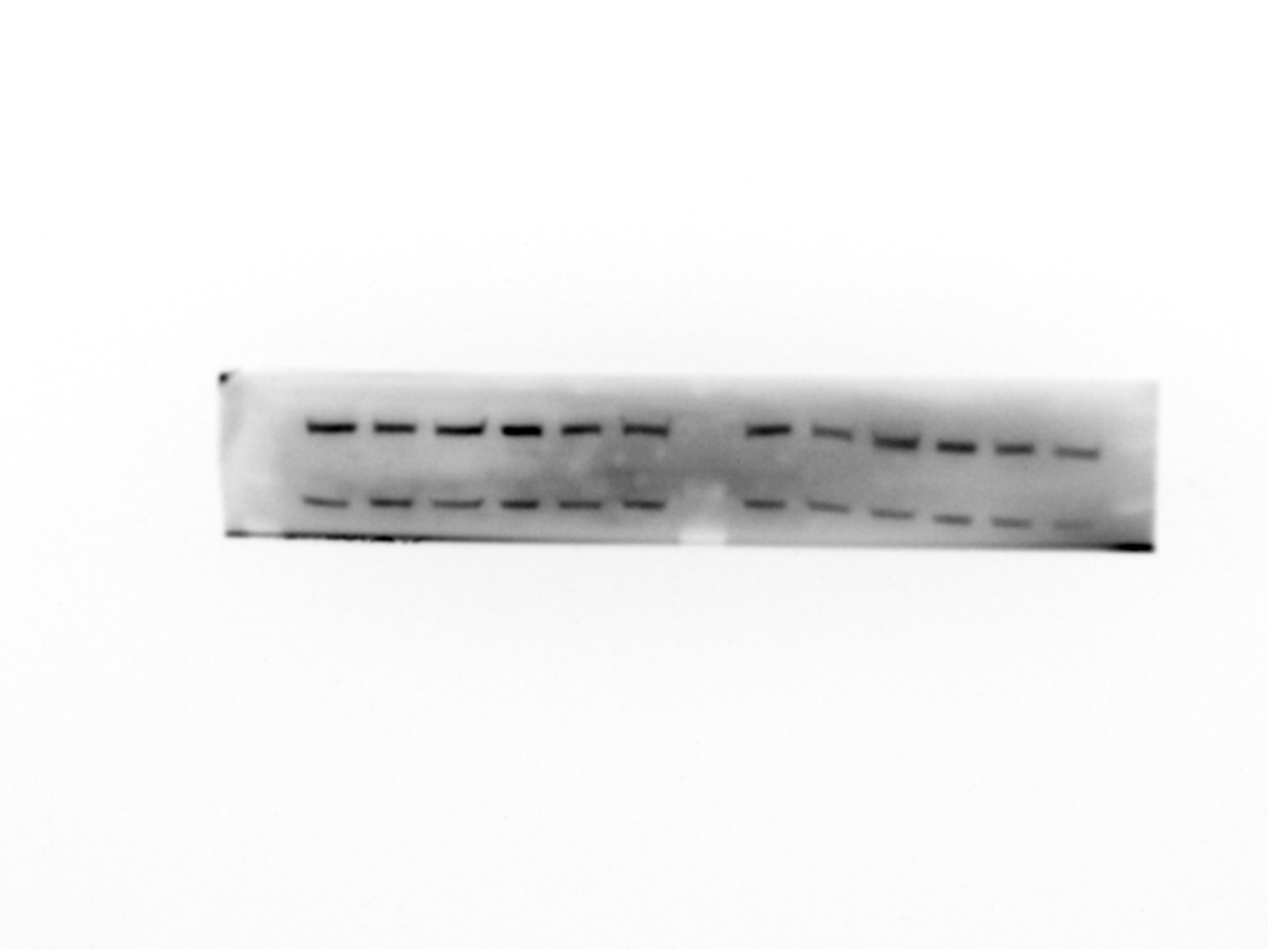


MCL-1


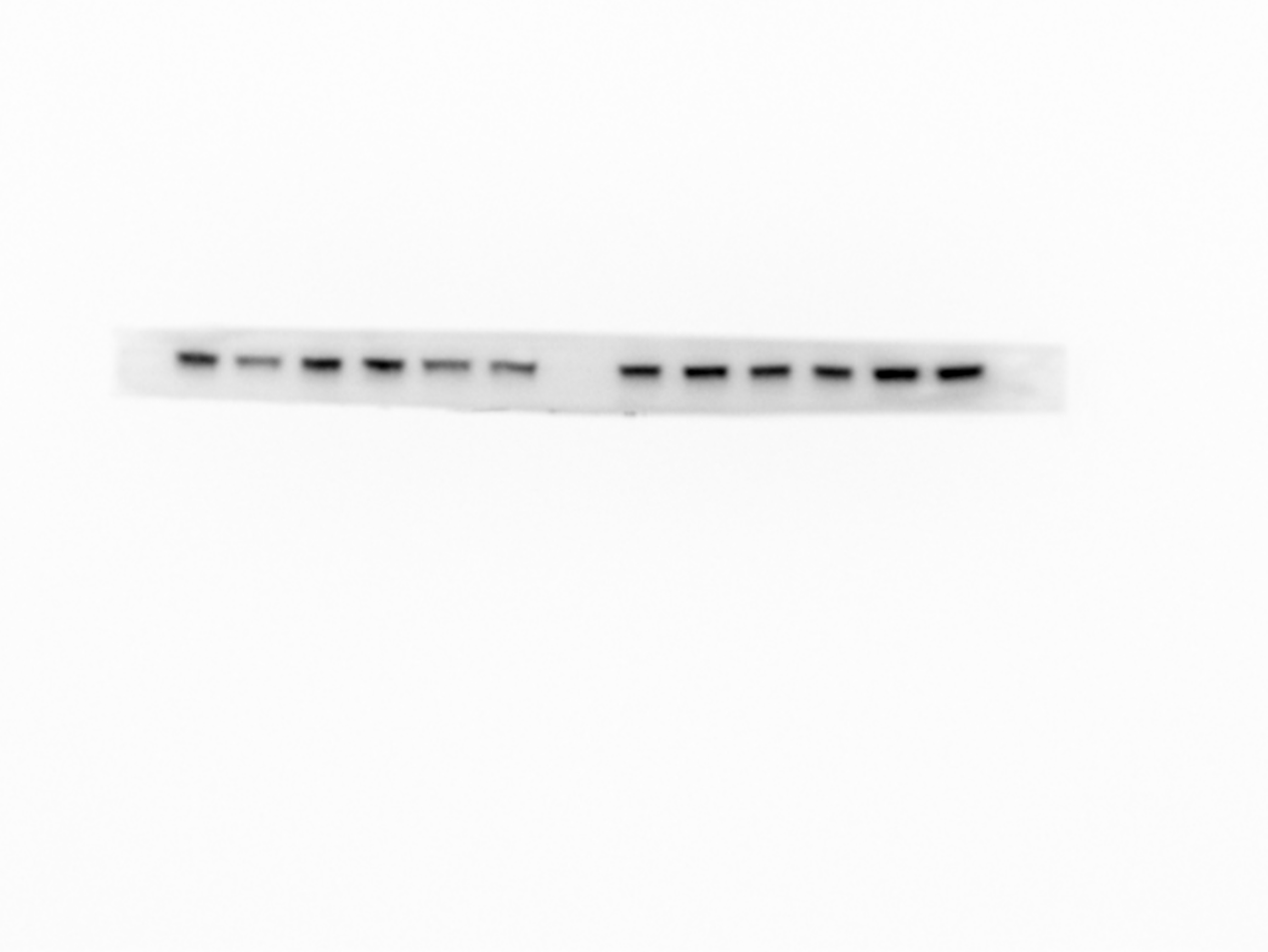


BCL-2


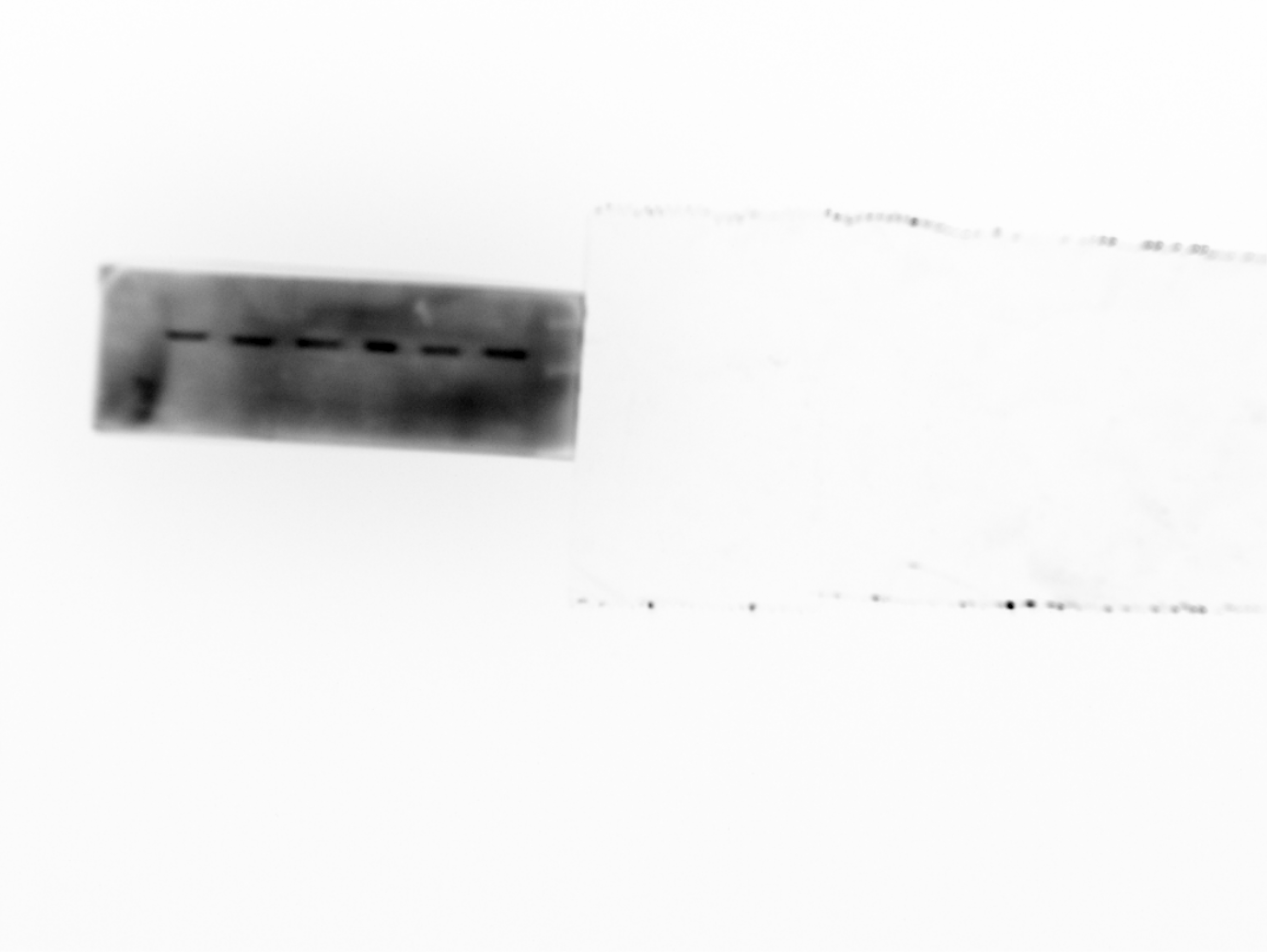


Tubulin


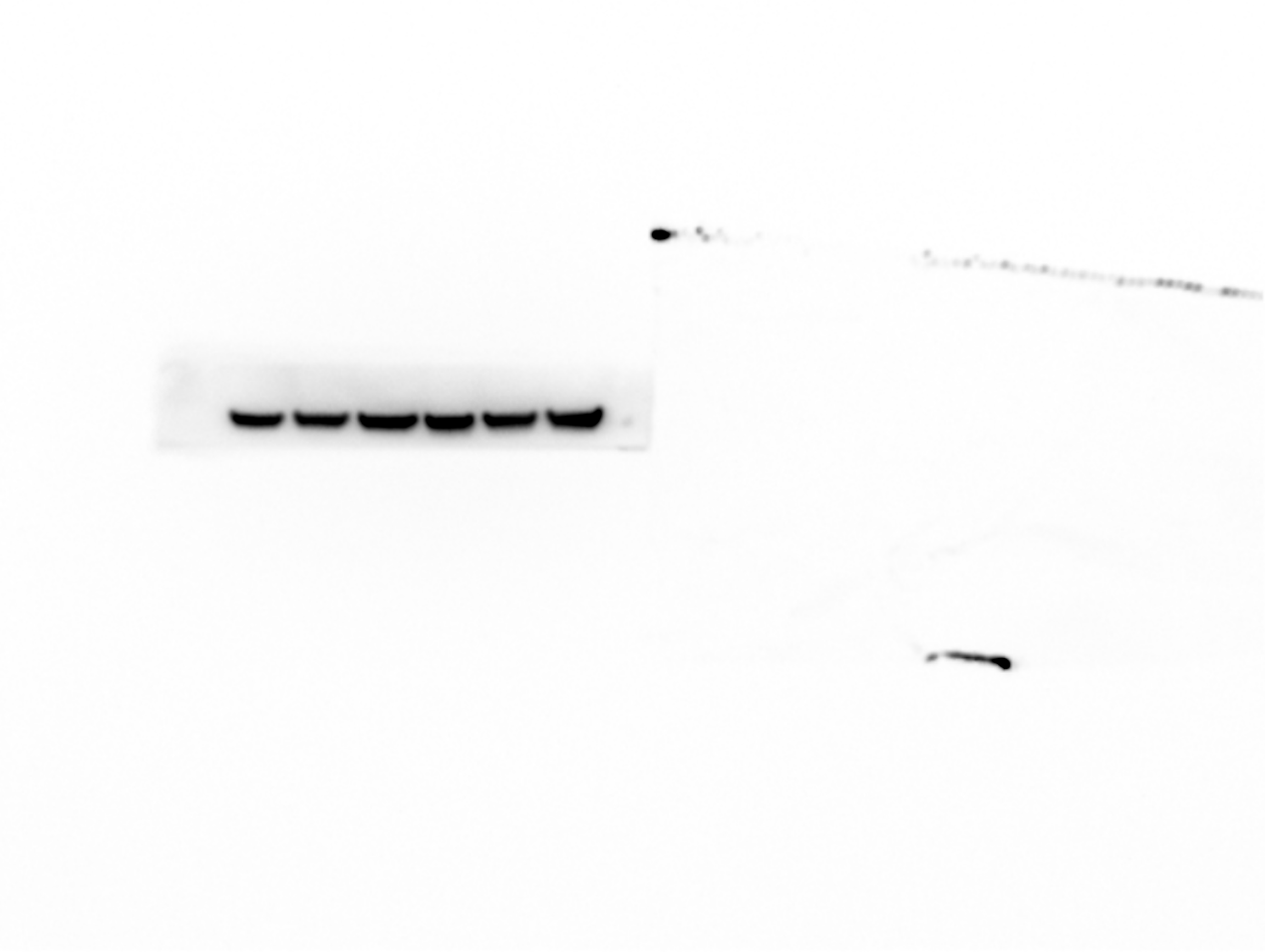


SCC15

ADA


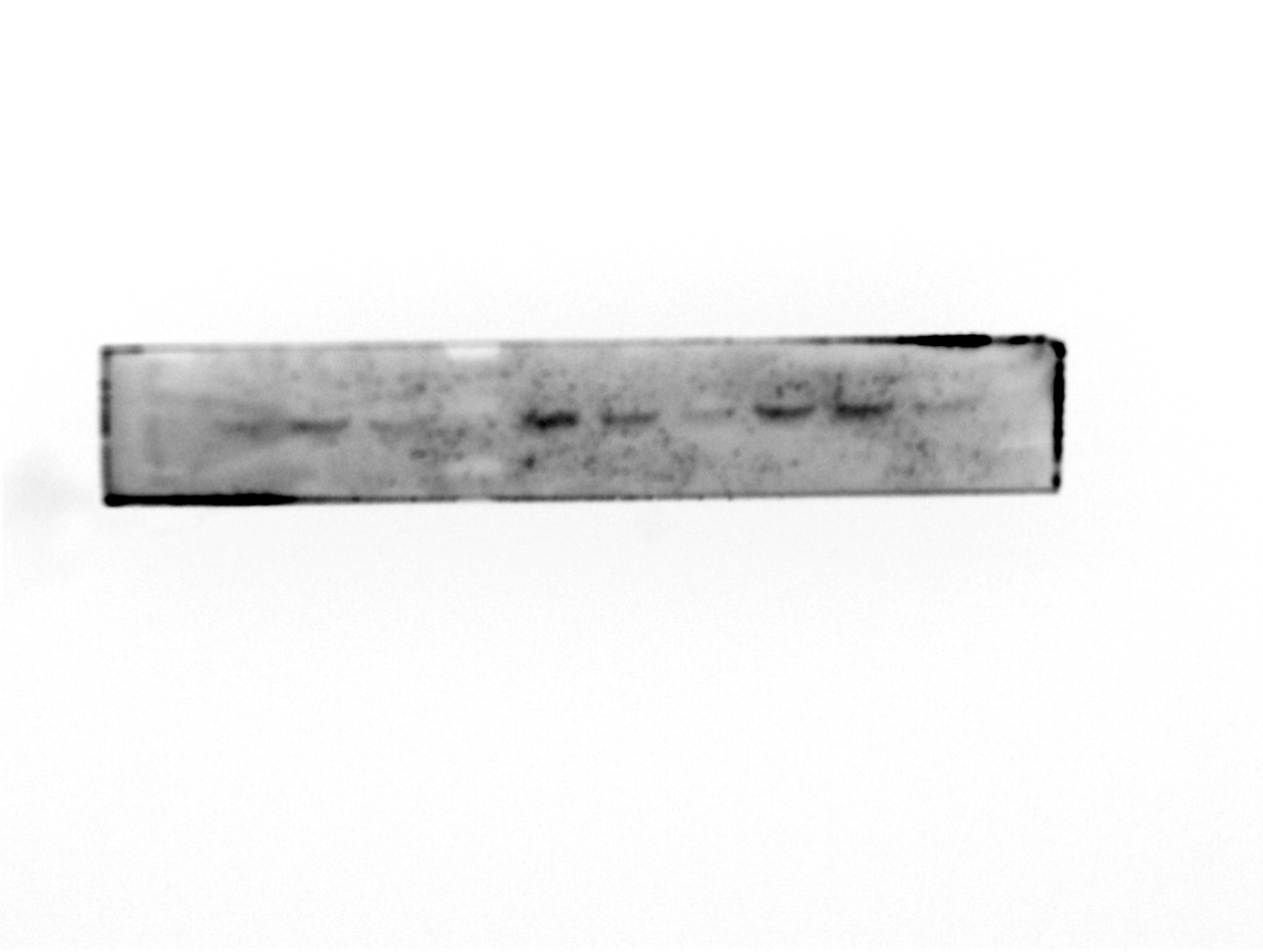


Cleaved-PARP


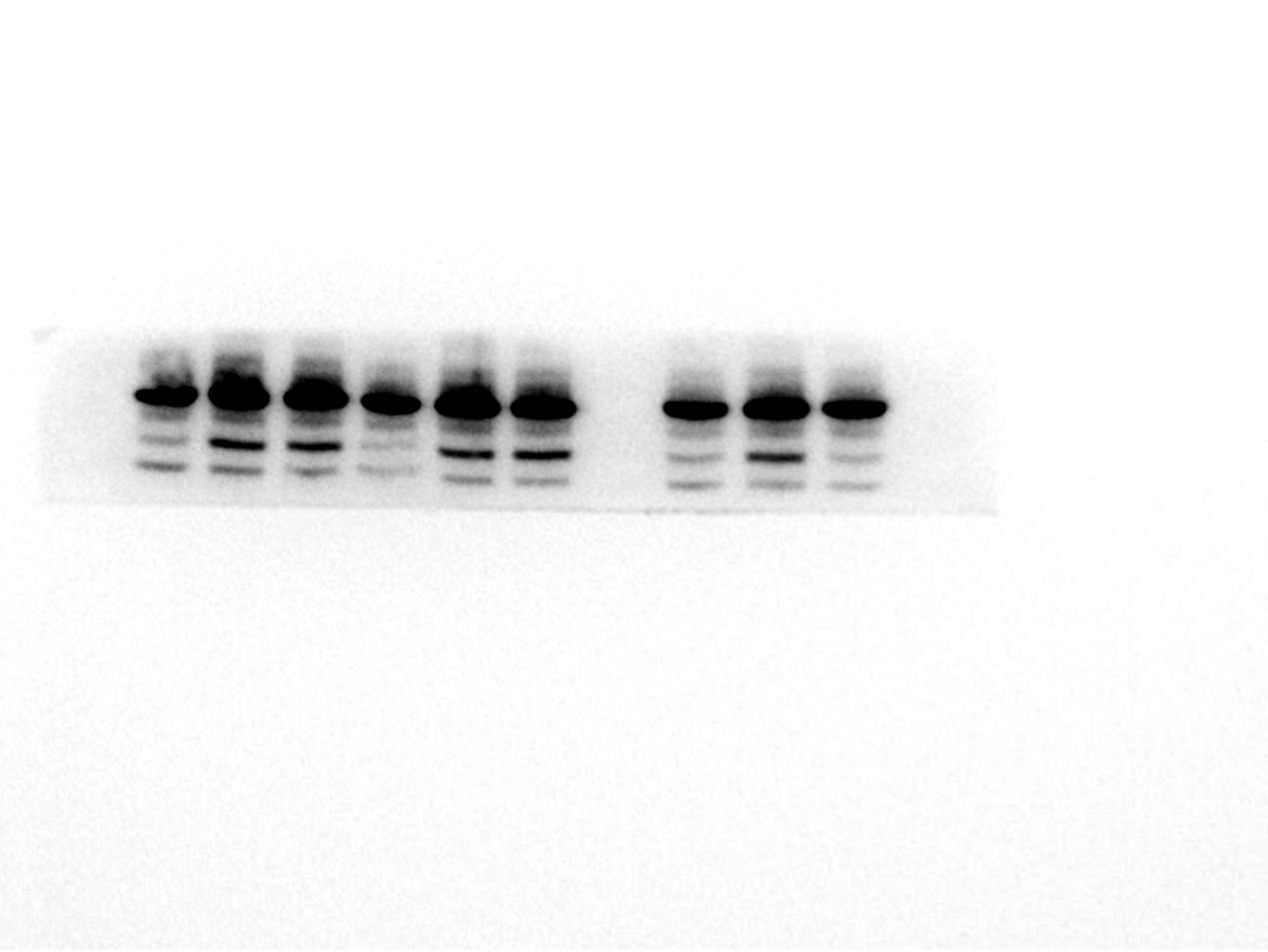


MCL-1


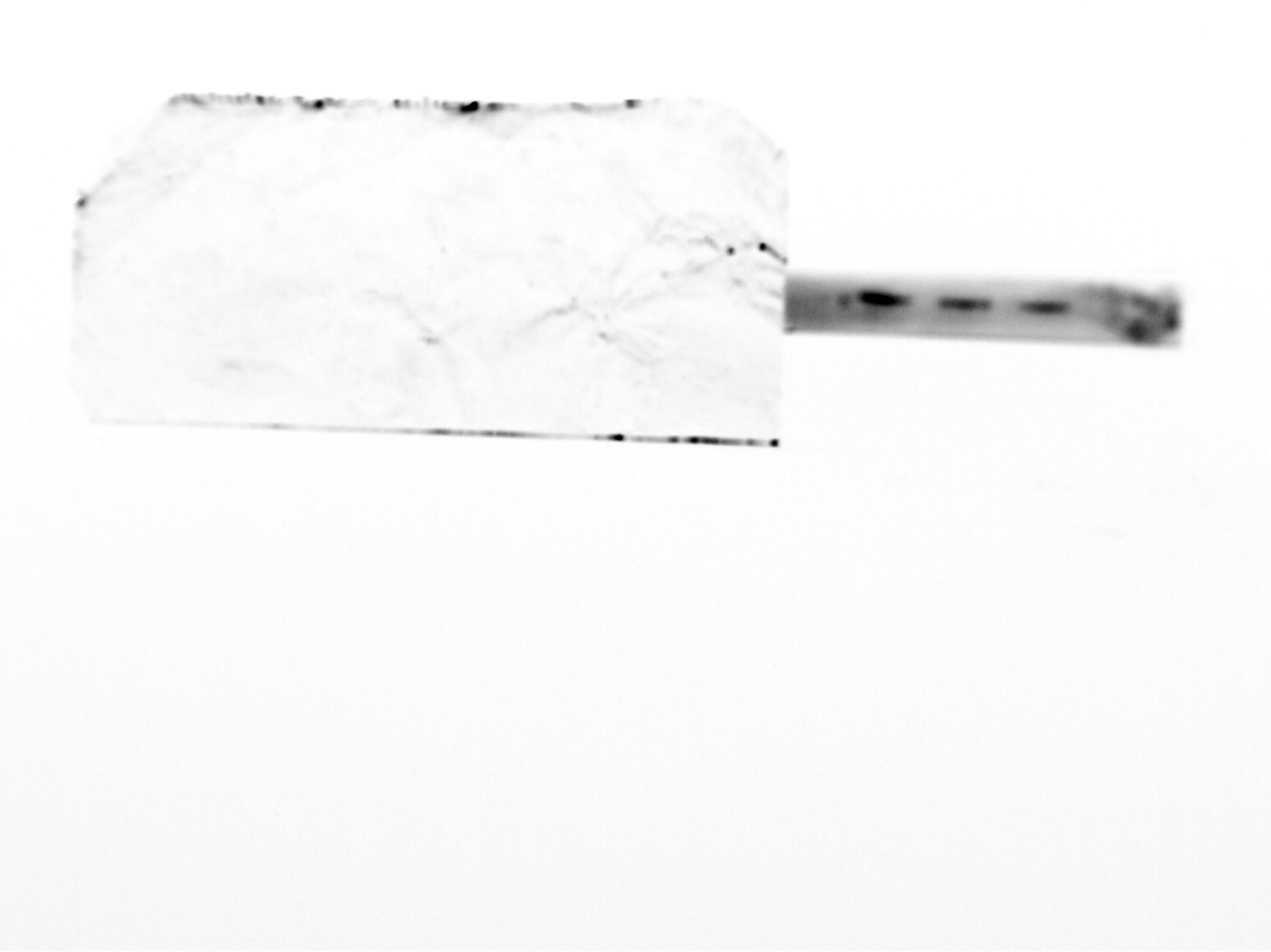


BCL-2


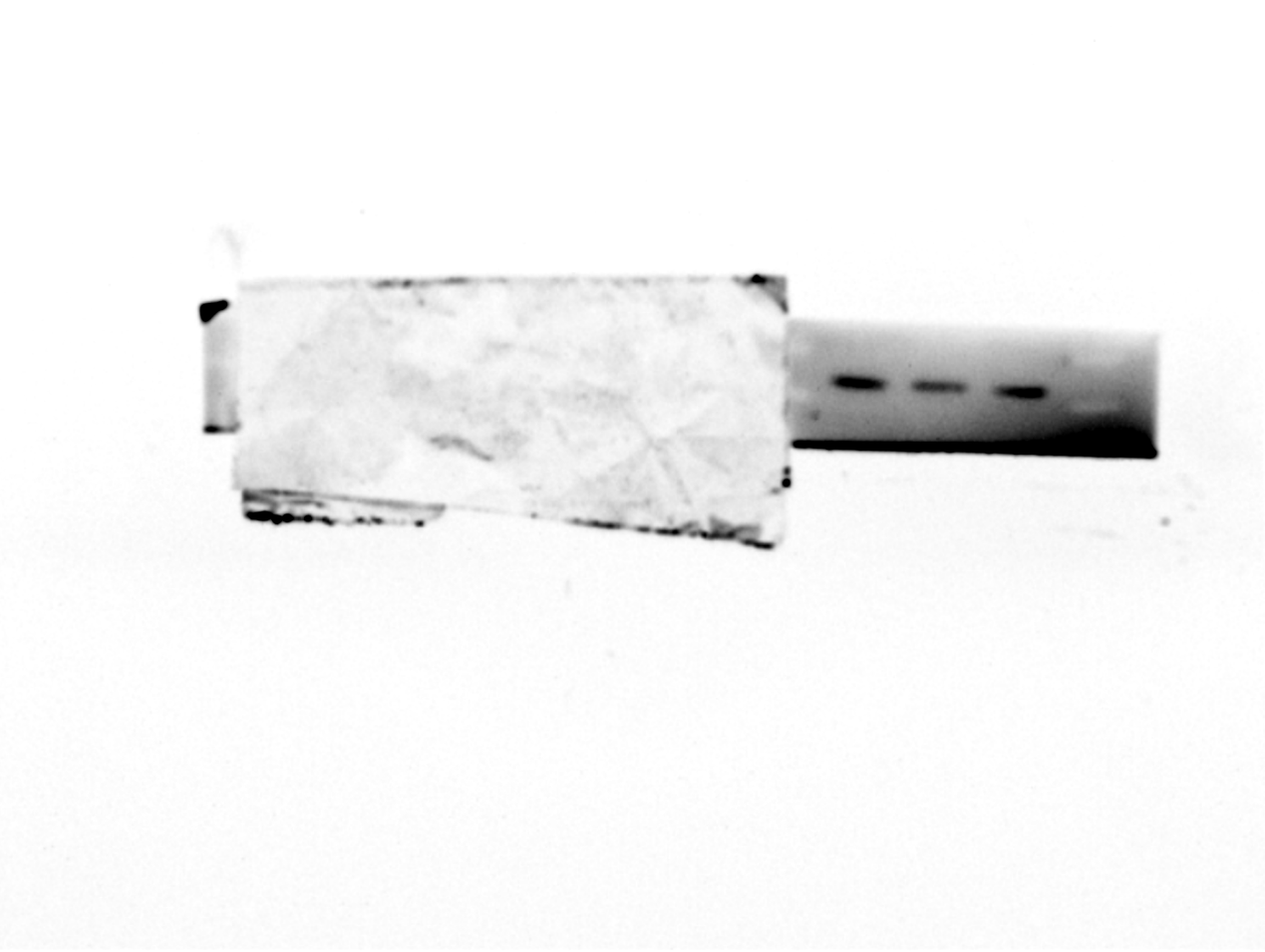


Tubulin


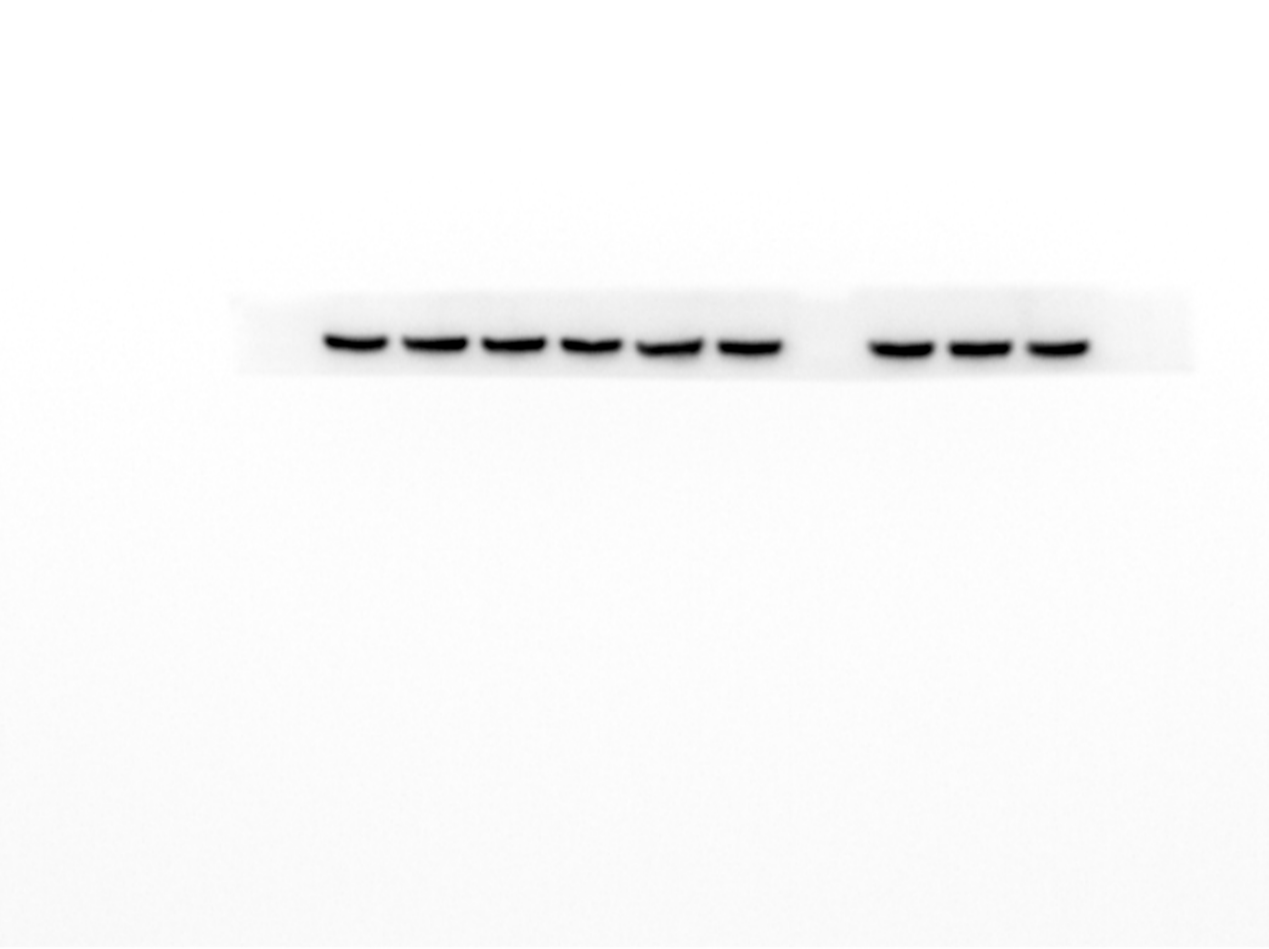


HSC3

ADA


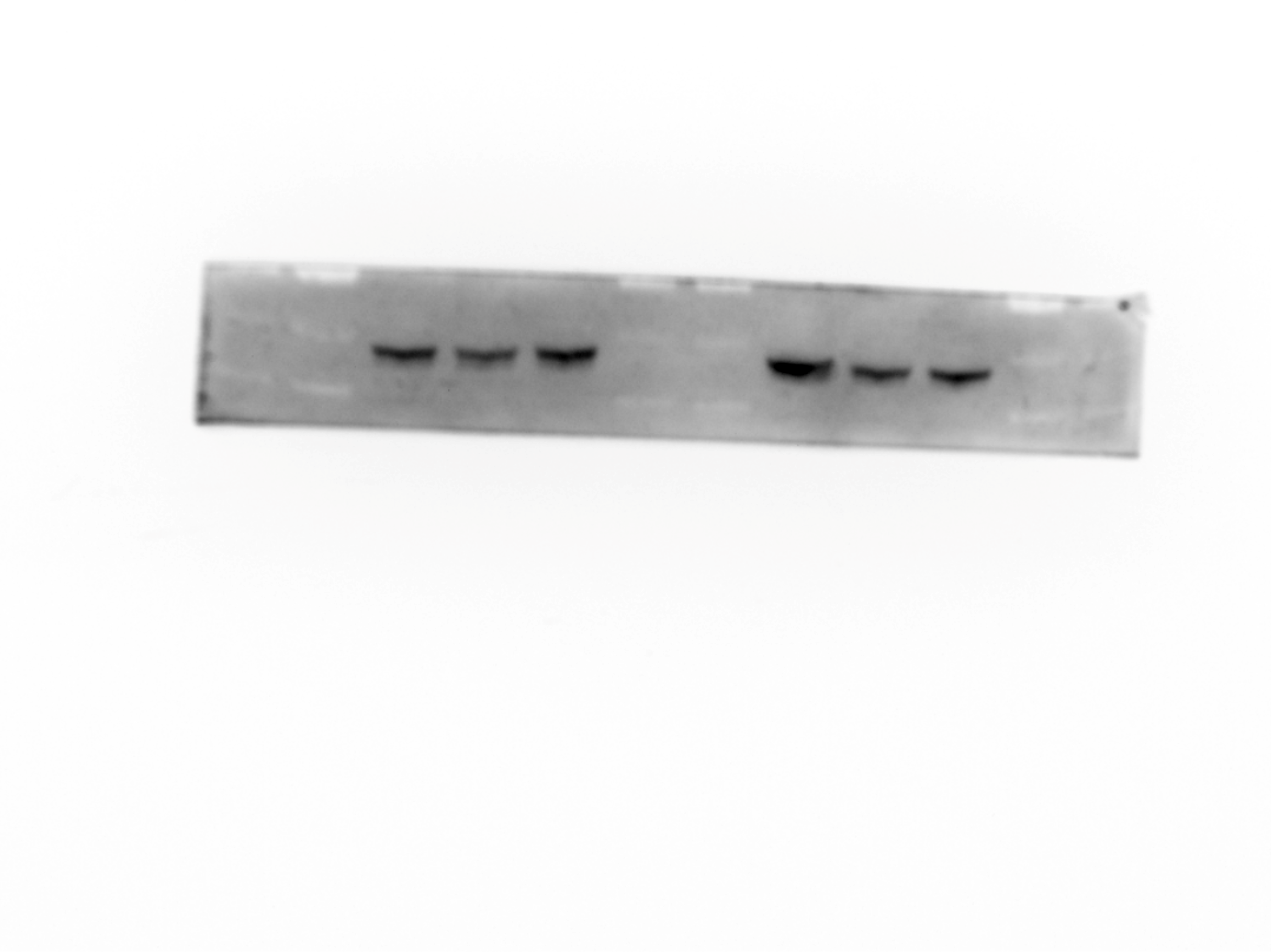


Cleaved-PARP


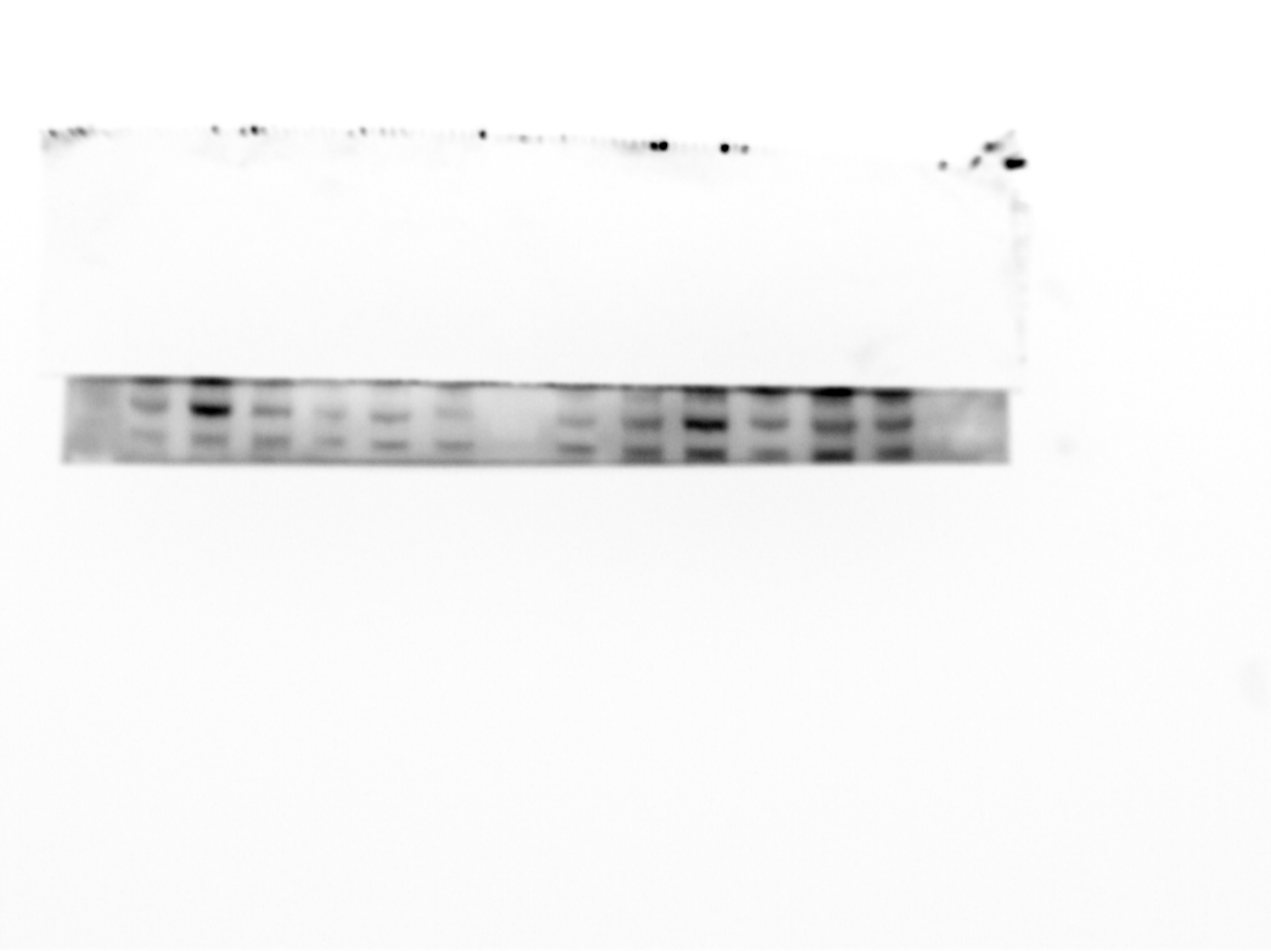


MCL-1


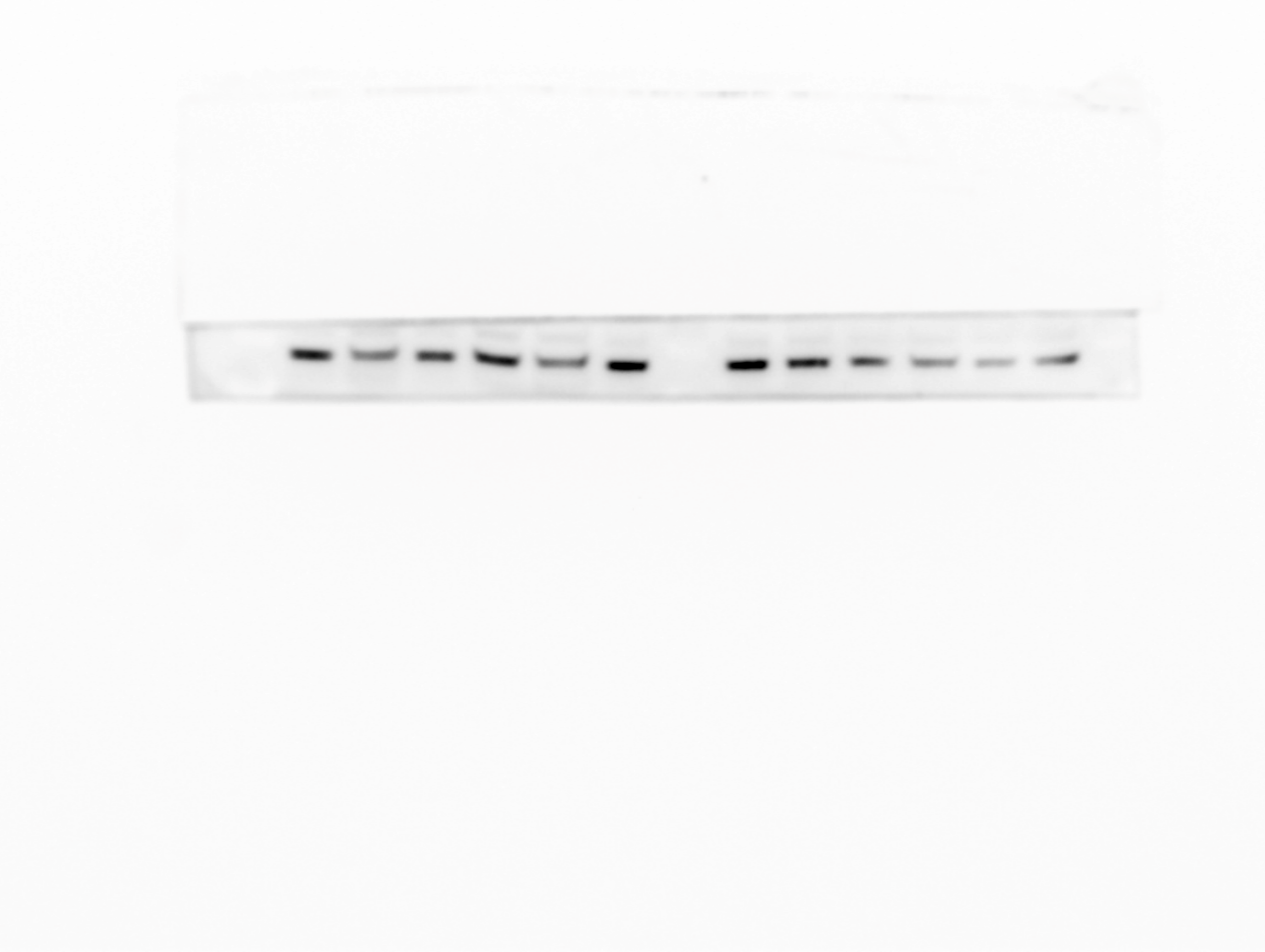


BCL-2


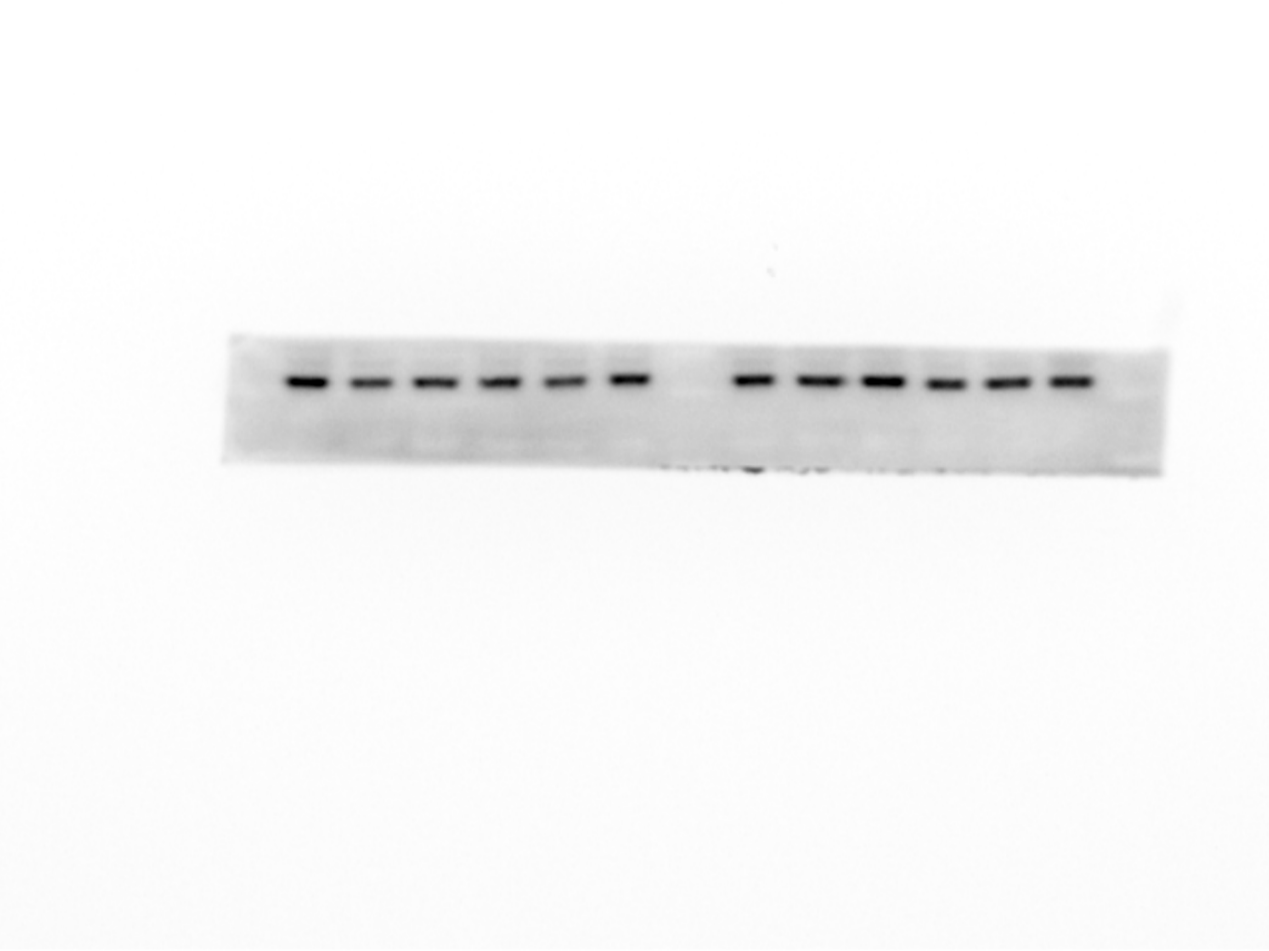


Tubulin


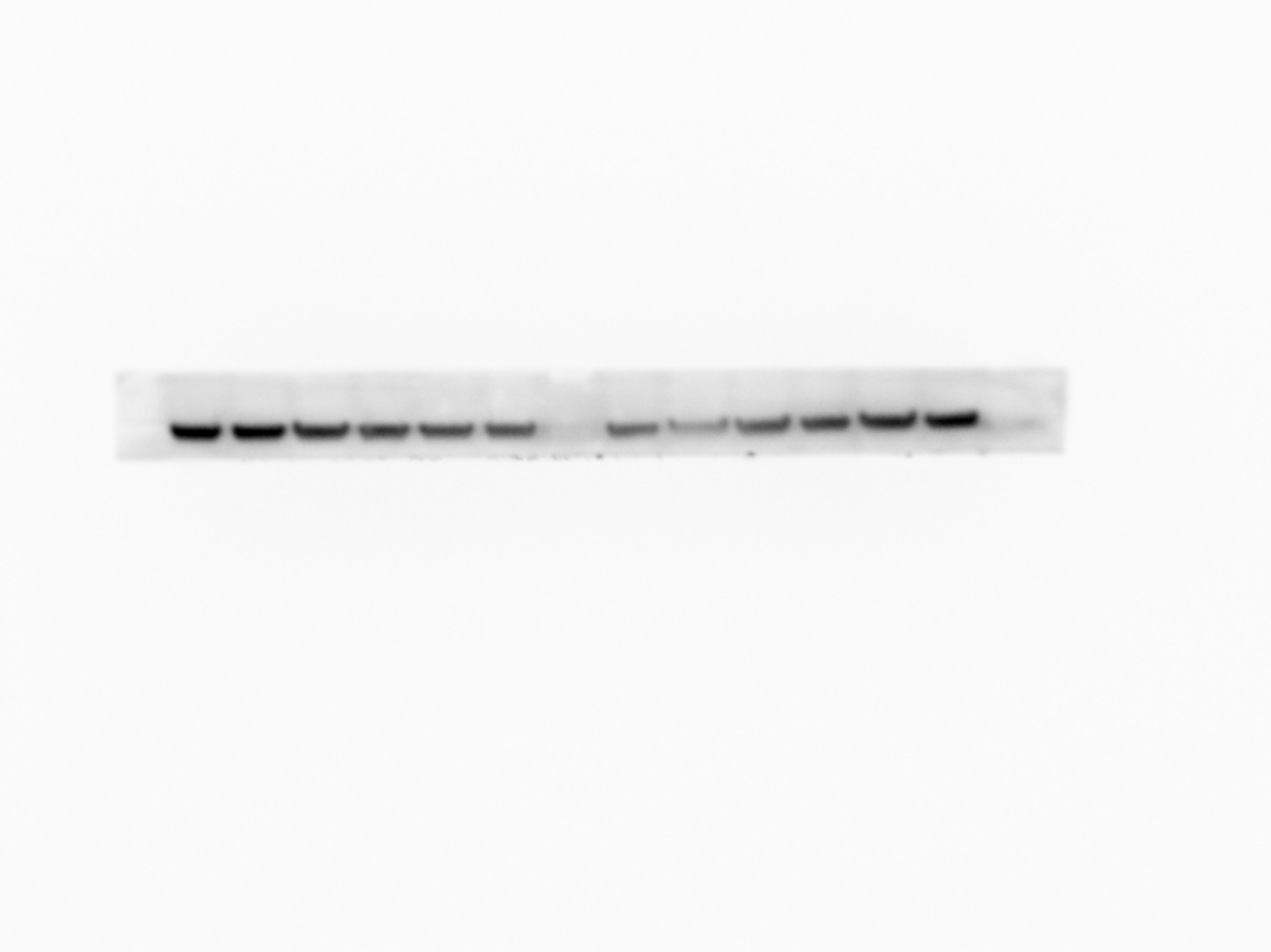


Figure7H

DOK

Cleaved-PARP


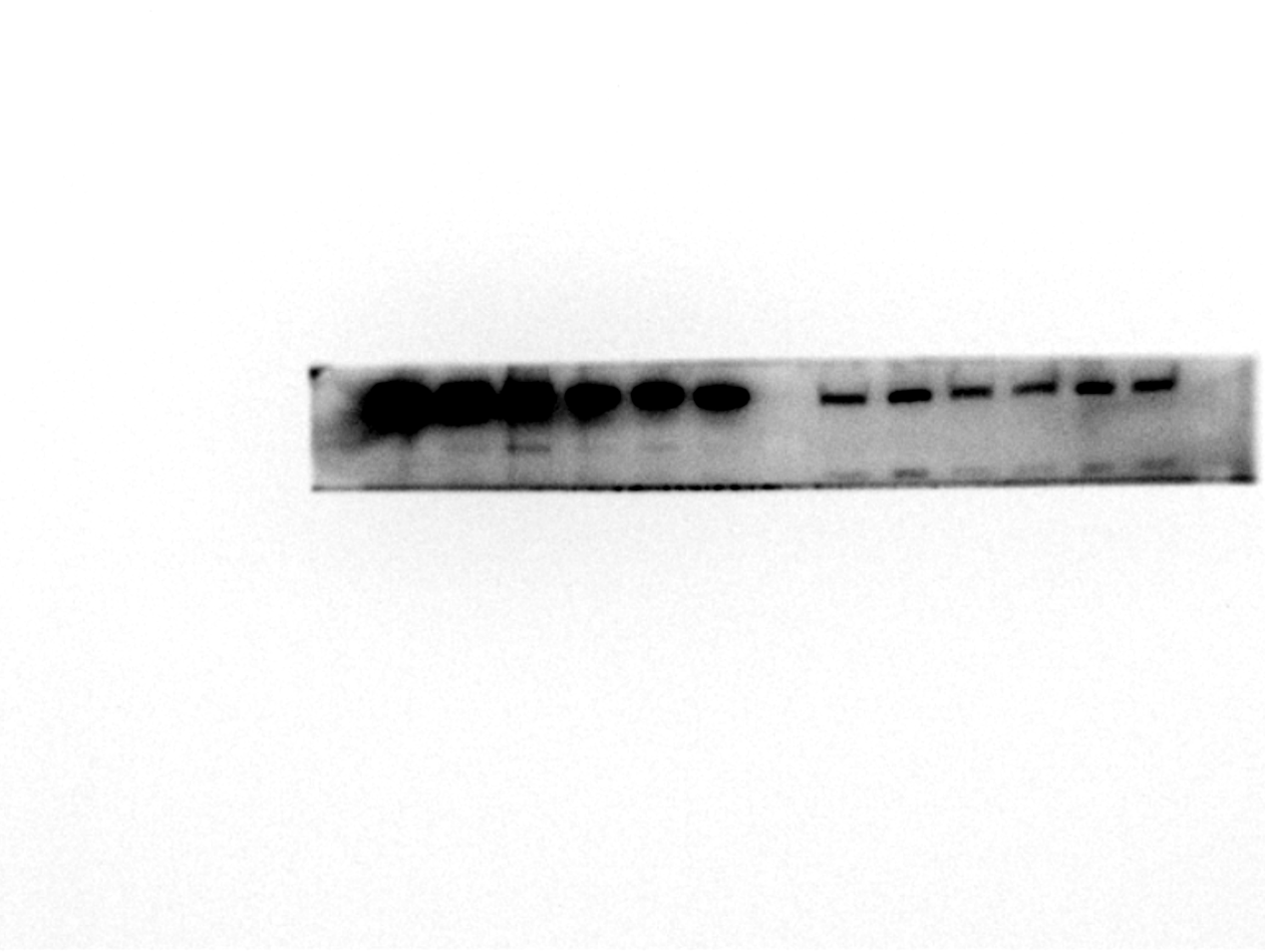


MCL-1


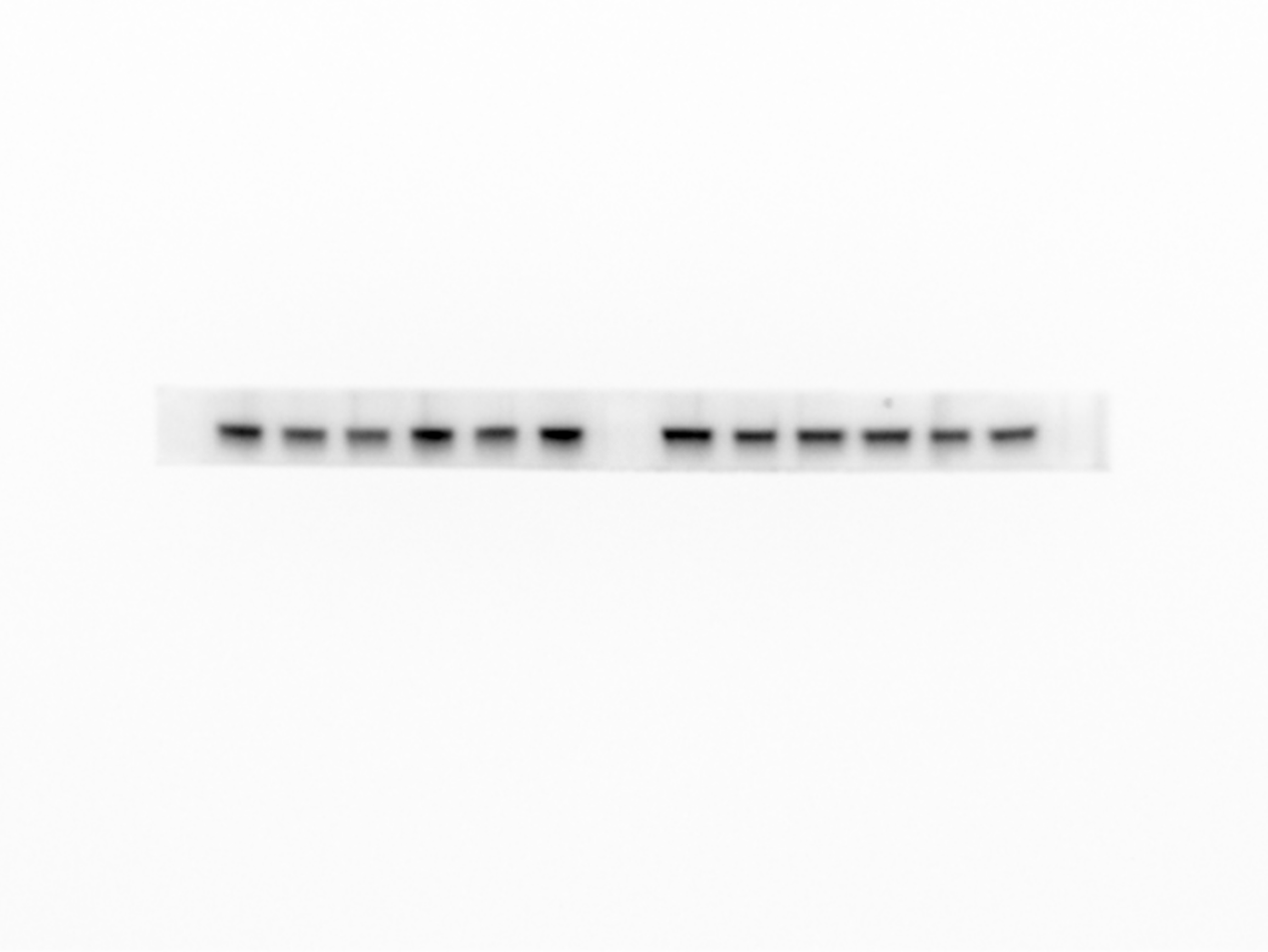


BCL-2


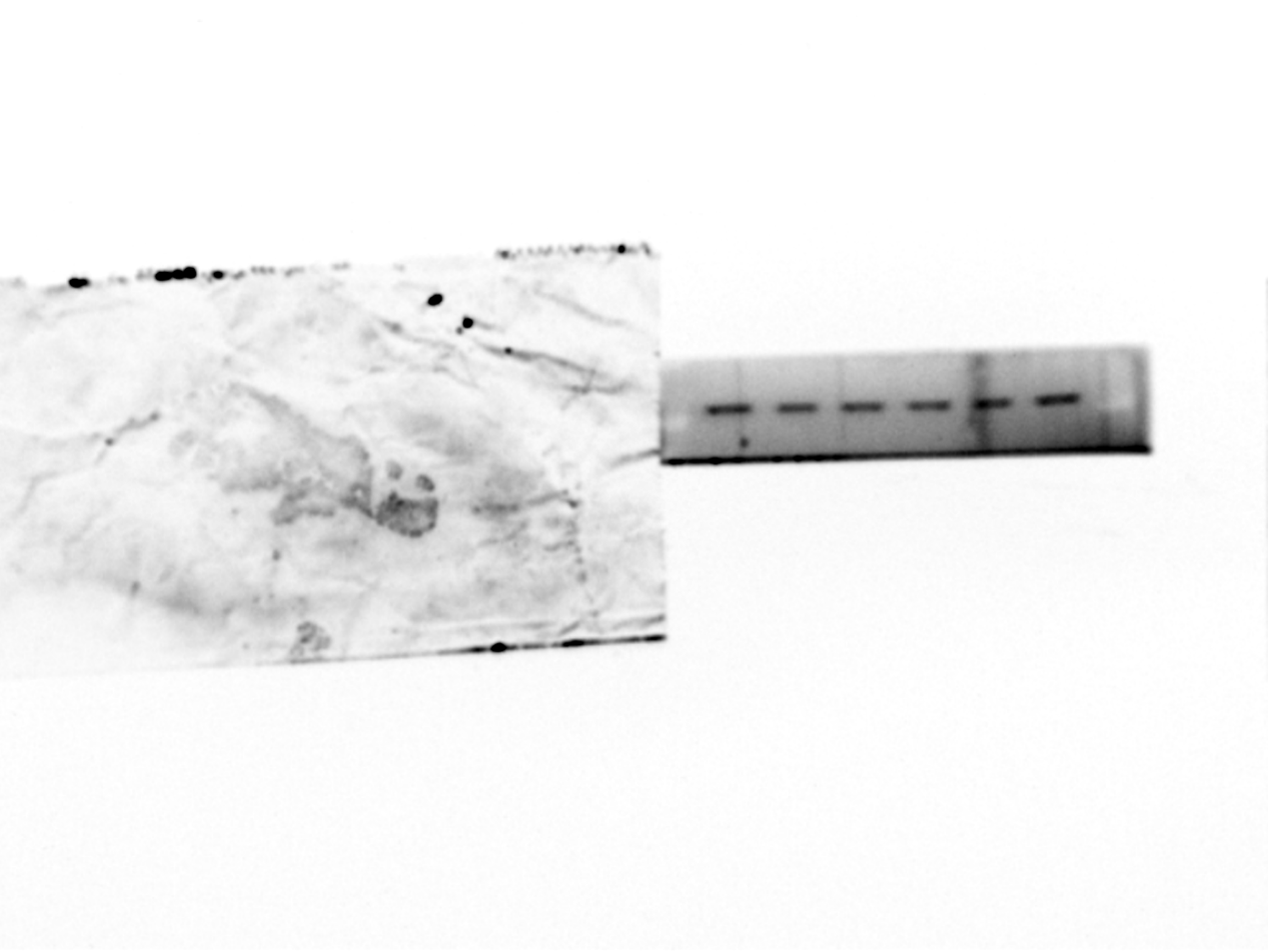


Tubulin


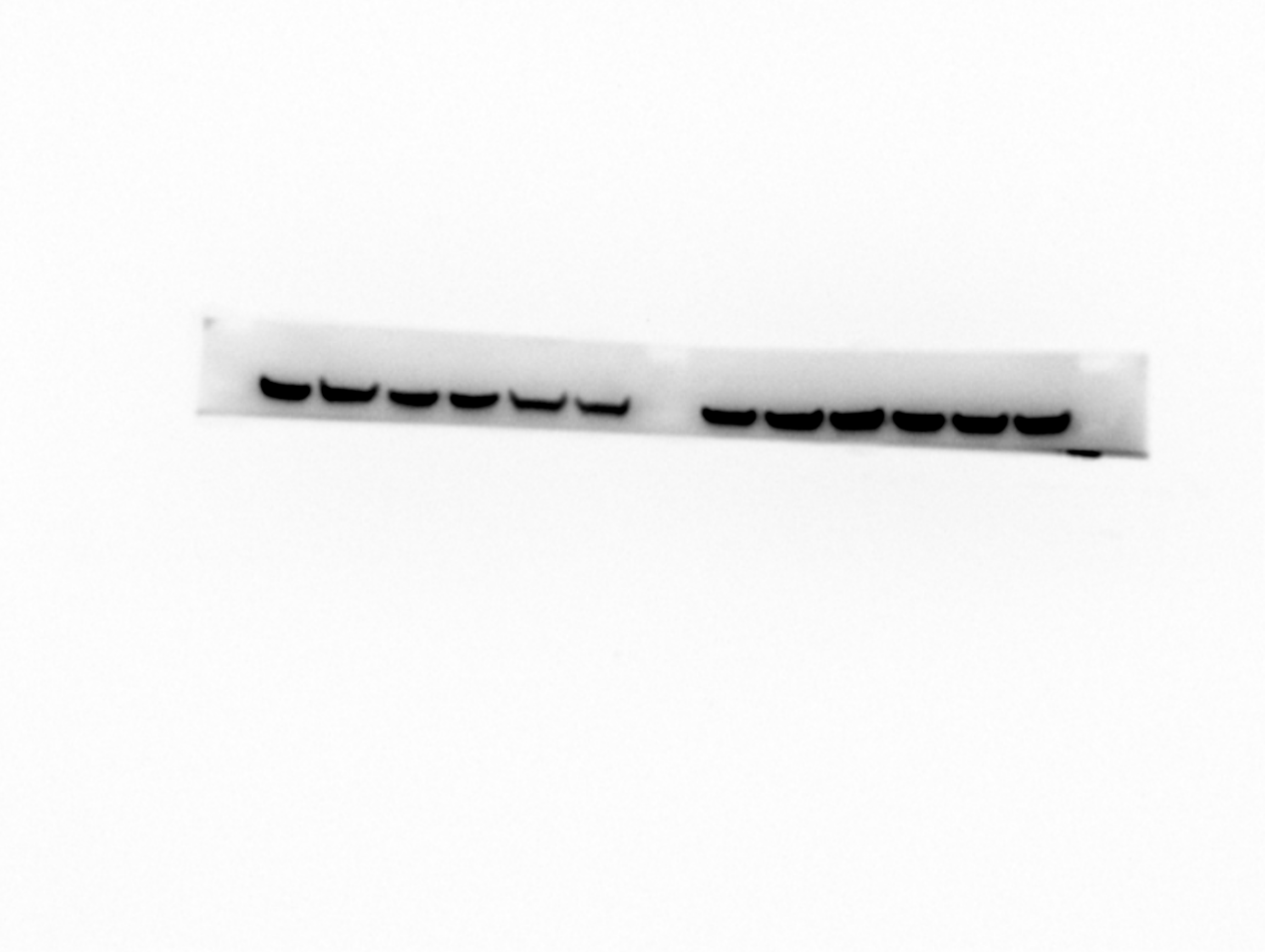


SCC15

Cleaved-PARP


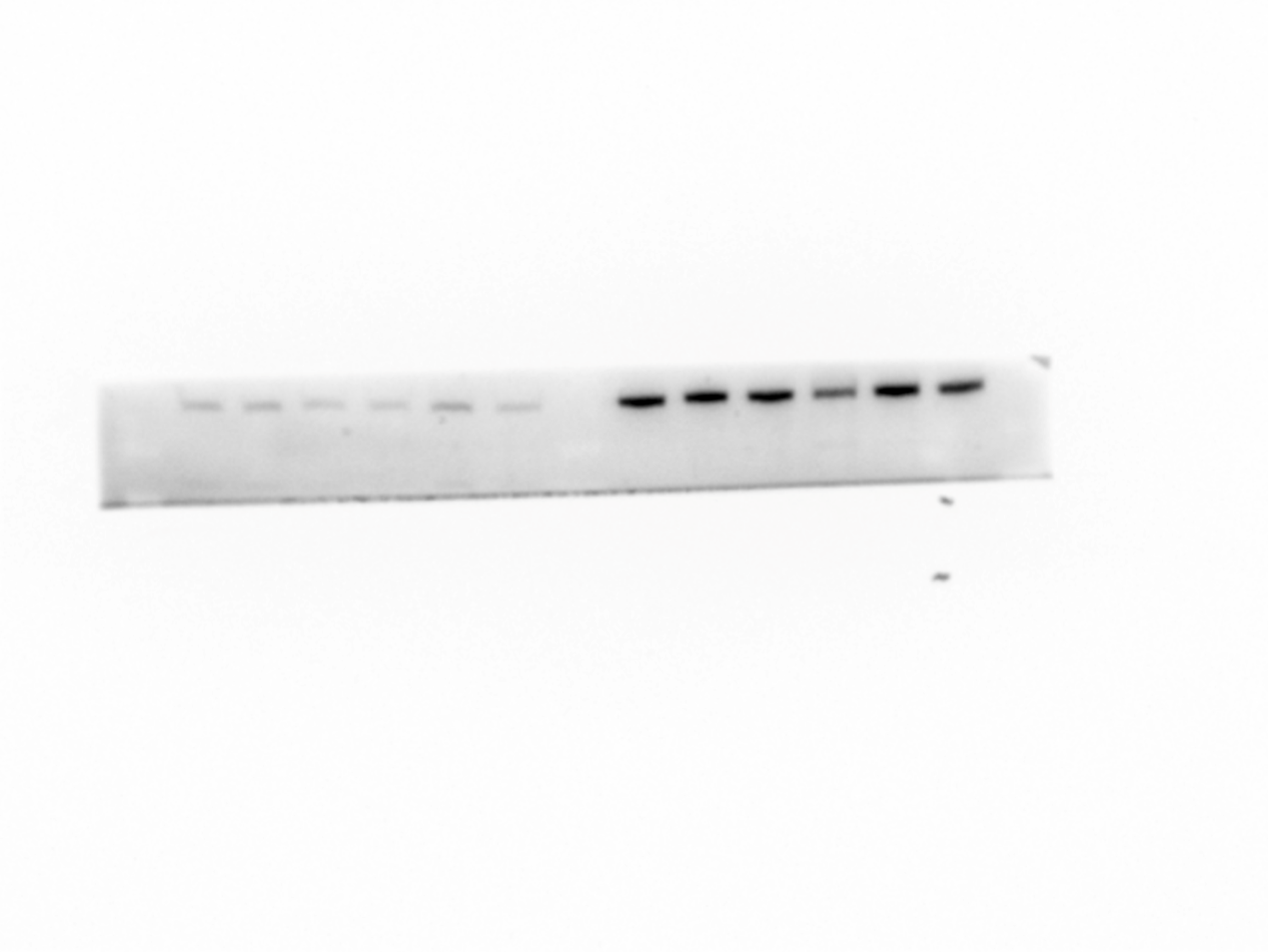


MCL-1


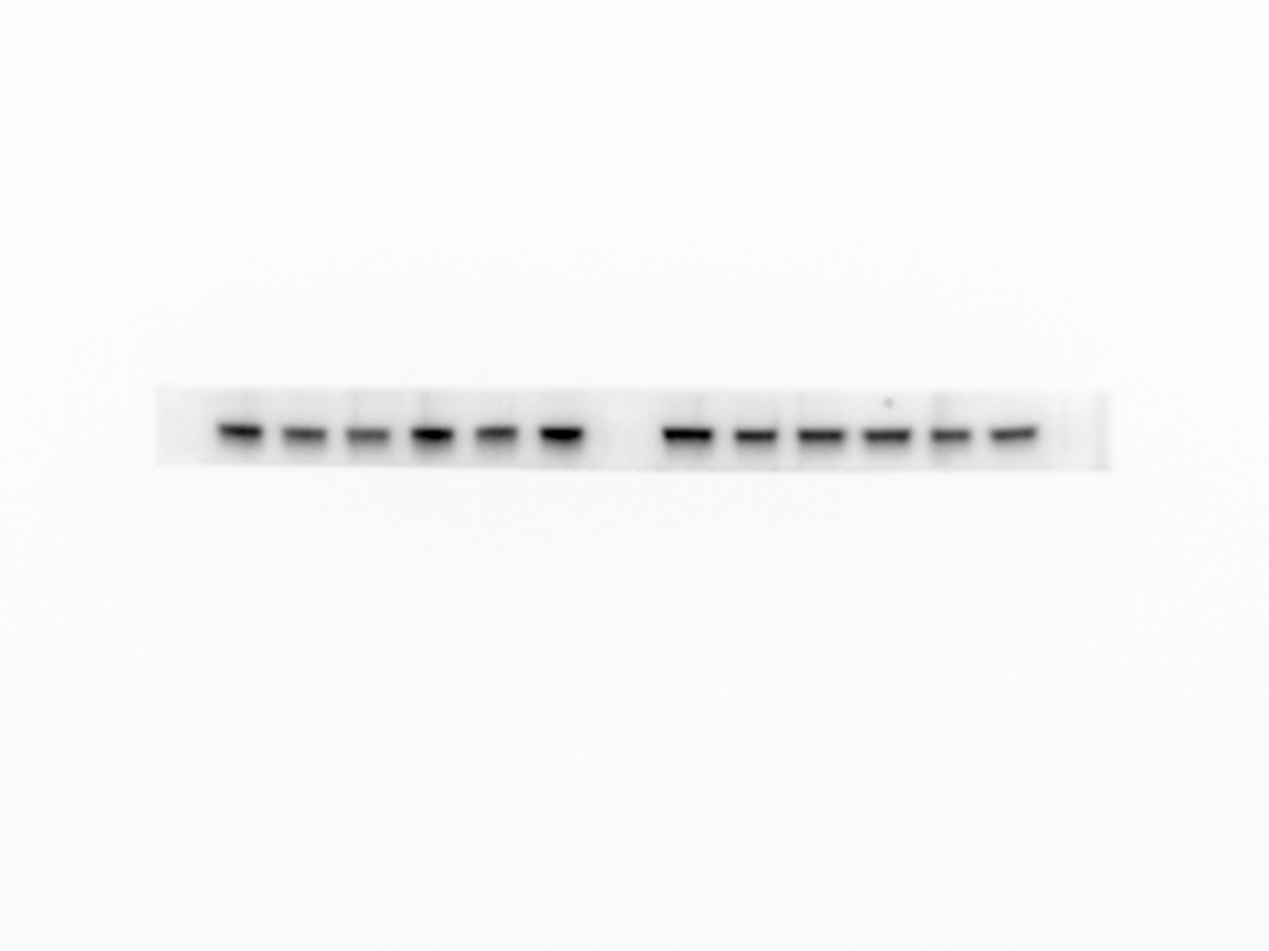


BCL-2


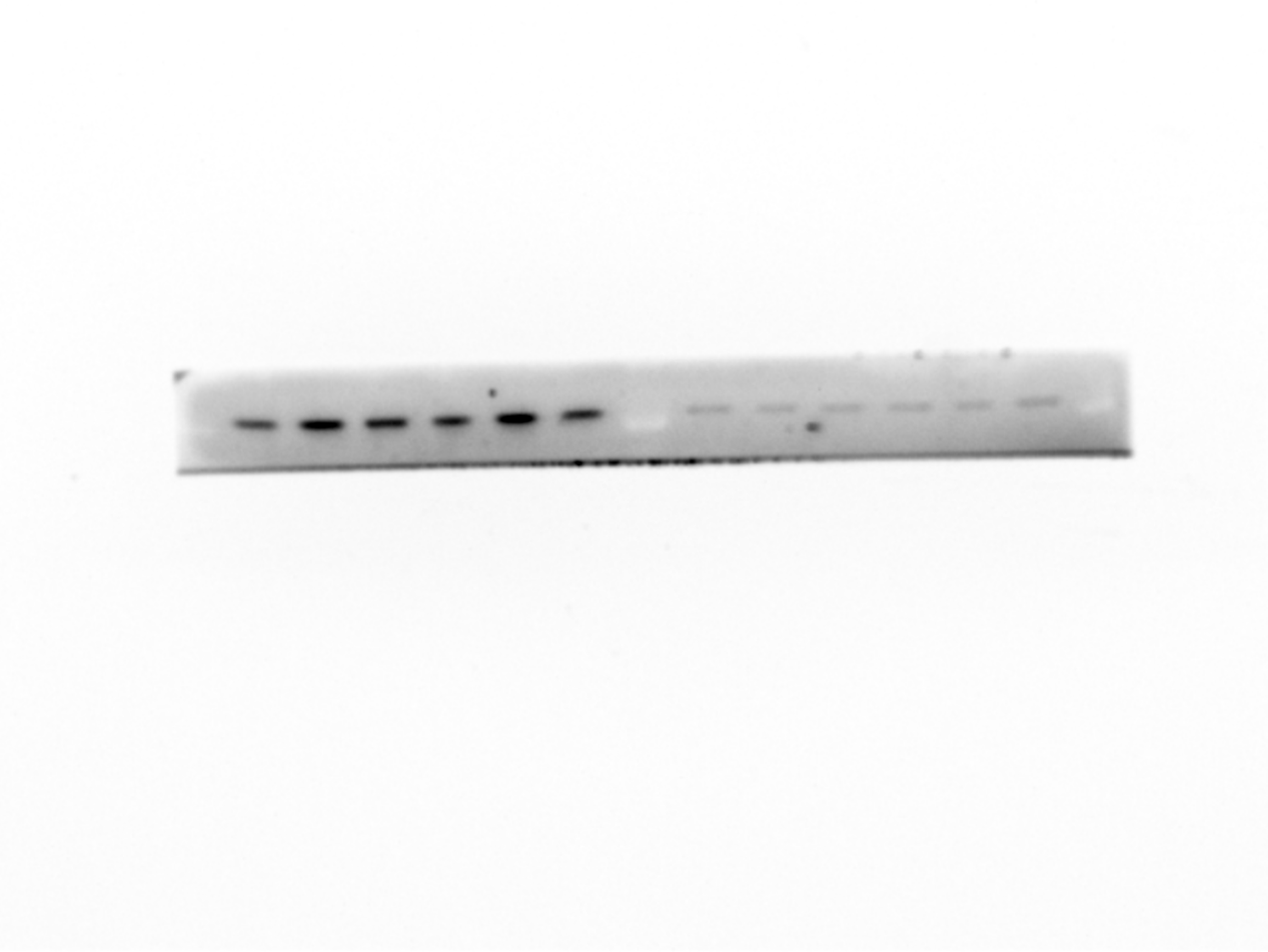


Tubulin


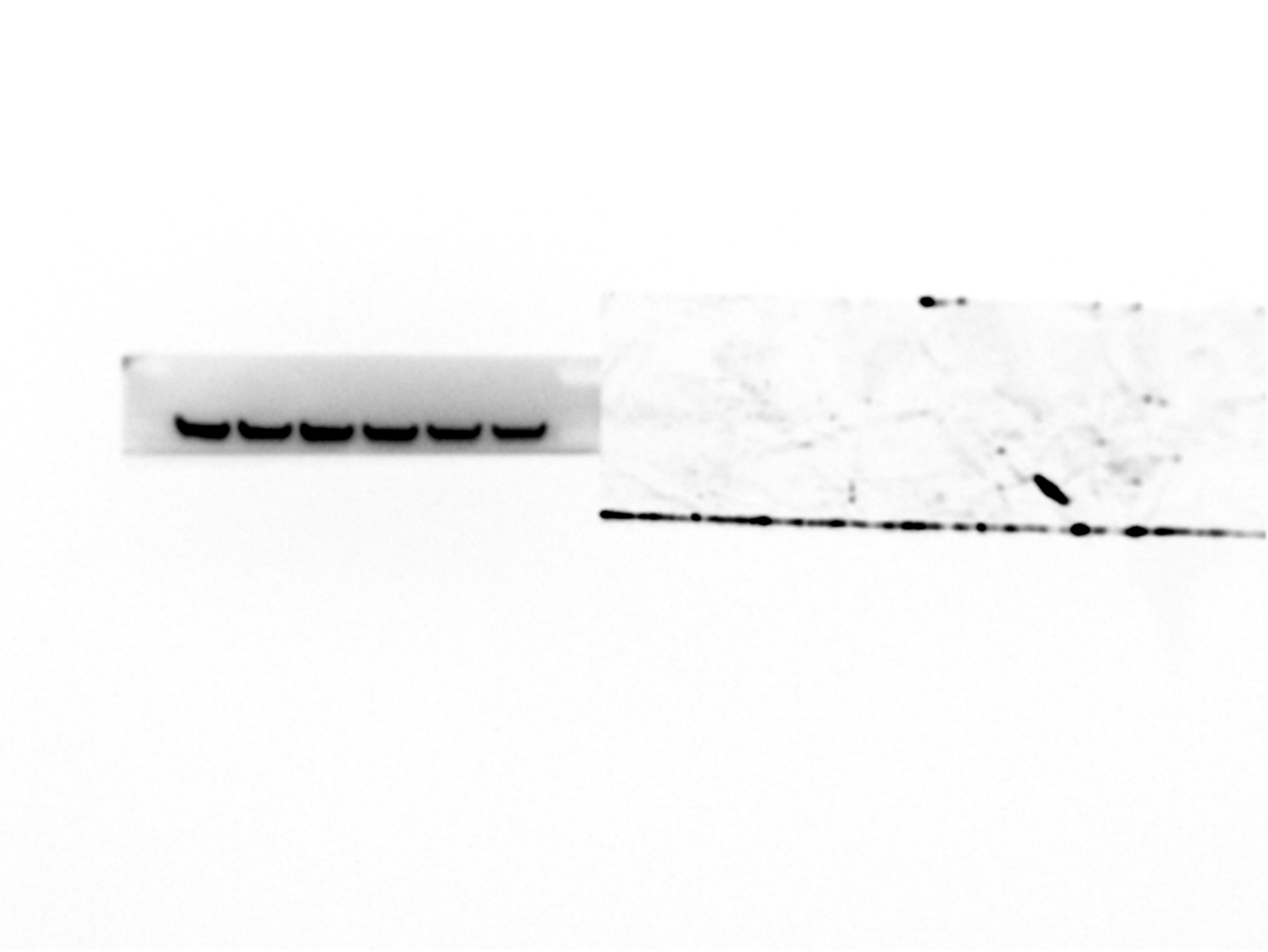


HSC3

Cleaved-PARP


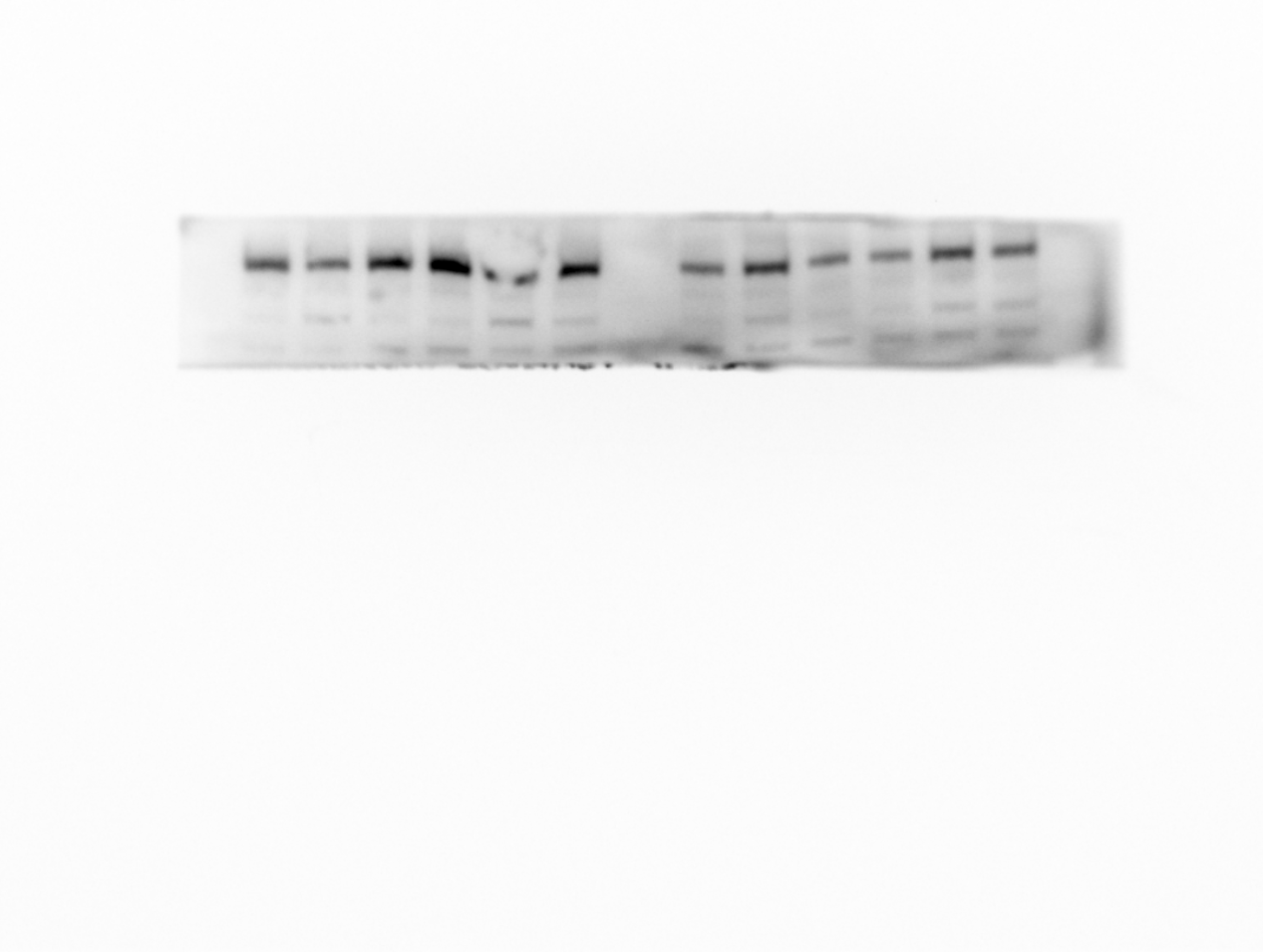


MCL-1


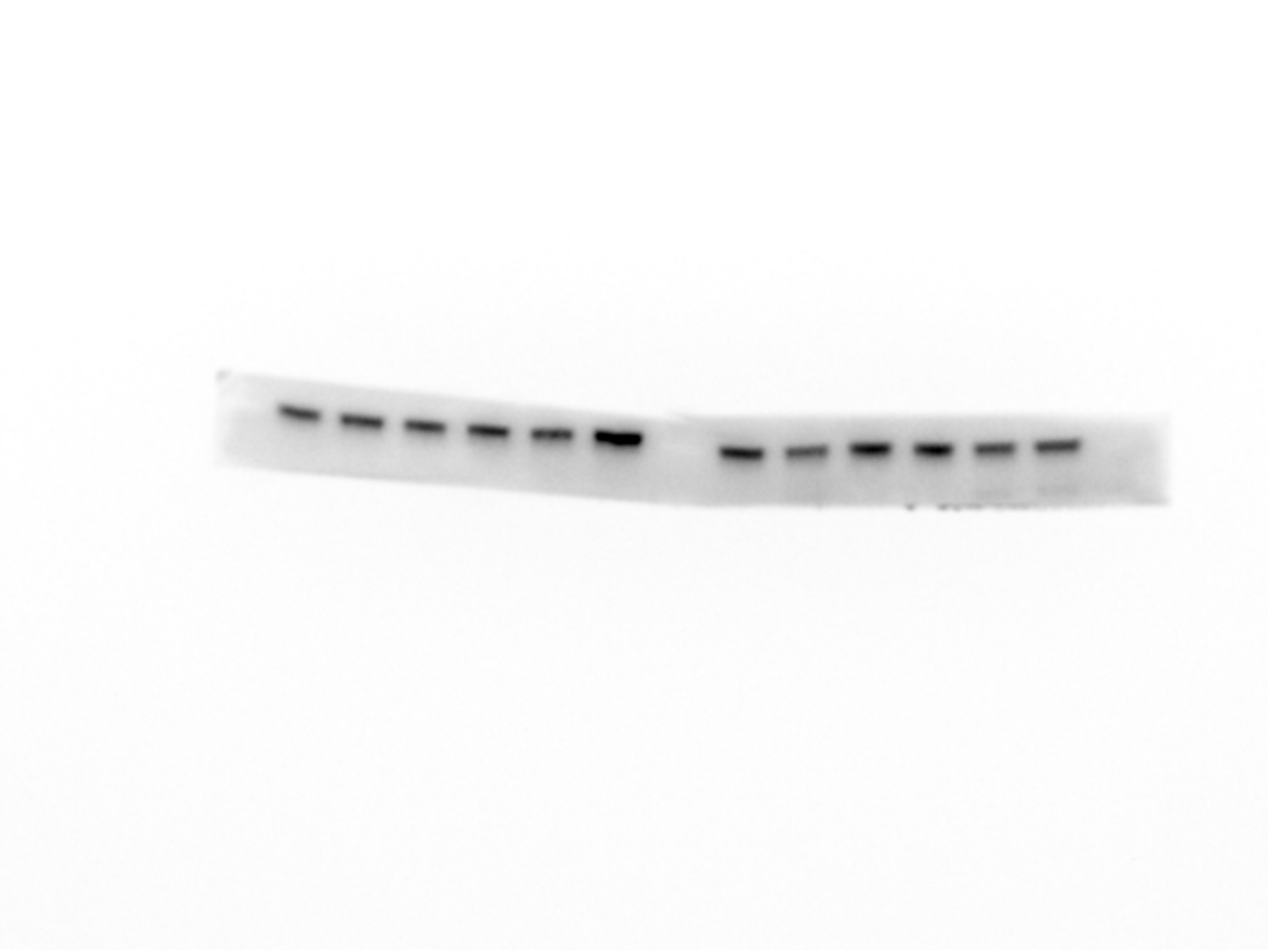


BCL-2


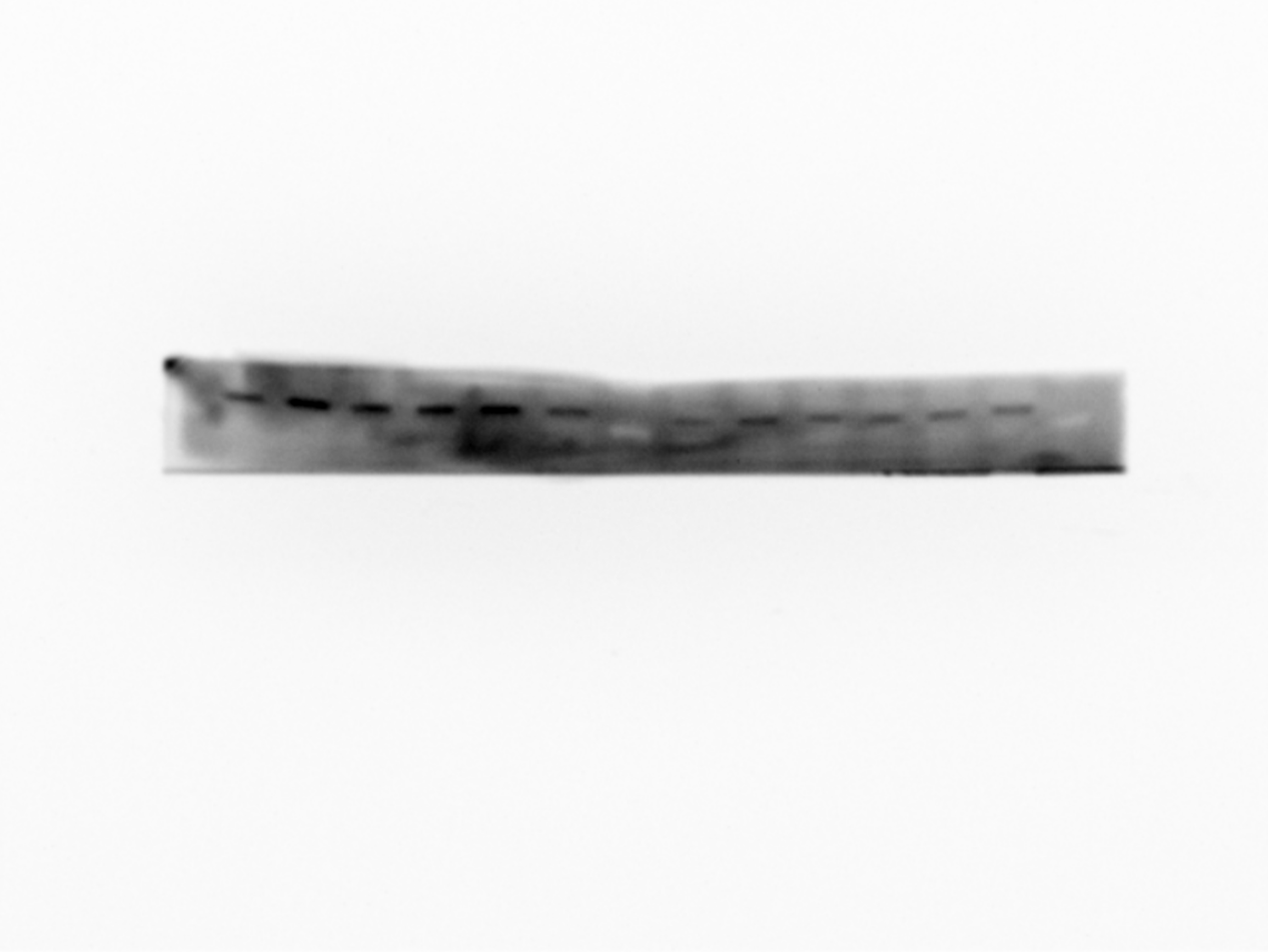


Tubulin


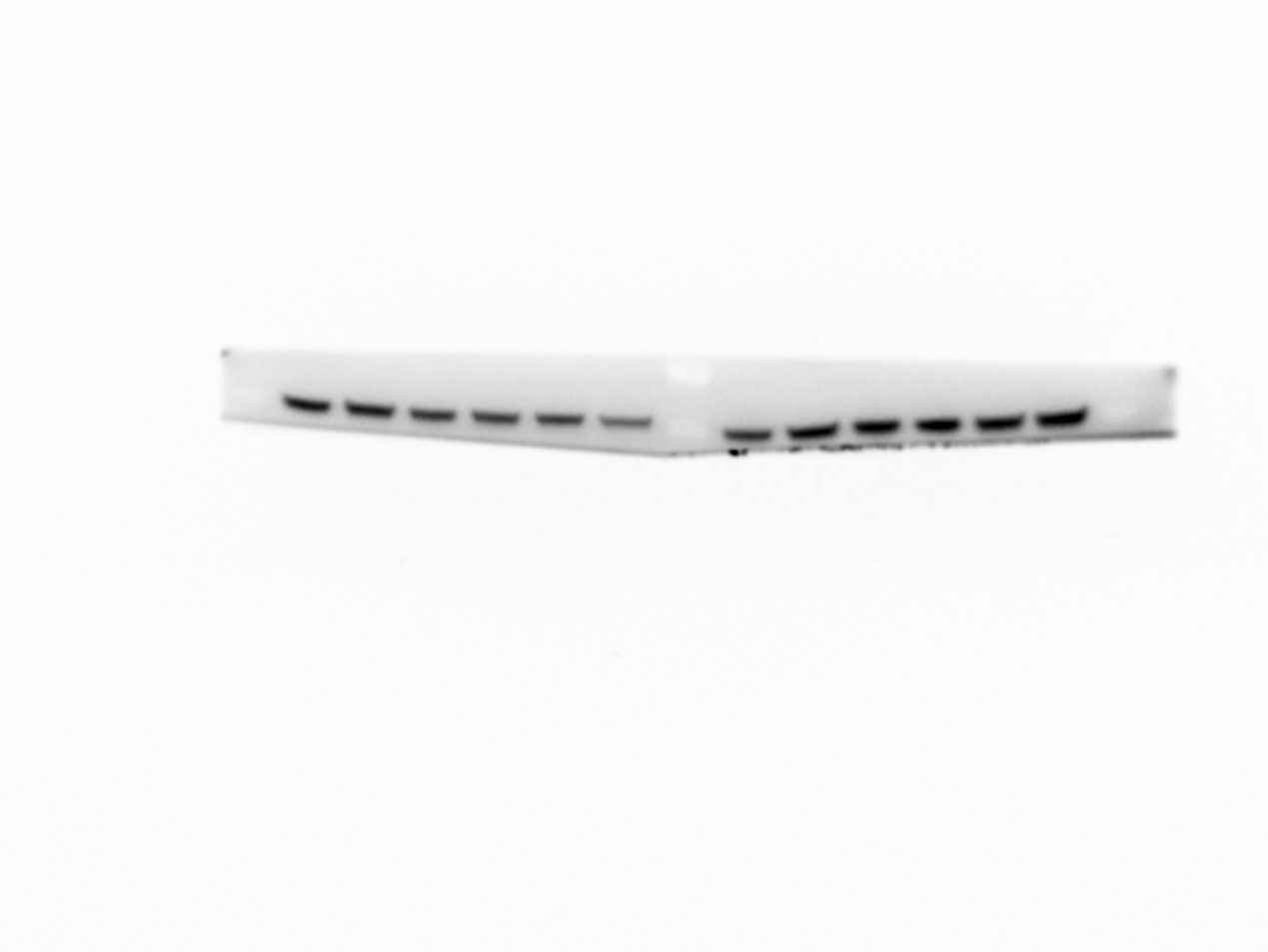

Supplement: Supplementary file 11 — Original western blots [file 41419_2025_8224_MOESM11_ESM.docx]
